# Association of mir-196a-2 rs11614913 and mir-149 rs2292832 Polymorphisms With Risk of Cancer: An Updated Meta-Analysis

**DOI:** 10.3389/fgene.2019.00186

**Published:** 2019-03-15

**Authors:** Jalal Choupani, Ziba Nariman-Saleh-Fam, Zahra Saadatian, Elaheh Ouladsahebmadarek, Andrea Masotti, Milad Bastami

**Affiliations:** ^1^Immunology Research Center, Tabriz University of Medical Sciences, Tabriz, Iran; ^2^Women's Reproductive Health Research Center, Tabriz University of Medical Sciences, Tabriz, Iran; ^3^Department of Medical Genetics, Faculty of Medicine, Shahid Beheshti University of Medical Sciences, Tehran, Iran; ^4^Research Laboratories, Bambino Gesù Children's Hospital-IRCCS, Rome, Italy; ^5^Drug Applied Research Center, Tabriz University of Medical Sciences, Tabriz, Iran; ^6^Department of Medical Genetics, Faculty of Medicine, Tabriz University of Medical Sciences, Tabriz, Iran

**Keywords:** microRNA, polymorphism, meta-analysis, cancer, mir-196a-2, mir-149

## Abstract

**Background:** Accumulating evidence suggests that functional dysregulations of miRNAs, especially miR-196a-2 and miR-149, in cancers could be attributed to polymorphisms in miRNA sequences. This study was aimed at clarifying the association of mir-196a-2 rs11614913 and mir-149 rs2292832 with cancer risk by performing an updated meta-analysis of genetic association studies.

**Methods:** PubMed, Embase, Scopus, and ScienceDirect databases were searched until 9 April 2018 to identify eligible studies. Studies should meet the following criteria to be included in the meta-analysis: evaluation of genetic association between rs11614913 and/or rs2292832 and susceptibility to cancer; A case-control design; Written in English; Availability of sufficient data for estimating odds ratio (OR) and its 95% confidence interval (95%CI). Studies that met the following criteria were excluded: review articles, meta-analysis, abstracts or conference papers; duplicate publications; studies on animals or cell-lines; studies without a case-control design; studies that did not report genotype frequencies. Pooled ORs and 95% CIs were estimated using a total of 111 studies (41,673 cases and 49,570 controls) for mir-196a rs11614913 and 44 studies (15,954 cases and 19,594 controls) for mir-149 rs2292832. Stratified analysis according to quality scores, genotyping method, ethnicity, broad cancer category and cancer type was also performed.

**Results:** Mir-196a-2 rs11614913 T allele was associated with decreased cancer risk in overall population. The association was only significant in Asians but not Caucasians. In subgroup analysis, significant associations were found in high quality studies, gynecological cancers, ovarian, breast, and hepatocellular cancer. Mir-149 rs2292832 was not associated with cancer risk in overall population and there were no differences between Asians and Caucasians. However, the T allele was associated with a decrease risk of gastrointestinal tract cancers under the heterozygote model and an increased risk of colorectal cancer under the recessive model.

**Conclusions:** The present meta-analysis suggests that mir-196a-2 rs11614913 may contribute to the risk of cancer especially in Asians. Mir-149 rs2292832 may modulate the risk of gastrointestinal tract cancers especially colorectal cancer. This study had some limitations such as significant heterogeneity in most contrasts, limited number of studies enrolling Africans or Caucasians ancestry and lack of adjustment for covariates and environmental interactions.

## Introduction

Despite remarkable recent progress in clinical management, diagnosis and treatment, cancer has remained one of the major causes of death worldwide. According to the recent World Health Organization (WHO) report, about one in six deaths were caused by cancer in 2015. It is predicted that cancer-related death will increase up to 13.2 million by 2030 worldwide (Ferlay et al., 2010; Bray et al., 2012). Complex genetic and environmental risk factors and also interactions between these components contribute to the etiopathology of different cancers. Until recent years, much effort has been devoted to link the alteration of protein coding genes to tumorigenesis. However, latest evidence has demonstrated the emerging role of noncoding RNAs in cancer development and, especially, introduced microRNAs (miRNAs) as new players in pathobiology of cancers (Peng and Croce, 2016). MiRNAs are short noncoding functional RNAs that are involved in the regulation of transcriptome (Ha and Kim, 2014). They modulate important cellular processes both in normal physiology and disease state and are involved in almost all cellular processes altered during tumorigenesis (Osada and Takahashi, 2007; Li et al., 2009). Human mir-196a (*MIR196A2*, HGNC:31568) and mir-149 (*MIR149*, HGNC: 31536) are well-studied miRNAs that may function either as oncomiRs, by targeting tumor suppressor genes, or as tumor suppressors, by targeting oncogenes, in different conditions (Lu et al., 2016; He J. et al., 2018; Ow et al., 2018). It has been shown that single nucleotide polymorphism (SNP) in miRNA genes, such as hsa-mir-196a-2 rs11614913 and hsa-mir-149 rs2292832, may influence their functions through altering miRNA expression, maturation and/or efficiency of targeting and, thereby, contribute to the risk of cancer (Hu et al., 2008; Hoffman et al., 2009; Tu et al., 2012; Nariman-Saleh-Fam et al., 2016, 2017). Several association studies in a range of populations evaluated the contribution of mir-196a-2 rs11614913 and mir-149 rs2292832 to cancer risk; but results are inconclusive. Therefore, this study was aimed at clarifying the association of mir-196a-2 rs11614913 and mir-149 rs2292832 with cancer risk by performing an updated meta-analysis of genetic association studies.

## Materials and Methods

### Publication Search

To identify all potentially eligible publications, PubMed, Embase, Scopus and ScienceDirect databases were searched, with respect to specific search tips of each database, using following keywords. (“microRNA 196a2” OR “miRNA-196a2” OR “mir-196a2” OR “mir196a” OR “mir-196a-2” OR “pre-mir-196a” OR “pre-mir196a” OR “196a” OR “rs11614913”) OR (“microRNA 149” OR “miRNA-149” OR “mir-149” OR “mir149” OR “pre-mir-149" OR “pre-mir149” OR “rs2292832”) AND (“single nucleotide polymorphism” OR “SNP” OR “variant” OR “variation” OR “polymorphism” OR “mutation” OR “locus”) AND (“neoplasm” OR “cancer” OR “tumor” OR “carcinoma” OR “sarcoma” OR “lymphoma” OR “adenoma” OR “leukemia” OR “leucemia” OR “malignancy” OR “malignance” OR “malignant” OR “glioma”). Last search was performed on 9 April 2018. References of the relevant literature and review articles were also evaluated to identify all potentially eligible articles. This meta-analysis carried out in accordance with the Preferred Reporting Items for Systematic Reviews and Meta-Analyses (PRISMA) statement (Liberati et al., 2009).

### Inclusion and Exclusion Criteria

Studies should meet the following criteria to be included in the meta-analysis: (1) evaluation of genetic association between rs11614913 and/or rs2292832 and susceptibility to cancer; (2) a case-control design; (3) Written in English; (4) Availability of sufficient data for estimating odds ratio (OR) and its 95% confidence interval (95%CI). Studies that met the following criteria were excluded: (1) review articles, meta-analysis, abstracts or conference papers; (2) duplicate publications; (3) studies on animals or cell-lines; (4) studies without a case-control design (5) studies that did not report genotype frequencies.

### Data Extraction

Data was extracted from each eligible study and manually checked. Then, items were recorded for each eligible study: the first author, publication year, category of cancer, type of cancer, country, ethnicity, source of controls, genotyping method, number of subjects in the case and the control groups, genotype counts for each SNP in the case and the control groups. A broad cancer category was assigned for each study according to the following scheme: gastrointestinal tract cancers (GI, including gastric, esophageal, colorectal, bladder, pancreatic, or hepatocellular cancers), head and neck squamous cell carcinoma (HNC, including oral, non-oral, and nasopharyngeal cancers), gynecologic cancer (GyC, including endometrial, ovarian, and cervical cancers), hematological malignancies (HM, including leukemia and lymphomas), urogenital cancers (UG, including prostate, renal cell, and bladder cancers), or other cancers.

### Quality Assessment

The quality of each study was assessed using quality assessment criteria for genetic association studies used elsewhere (Thakkinstian et al., 2011; Xue et al., 2015). This score is based on seven items including representativeness of cases, representativeness of controls, ascertainment of cancer case, control selection, genotyping examination, Hardy-Weinberg Equilibrium (HWE) status in controls, and total sample size. Quality scores ranged from 0 (lowest) to 15 (highest). Studies which were scored equal to or less than eight were regarded as low quality, while those with scores of greater than eight were regarded as high quality.

### Statistical Analysis

The Meta package for R was used to perform meta-analysis (Schwarzer, 2007). Association of rs11614913 and rs2292832 with cancer was estimated by calculating pooled ORs and their 95% CIs assuming homozygote, heterozygote, dominant, recessive, and allelic models. Heterogeneity was assessed using the Chi-squared based Q test (Lau et al., 1997). In the presence of a significant heterogeneity (i.e., *P-value* of *Q*-test < 0.05 or *I*^2^ > 50%), the random effect (RE) model (DerSimonian and Laird, 1986) was used to calculate pooled ORs and 95% CIs. Otherwise, the fixed effect (FE) model was used (Mantel and Haenszel, 1959). Significance of the pooled OR was determined by the *Z*-test (*P* < 0.05 was considered significant). In cases of remarkable heterogeneity (i.e., *I*^2^ > 50%), the potential sources of heterogeneity across studies was explored using univariate meta-regression and stratified analysis. Moreover, subgroup analyses based on genotyping method, study quality, ethnicities, broad cancer categories, and cancer types were carried out. To assess consistency of results and influence of each study on the pooled OR, sensitivity analysis was done by omitting one study at a time and recalculating summary OR and 95% CI. Publication bias was evaluated by the Begg's rank correlation test of funnel plot asymmetry (Begg and Mazumdar, 1994) the “Trim and Fill” approach was used to correct for asymmetry in cases of significant rank correlation test (Duval and Tweedie, 2000a,b). All *P-values* were two-sided and *P-value* < 0.05 was considered statistically significant. All statistical analyses were performed in R (version 3.3.1).

### Dealing With HWD (Departure From Hardy-Weinberg Equilibrium)

Departure from Hardy-Weinberg equilibrium (HWE) may be caused by a range of factors, among which genotyping error is more importantly relevant to the association study context. Currently there is no consensus on the way of handling association studies with the controls not in HWE, but it has been recommended that such studies should not be excluded from meta-analysis (Minelli et al., 2008). However, sensitivity analysis should be performed to evaluate the possible effects of such studies on the pooled estimates (Attia et al., 2003; Thakkinstian et al., 2005; Zintzaras and Lau, 2008; Wang X. B. et al., 2014). In the present meta-analysis, the following approach with regards to HWE-deviated studies was followed. Departure of genotype distributions from HWE (i.e., HWD) in the control group of each study was evaluated using the Chi-squared or the exact goodness of fit test. Meta-analyses, including the overall and subgroup analyses, were performed considering all eligible studies including HWD studies. However, to evaluate possible impacts of HWE-deviated studies, HWD sensitivity analysis was performed by evaluating the influence of excluding these studies on point estimates and identifying the influenced genotype contrasts. In cases that excluding HWD studies altered the result of meta-analysis, ORs of such studies were adjusted for HWE deviation by means of incorporating the HWE-expected genotype counts in the control group as recommended (Trikalinos et al., 2006; Zintzaras et al., 2006; Zintzaras, 2008; Zintzaras and Lau, 2008; Srivastava and Srivastava, 2012) and the HWD-adjusted pooled ORs were calculated in genotype contrasts.

## Results

### Study Characteristics

**Figure 1:**
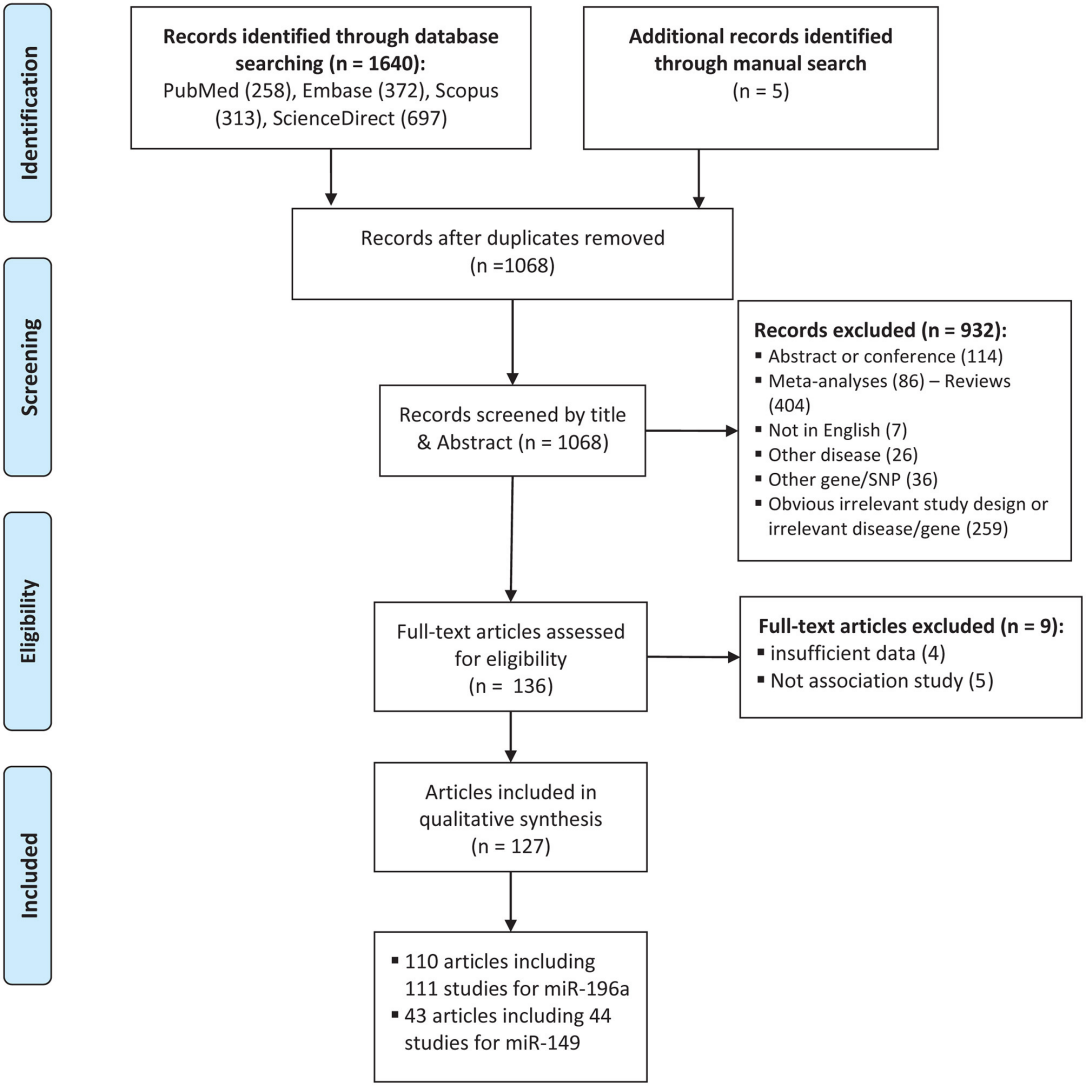
Flowchart of the identification of eligible studies for meta-analysis of cancer risk associated with mir-196a-2 rs11614913 and mir-149 rs2292832.

The process of selecting eligible studies is depicted in Figure 1. A total of 1,645 articles were found from different sources outlined in materials and methods and screened by reading titles and abstracts. A total of 1,509 articles were excluded in which 577 articles were duplicates, 114 articles were abstracts or conference meetings, 86 articles were meta-analysis, 404 were review articles, 7 articles were not written in English, 26 articles were related to other diseases, 36 articles were related to other genes, or polymorphisms and 259 more articles had either obvious irrelevant study design or irrelevant disease/gene. The full text of the remaining 136 articles were evaluated and 9 more articles were also excluded as they did not have either sufficient data to calculate ORs and 95%CIs (*n*: 4) or an association study design (*n*: 5). Finally, a total of 127 eligible articles remained (Horikawa et al., 2008; Yang et al., 2008, 2016; Hoffman et al., 2009; Hu et al., 2009, 2013; Tian et al., 2009; Catucci et al., 2010; Christensen et al., 2010; Dou et al., 2010; Kim et al., 2010, 2012; Kontorovich et al., 2010; Li et al., 2010, 2014; Liu et al., 2010, 2013, 2014; Okubo et al., 2010; Peng et al., 2010; Qi et al., 2010, 2014, 2015; Srivastava et al., 2010, 2017; Wang et al., 2010, 2013, 2016; Akkiz et al., 2011; George et al., 2011; Hong et al., 2011; Jedlinski et al., 2011; Mittal et al., 2011; Vinci et al., 2011, 2013; Zhan et al., 2011; Zhou et al., 2011, 2014; Alshatwi et al., 2012; Chen et al., 2012; Chu et al., 2012, 2014; Hezova et al., 2012; Linhares et al., 2012; Min et al., 2012; Tu et al., 2012; Zhang M. et al., 2012; Zhang M. W. et al., 2012; Zhu et al., 2012; Ahn et al., 2013; Han et al., 2013; Huang et al., 2013, 2017; Lv et al., 2013; Ma et al., 2013; Pavlakis et al., 2013; Umar et al., 2013; Wei et al., 2013, 2014; Zhang et al., 2013; Bansal et al., 2014; Dikeakos et al., 2014; Du et al., 2014; Hao et al., 2014; Kou et al., 2014; Kupcinskas et al., 2014a,b; Omrani et al., 2014; Parlayan et al., 2014; Pu et al., 2014; Qu et al., 2014; Roy et al., 2014; Tong et al., 2014; Wang N. et al., 2014; Wang R. et al., 2014; Wang X. H. et al., 2014; Deng et al., 2015; Dikaiakos et al., 2015; Dong et al., 2015; He et al., 2015; Li T. et al., 2015; Liu, 2015; Li X. et al., 2015; Martin-Guerrero et al., 2015; Nikolić et al., 2015; Pratedrat et al., 2015; Sodhi et al., 2015; Sushma et al., 2015; Yan et al., 2015; Yin et al., 2015, 2016, 2017; Dai et al., 2016; Gu and Tu, 2016; Hashemi et al., 2016; Jiang et al., 2016; Li H. et al., 2016; Li J. et al., 2016; Li M. et al., 2016; Miao et al., 2016; Morales et al., 2016; Ni and Huang, 2016; Peckham-Gregory et al., 2016; Shen et al., 2016; Song et al., 2016; Su et al., 2016; Sun et al., 2016; Toraih et al., 2016a,b; Zhang L. H. et al., 2016; Zhao et al., 2016; Afsharzadeh et al., 2017; Bodal et al., 2017; Cîmpeanu et al., 2017; Poltronieri-Oliveira et al., 2017; Rakmanee et al., 2017; Rogoveanu et al., 2017; Tandon et al., 2017; Zhang E. et al., 2017; Abdel-Hamid et al., 2018; Damodaran et al., 2018; Doulah et al., 2018; He J. et al., 2018; He Y. et al., 2018; Mashayekhi et al., 2018; Minh et al., 2018; Ranjbar et al., 2018). Characteristics of the included studies are tabulated in Tables 1, 2.

**Table 1:** Main characteristics of studies evaluating cancer risk associated with miR-196a-2 rs11614913 C/T which included in the current meta-analysis.

***N***	**References**	**Country**	**Ethnicity**	**Genotyping Method**	**Design**	**Cases/** **Controls**	**Genotype distribution** ^**a**^		**Cancer Type**	**Quality Score**	***P*_**HWE**_**
							**Cases**	**Controls**			
1	Abdel-Hamid et al., 2018	Egypt	African	PCR-RFLP	PB	50/50	21/26/3	24/20/6	HCC	10	0.53
2	Damodaran et al., 2018	India	Asian	PCR-RFLP	HB	100/100	32/51/17	47/36/17	PC	7	0.054
3	Doulah et al., 2018	Iran	Asian	ARMS-PCR	HB	98/100	33/51/14	25/62/13	BC	3	0.015
4	He J. et al., 2018	China	Asian	TaqMan	HB	393/812	94/192/107	183/399/230	NB	11	0.73
5	Mashayekhi et al., 2018	Iran	Asian	PCR-RFLP	HB	353/353	142/169/42	149/158/46	BC	10	0.75
6	Minh et al., 2018	Vietnam	Vietnamese	HRMA	HB	113/127	48/35/30	31/64/32	BC	5	0.95
7	Afsharzadeh et al., 2017	Iran	Asian	ARMS-PCR	Not mentioned	100/150	34/52/14	38/93/19	BC	3	0.002
8	Bodal et al., 2017	India	Asian	PCR-RFLP	HB	95/99	48/47/0	64/35/0	BC	8	0.051
9	Huang et al., 2017	China	Asian	PCR-RFLP	PB	165/284	22/81/62	39/134/111	HCC	9	0.95
10	Poltronieri-Oliveira et al., 2017	Brazil	Mixed	PCR-RFLP	PB	149/246	64/57/28	105/120/21	GC	9	0.12
11	Rakmanee et al., 2017	Thiland	Asian	PCR-RFLP	HB	104/180	48/43/13	49/78/53	ALL	9	0.09
12	Rogoveanu et al., 2017	Romania	Caucasian	TaqMan	HB	142/288	61/63/18	121/128/39	GC	9	0.64
13	Srivastava et al., 2017	India	Asian	PCR-RFLP	HB	184/164	20/93/71	21/81/62	CSCC	1-	0.57
14	Yin et al., 2017	China	Asian	TaqMan	HB	1,003/1,003	252/555/196	221/496/286	LC	1-	0.86
15	Zhang E. et al., 2017	China	Asian	TaqMan	HB	340/340	71/169/100	88/155/97	OC	11	0.12
16	Dai et al., 2016	China	Asian	Mass-ARRAY	HB	560/583	197/265/98	155/284/144	BC	10	0.58
17	Gu and Tu, 2016	China	Asian	PCR-RFLP	HB	186/186	39/96/51	57/98/31	GC	7	0.36
18	Hashemi et al., 2016	Iran	Asian	t-ARMS-PCR	HB	169/182	64/88/17	77/93/12	PC	7	0.029
19	Li M. et al., 2016	China	Asian	MassARRAY	HB	182/182	24/83/75	11/79/92	GC	8	0.33
20	Li J. et al., 2016	China	Asian	Sequencing	Not mentioned	109/105	25/64/20	18/52/35	HCC	8	0.95
21	Miao et al., 2016	China	Asian	Array (Illumina Infinium1)	HB	576/1,550	130/284/162	292/755/503	HNSCC	12	0.80
22	Morales et al., 2016	Chile	Mixed	TaqMan	PB	440/807	192/191/57	342/351/114	BC	12	0.13
23	Ni and Huang, 2016	China	Asian	PCR-RFLP	HB	155/342	32/82/41	66/176/100	OvC	9	0.51
24	Peckham-Gregory et al., 2016	USA	Caucasian	Allele-specific PCR Array	PB	179/529	72/88/19	196/257/76	NHL	13	0.62
25	Shen et al., 2016	China	Asian	SNaPshot	PB	1,400/2,185	295/698/407	392/1121/672	ESCC	10	0.046
26	Song et al., 2016	China	Asian	PCR-RFLP	PB	479/431	121/247/111	86/203/142	OvC	13	0.42
27	Su et al., 2016	China	Asian	PCR-RFLP	HB	245/315	83/128/34	119/158/38	GC	10	0.22
28	Sun et al., 2016	China	Asian	PCR-RFLP	HB	134/227	29/66/39	34/116/77	OvC	7	0.42
29	Toraih et al., 2016b	Egypt	African	TaqMan	HB	209/100	84/93/32	55/35/10	Cancer	8	0.30
30	Toraih et al., 2016a	Egypt	African	TaqMan	PB	125/150	48/63/14	80/53/17	Cancer	11	0.11
31	Wang et al., 2017	China	Asian	Mass-ARRAY	PB	283/283	73/158/52	65/124/94	BlC	10	0.066
32	Yin et al., 2016	China	Asian	TaqMan	HB	575/608	128/298/149	133/297/178	LC	14	0.70
33	Zhang L. H. et al., 2016	China	Asian	PCR-RFLP	PB	175/302	25/85/65	42/138/122	HCC	9	0.83
34	Zhao et al., 2016	China	Asian	Sequencing	HB	114/114	31/50/33	28/61/25	BC	9	0.53
35	Zhou et al., 2011	China	Asian	PCR-RFLP	HB	226/309	46/123/57	58/169/82	CSCC	12	0.09
36	Deng et al., 2015	China	Asian	PCR-RFLP	PB	159/298	41/66/52	56/166/76	BlC	9	0.048
37	Dikaiakos et al., 2015	Greece	Caucasian	PCR-RFLP	PB	157/299	19/69/69	33/149/117	CRC	10	0.18
38	He et al., 2015	China	Asian	Mass-ARRAY	PB	450/450	81/233/136	93/223/134	BC	11	0.94
39	Li T. et al., 2015	China	Asian	PCR-RFLP	PB	318/320	61/146/111	42/134/144	NHL	13	0.26
40	Liu, 2015	China	Asian	PCR-RFLP	PB	266/266	84/131/51	113/123/30	HCC	12	0.76
41	Liu, 2015	China	Asian	PCR-RFLP	HB	216/100	25/133/58	23/49/28	GyC	5	0.95
42	Martin-Guerrero et al., 2015	Spain	Caucasian	TaqMan	PB	104/345	35/40/29	137/159/49	CLL	9	0.86
43	Nikolić et al., 2015	Serbia	Caucasian	HRMA	PB	351/309	150/161/40	121/147/41	PC	10	0.79
44	Qi et al., 2015	China	Asian	TaqMan	PB	321/290	34/119/168	17/88/185	BC	11	0.18
45	Sodhi et al., 2015	India	Asian	PCR-RFLP	PB	250/255	70/161/19	101/146/8	LC	12	5.46*e*−7
46	Sushma et al., 2015	India	Asian	PCR-RFLP	PB	100/102	22/10/68	6/15/81	OC	8	0.001
47	Yan et al., 2015	China	Asian	PCR-RFLP	HB	274/328	46/147/81	27/165/136	HCC	9	0.02
48	Yin et al., 2015	China	Asian	TaqMan	HB	258/310	50/141/67	63/150/97	LC	11	0.78
49	Bansal et al., 2014	India	Asian	PCR-RFLP	PB	121/165	68/41/12	85/59/21	BC	9	0.058
50	Chu et al., 2014	Taiwan	Asian	PCR-RFLP	PB	188/337	41/81/66	70/167/100	HCC	12	0.94
51	Dikeakos et al., 2014	Greece	Caucasian	PCR-RFLP	HB	163/480	102/46/15	79/229/172	GC	9	0.9
52	Hao et al., 2014	China	Asian	PCR-RFLP	PB	235/282	77/126/32	67/160/55	HCC	11	0.027
53	Kou et al., 2014	China	Asian	PCR-RFLP	PB	271/532	84/150/37	125/304/103	HCC	9	0.001
54	Kupcinskas et al., 2014b	Latvia	Caucasian	TaqMan	HB	363/350	144/184/35	159/145/46	GC	8	0.18
55	Kupcinskas et al., 2014a	Latvia	Caucasian	TaqMan	HB	193/427	79/87/27	199/174/54	CRC	8	0.12
56	Li et al., 2014	China	Asian	TaqMan	PB	1,020/1,006	209/489/322	218/518/270	NC	13	0.32
57	Du et al., 2014	China	Asian	TaqMan	PB	1,000/1,022	149/514/337	211/497/314	RCC	13	0.61
58	Omrani et al., 2014	Iran	Asian	T-ARMS-PCR	PB	236/203	218/18/0	178/25/0	BC	11	0.70
59	Parlayan et al., 2014	Japan	Asian	TaqMan	HB	827/524	174/409/244	108/270/146	Cancer	11	0.44
60	Pu et al., 2014	China	Asian	PCR-RFLP	HB	159/511	39/95/25	101/324/86	GC	9	1.7*e*−9
61	Qi et al., 2014	China	Asian	HRMA	PB	314/406	45/209/60	71/214/121	HCC	12	0.17
62	Qu et al., 2014	China	Asian	PCR-RFLP	PB	381/426	126/207/48	133/211/82	ESCC	13	0.96
63	Roy et al., 2014	India	Asian	TaqMan	HB	451/448	218/187/46	242/168/38	OC	10	0.29
64	Tong et al., 2014	China	Asian	TaqMan	PB	570/673	103/308/159	129/307/237	ALL	14	0.11
65	Wang N. et al., 2014	China	Asian	PCR-LDR	PB	597/597	128/307/162	145/298/154	ESCC	13	0.97
66	Zhou et al., 2014	China	Asian	PCR-RFLP	HB	266/281	93/139/34	66/160/55	HCC	10	0.02
67	Ahn et al., 2013	South Korea	Asian	PCR-RFLP	PB	461/447	100/242/119	87/232/128	GC	12	0.35
68	Han et al., 2013	China	Asian	TaqMan	PB	1,017/1,009	207/505/305	220/485/304	HCC	10	0.33
69	Hu et al., 2013	China	Asian	Sequencing	HB	680/690	185/314/181	138/342/210	Glioma	14	0.98
70	Liu et al., 2013	Taiwan	Asian	PCR-RFLP	NA	315/92	64/147/104	26/36/30	OC	6	0.055
71	Lv et al., 2013	China	Asian	PCR-RFLP	PB	347/531	10/223/114	109/331/91	CRC	10	1.5*e*−8
72	Ma et al., 2013	China	Asian	Mass-ARRAY	PB	190/187	44/92/54	49/79/59	BC	11	0.047
73	Min et al., 2012	South Korea	Asian	PCR-RFLP	PB	446/502	120/201/125	100/254/148	CRC	12	0.68
74	Pavlakis et al., 2013	Greece	Caucasian	PCR-RFLP	PB	93/122	12/33/48	14/58/50	PNC	10	0.75
75	Umar et al., 2013	India	Asian	PCR-RFLP	PB	289/309	146/121/22	171/122/16	ESCC	12	0.39
76	Vinci et al., 2013	Italy	Caucasian	HRMA	HB	160/178	62/86/12	83/84/11	CRC	9	0.11
77	Wang et al., 2013	China	Asian	TaqMan	HB	1,689/1,946	319/851/519	482/940/524	GC	10	0.14
78	Wei et al., 2013	China	Asian	Mass-ARRAY	HB	367/370	65/196/106	87/170/113	ESCC	12	0.16
79	Zhang et al., 2013	China	Asian	Mass-ARRAY	PB	996/995	214/488/294	165/502/328	HCC	10	0.26
80	Alshatwi et al., 2012	Saudi Arabia	Asian	TaqMan	HB	100/100	35/63/2	46/50/4	BC	4	0.051
81	Chen et al., 2012	China	Asian	PCR-LDR	HB	126/407	27/64/35	94/206/107	CRC	12	0.84
82	Chu et al., 2012	Taiwan	Asian	PCR-RFLP	PB	470/425	57/277/136	87/206/132	OC	11	0.68
83	Hezova et al., 2012	Czech Republic	Caucasian	TaqMan	PB	197/212	82/89/26	87/103/22	CRC	9	0.34
84	Kim et al., 2012	South Korea	Asian	PCR-RFLP	PB	159/201	34/84/41	45/107/49	HCC	11	0.41
85	Linhares et al., 2012	Brazil	Mixed	TaqMan	HB	388/388	94/177/117	127/165/96	BC	7	0.005
86	Zhang M. et al., 2012	China	Asian	PCR-RFLP	PB	248/243	11/89/148	17/93/133	BC	13	0.96
87	Zhu et al., 2012	China	Asian	TaqMan	HB	573/588	140/303/130	121/295/172	CRC	12	0.83
88	Akkiz et al., 2011	Turkey	Asian	PCR-RFLP	HB	185/185	77/86/22	58/87/40	HCC	12	0.56
89	George et al., 2011	India	Asian	PCR-RFLP	PB	159/230	55/101/3	106/114/10	PC	10	0.003
90	Hong et al., 2011	South Korea	Asian	TaqMan	HB	406/428	86/224/96	96/198/134	LC	12	0.18
91	Jedlinski et al., 2011	Australia	Caucasian	PCR-RFLP	PB	187/171	68/86/33	58/82/31	BC	9	0.91
92	Mittal et al., 2011	India	Asian	PCR-RFLP	HB	212/250	76/131/5	109/127/14	BlC	10	0.004
93	Vinci et al., 2011	Italy	Caucasian	HRMA	HB	101/129	35/54/12	58/61/10	LC	9	0.34
94	Zhan et al., 2011	China	Asian	PCR-RFLP	HB	252/543	68/128/56	113/267/163	CRC	11	0.89
95	Catucci et al., 2010	Italy	Caucasian	TaqMan	PB	751/1243	334/330/87	532/550/161	BC	11	0.33
		Germany	Caucasian	TaqMan	PB	1,101/1,496	432/512/157	584/696/216	BC	11	0.74
96	Christensen et al., 2010	USA	Caucasian	TaqMan	PB	484/555	182/224/78	188/279/88	HNSCC	14	0.39
97	Dou et al., 2010	China	Asian	PCR-LDR	HB	643/656	111/343/189	143/305/208	Glioma	11	0.13
98	Li et al., 2010	China	Asian	PCR-RFLP	HB	310/222	78/150/82	42/102/78	HCC	9	0.46
99	Liu et al., 2010	USA	Caucasian	PCR-RFLP	HB	1,109/1,130	350/565/194	383/545/202	HNSCC	13	0.77
100	Okubo et al., 2010	Japan	Asian	PCR-RFLP	HB	552/697	105/281/166	124/350/223	GC	11	0.54
101	Peng et al., 2010	China	Asian	PCR-RFLP	HB	213/213	76/94/43	56/107/50	GC	10	0.96
102	Qi et al., 2010	China	Asian	PCR-LDR	PB	361/391	82/179/100	92/197/102	HCC	10	0.92
103	Srivastava et al., 2010	India	Asian	PCR-RFLP	PB	230/230	119/95/16	136/75/19	GBC	12	0.09
104	Wang et al., 2010	China	Asian	SNaPshot	HB	458/489	148/262/48	128/250/111	ESCC	11	0.64
105	Kim et al., 2010	Korea	Asian	PCR-RFLP	PB	654/640	187/305,162	155/300/185	LC	14	0.14
106	Hoffman et al., 2009	USA	Caucasian	Mass-ARRAY	HB	426/466	181/209/36	166/229/71	BC	9	0.63
107	Hu et al., 2009	China	Asian	PCR-RFLP	PB	1,009/1,093	239/483/287	218/517/358	BC	14	0.22
108	Tian et al., 2009	China	Asian	PCR-RFLP	PB	1,058/1,035	253/512/293	209/519/307	LC	14	0.73
109	Horikawa et al., 2008	USA	Caucasian	Capillary electrophoresis	PB	276/277	105/126/45	101/117/59	RCC	11	0.03
110	Yang et al., 2008	USA	Caucasian	Capillary electrophoresis	PB	736/731	255/348/133	257/342/132	BlC	13	0.35

*Genotype distributions are sorted as CC/CT/TT*.

a *Genotype distributions are sorted as CC/CT/TT. PB, population-based design; HB, hospital-based design; NC, Not clear; ALL, Acute myeloblastic leukemia; BC, Breast cancer; BlC, Bladder cancer; CLL, Chronic lymphoblastic leukemia; CRC, Colorectal cancer; CSCC, Cervical Squamous Cell Carcinoma; ESCC, Esophageal squamous cell carcinoma; GBC, Gallbladder carcinoma; GC, Gastric cancer; HCC, Hepatocellular carcinoma; HNSCC, Head and neck squamous cell carcinoma; LC, Lung cancer; NB, Neuroblastoma; NC, Nasopharyngeal carcinoma; NHL, non-Hodgkin lymphoma; OC, Oral cancer; OvC, Ovarian cancer; PC, Prostate cancer; PNC, Pancreatic cancer; RCC, Renal cell carcinoma*.

**Table 2:** Main characteristics of studies evaluating cancer risk associated with miR-149 rs2292832 C/T which included in the current meta-analysis.

***N***	**References**	**Country**	**Ethnicity**	**Genotyping method**	**Design**	**Cases/Controls**	**Genotype distribution** ^a^		**Cancer type**	**Quality score**	***P*_**HWE**_**
							**Cases**	**Controls**			
1	He J. et al., 2018	China	Asian	TaqMan	HB	380/791	32/62/286	59/172/560	NB	10	2.98e−14
2	Ranjbar et al., 2018	Iran	Asian	PCR-RFLP	PB	73/70	8/14/51	4/24/42	CRC	8	0.92
3	Cîmpeanu et al., 2017	Romania	Caucasian	TaqMan	HB	142/288	70/49/23	126/120/42	GC	10	0.15
4	Tandon et al., 2017	India	Asian	PCR-RFLP	HB	200/200	140/37/23	174/16/10	OC	9	7.96e−12
5	Gu and Tu, 2016	China	Asian	PCR-RFLP	HB	186/186	55/89/42	60/87/39	GC	7	0.54
6	Hashemi et al., 2016	Iran	Asian	t-ARMS-PCR	HB	169/182	77/68/24	101/57/24	PC	6	0.002
7	Jiang et al., 2016	China	Asian	MassARRAY	PB	869/955	65/164/640	66/204/685	GC	12	8.88e−16
8	Li H. et al., 2016	China	Asian	TaqMan	PB	555/395	49/252/254	42/176/177	LC	13	0.92
9	Miao et al., 2016	China	Asian	Array	HB	575/1,548	61/231/283	175/647/726	HNSCC	12	0.10
10	Ni and Huang, 2016	China	Asian	PCR-RFLP	HB	155/342	47/82/26	108/179/55	OvC	9	0.20
11	Su et al., 2016	China	Asian	PCR-RFLP	HB	245/315	108/112/25	149/140/26	GC	10	0.44
12	Yang et al., 2016	China	Asian	PCR-RFLP	PB	30/98	3/16/11	9/49/40	KS	9	0.35
13	Dong et al., 2015	China	Asian	MassARRAY	PB	369/751	63/175/131	91/339/321	PTC	12	0.96
14	He et al., 2015	China	Asian	Mass-ARRAY	PB	450/450	36/183/231	60/188/202	BC	11	0.14
15	Liu, 2015	China	Asian	PCR-RFLP	PB	266/266	91/130/45	108/124/34	HCC	12	0.76/
16	Pratedrat et al., 2015	Thailand	Asian	TaqMan	HB	104/95	11/27/66	9/24/62	HCC	8	0.018
17	Sushma et al., 2015	India	Asian	PCR-RFLP	PB	100/102	72/22/6	90/12/0	OC	9	0.79
18	Yan et al., 2015	China	Asian	PCR-RFLP	HB	274/328	66/133/75	72/156/100	HCC	10	0.50
19	Chu et al., 2014	Taiwan	Asian	PCR-RFLP	PB	188/337	13/36/139	27/64/246	HCC	11	8.9e-10
20	Dikeakos et al., 2014	Greece	Caucasian	PCR-RFLP	HB	163/480	21/73/69	33/198/249	GC	9	0.50
21	Du et al., 2014	China	Asian	TaqMan	PB	355/362	47/163/145	37/148/177	RCC	12	0.52
22	Kou et al., 2014	China	Asian	PCR-RFLP	PB	270/532	113/122/35	202/253/77	HCC	10	0.93
23	Liu et al., 2014	China	Asian	PCR-RFLP	PB	327/327	84/143/100	56/138/133	HCC	11	0.066
24	Pu et al., 2014	China	Asian	PCR-RFLP	HB	187/459	22/31/134	48/103/308	GC	9	8.5e-13
25	Wang R. et al., 2014	China	Asian	MassARRAY	HB	772/717	83/318/371	56/309/352	HCC	9	0.33
26	Wang X. H. et al., 2014	China	Asian	PCR-RFLP	PB	152/304	13/72/67	43/148/113	HCC	11	0.68
27	Wei et al., 2014	China	Asian	MassARRAY	PB	838/1,006	105/354/379	86/424/496	PTC	14	0.77
28	Ahn et al., 2013	South Korea	Asian	PCR-RFLP	PB	461/447	44/176/241	40/187/220	GC	12	0.94
29	Hu et al., 2013	China	Asian	Sequencing	HB	680/690	70/297/313	78/302/310	Glioma	14	0.78
30	Huang et al., 2013	China	Asian	PCR-RFLP	PB	158/242	22/67/69	39/113/90	NPC	11	0.8
31	Lv et al., 2013	China	Asian	PCR-RFLP	PB	347/459	30/64/253	48/103/308	CRC	10	8.5e-13
32	Min et al., 2012	South Korea	Asian	PCR-RFLP	PB	446/502	48/177/221	51/219/232	CRC	12	0.97
33	Vinci et al., 2013	Italy	Caucasian	HRMA	HB	160/178	79/58/23	86/75/17	CRC	9	0.95
34	Chu et al., 2012	Taiwan	Asian	PCR-RFLP	PB	470/425	37/88/345	26/84/315	OC	10	1.1e-7
35	Kim et al., 2012	South Korea	Asian	PCR-RFLP	PB	159/201	14/64/81	21/97/83	HCC	11	0.40
36	Tu et al., 2012	Taiwan	Asian	PCR-RFLP	PB	273/122	20/129/124	21/52/49	HNSCC	7	0.33
37	Zhang M. et al., 2012	China	Asian	PCR-RFLP	PB	245/229	23/102/120	24/113/92	BC	13	0.25
38	Zhang M. W. et al., 2012	China	Asian	PCR-RFLP	PB	443/435	50/190/203	46/202/187	CRC	14	0.48
					PB	274/269	41/101/132	35/120/114	GC	14	0.77
39	Vinci et al., 2011	Italy	Caucasian	HRMA	HB	101/129	44/41/16	65/53/11	LC	9	0.89
40	Kontorovich et al., 2010	Israel	Asian	MassARRAY	PB	167/122	87/40/40	53/30/39	Cancer	9	6.5e-8
41	Liu et al., 2010	USA	Caucasian	PCR-RFLP	HB	1,109/1,130	580/441/88	586/445/99	HNSCC	13	0.29
42	Hu et al., 2009	China	Asian	PCR-RFLP	PB	1,009/1,093	450/460/99	482/503/108	BC	14	0.17
43	Tian et al., 2009	China	Asian	PCR-RFLP	PB	1,058/1,035	123/472/463	112/453/470	LC	14	0.89

*Genotype distributions are sorted as CC/CT/TT*.

a *Genotype distributions are sorted as CC/CT/TT. PB, population-based design; HB, hospital-based design; BC, Breast cancer; CRC, Colorectal cancer; GC, Gastric cancer; HCC, Hepatocellular carcinoma; HNSCC, Head and neck squamous cell carcinoma; LC, Lung cancer; NB, Neuroblastoma; NC, Nasopharyngeal carcinoma; NSCLC, non-Small cell lung carcinoma; OC, Oral cancer; OvC, Ovarian cancer; PC, Prostate cancer; PTC, Papillary thyroid carcinoma; RCC, Renal cell carcinoma; KS, Kaposi sarcoma*.

A total of 110 articles which included 111 studies (41,673 cases and 49,570 controls) evaluated the association of miR-196a-2 rs11614913 and cancer risk (Table 1). The article by Catucci et al. included two studies on separate populations (Catucci et al., 2010). In a study on head and neck carcinoma the genotype frequencies were not reported in the paper and data were retrieved by contacting the authors (Christensen et al., 2010). The final meta-analysis of mir-196a-2 rs11614913 and cancer risk included 111 studies (including 41,673 patients and 49,570 controls), among which 93 were scored greater than eight in the quality assessment and regarded as high quality studies. In 93 out of 111 studies, the genotype distribution of rs11614913 in the control group was concordant with HWE. Mir-196a-2 rs11614913 were genotype using a range of techniques with the most common being PCR-RFLP (*n* = 53). With regard to the ethnicity, 85 studies were performed in Asians, 20 were performed in Caucasians and the remaining six studies were included either Africans or individuals from different ancestries (mixed ancestry). In four studies the patients were subgrouped into multiple cancer types. Namely, Liu evaluated both ovarian and endometrial cancers (Liu, 2015), Parlayan evaluated gastric, lung, colorectal, prostate, and acute leukemia (Parlayan et al., 2014), Toraih studied both GI and non-GI cancers in a study (Toraih et al., 2016b) and hepatic and renal cancers in another study (Toraih et al., 2016a). For these studies genotype distribution of all patients were used to calculate point estimates in the overall analysis. However, in subgroup analysis, as these studies were assigned to more than one subgroup, the genotype distribution of patients with the relevant cancer category/type was used for pooling data. When studies were subgrouped according to the broad cancer category, there were 49 gastrointestinal tract cancers (GI), nine head and neck squamous cell carcinoma (HNSCC), six gynecologic cancers (GyC), six hematological malignancies (HM), 12 urogenital cancers (UG), and 34 other cancers. When studies subgrouped according to cancer type, there were 21 breast cancer (BC), 18 hepatocellular carcinoma (HCC), 13 gastric cancer (GC), ten colorectal cancer (CRC), nine lung cancer (LC), four bladder cancer (BlC), five prostate cancer (PC), six oral carcinoma (OC), four ovarian cancer (OvC), six esophageal cancer (ESCC), and 22 other cancer types.

Moreover, 43 articles comprising 44 studies (15,954 cases and 19,594 controls) evaluated the association of mir-149 rs2292832 and cancer risk (Table 2), among which 39 studies were evaluated as being high quality (quality score > 8). The genotype distribution of rs2292832 in the control groups of 34 studies were in agreement with HWE. The main genotyping technique was PCR-RFLP (29 studies). Most studies (*n*: 39) were performed in Asian populations and only few studies (*n*: 5) had focused on Caucasians. According to the broad cancer category, there were 22 GI studies, seven HNC studies and 15 studies on other cancer types. When studies were subgrouped by cancer type, there were four studies on BC, nine studies on HCC, eight studies on GC, five studies on CRC, three studies on LC and 15 studies on other types of cancer.

### Quantitative Synthesis

#### Association of mir-196a2 rs11614913 and Cancer Risk

**Figure 2:**
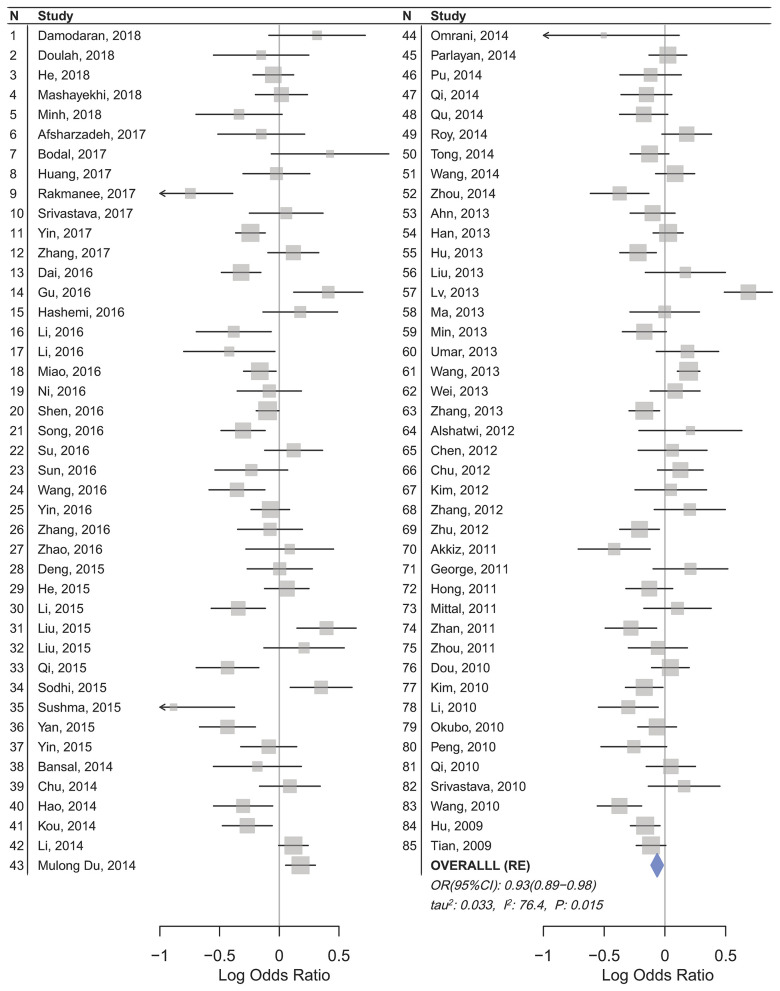
Forest plot of cancer risk associated with mir-196a-2 rs11614913 in the Asian subgroup (Allele contrast T vs. C). The plot is designed in a side-by-side mode to represent 85 studies in the Asian subgroup. The result of meta-analysis is shown beneath the second column.

**Figure 3:**
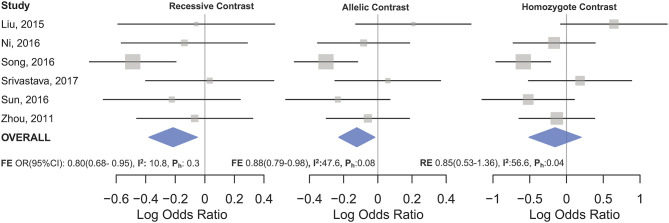
Forest plot of cancer risk associated with mir-196a-2 rs11614913 in the gynecological cancers subgroup. From left to right: recessive (TT vs. CT+CC), allelic (T vs. C), and homozygote (TT vs. CC) contrast.

**Figure 4:**
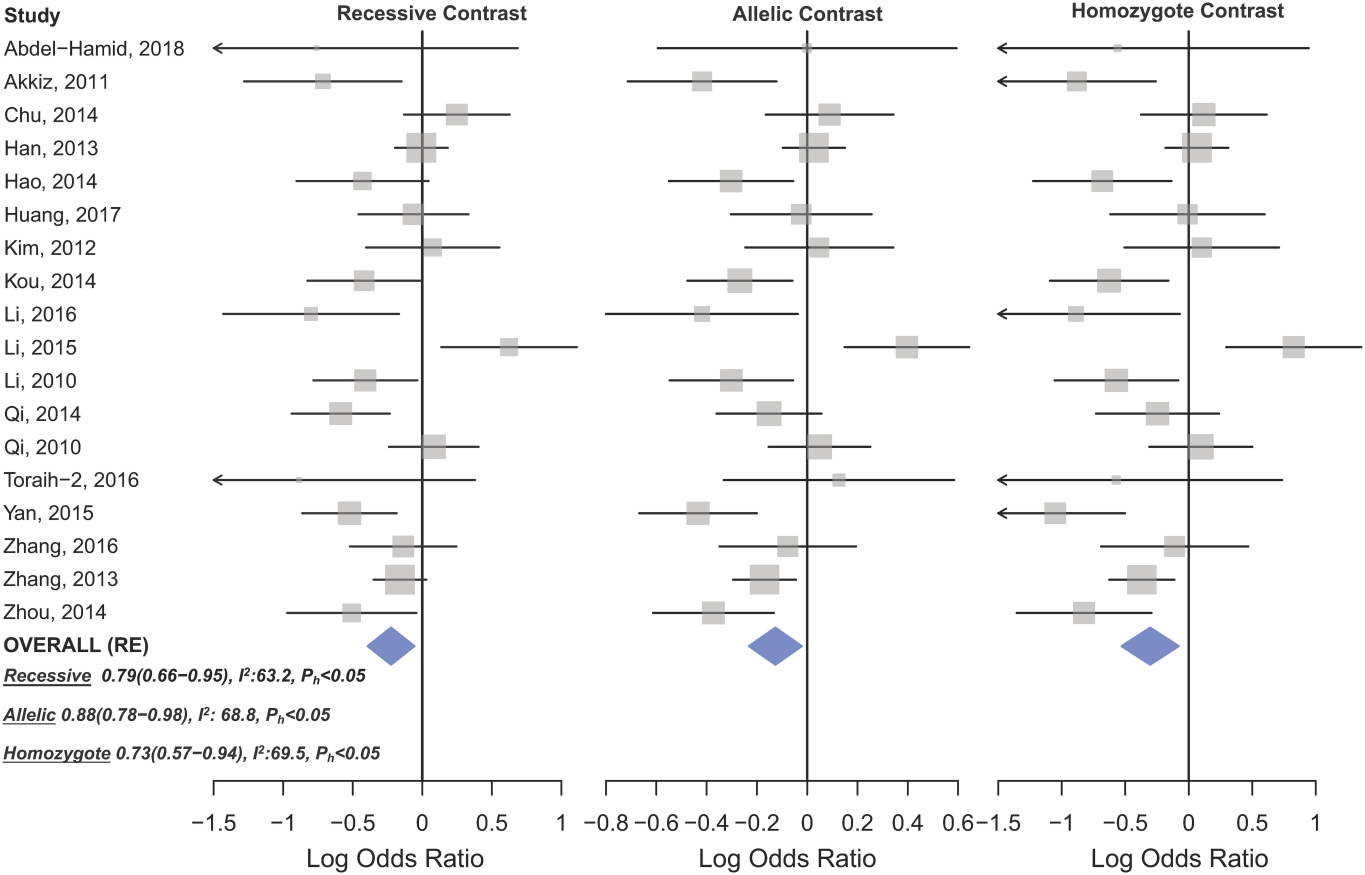
Forest plot of cancer risk associated with mir-196a-2 rs11614913 in the hepatocellular cancer subgroup. From left to right: recessive (TT vs. CT+CC), allelic (T vs. C), and homozygote (TT vs. CC) contrast.

**Figure 5:**
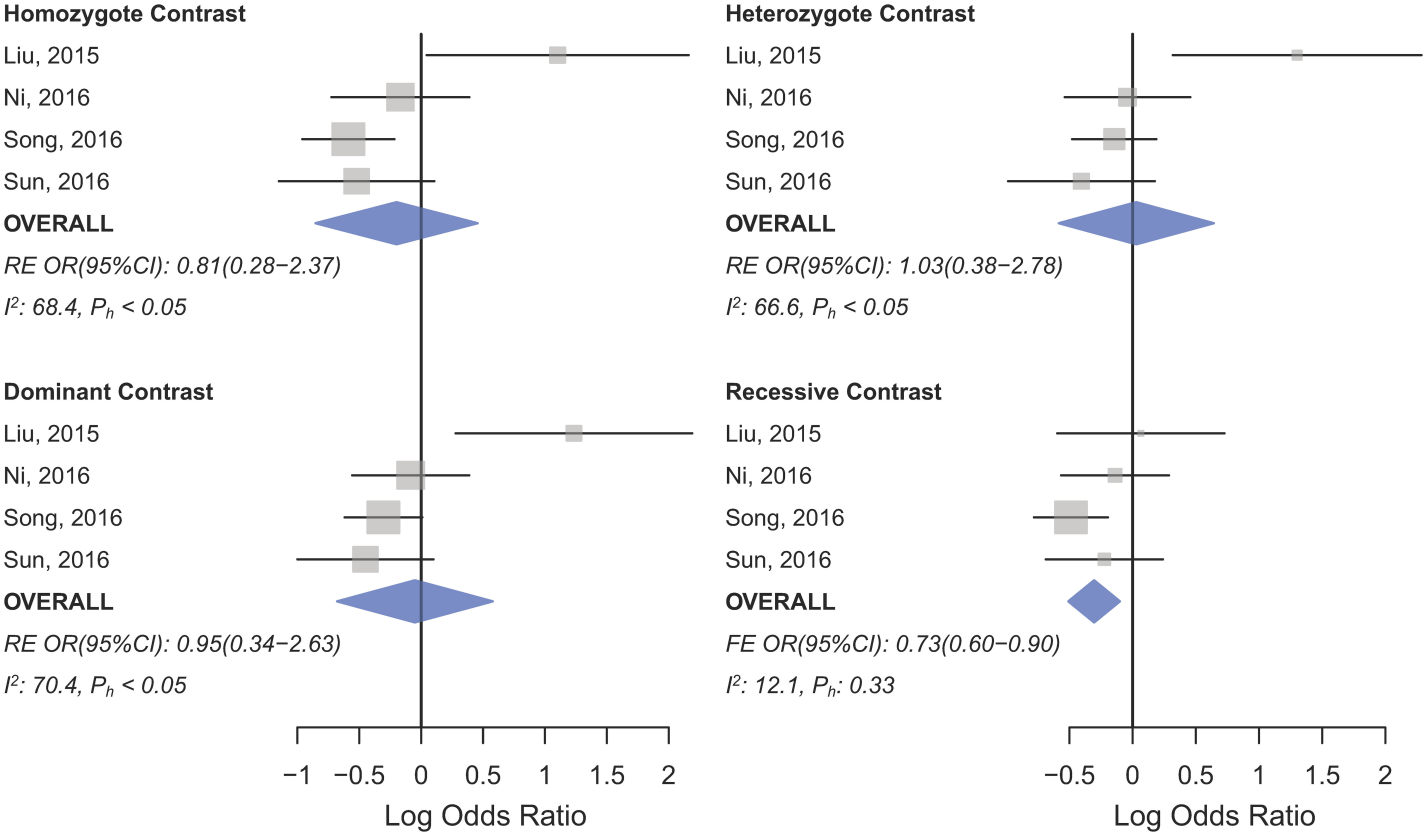
Forest plot of cancer risk associated with mir-196a-2 rs11614913 in the ovarian cancer subgroup. Top left: homozygote (TT vs. CC) contrast; Top right: heterozygote comparison (CT vs. CC); Bottom left: dominant comparison (CT+TT vs. CC); Bottom right: recessive comparison (TT vs. CT+CC).

Statistically significant associations between mir-196a2 rs11614913 and cancer risk were observed assuming the homozygote (TT vs. CC, OR_RE_ [95% CI]: 0.88 [0.79–0.98], *P*: 0.027) and the recessive (TT vs. CC+CT, OR_RE_ [95% CI]: 0.89 [0.83–0.95], *P*: 0.001) models (Table 3). Mir-196a-2 rs11614913 was not associated with cancer risk in the heterozygote and dominant models and there was a non-significant borderline association in the allele contrast (Table 3). Supplementary Figure S1 shows the forest plots of association of mir-196a-2 rs11614913 and cancer risk in five models. The results of subgroup analysis for mir-196a-2 rs11614913 are shown in Table 4. Decreased risk of cancer was found in high quality studies under the homozygote (TT vs. CC, OR_RE_ [95% CI]: 0.87 [0.77–0.97], *P*: 0.017), the recessive (TT vs. CC+CT, OR_RE_ [95% CI]: 0.88 [0.81–0.95], *P*: 0.001) and the allele contrasts (T vs. C, OR_RE_ [95% CI]: 0.93 [0.88–0.99], *P*: 0.020). In subgroup analysis by genotyping method, the only significant association was observed under the recessive model for studies which used a method other than PCR-RFLP (TT vs. CC+CT, OR_RE_ [95% CI]: 0.89 [0.82–0.96], *P*: 0.007). When sub-grouped by ethnicity, decreased risks of cancer under the homozygote (TT vs. CC, OR_RE_ [95% CI]: 0.86 [0.77–0.97], *P*: 0.016), the recessive (TT vs. CC+CT, OR_RE_ [95% CI]: 0.87 [0.81–0.93] *P*: 0.0004) and the allelic (T vs. C, OR_RE_ [95% CI]: 0.93 [0.89–0.98], *P*: 0.015) models were found only in Asians but not in Caucasians or the African/mixed ancestry subgroups (Table 4, Figure 2). Subgrouping by broad cancer categories indicated that mir-196a-2 rs11614913 was associated by a decreased risk of gynecologic cancer (GyC) assuming the recessive model (TT vs. CC+CT, OR_FE_ [95% CI]: 0.80 [0.68–0.95], *P*: 0.010) and the allelic contrast (T vs. C, OR_FE_ [95% CI]: 0.88 [0.79–0.98], *P*: 0.021) (Figure 3). No significant findings were observed for gastrointestinal, head and neck, hematological or urogenital cancers (Table 4). Supplementary Figure S2 presents the forest plots for subgroups according to the broad cancer categories. Further subgrouping by cancer type revealed significant association of mir-196a-2 rs11614913 with hepatocellular carcinoma (Figure 4) under the homozygote model (TT vs. CC, OR_RE_ [95% CI]: 0.73[0.57–0.94], *P*: 0.017), the recessive model (TT vs. CC+CT, OR_RE_ [95% CI]: 0.79 [0.66–0.95], *P*: 0.017) and the allele contrast (T vs. C, OR_FE_ [95% CI]: 0.88 [0.78–0.98], *P*: 0.030), and with ovarian cancer (Figure 5) under the recessive model (TT vs. CC+CT, OR_FE_ [95% CI]: 0.73[0.60–0.90], *P*: 0.003). Supplementary Figure S3 presents the forest plots for subgroups according to cancer type.

**Table 3:** Summary of the results of meta-analysis of cancer risk associated with miR-196a-2 rs11614913 and miR-149 rs2292832.

**Genetic models**	**n^a^**	**Cases/controls**	**OR^b^ (95% CI)**	***P* ^c^**	***P*_Het_^d^**	***I* ^**2**^**	***P*_blas_^e^**
**miR-196a2 rs11614913**							
Homozygote (TT vs. CC)	109	41,342/49,268	0.88 (0.79–0.98)	**0.027**	<0.0001	77.1%	0.370
Heterozygote (CT vs. CC)	111	41,673/49,570	1.00 (0.92–1.07)	0.960	<0.0001	70.4%	0.832
Dominant (TT+CT vs. CC)	111	41,673/49,570	0.96 (0.89–1.04)	0.429	<0.0001	76.8%	0.666
Recessive (TT vs. CT+CC)	109	41,342/49,268	0.89 (0.83–0.95)	**0.001**	<0.0001	68.6%	0.496
Allelic (T vs. C)	111	41,673/49,570	0.95 (0.90–1.00)	0.051	<0.0001	78.9%	0.148
**miR-149 rs2292832**							
Homozygote^f^ (TT vs. CC)	44	15,954/19,594	0.89 (0.77–1.02)	0.119	<0.0001	64.2	0.004
Heterozygote (CT vs. CC)	44	15,954/19,594	0.96 (0.87–1.05)	0.427	0.008	37.1	0.478
Dominant (CT+TT vs. CC)	44	15,954/19,594	1.00 (0.90–1.11)	0.955	<0.0001	53.5	0.175
Recessive^f^ (TT vs. CC+CT)	44	15,954/19,594	1.00 (0.92–1.08)	0.907	<0.0001	48.6	0.045
Allelic (T vs. C)^f^	44	15,954/19,594	0.96 (0.88–1.04)	0.358	<0.0001	73.6	0.026

a *Number of studies in each contrast*.

b *Pooled OR and 95% CI (Random-effect model)*.

c *P-value of the Z-test*.

d *P-value of the Q-test*.

e *P-value of the Begg's test*.

f *For these models (homozygote, recessive, and allelic comparisons of rs2292832) “trim and fill” adjusted results are shown (consult with Supplementary Figure S4 for forest plots and Supplementary Figure S11 for funnel plots of these models)*.

*Significant associations are shown in boldface*.

**Table 4:** Meta-analysis of miR-196a-2 rs11614913 and cancer risk.

		**Homozygote (TT vs. CC)**			**Heterozygote (CT vs. CC)**			**Dominant (TT+CT vs. CC)**			**Recessive (TT vs. CT+CC)**			**Allelic (T vs. C)**		
**Groups**	**Cases/Controls^a^**	**OR^b^** ** (95% CI)**	***P* ^c^**	***I^**2**^***	**OR^b^** ** (95% CI)**	***P* ^c^**	***I^**2**^***	**OR^b^** ** (95% CI)**	***P* ^c^**	***I^**2**^***	**OR^b^** ** (95% CI)**	***P* ^c^**	***I^**2**^***	**OR^b^** ** (95% CI)**	***P* ^c^**	***I^**2**^***
All studies	41,673/49,570	**0.88 (0.79–0.98)**	<1e−4	77.1	1.00 (0.93–1.08)	<1e−4	70.4	0.97 (0.89–1.05)	<1e−4	76.8	**0.89 (0.83–0.96)**	<1e−4	68.6	0.95 (0.90–1.00)	<1e−4	78.9
Controls in HWE ^d^	36,620/42,501	**0.88 (0.80–0.98)**	<1e−4	74.6	1.00 (0.93–1.08)	<1e−4	66.3	0.97 (0.89–1.04)	<1e−4	74.3	**0.88 (0.82–0.95)**	<1e−4	67.7	**0.94 (0.89–0.99)**	<1e−4	77.3
**QUALITY**																
High (>8)	38,503/46,453	**0.87 (0.77–0.97)**	<1e−4	78.1	0.98 (0.91–1.06)	<1e−4	69.6	0.94 (0.87–1.03)	<1e−4	76.7	**0.88 (0.81–0.95)**	<1e−4	70.9	**0.93 (0.88–0.99)**	<1e−4	79.7
Low (≤8)	3170/3117	098 (0.70**–**1.38)	<1e−4	68.3	1.09 (0.80–1.48)	<1e−4	71.9	1.07 (0.80–1.44)	<1e−4	74	0.97 (0.79–1.18)	0.025	44.5	1.03 (0.87–1.22)	<1e−4	71
**GENOTYPING**																
PCR–RFLP	15,282/18,081	0.85 (0.69**–**1.04)	<1e−4	81.4	0.96 (0.83–1.11)	<1e−4	77.8	0.93 (0.80–1.09)	<1e−4	82.9	0.89 (0.78–1.00)	<1e−4	71.1	0.93 (0.85–1.02)	<1e−4	84
Others	26,391/31,489	0.91 (0.82–1.01)	<1e−4	70.3	1.03 (0.96–1.11)	<1e−4	57.2	1.00 (0.92–1.07)	<1e−4	64.8	**0.89 (0.82–0.96)**	<1e−4	66.5	0.96 (0.91–1.01)	<1e−4	70.7
**ETHNICITIES**																
Asian	33,039/38,092	**0.86 (0.77–0.97)**	<1e−4	76.4	1.0 (0.92–1.09)	<1e−4	68.3	0.96 (0.88–1.05)	<1e−4	74.1	**0.87 (0.81–0.93)**	<1e−4	68.2	**0.93 (0.89–0.98)**	<1e−4	76.4
Caucasian	7273/9737	0.86 (0.63–1.18)	<1e−4	81.1	0.92 (0.75–1.13)	<1e−4	78.3	0.91 (0.72–1.15)	<1e−4	85.1	0.92 (0.73–1.15)	<1e−4	71.2	0.94 (0.78–1.13)	0.00	86.5
Africans/mixed	1361/1741	1.40 (0.89–2.21)	0.058	53.1	1.27 (0.87–1.86)	0.01	63.1	1.31 (0.95–1.80)	0.02	60.5	1.24 (0.77–2.0)	0.05	54.3	1.21 (0.99–1.49)	0.047	55.3
**CANCER CATEGORIES**																
GI	16,537/21,226	0.87 (0.71–1.06)	<1e−4	83.7	0.96 (0.84–1.10)	<1e−4	76.8	0.93 (0.80–1.08)	<1e−4	82.9	0.90 (0.79–1.02)	<1e−4	76.1	0.94 (0.85–1.03)	<1e−4	85
HNC	4,865/5,648	1.05 (0.76–1.46)	0.001	68.9	1.10 (0.79–1.54)	<1e−4	75	1.08 (0.78–1.49)	<1e−4	76.2	0.99 (0.90–1.09)	0.085	42.3	1.01 (0.85–1.21)	5e−4	71.2
GyC	1,394/1,573	0.85 (0.53–1.36)	0.042	56.6	1.02 (0.66–1.58)	0.061	52.5	0.96 (0.61–1.50)	0.030	59.5	**0.80 (0.68–0.95)**	0.346	10.8	**0.88 (0.79–0.98)**	0.089	47.6
HM	1,347/2,571	0.71 (0.34–1.49)	1e−4	80.4	0.91 (0.77–1.09)	0.071	50.7	0.79 (0.52–1.19)	0.008	67.5	0.80 (0.42–1.51)	0.00	80.2	0.83 (0.57–1.21)	0.00	80.5
UG	3,599/4,356	0.98 (0.74–1.30)	0.002	61.5	1.19 (0.96–1.48)	0.003	60.5	1.15 (0.94–1.42)	0.002	61.3	0.91 (0.70–1.19)	0.001	63.8	1.047 (0.91–1.19)	0.002	61.5
Others	13,710/16,018	**0.86 (0.76–0.98)**	<1e−4	57.2	0.98 (0.88–1.10)	<1e−4	56.7	0.95 (0.85–1.06)	<1e−4	62.4	**0.87 (0.79–0.95)**	0.003	44.6	0.94 (0.87–1.00)	<1e−4	62.3
**CANCER TYPES**																
BC ^e^	7,401/8,828	0.85 (0.71–1.01)	0.001	56.4	0.93 (0.81–1.08)	0.002	53.3	0.91 (0.78–1.06)	2e−4	60.8	0.89 (0.79–1.01)	0.024	43	0.93 (0.84–1.02)	<1e−4	62.7
HCC^e^	5,401/6,326	**0.73 (0.57–0.94)**	<1e−4	69.5	0.92 (0.78–1.09)	0.002	55.2	0.87 (0.73–1.04)	<1e−4	64.7	**0.79 (0.66–0.95)**	2e−4	63.2	**0.88 (0.78–0.98)**	<1e−4	68.8
GC	4,664/6,385	0.80 (0.47–1.36)	<1e−4	90.3	0.81 (0.56; 1.15)	<1e−4	88.6	0.80 (0.53–1.20)	<1e−4	92.1	0.91 (0.65–1.29)	<1e−4	81.5	0.88 (0.66–1.17)	<1e−4	92.4
CRC^e^	2,567/4,211	1.21 (0.65–2.27)	<1e−4	87.6	1.12 (0.71–1.75)	<1e−4	81.1	1.13 (0.70–1.85)	<1e−4	84.7	1.08 (0.83–1.42)	<1e−4	78.6	1.06 (0.86–1.29)	<1e−4	86.4
LC ^e^	4,453/4,932	0.86 (0.63–1.17)	0.009	60.2	1.01 (0.91–1.12)	0.057	46.9	1.00 (0.82–1.21)	0.015	57.7	0.82 (0.65–1.03)	0.012	59	0.93 (0.81–1.08)	0.004	64.5
BlC	1,390/1,562	0.75 (0.41–1.39)	0.060	59.3	1.01 (0.53–1.89)	0.017	70.4	0.97 (0.62–1.52)	0.088	54	0.77 (0.30–1.97)	3e−4	84.3	0.94 (0.68–1.29)	0.036	64.7
PC	868/1,345	1.01 (0.74–1.39)	0.396	1.8	1.27 (0.83–1.95)	0.057	56.4	1.23 (0.83–1.83)	0.069	54	0.94 (0.71–1.24)	0.485	0	1.06 (0.93–1.22)	0.241	27.1
OC	2,138/2,957	0.98 (0.49–1.95)	<1e−4	81.6	1.14 (0.57–2.29)	<1e−4	82.3	1.07 (0.53–2.12)	<1e−4	85	0.87 (0.76–1.00)	0.189	32.8	0.96 (0.66–1.40)	<1e−4	82
OvC	843/1,100	0.81 (0.28–2.37)	0.023	68.4	1.03 (0.38–2.78)	0.029	66.6	0.95 (0.34–2.63)	0.017	70.4	**0.73 (0.60–0.90)**	0.332	12.1	0.88 (0.58–1.32)	0.050	61.6
ESCC	3,492/4,376	0.85 (0.49–1.47)	<1e−4	82.4	1.04 (0.83–1.31)	0.039	57.2	0.99 (0.76; 1.29)	0.008	67.8	0.81 (0.51–1.28)	<1e−4	81.3	0.94 (0.76–1.16)	6e−4	76.8
Others	8,214/11,444	0.98 (0.80–1.20)	<1e−4	68.5	1.05 (0.91–1.22)	<1e−4	61.5	1.03 (0.88–1.21)	<1e−4	68.3	0.96 (0.83–1.11)	<1e−4	61.7	1.00 (0.89–1.11)	<1e−4	72

*The overall, HWD-sensitivity and subgroup analyses*.

a *n represents number of cases and controls in each group*.

b *Pooled ORs and 95% confidence intervals*.

c *P-value of the heterogeneity test*.

d *Meta-analysis of all studies excluding those with the control group not in HWE*.

e *These subgroups were found to be influenced by departure from HWE. Please consult with the Supplementary Table S2 for details on HWD sensitivity and adjustments*.

*GI, cancers of digestive system; HNC, Head and neck carcinoma; GyC, Gynecologic cancers; HM, Hematological malignancies; UG, Urogenital cancers; BC, breast cancer; HCC, Hepatocellular cancer; GC, gastric cancer; CRC, colorectal cancer; LC, lung cancer; BlC, bladder cancer; PC, prostate cancer; OC, Oral cancer; OvC, ovarian cancer; ESCC, esophageal squamous cell carcinoma*.

*Significant associations are shown in boldface*.

**Figure 6:**
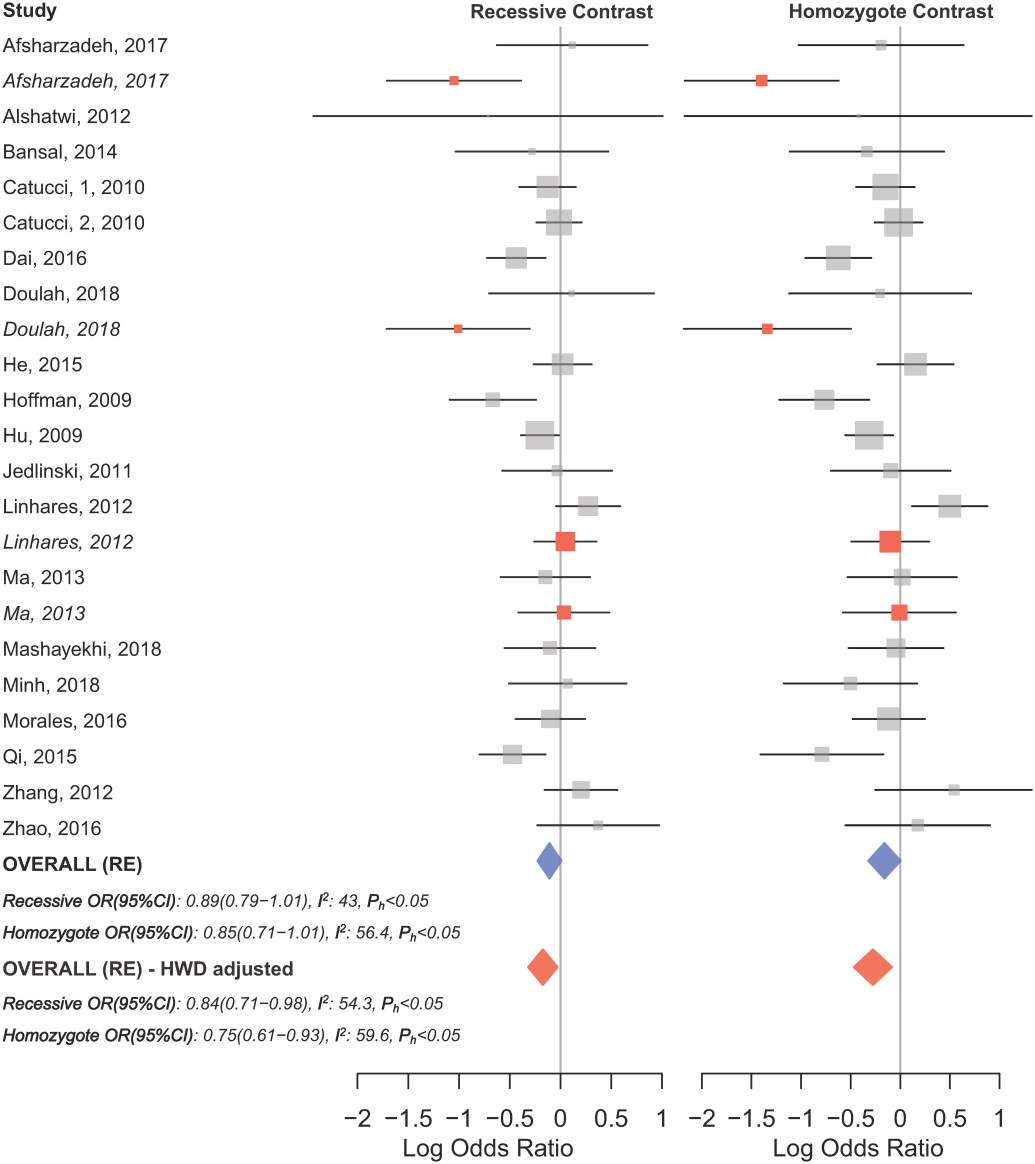
Forest plot of the original and HWD-adjusted meta-analysis of breast cancer risk and mir-196a-2 rs11614913 under the recessive (TT vs. CT+CC) and homozygote (TT vs. CC) models. Red boxes represent HWD-adjusted log ORs and 95%CIs of studies with HWE-deviated controls (i.e., Linhares et al., 2012; Ma et al., 2013; Afsharzadeh et al., 2017; Doulah et al., 2018). The blue and red diamonds represent the estimated pooled effects of the original and HWD-adjusted meta-analysis, respectively.

HWD sensitivity analysis (i.e., excluding studies with controls deviated from HWE) revealed stable results in the overall analysis under the homozygote, heterozygote, dominant, and recessive models (Table 4). However, excluding HWD studies made the borderline allele contrast statistically significant (Table 4). Moreover, excluding HWE violating studies had no dramatic effects on subgroup meta-analyses using quality of studies, genotyping methods, the ethnicity and the broad cancer category (Supplementary Table S1). In meta-analysis subgrouped by cancer type, the results were also stable for gastric, bladder, oral, ovarian, prostate, and esophageal cancer subgroups after excluding HWD studies (Supplementary Table S1). However, excluding such studies altered the results for the breast, hepatocellular, colorectal, and lung cancer subgroups. Therefore, for these subgroups, pooled ORs were estimated to account for departures from HWE (denoted as HWD-adjusted ORs) (Supplementary Table S2). When corrected for HWD, mir-196a-2 rs11614913 was found to be significantly associated with breast cancer under the homozygote (TT vs. CC, HWD-adjusted OR_RE_ [95% CI]: 0.75 [0.61–0.93], *P*: 0.011) and recessive (TT vs. CC+CT, HWD-adjusted OR_RE_ [95% CI]: 0.84 [0.71–0.98], *P*: 0.030) models (Figure 6). The association with hepatocellular cancer under the homozygote and the recessive models was remained significant after adjustment for HWD (TT vs. CC, HWD-adjusted OR_RE_ [95% CI]: 0.69 [0.53–0.91], *P*: 0.011 and TT vs. CC+CT, HWD-adjusted OR_RE_ [95% CI]: 0.72 [0.57–0.90], *P*: 0.008). Furthermore, adjustment for HWD confirmed that mir-196a-2 rs11614913 is not associated with colorectal or lung cancer assuming any genetic model (Supplementary Table S2).

#### Association of mir-149 rs2292832 and Cancer Risk

**Figure 7:**
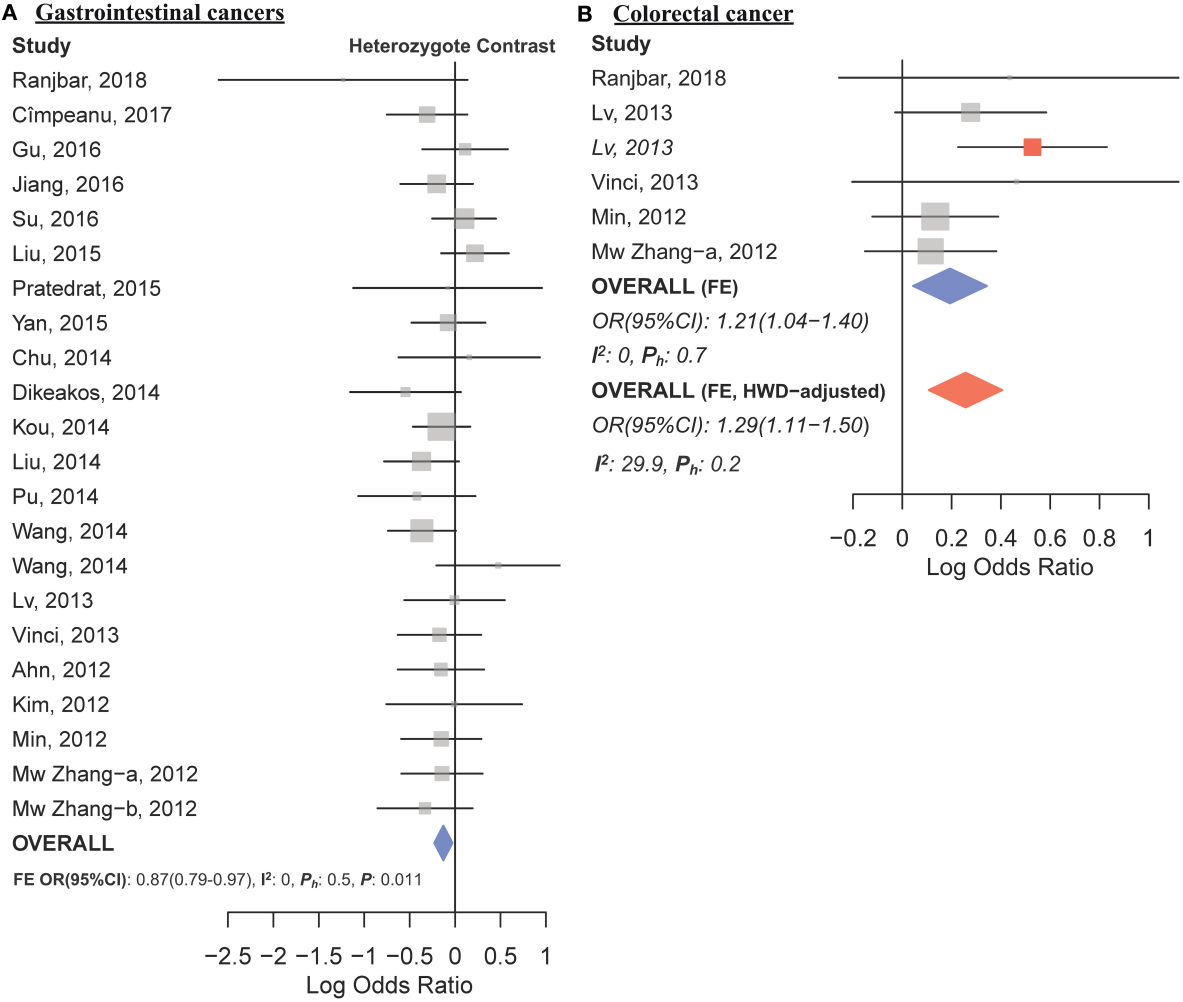
Forest plots of cancer risk associated with mir-149 rs2292832; **(A)** In the gastrointestinal cancers subgroup assuming the heterozygote model (CT vs. CC). **(B)** In the colorectal cancer subgroup assuming the recessive model (TT vs. CT+CC). The red box shows the HWD-adjusted log OR (95%CI) for the study by Lv et al. (2013). The blue and red diamonds represent the estimated pooled effect of the original and HWD-adjusted meta-analysis.

**Figure 8:**
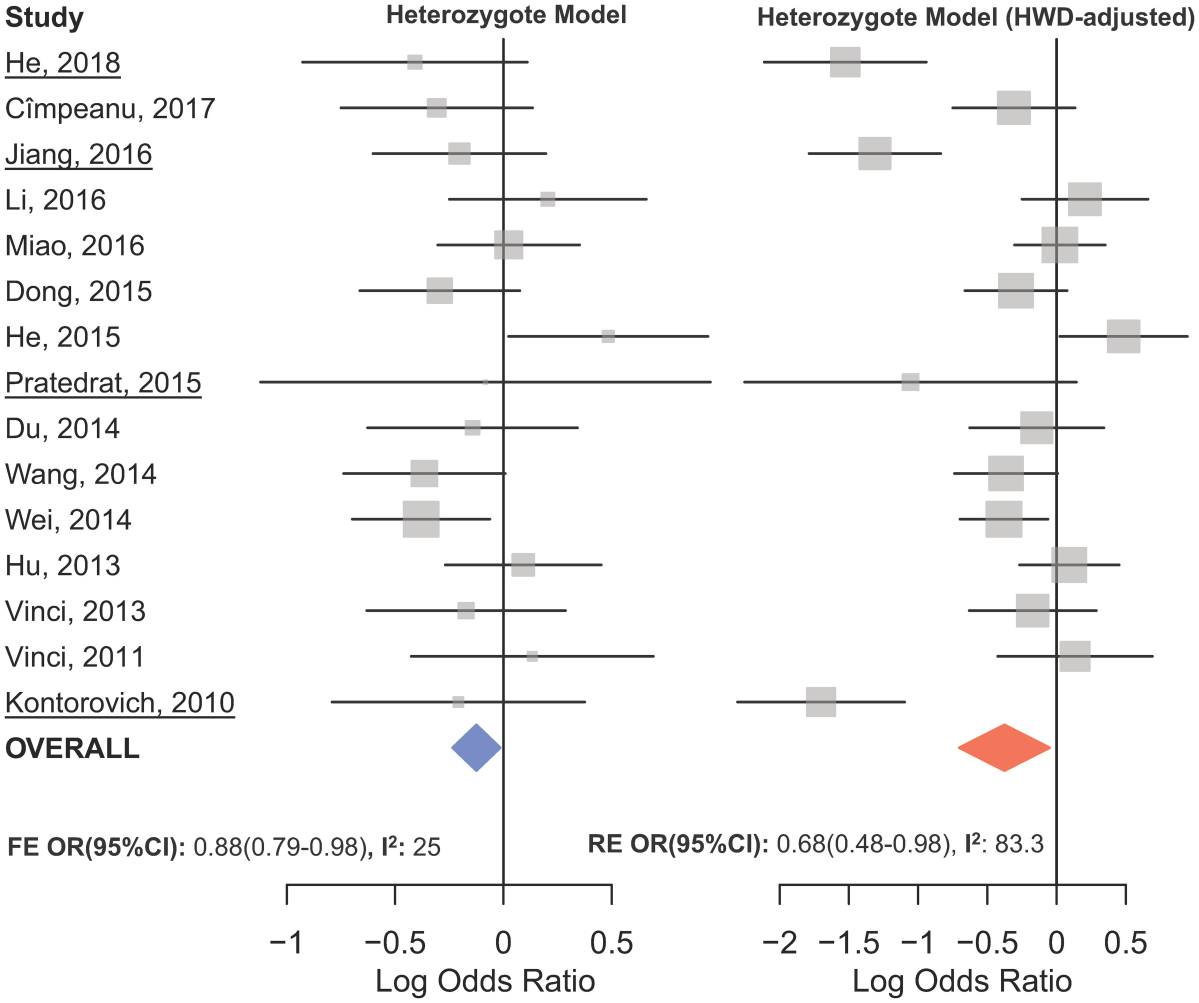
Forest plot of the original **(Left)** and HWD-adjusted **(Right)** meta-analysis of cancer risk associated with mir-149 rs2292832 in subgroup of studies which used a non-RFLP genotyping method assuming the heterozygote model. In the right plot, HWD-adjusted log ORs and 95%CI were used for the underlined studies (i.e., He, 2018; Kontorovich et al., 2010; Pratedrat et al., 2015; Jiang et al., 2016).

The overall analysis showed no significant association with cancer risk under any genetic model (Table 3). Supplementary Figure S4 shows the forest plots for the association of mir-149 rs2292832 and cancer risk under different genetic models. However, in subgroup analyses (Table 5) significant association of rs2292832 with cancer risk was observed in studies which used a genotyping method other than PCR-RFLP (CT vs. CC, OR_FE_ [95% CI]: 0.88 [0.79–0.98], *P*: 0.025). When subgrouped by broad cancer category, a decreased risk of gastrointestinal tract cancers was found in the heterozygote model (Figure 7A, CT vs. CC, OR_FE_ [95% CI]: 0.87 [0.79–0.97], *P*: 0.011). Subgrouping by cancer type, however, revealed an increased risk of colorectal cancer for individuals carrying TT genotype compared to those who carry at least one C allele (Figure 7B, TT vs. CT+CC, OR_FE_ [95% CI]: 1.21 [1.04–1.40], *P*: 0.011). No significant association was observed for other comparisons (Table 5). Supplementary Figures S5, S6 show the forest plots for subgroup analysis according to the broad cancer category and cancer type, respectively. Sensitivity analysis revealed that HWD studies had no significant effect on point estimates in the overall meta-analysis of mir-149 rs2292832 and cancer risk, and still no significant association was observed in overall analysis (Table 5). Moreover, most subgroup analyses were also stable after removing HWD studies (Supplementary Table S3). However, removing studies with HWD controls influenced comparisons in three subgroups: (i) non-PCR-RFLP subgroup (heterozygote model); (ii) the breast cancer subgroup (recessive model); (iii) the colorectal cancer subgroup (recessive model). Therefore, for these subgroups, HWE-expected genotype distributions in controls were used for pooling ORs (denoted as HWD-adjusted OR) (Supplementary Table S4). Adjusting for HWD in these subgroups confirmed the results of original analyses and showed that rs2292832 is associated with cancer risk in non-RFLP subgroup under the heterozygote model (Figure 8, CT vs. CC, HWD-adjusted OR_RE_ [95% CI]: 0.68 [0.48–0.98], *P*: 0.040) and with colorectal cancer risk under the recessive model (TT vs. CT+CC, HWD-adjusted OR_FE_ [95% CI]: 1.29 [1.11–1.50], *P*: 0.0007). No association with breast cancer risk was identified after adjusting for HWD (Supplementary Table S4).

**Table 5:** Meta-analysis of miR-149 rs2292832 and cancer risk.

		**Homozygote (TT vs. CC)**			**Heterozygote (CT vs. CC)**			**Dominant (TT+CT vs. CC)**			**Recessive (TT vs. CT+CC)**			**Allelic (T vs. C)**		
**Groups**	**Cases/Controls^a^**	**OR^b^** ** (95% CI)**	***P* ^c^**	***I^**2**^***	**OR^b^** ** (95% CI)**	***P* ^c^**	***I^**2**^***	**OR^b^** ** (95% CI)**	***P* ^c^**	***I^**2**^***	**OR^b^** ** (95% CI)**	***P* ^c^**	***I^**2**^***	**OR^b^** ** (95% CI)**	***P* ^c^**	***I^**2**^***
All studies	15,954/19,594	0.89 (0.77–1.02)	<1e−4	64.2	0.96 (0.87–1.05)	0.008	37.1	1.00 (0.90– 1.11)	<1e−4	53.5	1.00 (0.92–1.08)	<1e−4	48.6	0.96 (0.88–1.04)	<1e−4	73.6
Controls in HWE^d^	12,873/15,569	0.88 (0.75–1.04)	<1e−4	67	0.95 (0.89–1.02)	0.054	29.7	0.98 (0.87–1.09)	4e−4	50.8	0.98 (0.89–1.07)	<1e−4	51.3	095 (0.87–1.03)	<1e−4	70.8
**QUALITY**																
High (>8)	15,149/18,939	0.98 (0.87–1.11)	<1e−4	52.8	0.94 (0.88–1.00)	0.060	27.4	0.97 (0.88–1.08)	1e−4	51.3	1.04 (0.96–1.13)	7e−4	47.3	1.02 (0.95–1.10)	<1e−4	66.3
Low (≤8)	805/655	1.32 (1.83–1.34)	0.183	35.7	1.25 (0.55–2.87)	0.043	59.3	1.31 (0.69– 2.48)	0.087	50.7	1.15 (0.90–1.46)	0.84	0	1.19 (0.98–1.4)	0.599	0^f^
**GENOTYPING**																
PCR–RFLP	9,437/11,117	1.05 (0.90–1.22)	0.003	46.6	1.01 (0.88–1.14)	0.016	39.5	1.06 (0.92–1.22)	3e−4	54.5	1.08 (0.98–1.19)	0.028	36.3	1.08 (0.98–1.18)	<1e−4	64.5
Others^e^	6,517/8,477	0.94 (0.76–1.16)	9e−4	61.6	**0.88 (0.79**–**0.98)**	0.178	25	0.911 (0.77–1.06)	0.018	48.3	1.0 (0.88–1.13)	0.011	51.4	0.97 (0.88–1.08)	6e−4	62.8
**ETHNICITIES**																
Asian	14,279/17,389	1.01 (0.89–1.15)	0.0001	51.9	0.97 (0.88–1.08)	0.006	40	0.97 (0.88–1.08)	0.006	40	1.05 (0.98–1.13)	0.005	40.6	1.05 (0.97–1.13)	<1e−4	65.1
Caucasians	1,675/2,205	0.98 (0.48–1.96)	0.022	64.9	0.93 (0.80–1.07)	0.30	16.5	0.94 (0.82–1.08)	0.13	43.5	1.04 (0.62–1.72)	0.052	57.4	0.95 (0.72–1.27)	0.049	57.9
**CANCER CATEGORIES**																
GI	6,508/8,150	0.96 (0.83–1.12)	0.043	36.9	**0.87 (0.79**–**0.97)**	0.57	0	0.92 (0.84–1.01)	0.206	19.2	1.07 (0.99–1.15)	0.079	31.5	1.01 (0.94–1.10)	0.010	46
HNC	2,885/3,769	1.35 (0.76–2.38)	0.003	68.6	1.34 (0.82–2.19)	0.001	72.2	1.45 (0.86–2.42)	<1e−4	80.3	1.10 (0.97–1.24)	0.096	44.2	1.30 (0.87–1.94)	<1e−4	82.8
Others	6,561/7,675	0.96 (0.78–1.19)	0.001	59.5	0.97 (0.88–1.06)	0.126	30.4	0.98 (0.83–1.15)	0.010	51.9	0.99 (0.87–1.13)	0.011	51.5	0.99 (0.89–1.11)	2e−4	65.5
**CANCER TYPES**																
BC^e^	1,871/1,894	1.12 (0.53–2.36)	0.014	71.6	1.02 (0.87–1.19)	0.192	36.7	1.07 (0.59–1.93)	0.031	65.9	1.10 (0.68–1.77)	0.063	58.8	1.05 (0.69–1.60)	0.005	76.4
HCC	2,512/3,107	0.95 (0.68–1.34)	0.008	61	0.91 (0.78–1.06)	0.285	17.7	0.93 (0.72–1.20)	0.039	50.6	0.97 (0.86–1.09)	0.049	48.5	0.99 (0.83–1.17)	0.003	65.6
GC	2,527/3,399	0.95 (0.79–1.14)	0.323	13.6	0.85 (0.72–1.00)	0.493	0	0.92 (0.79–1.07)	0.397	4.2	1.08 (0.96–1.22)	0.319	14.2	1.01 (0.93–1.10)	0.194	29.2
CRC^e^	1,469/1,644	1.10 (0.86–1.40)	0.671	0	0.85 (0.67–1.07)	0.615	0	0.97 (0.78–1.20)	0.704	0	**1.21 (1.04**–**1.40)**	0.748	0	1.10 (0.98–1.23)	0.801	0
LC	1,714/1,559	1.04 (0.8246–1.31)	0.116	53.5	1.03 (0.83–1.29)	0.602	0	1.04 (0.84–1.29)	0.351	4.3	0.98 (0.85–1.13)	0.175	42.4	1.04 (0.70–1.52)	0.157	45.8
Others	5,861/7,991	1.02 (0.77–1.34)	2e−4	65.2	1.071 (0.84–1.36)	5e−4	63.5	1.10 (0.85–1.44)	<1e−4	73.5	1.00 (0.86–1.16)	0.012	50.7	1.07 (0.89–1.29)	<1e−4	77.9

*The overall, HWD-sensitivity and subgroup analyses*.

a *n represents number of cases and controls in each group*.

b *Pooled ORs and 95% confidence intervals*.

c *P-value of the heterogeneity test*.

d *Meta-analysis of all studies excluding those with the control group not in HWE*.

e *These subgroups were found to be influenced by departure from HWE. Please refer to the Supplementary Table S4 for details on HWD sensitivity and adjustment analysis for these subgroups*.

f *Although the point estimate of I^2^ was zero for the allele contrast in the low quality subgroup, the random effect model was used based on the 95%CI of I^2^ (0–69.8%) and small number of studies*.

*GI, Gastrointestinal tract cancers; HNC, Head and neck cancers; BC, Breast cancer; HCC, Hepatocellular cancers; GC, Gastric cancer; CRC, Colorectal cancer; LC, Lung cancer*.

*Significant associations are shown in boldface*.

### Heterogeneity, Meta-regression, and Sensitivity Analysis

Heterogeneity was evaluated for both polymorphisms in all genetic models (Tables 3, 4, 5). Significant between study heterogeneity was observed in the overall estimation under all genetic models for mir-196a-2 rs11614913 and consequently random effect model was used. Univariate meta-regression using cancer type, country, ethnicity, the quality of study (either high or low), genotyping method, source of controls (PB or HB) or HWE was performed to identify potential sources of heterogeneity. For mir-196a-2, meta-regression showed that at least a part of the observed between study heterogeneity in the heterozygote (*R*^2^: 24.23%, *P*: 0.007) and dominant (*R*^2^: 19.86%, *P*: 0.028) models could be attributed to the country moderator. However, there was still significant unaccounted heterogeneity even after correcting for the effect of country moderator (Heterozygote *I*^2^: 63.78 and dominant *I*^2^: 72.05, *P* for test of residual heterogeneity < 0.0001). Moreover, Galbraith plot analysis demonstrated three studies (Lv et al., 2013; Wang et al., 2013; Dikeakos et al., 2014) as the most extreme outliers in all genetic models that account for a considerable portion of the observed heterogeneities (Supplementary Figure S7). Excluding these studies led to a 11.6% reduction of *I*^2^ in the homozygote model (from 77.1 to 65.5%), a 12.8% reduction in the heterozygote model (from 70.6 to 57.8%), a 12.3% reduction in the dominant model (from 76.9 to 64.6%), an 9.1% reduction in the recessive model (from 68.8 to 59.7%) and a 11.3% reduction in the allelic model (from 79 to 67.7%). However, excluding these studies did not alter any genotypic contrast and results were comparable to the overall analyses (data not shown). Sensitivity analysis by omitting one study at a time revealed that no individual study significantly influenced the genotype contrasts (Supplementary Figure S8). In the allele contrast, omitting no single study dramatically influenced pooled OR or its 95%CI. However, given that the original 95%CI was borderline (0.90–1.00), omitting some studies lead the upper limit of 95%CI to fall slightly below one (Supplementary Figure S8-e).

Statistically significant heterogeneity was also observed in the overall analysis of miR-149 rs2292832 and cancer risk and, consequently, RE model was used to estimate pooled OR (Table 3). Subgrouping by study level moderators led to a reduction in heterogeneity in some subgroups (Table 5). However, univariate meta-regression showed no statistically significant source of heterogeneity (All *P* > 0.05). Sensitivity analysis by omitting one study at a time revealed no single influential study (Supplementary Figure S9).

### Publication Bias

Rank correlation test of the mir-196a-2 rs11614913 Begg's funnel plot asymmetry revealed no statistically significant evidence of publication bias in any contrast (Table 3 and Supplementary Figure S10). However, rank correlation test for asymmetry of mir-149 rs2292832 funnel plots showed statistically significant results in the homozygote, recessive and the allelic contrasts (Table 3 and Supplementary Figure S11). Consequently, the “trim and fill” approach (Duval and Tweedie, 2000a,b) employed to correct for funnel plot asymmetry arising from publication bias in these models. The results of overall analysis using original studies or trim and fill method under the three models were comparable (see Supplementary Figure S4 to compare forest plots of the original studies vs. trim-and-fill method and Supplementary Figure S11 for funnel plots). After excluding studies with controls deviating from HWE in sensitivity analysis, rank correlation test was still significant in the mentioned three contrasts.

## Discussion

**Figure 9:**
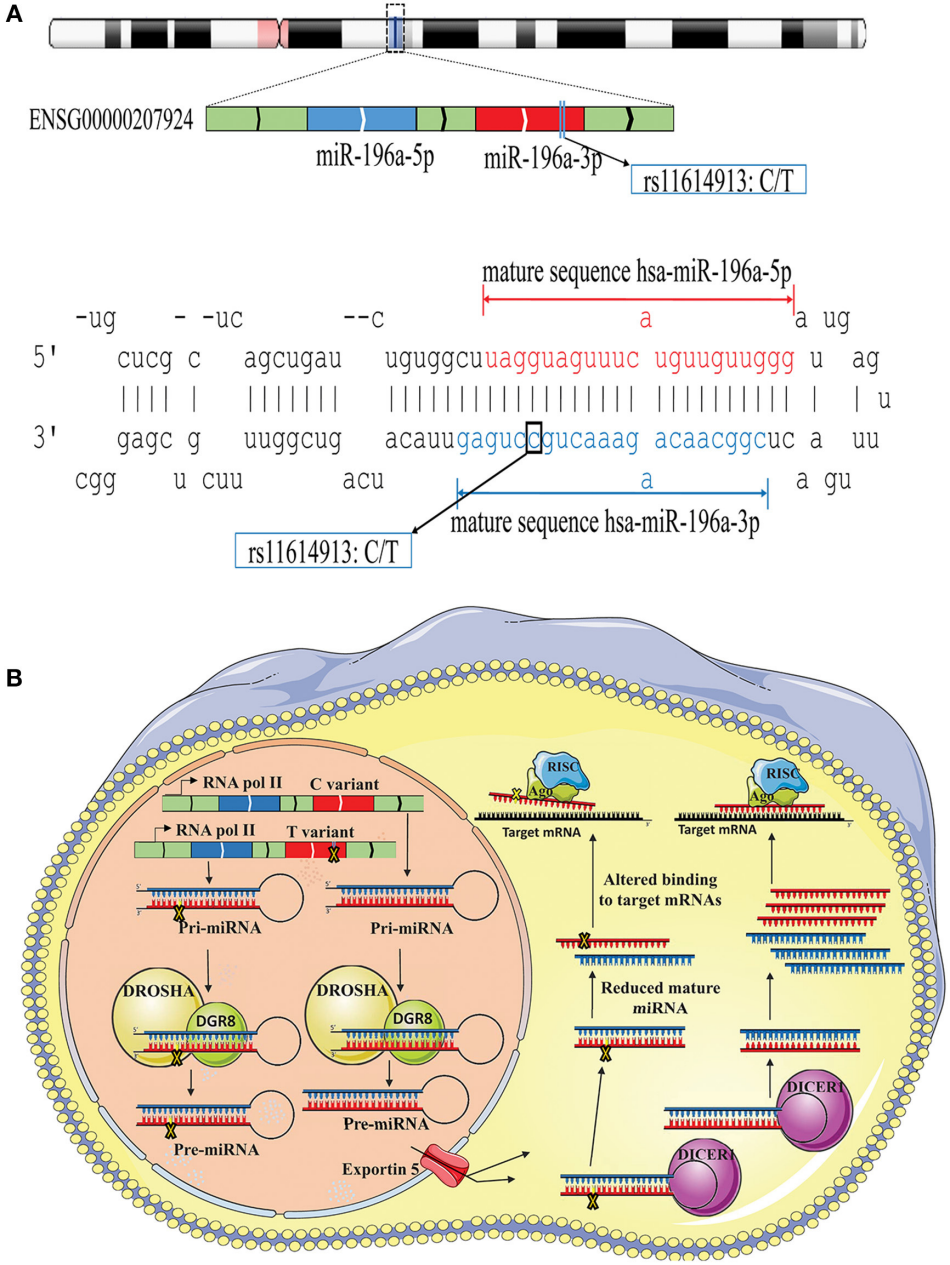
Schematic illustration of hsa-mir-196a-2 locus and effect of rs11614913 on its processing and targeting. **(A)** Top: Hsa-mir-196-a-2 generates two mature miRNAs, miR-196a-5p and mir-196a-3p, and rs11614913 lies in miR-196a-3p at GRCh37 chr12: 54,385,599. Bottom: Stem-loop structure of hsa-mir-196, including mature miR-196-5p (Red) and miR-196-3p (Blue) sequences. **(B)** Rs11614913 alters miRNA processing and/or binding to related target mRNAs.

**Figure 10:**
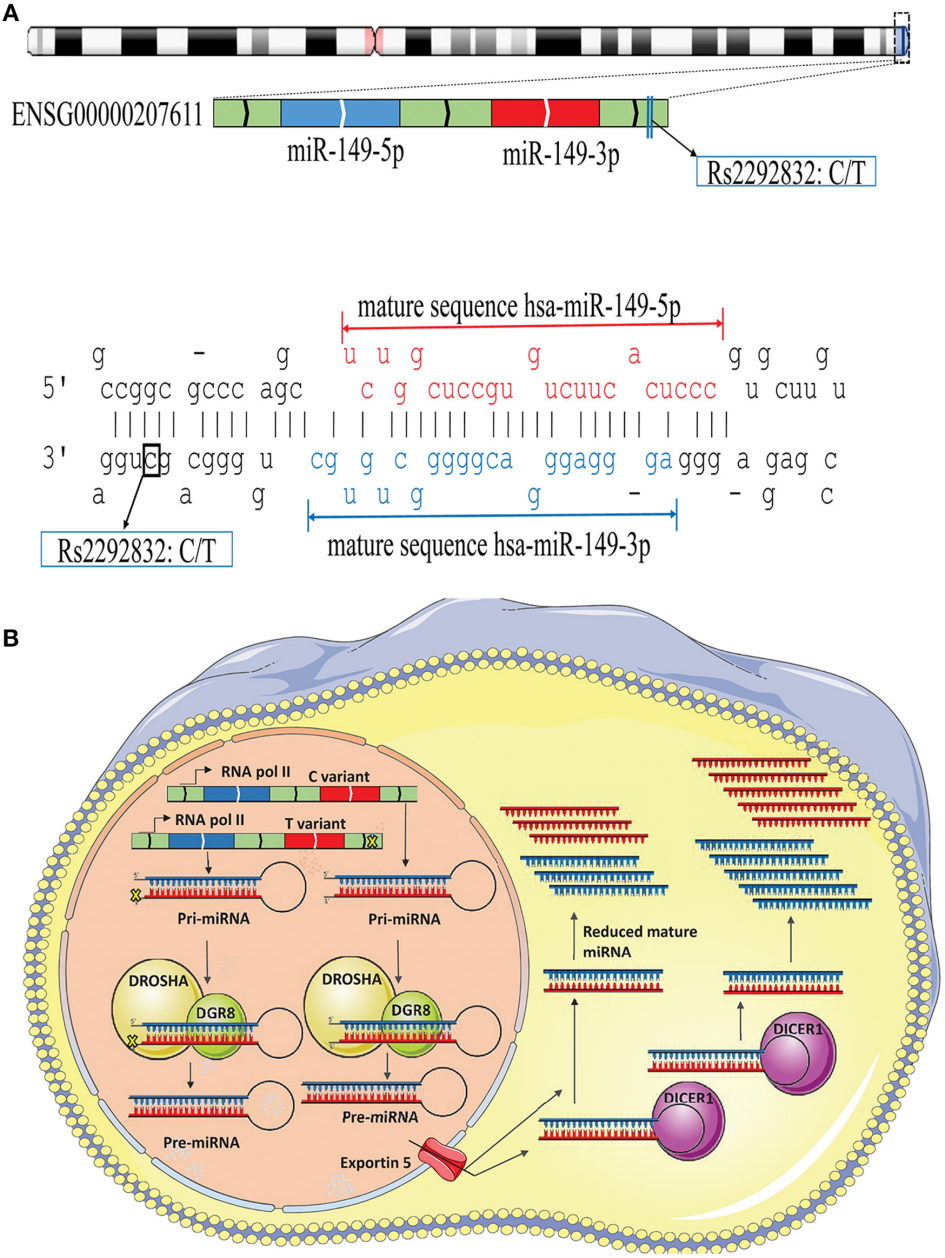
Schematic illustration of hsa-mir-149 locus and effect of rs2292832 on its processing. **(A)** Top: Hsa-mir-149 generates two mature miRNAs (miR-149-5p and miR-149-3p), and rs2292832 resides in pre-miRNA sequence, but not in either mature miRNAs, at GRCh37 chr2: 241,395,593. Bottom: Stem-loop structure of hsa-mir-149, including mature miR-149-5p (Red), and miR-149-3p (Blue) sequences. **(B)** Rs12292832 may alter miRNA processing and decrease mature miR-149 (−5p and −3p forms) expression.

The possible contribution of miRNAs, especially mir-196a-2 and mir-149, to the risk of cancer has stimulated great attention in recent years. Many studies evaluated the functional alterations of these micro-regulators in a wide range of cancers. Accumulating evidence suggests that, at least a part of functional dysregulations of miRNAs in cancers could be attributed to polymorphisms in miRNA sequences (Hu et al., 2008; Hoffman et al., 2009; Tu et al., 2012; Ghaedi et al., 2015; Nariman-Saleh-Fam et al., 2016, 2017). Two mature miRNAs, miR-196a-5p and miR-196a-3p, are generated from the stem-loop structure of hsa-mir-196a-2 (Kozomara and Griffiths-Jones, 2014) with the studied polymorphism, rs11614913, residing in the 3′ arm (Figure 9A). This polymorphism, therefore, may potentially alter miRNA processing and also binding to related target mRNAs (Hoffman et al., 2009) (Figure 9B). Previous studies have shown that the expression level of mature miR-196a-3p was higher in CC carriers with lung cancer compared to CT and TT individuals (Hu et al., 2008). More evidences have been provided by Hoffman et al. (2009) who observed elevated expression of mature mir-196a-2 forms in MCF-7 cells transfected with pre-mir-196a-C vector compared with cells transfected with pre-mir-196a-T vector. The potential of rs11614913 in influencing targeting function of mir-196a-2 has also been documented by whole-genome expression microarrays which found different numbers of dysregulated mRNAs after transfecting cells with pre-mir-196a-C or pre-mir-196a-T vector (Hoffman et al., 2009). Hsa-mir-149 also generates two mature miRNAs (miR-149-5p and miR-149-3p) and the studied polymorphism, rs2292832, does not reside in the mature sequence of neither miR-149-5p or miR-149-3p (Figure 10A). Therefore, it has been hypothesized that rs2292832 is not a structure-shifting polymorphism for pri-mir-149 or pre-mir-149 (Wei et al., 2014). However, Tu et al. reported that the T allele may disrupt the maturation process compared with the C allele and, consequently, decrease miR-149 expression (Tu et al., 2012) (Figure 10B) in head and neck squamous cell carcinoma patients.

Increasing number of association studies evaluating miRNA polymorphisms and cancer risk with contradictory results merits the need for comprehensive systematic reviews and meta-analyses. Several meta-analyses have evaluated the risk of cancer associated with mir-196a-2 rs11614913 or mir-149 rs2292832 (Chu et al., 2011; Zhang H. et al., 2012; Feng et al., 2016; Yan et al., 2017; Liu et al., 2018). However, the conclusion of these studies with regards to the subgroup analysis and the significant genetic model varies due, at least in part, to differences in the number of studies included or in the methodology. Moreover, several recent genetic association studies have not been included in previously published meta-analyses. Therefore, it was necessary to perform an updated meta-analysis with larger number of studies to clarify the association of mir-196a-2 rs11614913 or mir-149 rs2292832 with cancer risk. Therefore, compared to the previous meta-analysis, we included more studies in the analyses. The present meta-analysis also evaluated and corrected for the possible influence of departure of the control group of association studies from HWE. Although checking for departure from HWE has been recommended, currently there is no consensus about how HWD studies should be handled in meta-analysis (Minelli et al., 2008). Result of simulations suggests no advantage for excluding these studies (Minelli et al., 2008). However, sensitivity analysis to detect any possible bias imposed by such studies and/or using HWE-expected counts instead of the observed genotype frequencies have been recommended and implemented in several studies (Attia et al., 2003; Thakkinstian et al., 2005; Trikalinos et al., 2006; Zintzaras et al., 2006; Zintzaras, 2008; Zintzaras and Lau, 2008; Srivastava and Srivastava, 2012; Wang X. B. et al., 2014). The current study noticed that most analyses, especially those with sufficiently large number of studies, were not influenced by excluding HWD studies. However, as it is rationally expected, excluding HWD studies from some subgroup with relatively small number of studies may influence the analysis, and therefore in such situations adjusted analyses were preferred.

For mir-196a-2 rs11614913, the previous largest meta-analysis, conducted by Liu et al. (2018), included 84 studies compared to 111 studies in the present meta-analysis. By including 41,673 patients and 49,570 control subjects, the present meta-analysis showed a decreased risk of cancer in the homozygote and the recessive models (Table 3). Although, the association was not significant in allele contrast, the OR and 95%CI of the allele contrast were borderline and influenced by excluding some individual studies. Excluding HWE-deviated or low quality studies yielded significant associations under allelic model. As there is, currently, no way to adjust allele frequencies for departure from HWE, the possibility that HWD studies may bias the allele contrast cannot be rolled out and a definite conclusion cannot be drawn under allelic model. Apart from the allele contrast, the results of other genetic models were statistically stable and not influenced by removing any single study, HWE deviated or low quality studies. The results also suggest that mir-196a-2 rs11614913 may pose an ethnic dependent effect on cancer risk as associations with cancer were only observed in Asians. However, it should be noted that most studies enrolled Asian patients, mainly Chinese patients, and the number of studies involving other ethnicities were relatively small. Moreover, different minor allele frequencies (MAF) may partly contribute to the observed differences among ethnicities (Average MAF in Asians T: 0.501 ± 0.127, Caucasians T: 0.410 ± 0.1, Others T: 0.338 ± 0.066). In-line with previous studies, the current meta-analysis also confirmed that rs11614913 is associated with decreased risks of hepatocellular cancer under three genetic models (Liu et al., 2018) and that it may not modulate risk of urogenital cancers (Wang et al., 2017). The results of meta-analysis of all studies, subgroup analysis by ethnicity and hepatocellular cancer are in agreement with findings of the previous largest meta-analysis (Liu et al., 2018). However, the increase in the number of analyzed studies led to discrepancies with regards to conclusions in some subgroup analysis. (i) In contrast to the studies by Liu and Pan (Pan et al., 2017; Liu et al., 2018), the present meta-analysis did not find a significant association with head and neck carcinoma in any genetic model. This discrepancy may be attributed to the number of studies included in meta-analyses [nine studies in the present meta-analysis compared to four studies in the meta-analysis by Liu et al. (2018)]. Furthermore, differences in defining head and neck cancer may also explain different conclusions drawn from the present study and the study by Pan and colleagues (Pan et al., 2017). They included esophageal cancer as a type of head and neck cancer, whereas we considered it as a type of gastrointestinal tract cancers (according to the ICD-10-CM C15-C26). (ii) Additionally, the present meta-analysis found significant associations between mir-196a-2 rs11614913 and decreased risks of gynecologic cancers (especially ovarian cancer), which have not been reported in any previous meta-analysis. Interestingly, low heterogeneity was observed in the gynecological cancers subgroup assuming the two significant contrasts (i.e., recessive and allelic). (iii) Although previous meta-analyses (Yan et al., 2017; Zhang H. et al., 2017; Liu et al., 2018) failed to find a significant association between mir-196a-2 rs11614913 and breast cancer, the current study showed, by incorporating more association studies and performing HWD sensitivity analysis, that adjusting for departure from HWE may reveal significant associations under the homozygote and recessive contrasts (Figure 6 and Supplementary Table S2). (iv) Moreover, contradictory to previous meta-analyses, no association with gastric (Yan et al., 2017), colorectal (Xie et al., 2015; Yan et al., 2017) or lung cancer (Ren et al., 2016; Yan et al., 2017; Liu et al., 2018), was found. Apart from larger sample sizes and correcting for HWD, sometimes this discrepancy in results may also be related to methodological differences in the design, specifically the inclusion criteria, of meta-analyses. As a case in point, studies by Hu et al. (2008) and Yoon et al. (2012) did not meet the inclusion criteria of our study, as they deal with the survival or recurrence risk of lung cancer patients with approaches that differed from routine case-control genetic association studies. However, we noticed that these studies were included in a previous meta-analysis (Liu et al., 2018).

The current meta-analysis also showed that mir-149 rs2292832 is not associated with risk of cancer in any genetic model and the results were statistically reliable, as summary effects were not influenced by excluding any single study, HWE-deviated or low quality studies. No differences in cancer risk was observed between ethnicities. Similar to miR-196a-2 polymorphism, the Asian subgroup comprised a large proportion of studies and relatively few studies with limited sample sizes were performed in Caucasians. Therefore, a definite conclusion cannot be drawn in Caucasians and more studies are needed further clarify the association of this SNP with cancer risk in Caucasians. The results of overall analysis were comparable to previous meta-analyses (Li L. et al., 2015; Feng et al., 2016). By pooling the results of 22 studies, this meta-analysis found a decreased risk of gastrointestinal tract (GI) cancers for individuals who carry the CT genotype compared to those with the CC genotype in a heterozygote model. Interestingly, there was no significant heterogeneity in the GI subgroup assuming the heterozygote model indicating the reliability of meta-analysis in this subgroup. Previous meta-analyses yielded different results with regard to GI cancers. A previous meta-analysis of seven studies on GI cancers suggested a marginally elevated risk under the recessive model (TT vs. CT+CC), while another pooling of 10 studies found a borderline decreased risk for the CT vs. TT contrast. Although no significant association was identified in the head and neck cancers subgroup, it should be noted that significant heterogeneity was present in all models except the recessive contrast and number of samples were relatively small. For colorectal cancer the association was in reverse direction and an increased risk was observed in individuals who carry the TT genotype compared with subject who carry at-least one C allele (Table 5). A similar association based on three studies was previously reported (Rong et al., 2017), but not reproduced in other meta-analyses (Li et al., 2013; Feng et al., 2016). Taken together, the current results based on five studies suggest an increased risk for colorectal cancer that was stable after correcting for departure from HWE. Although no significant heterogeneity was detected in the colorectal cancer subgroup under any genetic model, it should be noted that the limited number of studies may influence heterogeneity evaluation and more definite conclusion may be drawn by analyzing larger sample sizes. In the case of breast cancer, a previous meta-analysis of three studies found a significant association (Feng et al., 2016). We found no significant association in the original and HWD-adjusted analysis. However, number of studies in the colorectal and breast cancer subgroups are relatively limited and results should be interpreted with caution. More studies with large sample sizes are needed for a definite conclusion.

However, the present study has some limitations. First, significant heterogeneity was present in most analyses especially for mir-196a-2 polymorphism. We, therefore, used random effect model and performed meta-regression; but no significant source of heterogeneity was observed for most analyses, suggesting that other unknown study level moderators may contribute to the heterogeneity. Second: The molecular mechanisms underlying association of these miRNAs-SNP with risk of cancer are complex and might be strongly affected by different genetic background as well as other masked variables. This, in turn, may limit the efficacy of the overall analysis especially in the case of miR-196a-2 rs11614913. Stratified analyses based on a specific cancer category or a cancer type may help to reduce this heterogeneity and, therefore, are considered to be more reliable. Third, this study was based on unadjusted ORs of the original studies and no adjustment for covariates like age and gender or interaction with environmental factors were done and this fact may also potentially contribute to the between study heterogeneity. Fourth, some limitations such as language restriction or lack of access to the genotype counts of mir-196a-2 rs11614913 in four studies with insufficiently reported data may bring in publication bias. The trim and fill method has been shown to reduce the bias in estimates in the presence of publication bias and heterogeneity (Peters et al., 2007). However, it has been recommended that this method should be considered as sensitivity analysis as we cannot be sure that asymmetry in funnel plot is truly caused by publication bias (Peters et al., 2007). Although rank correlation test of funnel plots of mir-149 rs2292832 was significant in three genetic models raising the possibility of publication bias, adjusting for such a bias using trim-and-fill method did not afford any change in analysis of overall studies in these models (Supplementary Figure S4). Fifth, number of studies in some subgroup analyses was limited and, consequently, results of such analysis should be interpreted with caution. Most studies were performed enrolling Asian patients and the number of studies on Caucasians or Africans was limited. Therefore, more association studies with larger sample sizes on Africans and Caucasians are needed to make precise estimations of cancer risk associated with the studied polymorphisms. Assigning ethnicity to each study population could be another limitation of meta-analysis of association studies as each ethnicity may regroup several sub-populations with somewhat different genetic background. Sixth, the control groups of association studies were not uniformly defined and non-differential misclassification bias may have occurred.

In conclusion, this meta-analysis showed that mir-196a-2 rs11614913 T allele is associated with decreased cancer risk in overall population, high quality studies and studies on Asian populations. It is also associated with a decreased risk of gynecological cancers, ovarian, breast and hepatocellular cancer. Mir-149 rs2292832 was not associated with cancer risk in overall population, high quality studies, Asians or Caucasians. However, the T allele was associated with a decrease risk of gastrointestinal tract cancers under the heterozygote model and an increased risk of colorectal cancer under the recessive model.

## Author Contributions

MB and AM conceived the original idea and supervised the project. JC, ZN-S-F, and ZS contributed to the literature search and data management. MB, JC, and ZN-S-F wrote the manuscript with support from all authors. MB contributed to the data analysis, interpretation of results, and data visualization with inputs from AM. EO and ZS assisted with data visualization. All authors provided critical feedback, discussed the results, and contributed to the final manuscript.

### Conflict of Interest Statement

The authors declare that the research was conducted in the absence of any commercial or financial relationships that could be construed as a potential conflict of interest.

## Abbreviations

BC: breast cancer
BlC: bladder cancer
CRC: colorectal cancer
ESCC: esophageal squamous cell carcinoma
GC: gastric cancer
GI: cancers of digestive system
GyC: Gynecologic cancers
HCC: Hepatocellular cancer
HM: Hematological malignancies
HNC: Head and neck carcinoma
HWE: Hardy-Weinberg equilibrium
HWD: Hardy-Weinberg deviation
LC: lung cancer
UG: Urogenital cancers
OC: Oral cancer
OR: Odds ratio
OvC: ovarian cancer
PC: prostate cancer.
