# Correction: Association of mir-196a-2 rs11614913 and mir-149 rs2292832 polymorphisms with risk of cancer: an updated meta-analysis

**DOI:** 10.3389/fgene.2026.1814124

**Published:** 2026-03-25

**Authors:** Jalal Choupani, Ziba Nariman-Saleh-Fam, Zahra Saadatian, Elaheh Ouladsahebmadarek, Andrea Masotti, Milad Bastami

**Affiliations:** 1 Immunology Research Center, Tabriz University of Medical Sciences, Tabriz, Iran; 2 Women’s Reproductive Health Research Center, Tabriz University of Medical Sciences, Tabriz, Iran; 3 Department of Medical Genetics, Faculty of Medicine, Shahid Beheshti University of Medical Sciences, Tehran, Iran; 4 Research Laboratories, Bambino Gesù Children’s Hospital-IRCCS, Rome, Italy; 5 Drug Applied Research Center, Tabriz University of Medical Sciences, Tabriz, Iran; 6 Department of Medical Genetics, Faculty of Medicine, Tabriz University of Medical Sciences, Tabriz, Iran

**Keywords:** microRNA, polymorphism, meta-analysis, cancer, mir-196a-2, mir-149

The term “Begger’s funnel plot” was incorrectly used. The correct term is “Begg’s funnel plot.”

A correction has been made to the section Results, Publication Bias, Paragraph 1:

“Rank correlation test of the mir-196a-2 rs11614913 Begg’s funnel plot asymmetry revealed no statistically significant evidence of publication bias in any contrast (Table 3 and Supplementary Figure S10). However, rank correlation test for asymmetry of mir-149 rs2292832 funnel plots showed statistically significant results in the homozygote, recessive and the allelic contrasts (Table 3 and Supplementary Figure S11). Consequently, the “trim and fill” approach (Duval and Tweedie, 2000a,b) employed to correct for funnel plot asymmetry arising from publication bias in these models. The results of overall analysis using original studies or trim and fill method under the three models were comparable (see Supplementary Figure S4 to compare forest plots of the original studies vs. trim-and-fill method and Supplementary Figure S11 for funnel plots). After excluding studies with controls deviating from HWE in sensitivity analysis, rank correlation test was still significant in the mentioned three contrasts.”

The original article has been updated.

