# Supplementary material for: Association of mir-196a-2 rs11614913 and mir-149 rs2292832 Polymorphisms With Risk of Cancer: An Updated Meta-Analysis
Source: Front Genet. 2019 Mar 15;10:186. doi: 10.3389/fgene.2019.00186 (PMC6429108; doi:10.3389/fgene.2019.00186)

Supplementary Figure S1. Forest plots for association of miR-196a-2 rs11614913 and cancer risk under different genetic models. **A)** Homozygote comparison; **B)** Heterozygote comparison; **C)** Dominant comparison; **D)** Recessive comparison; **E)** Allele contrast

A)Homozygote Contrast

Studies	OR	95% − CI	Weights
Abdel−Hamid, 2018	0.57	[0.13; 2.57]	0.3%
Afsharzadeh, 2017	0.82	[0.36; 1.89]	0.6%
Ahn, 2013	0.81	[0.55; 1.18]	1.1%
Akkiz, 2011	0.41	[0.22; 0.77]	0.8%
Alshatwi, 2012	0.66	[0.11; 3.79]	0.2%
Bansal, 2014	0.71	[0.33; 1.55]	0.7%
Bodal, 2017			0.0%
Catucci, 1, 2010	0.86	[0.64; 1.16]	1.2%
Catucci, 2, 2010	0.98	[0.77; 1.25]	1.2%
Chen, 2012	1.14	[0.64; 2.02]	0.9%
Christensen, 2010	0.92	[0.63; 1.32]	1.1%
Chu, 2014	1.13	[0.69; 1.85]	1.0%
Chu, 2012	1.57	[1.04; 2.37]	1.1%
Dai, 2016	0.54	[0.38; 0.75]	1.1%
Damodaran, 2018	1.47	[0.65; 3.30]	0.6%
Deng, 2015	0.93	[0.55; 1.60]	0.9%
Dikaiakos, 2015	1.02	[0.54; 1.94]	0.8%
Dikeakos, 2014	0.07	[0.04; 0.12]	0.8%
Dou, 2010	1.17	[0.85; 1.61]	1.2%
Doulah, 2018	0.82	[0.33; 2.04]	0.6%
George, 2011	0.58	[0.15; 2.19]	0.3%
Gu, 2016	2.40	[1.31; 4.40]	0.8%
Han, 2013	1.07	[0.83; 1.37]	1.2%
Hao, 2014	0.51	[0.29; 0.87]	0.9%
Hashemi, 2016	1.70	[0.76; 3.83]	0.6%
He, 2018	0.91	[0.65; 1.27]	1.1%
He, 2015	1.17	[0.80; 1.71]	1.1%
Hezova, 2012	1.25	[0.66; 2.38]	0.8%
Hoffman, 2009	0.47	[0.30; 0.73]	1.0%
Hong, 2011	0.80	[0.54; 1.18]	1.1%
Horikawa, 2008	0.73	[0.46; 1.18]	1.0%
Hu, 2013	0.64	[0.48; 0.87]	1.2%
Hu, 2009	0.73	[0.57; 0.93]	1.2%
Huang, 2017	0.99	[0.54; 1.82]	0.8%
Jedlinski, 2011	0.91	[0.50; 1.66]	0.8%
Kim, 2012	1.11	[0.60; 2.04]	0.8%
Kou, 2014	0.53	[0.34; 0.85]	1.0%
Kupcinskas, 2014	0.84	[0.51; 1.38]	1.0%
Kupcinskas, 2014	1.26	[0.74; 2.14]	0.9%
Li, 2016	0.37	[0.17; 0.81]	0.7%
Li, 2016	0.41	[0.18; 0.93]	0.6%
Li, 2015	0.53	[0.33; 0.84]	1.0%
Li, 2015	2.29	[1.34; 3.89]	0.9%
Li, 2014	1.24	[0.97; 1.60]	1.2%
Li, 2010	0.57	[0.35; 0.92]	1.0%
Linhares, 2012	1.65	[1.13; 2.41]	1.1%
Liu, 2015	1.91	[0.92; 3.93]	0.7%
Liu, 2013	1.41	[0.76; 2.59]	0.8%
Liu, 2010	1.05	[0.82; 1.34]	1.2%
Lv, 2013	13.65	[6.76; 27.60]	0.7%
Ma, 2013	1.02	[0.59; 1.77]	0.9%
Martin−Guerrero, 2015	2.32	[1.28; 4.18]	0.9%
Mashayekhi, 2018	0.96	[0.59; 1.54]	1.0%
Miao, 2016	0.72	[0.55; 0.95]	1.2%
Min, 2013	0.70	[0.49; 1.01]	1.1%
Minh, 2018	0.61	[0.31; 1.19]	0.8%
Mittal, 2011	0.51	[0.18; 1.48]	0.5%
Morales, 2016	0.89	[0.62; 1.28]	1.1%
Mulong Du, 2014	1.52	[1.17; 1.97]	1.2%
Ni, 2016	0.85	[0.48; 1.48]	0.9%
Nikolic, 2015	0.79	[0.48; 1.29]	1.0%
Okubo, 2010	0.88	[0.63; 1.22]	1.1%
Omrani, 2014			0.0%
Parlayan, 2014	1.04	[0.76; 1.42]	1.2%
Pavlakis, 2013	1.12	[0.47; 2.66]	0.6%
Peckham−Gregory, 2016	0.68	[0.38; 1.20]	0.9%
Peng, 2010	0.63	[0.37; 1.08]	0.9%
Poltronieri−Oliveira, 2017	2.19	[1.15; 4.17]	0.8%
Pu, 2014	0.75	[0.42; 1.34]	0.9%
Qi, 2015	0.45	[0.24; 0.84]	0.8%
Qi, 2014	0.78	[0.48; 1.27]	1.0%
Qi, 2010	1.10	[0.73; 1.65]	1.1%
Qu, 2014	0.62	[0.40; 0.95]	1.0%
Rakmanee, 2017	0.25	[0.12; 0.52]	0.7%
Rogoveanu, 2017	0.92	[0.48; 1.73]	0.8%
Roy, 2014	1.34	[0.84; 2.14]	1.0%
Shen, 2016	0.80	[0.66; 0.98]	1.3%
Sodhi, 2015	3.43	[1.42; 8.27]	0.6%
Song, 2016	0.56	[0.38; 0.81]	1.1%
Srivastava, 2017	1.20	[0.60; 2.42]	0.7%
Srivastava, 2010	0.96	[0.47; 1.96]	0.7%
Su, 2016	1.28	[0.75; 2.20]	0.9%
Sun, 2016	0.59	[0.32; 1.11]	0.8%
Sushma, 2015	0.23	[0.09; 0.60]	0.5%
Tian, 2009	0.79	[0.62; 1.01]	1.2%
Tong, 2014	0.84	[0.61; 1.17]	1.1%
Toraih, 2016	2.10	[0.95; 4.60]	0.7%
Toraih, 2016	1.37	[0.62; 3.03]	0.7%
Umar, 2013	1.61	[0.82; 3.18]	0.8%
Vinci, 2013	1.46	[0.60; 3.53]	0.6%
Vinci, 2011	1.99	[0.78; 5.08]	0.5%
Wang, 2016	0.49	[0.31; 0.79]	1.0%
Wang, 2014	1.19	[0.86; 1.65]	1.1%
Wang, 2013	1.50	[1.24; 1.80]	1.3%
Wang, 2010	0.37	[0.25; 0.57]	1.1%
Wei, 2013	1.26	[0.83; 1.90]	1.0%
Yan, 2015	0.35	[0.20; 0.61]	0.9%
Yang, 2008	1.02	[0.75; 1.37]	1.2%
Yin, 2017	0.60	[0.47; 0.78]	1.2%
Yin, 2016	0.87	[0.63; 1.20]	1.1%
Yin, 2015	0.87	[0.54; 1.41]	1.0%
Zhan, 2011	0.57	[0.37; 0.88]	1.0%
Zhang, 2017	1.28	[0.84; 1.94]	1.0%
Zhang, 2016	0.90	[0.50; 1.60]	0.9%
Zhang, 2013	0.69	[0.53; 0.89]	1.2%
Zhang, 2012	1.72	[0.78; 3.80]	0.7%
Zhao, 2016	1.19	[0.58; 2.47]	0.7%
Zhou, 2014	0.44	[0.26; 0.75]	0.9%
Zhou, 2011	0.88	[0.52; 1.47]	0.9%
Zhu, 2012	0.65	[0.47; 0.91]	1.1%
Kim, 2010	0.73	[0.54; 0.98]	1.2%
**Random effects model**	**0.89**	**[0.80; 0.99]**	**100.0%**

Heterogeneity: $I^2 = 77\%$, $\tau^2 = 0.1541$, $p < 0.01$

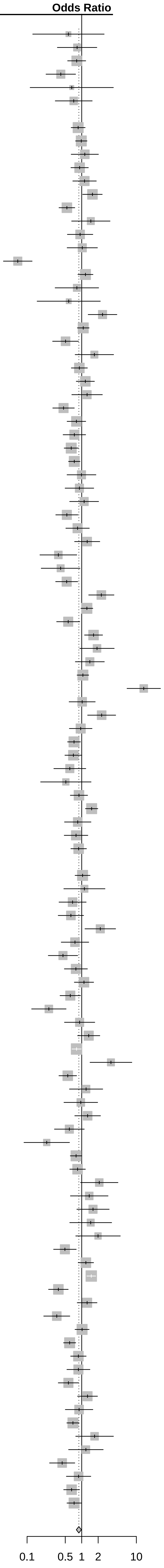

B) Heterozygote Contrast

Studies	OR	95% − CI	Weights
Abdel−Hamid, 2018	1.49	[0.65; 3.39]	0.4%
Afsharzadeh, 2017	0.62	[0.35; 1.11]	0.7%
Ahn, 2013	0.91	[0.65; 1.27]	1.0%
Akkiz, 2011	0.74	[0.47; 1.17]	0.8%
Alshatwi, 2012	1.66	[0.93; 2.94]	0.7%
Bansal, 2014	0.87	[0.52; 1.45]	0.7%
Bodal, 2017	1.79	[1.01; 3.18]	0.7%
Catucci, 1, 2010	0.96	[0.79; 1.16]	1.3%
Catucci, 2, 2010	0.99	[0.84; 1.18]	1.3%
Chen, 2012	1.08	[0.65; 1.80]	0.7%
Christensen, 2010	0.83	[0.63; 1.09]	1.1%
Chu, 2014	0.83	[0.52; 1.32]	0.8%
Chu, 2012	2.05	[1.40; 3.00]	1.0%
Dai, 2016	0.73	[0.56; 0.96]	1.1%
Damodaran, 2018	2.08	[1.12; 3.87]	0.6%
Deng, 2015	0.54	[0.33; 0.89]	0.8%
Dikaiakos, 2015	0.80	[0.43; 1.51]	0.6%
Dikeakos, 2014	0.16	[0.10; 0.24]	0.9%
Dou, 2010	1.45	[1.08; 1.94]	1.1%
Doulah, 2018	0.62	[0.33; 1.18]	0.6%
George, 2011	1.71	[1.12; 2.60]	0.9%
Gu, 2016	1.43	[0.87; 2.35]	0.8%
Han, 2013	1.11	[0.88; 1.39]	1.2%
Hao, 2014	0.69	[0.46; 1.02]	0.9%
Hashemi, 2016	1.14	[0.73; 1.77]	0.9%
He, 2018	0.94	[0.69; 1.27]	1.1%
He, 2015	1.20	[0.85; 1.70]	1.0%
Hezova, 2012	0.92	[0.61; 1.39]	0.9%
Hoffman, 2009	0.84	[0.63; 1.11]	1.1%
Hong, 2011	1.26	[0.89; 1.79]	1.0%
Horikawa, 2008	1.04	[0.71; 1.50]	1.0%
Hu, 2013	0.68	[0.52; 0.90]	1.1%
Hu, 2009	0.85	[0.68; 1.06]	1.2%
Huang, 2017	1.07	[0.59; 1.93]	0.6%
Jedlinski, 2011	0.89	[0.56; 1.42]	0.8%
Kim, 2012	1.04	[0.61; 1.76]	0.7%
Kou, 2014	0.73	[0.52; 1.03]	1.0%
Kupcinskas, 2014	1.40	[1.02; 1.92]	1.1%
Kupcinskas, 2014	1.26	[0.87; 1.82]	1.0%
Li, 2016	0.48	[0.22; 1.05]	0.5%
Li, 2016	0.89	[0.44; 1.80]	0.5%
Li, 2015	0.75	[0.47; 1.19]	0.8%
Li, 2015	1.43	[0.99; 2.08]	1.0%
Li, 2014	0.98	[0.79; 1.23]	1.2%
Li, 2010	0.79	[0.50; 1.24]	0.8%
Linhares, 2012	1.45	[1.03; 2.04]	1.0%
Liu, 2015	2.50	[1.30; 4.80]	0.6%
Liu, 2013	1.66	[0.93; 2.97]	0.7%
Liu, 2010	1.13	[0.94; 1.37]	1.3%
Lv, 2013	7.34	[3.76; 14.34]	0.6%
Ma, 2013	1.30	[0.78; 2.15]	0.8%
Martin−Guerrero, 2015	0.98	[0.59; 1.64]	0.8%
Mashayekhi, 2018	1.12	[0.82; 1.54]	1.1%
Miao, 2016	0.84	[0.66; 1.08]	1.2%
Min, 2013	0.66	[0.48; 0.91]	1.0%
Minh, 2018	0.35	[0.19; 0.65]	0.6%
Mittal, 2011	1.48	[1.01; 2.17]	0.9%
Morales, 2016	0.97	[0.76; 1.24]	1.2%
Mulong Du, 2014	1.46	[1.15; 1.87]	1.2%
Ni, 2016	0.96	[0.58; 1.58]	0.8%
Nikolic, 2015	0.88	[0.64; 1.23]	1.0%
Okubo, 2010	0.95	[0.70; 1.28]	1.1%
Omrani, 2014	0.59	[0.31; 1.11]	0.6%
Parlayan, 2014	0.94	[0.71; 1.25]	1.1%
Pavlakis, 2013	0.66	[0.27; 1.60]	0.4%
Peckham−Gregory, 2016	0.93	[0.65; 1.34]	1.0%
Peng, 2010	0.65	[0.42; 1.01]	0.8%
Poltronieri−Oliveira, 2017	0.78	[0.50; 1.21]	0.8%
Pu, 2014	0.76	[0.49; 1.17]	0.9%
Qi, 2015	0.68	[0.36; 1.29]	0.6%
Qi, 2014	1.54	[1.01; 2.34]	0.9%
Qi, 2010	1.02	[0.71; 1.46]	1.0%
Qu, 2014	1.04	[0.76; 1.41]	1.1%
Rakmanee, 2017	0.56	[0.33; 0.97]	0.7%
Rogoveanu, 2017	0.98	[0.63; 1.50]	0.9%
Roy, 2014	1.24	[0.94; 1.63]	1.1%
Shen, 2016	0.83	[0.69; 0.99]	1.3%
Sodhi, 2015	1.59	[1.09; 2.32]	1.0%
Song, 2016	0.86	[0.62; 1.21]	1.0%
Srivastava, 2017	1.21	[0.61; 2.38]	0.5%
Srivastava, 2010	1.45	[0.98; 2.14]	0.9%
Su, 2016	1.16	[0.81; 1.67]	1.0%
Sun, 2016	0.67	[0.37; 1.19]	0.7%
Sushma, 2015	0.18	[0.05; 0.61]	0.2%
Tian, 2009	0.81	[0.65; 1.02]	1.2%
Tong, 2014	1.26	[0.93; 1.70]	1.1%
Toraih, 2016	1.74	[1.04; 2.92]	0.7%
Toraih, 2016	1.98	[1.19; 3.30]	0.7%
Umar, 2013	1.16	[0.83; 1.62]	1.0%
Vinci, 2013	1.37	[0.88; 2.14]	0.8%
Vinci, 2011	1.47	[0.84; 2.56]	0.7%
Wang, 2016	1.13	[0.75; 1.71]	0.9%
Wang, 2014	1.17	[0.88; 1.55]	1.1%
Wang, 2013	1.37	[1.16; 1.62]	1.3%
Wang, 2010	0.91	[0.68; 1.22]	1.1%
Wei, 2013	1.54	[1.05; 2.26]	0.9%
Yan, 2015	0.52	[0.31; 0.88]	0.7%
Yang, 2008	1.03	[0.82; 1.29]	1.2%
Yin, 2017	0.98	[0.79; 1.22]	1.2%
Yin, 2016	1.04	[0.78; 1.39]	1.1%
Yin, 2015	1.18	[0.77; 1.83]	0.9%
Zhan, 2011	0.80	[0.55; 1.15]	1.0%
Zhang, 2017	1.35	[0.92; 1.98]	0.9%
Zhang, 2016	1.03	[0.59; 1.82]	0.7%
Zhang, 2013	0.75	[0.59; 0.95]	1.2%
Zhang, 2012	1.48	[0.66; 3.33]	0.4%
Zhao, 2016	0.74	[0.39; 1.39]	0.6%
Zhou, 2014	0.62	[0.42; 0.91]	0.9%
Zhou, 2011	0.92	[0.58; 1.44]	0.8%
Zhu, 2012	0.89	[0.66; 1.19]	1.1%
Kim, 2010	0.84	[0.65; 1.10]	1.2%
**Random effects model**	**1.00**	**[0.93; 1.08]**	**100.0%**

Heterogeneity: $I^2 = 70\%$, $\tau^2 = 0.0736$, $p < 0.01$

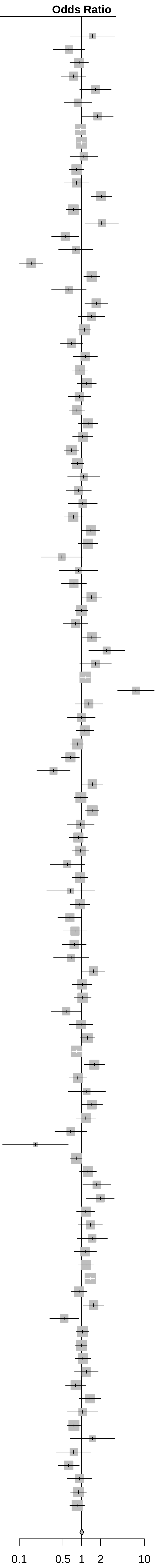

C) **Dominant Model**

Studies	OR	95% − CI	Weights
Abdel−Hamid, 2018	1.27	[0.58; 2.81]	0.5%
Afsharzadeh, 2017	0.66	[0.38; 1.15]	0.7%
Ahn, 2013	0.87	[0.63; 1.20]	1.0%
Akkiz, 2011	0.64	[0.42; 0.98]	0.9%
Alshatwi, 2012	1.58	[0.90; 2.79]	0.7%
Bansal, 2014	0.83	[0.52; 1.33]	0.8%
Bodal, 2017	1.79	[1.01; 3.18]	0.7%
Catucci, 1, 2010	0.93	[0.78; 1.12]	1.2%
Catucci, 2, 2010	0.99	[0.85; 1.16]	1.2%
Chen, 2012	1.10	[0.68; 1.79]	0.8%
Christensen, 2010	0.85	[0.66; 1.10]	1.1%
Chu, 2014	0.94	[0.61; 1.45]	0.8%
Chu, 2012	1.86	[1.30; 2.68]	0.9%
Dai, 2016	0.67	[0.52; 0.86]	1.1%
Damodaran, 2018	1.88	[1.06; 3.35]	0.7%
Deng, 2015	0.67	[0.42; 1.05]	0.8%
Dikaiakos, 2015	0.90	[0.49; 1.64]	0.6%
Dikeakos, 2014	0.12	[0.08; 0.18]	0.9%
Dou, 2010	1.34	[1.01; 1.76]	1.1%
Doulah, 2018	0.66	[0.35; 1.22]	0.6%
George, 2011	1.62	[1.07; 2.45]	0.9%
Gu, 2016	1.67	[1.04; 2.67]	0.8%
Han, 2013	1.09	[0.88; 1.35]	1.2%
Hao, 2014	0.64	[0.43; 0.94]	0.9%
Hashemi, 2016	1.20	[0.78; 1.85]	0.9%
He, 2018	0.93	[0.70; 1.23]	1.1%
He, 2015	1.19	[0.85; 1.65]	1.0%
Hezova, 2012	0.98	[0.66; 1.45]	0.9%
Hoffman, 2009	0.75	[0.57; 0.98]	1.1%
Hong, 2011	1.08	[0.77; 1.50]	1.0%
Horikawa, 2008	0.93	[0.66; 1.32]	1.0%
Hu, 2013	0.67	[0.52; 0.86]	1.1%
Hu, 2009	0.80	[0.65; 0.99]	1.2%
Huang, 2017	1.03	[0.59; 1.81]	0.7%
Jedlinski, 2011	0.90	[0.58; 1.39]	0.8%
Kim, 2012	1.06	[0.64; 1.75]	0.8%
Kou, 2014	0.68	[0.49; 0.95]	1.0%
Kupcinskas, 2014	1.27	[0.94; 1.70]	1.0%
Kupcinskas, 2014	1.26	[0.89; 1.78]	1.0%
Li, 2016	0.42	[0.20; 0.89]	0.5%
Li, 2016	0.70	[0.35; 1.37]	0.6%
Li, 2015	0.64	[0.41; 0.98]	0.9%
Li, 2015	1.60	[1.12; 2.28]	1.0%
Li, 2014	1.07	[0.87; 1.33]	1.2%
Li, 2010	0.69	[0.45; 1.06]	0.9%
Linhares, 2012	1.52	[1.11; 2.08]	1.0%
Liu, 2015	2.28	[1.22; 4.26]	0.6%
Liu, 2013	1.54	[0.91; 2.63]	0.7%
Liu, 2010	1.11	[0.93; 1.33]	1.2%
Lv, 2013	8.70	[4.48; 16.90]	0.6%
Ma, 2013	1.18	[0.74; 1.88]	0.8%
Martin−Guerrero, 2015	1.30	[0.82; 2.06]	0.8%
Mashayekhi, 2018	1.09	[0.80; 1.46]	1.0%
Miao, 2016	0.80	[0.63; 1.01]	1.1%
Min, 2013	0.68	[0.50; 0.91]	1.0%
Minh, 2018	0.44	[0.25; 0.76]	0.7%
Mittal, 2011	1.38	[0.95; 2.01]	0.9%
Morales, 2016	0.95	[0.75; 1.20]	1.1%
Mulong Du, 2014	1.49	[1.18; 1.87]	1.1%
Ni, 2016	0.92	[0.57; 1.47]	0.8%
Nikolic, 2015	0.86	[0.63; 1.18]	1.0%
Okubo, 2010	0.92	[0.69; 1.23]	1.1%
Omrani, 2014	0.59	[0.31; 1.11]	0.6%
Parlayan, 2014	0.97	[0.74; 1.28]	1.1%
Pavlakis, 2013	0.88	[0.38; 1.99]	0.4%
Peckham−Gregory, 2016	0.87	[0.62; 1.24]	1.0%
Peng, 2010	0.64	[0.42; 0.97]	0.9%
Poltronieri−Oliveira, 2017	0.99	[0.66; 1.49]	0.9%
Pu, 2014	0.76	[0.50; 1.16]	0.9%
Qi, 2015	0.53	[0.29; 0.96]	0.6%
Qi, 2014	1.27	[0.84; 1.90]	0.9%
Qi, 2010	1.05	[0.75; 1.47]	1.0%
Qu, 2014	0.92	[0.68; 1.23]	1.0%
Rakmanee, 2017	0.44	[0.26; 0.72]	0.8%
Rogoveanu, 2017	0.96	[0.64; 1.44]	0.9%
Roy, 2014	1.26	[0.97; 1.63]	1.1%
Shen, 2016	0.82	[0.69; 0.97]	1.2%
Sodhi, 2015	1.69	[1.16; 2.45]	0.9%
Song, 2016	0.74	[0.54; 1.01]	1.0%
Srivastava, 2017	1.20	[0.63; 2.31]	0.6%
Srivastava, 2010	1.35	[0.93; 1.95]	0.9%
Su, 2016	1.19	[0.84; 1.68]	1.0%
Sun, 2016	0.64	[0.37; 1.10]	0.7%
Sushma, 2015	0.22	[0.09; 0.57]	0.4%
Tian, 2009	0.81	[0.65; 0.99]	1.2%
Tong, 2014	1.08	[0.81; 1.43]	1.1%
Toraih, 2016	1.82	[1.12; 2.94]	0.8%
Toraih, 2016	1.83	[1.13; 2.97]	0.8%
Umar, 2013	1.21	[0.88; 1.67]	1.0%
Vinci, 2013	1.38	[0.90; 2.13]	0.8%
Vinci, 2011	1.54	[0.90; 2.64]	0.7%
Wang, 2016	0.86	[0.58; 1.26]	0.9%
Wang, 2014	1.18	[0.90; 1.54]	1.1%
Wang, 2013	1.41	[1.21; 1.66]	1.2%
Wang, 2010	0.74	[0.56; 0.98]	1.1%
Wei, 2013	1.43	[1.00; 2.05]	1.0%
Yan, 2015	0.44	[0.27; 0.74]	0.8%
Yang, 2008	1.02	[0.83; 1.27]	1.2%
Yin, 2017	0.84	[0.69; 1.04]	1.2%
Yin, 2016	0.98	[0.74; 1.29]	1.1%
Yin, 2015	1.06	[0.70; 1.61]	0.9%
Zhan, 2011	0.71	[0.50; 1.01]	1.0%
Zhang, 2017	1.32	[0.93; 1.89]	1.0%
Zhang, 2016	0.97	[0.57; 1.65]	0.7%
Zhang, 2013	0.73	[0.58; 0.91]	1.1%
Zhang, 2012	1.62	[0.74; 3.54]	0.5%
Zhao, 2016	0.87	[0.48; 1.58]	0.7%
Zhou, 2014	0.57	[0.39; 0.83]	0.9%
Zhou, 2011	0.90	[0.59; 1.39]	0.9%
Zhu, 2012	0.80	[0.61; 1.06]	1.1%
Kim, 2010	0.80	[0.62; 1.02]	1.1%
**Random effects model**	**0.97**	**[0.89; 1.05]**	**100.0%**

Heterogeneity: $I^2 = 77\%$, $\tau^2 = 0.0914$, $p < 0.01$

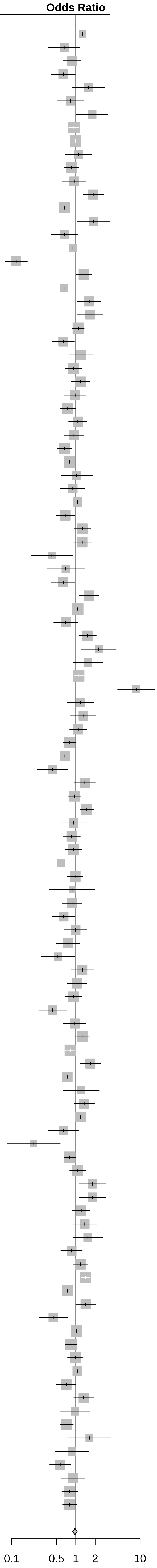

D) Recessive Model

Studies	OR	95% − CI	Weights
Abdel−Hamid, 2018	0.47	[0.11; 1.99]	0.2%
Afsharzadeh, 2017	1.12	[0.53; 2.36]	0.5%
Ahn, 2013	0.87	[0.65; 1.16]	1.2%
Akkiz, 2011	0.49	[0.28; 0.86]	0.7%
Alshatwi, 2012	0.49	[0.09; 2.74]	0.1%
Bansal, 2014	0.75	[0.36; 1.60]	0.5%
Bodal, 2017			0.0%
Catucci, 1, 2010	0.88	[0.67; 1.16]	1.2%
Catucci, 2, 2010	0.99	[0.79; 1.23]	1.3%
Chen, 2012	1.08	[0.69; 1.69]	0.9%
Christensen, 2010	1.02	[0.73; 1.42]	1.1%
Chu, 2014	1.28	[0.88; 1.87]	1.0%
Chu, 2012	0.90	[0.68; 1.20]	1.2%
Dai, 2016	0.65	[0.48; 0.86]	1.2%
Damodaran, 2018	1.00	[0.48; 2.09]	0.5%
Deng, 2015	1.42	[0.93; 2.16]	0.9%
Dikaiakos, 2015	1.22	[0.82; 1.80]	1.0%
Dikeakos, 2014	0.18	[0.10; 0.32]	0.7%
Dou, 2010	0.90	[0.71; 1.14]	1.3%
Doulah, 2018	1.12	[0.50; 2.51]	0.4%
George, 2011	0.42	[0.11; 1.56]	0.2%
Gu, 2016	1.89	[1.14; 3.12]	0.8%
Han, 2013	0.99	[0.82; 1.20]	1.4%
Hao, 2014	0.65	[0.40; 1.05]	0.8%
Hashemi, 2016	1.58	[0.73; 3.42]	0.5%
He, 2018	0.95	[0.72; 1.24]	1.2%
He, 2015	1.02	[0.77; 1.36]	1.2%
Hezova, 2012	1.31	[0.72; 2.40]	0.6%
Hoffman, 2009	0.51	[0.34; 0.79]	0.9%
Hong, 2011	0.68	[0.50; 0.92]	1.1%
Horikawa, 2008	0.72	[0.47; 1.11]	0.9%
Hu, 2013	0.83	[0.66; 1.05]	1.3%
Hu, 2009	0.82	[0.68; 0.98]	1.4%
Huang, 2017	0.94	[0.63; 1.39]	1.0%
Jedlinski, 2011	0.97	[0.56; 1.66]	0.7%
Kim, 2012	1.08	[0.67; 1.74]	0.8%
Kou, 2014	0.66	[0.44; 0.99]	0.9%
Kupcinskas, 2014	0.71	[0.44; 1.12]	0.8%
Kupcinskas, 2014	1.12	[0.68; 1.85]	0.8%
Li, 2016	0.69	[0.45; 1.04]	0.9%
Li, 2016	0.45	[0.24; 0.85]	0.6%
Li, 2015	0.66	[0.48; 0.90]	1.1%
Li, 2015	1.87	[1.15; 3.04]	0.8%
Li, 2014	1.26	[1.04; 1.52]	1.4%
Li, 2010	0.66	[0.46; 0.96]	1.0%
Linhares, 2012	1.31	[0.96; 1.80]	1.1%
Liu, 2015	0.94	[0.56; 1.60]	0.7%
Liu, 2013	1.02	[0.62; 1.67]	0.8%
Liu, 2010	0.97	[0.78; 1.21]	1.3%
Lv, 2013	2.37	[1.72; 3.25]	1.1%
Ma, 2013	0.86	[0.55; 1.34]	0.9%
Martin−Guerrero, 2015	2.34	[1.38; 3.95]	0.7%
Mashayekhi, 2018	0.90	[0.58; 1.41]	0.9%
Miao, 2016	0.81	[0.66; 1.01]	1.3%
Min, 2013	0.93	[0.70; 1.23]	1.2%
Minh, 2018	1.07	[0.60; 1.91]	0.7%
Mittal, 2011	0.41	[0.14; 1.15]	0.3%
Morales, 2016	0.90	[0.64; 1.27]	1.1%
Mulong Du, 2014	1.15	[0.95; 1.38]	1.4%
Ni, 2016	0.87	[0.57; 1.33]	0.9%
Nikolic, 2015	0.84	[0.53; 1.34]	0.8%
Okubo, 2010	0.91	[0.72; 1.16]	1.3%
Omrani, 2014			0.0%
Parlayan, 2014	1.08	[0.85; 1.38]	1.3%
Pavlakis, 2013	1.54	[0.89; 2.65]	0.7%
Peckham−Gregory, 2016	0.71	[0.41; 1.21]	0.7%
Peng, 2010	0.82	[0.52; 1.31]	0.8%
Poltronieri−Oliveira, 2017	2.48	[1.35; 4.55]	0.6%
Pu, 2014	0.92	[0.57; 1.50]	0.8%
Qi, 2015	0.62	[0.45; 0.86]	1.1%
Qi, 2014	0.56	[0.39; 0.79]	1.0%
Qi, 2010	1.09	[0.79; 1.50]	1.1%
Qu, 2014	0.60	[0.41; 0.89]	1.0%
Rakmanee, 2017	0.34	[0.18; 0.66]	0.6%
Rogoveanu, 2017	0.93	[0.51; 1.69]	0.6%
Roy, 2014	1.23	[0.78; 1.92]	0.9%
Shen, 2016	0.92	[0.80; 1.07]	1.4%
Sodhi, 2015	2.54	[1.09; 5.91]	0.4%
Song, 2016	0.61	[0.46; 0.82]	1.2%
Srivastava, 2017	1.03	[0.67; 1.59]	0.9%
Srivastava, 2010	0.83	[0.42; 1.66]	0.5%
Su, 2016	1.17	[0.72; 1.93]	0.8%
Sun, 2016	0.80	[0.50; 1.27]	0.8%
Sushma, 2015	0.55	[0.29; 1.04]	0.6%
Tian, 2009	0.91	[0.75; 1.10]	1.4%
Tong, 2014	0.71	[0.56; 0.91]	1.3%
Toraih, 2016	1.63	[0.77; 3.46]	0.5%
Toraih, 2016	0.99	[0.47; 2.09]	0.5%
Umar, 2013	1.51	[0.78; 2.93]	0.6%
Vinci, 2013	1.23	[0.53; 2.87]	0.4%
Vinci, 2011	1.60	[0.66; 3.88]	0.4%
Wang, 2016	0.45	[0.31; 0.67]	1.0%
Wang, 2014	1.07	[0.83; 1.39]	1.2%
Wang, 2013	1.20	[1.04; 1.39]	1.5%
Wang, 2010	0.40	[0.28; 0.58]	1.0%
Wei, 2013	0.92	[0.67; 1.27]	1.1%
Yan, 2015	0.59	[0.42; 0.83]	1.1%
Yang, 2008	1.00	[0.77; 1.31]	1.2%
Yin, 2017	0.61	[0.49; 0.75]	1.3%
Yin, 2016	0.84	[0.65; 1.09]	1.2%
Yin, 2015	0.77	[0.53; 1.11]	1.0%
Zhan, 2011	0.67	[0.47; 0.94]	1.0%
Zhang, 2017	1.04	[0.75; 1.45]	1.1%
Zhang, 2016	0.87	[0.59; 1.28]	1.0%
Zhang, 2013	0.85	[0.70; 1.03]	1.4%
Zhang, 2012	1.22	[0.86; 1.75]	1.0%
Zhao, 2016	1.45	[0.80; 2.64]	0.6%
Zhou, 2014	0.60	[0.38; 0.96]	0.8%
Zhou, 2011	0.93	[0.63; 1.38]	1.0%
Zhu, 2012	0.71	[0.54; 0.92]	1.2%
Kim, 2010	0.81	[0.63; 1.04]	1.3%
**Random effects model**	**0.89**	**[0.83; 0.96]**	**100.0%**

Heterogeneity: $I^2 = 69\%$, $\tau^2 = 0.0629$, $p < 0.01$

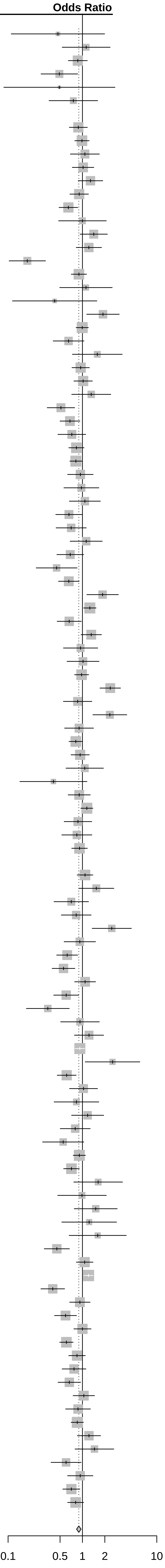

E) **Allele Contrast**

Studies	OR	95% − CI	Weights
Abdel−Hamid, 2018	1.00	[0.55; 1.81]	0.4%
Afsharzadeh, 2017	0.86	[0.60; 1.24]	0.7%
Ahn, 2013	0.90	[0.75; 1.09]	1.0%
Akkiz, 2011	0.66	[0.49; 0.89]	0.8%
Alshatwi, 2012	1.23	[0.81; 1.88]	0.6%
Bansal, 2014	0.83	[0.58; 1.20]	0.7%
Bodal, 2017	1.53	[0.94; 2.50]	0.5%
Catucci, 1, 2010	0.93	[0.82; 1.07]	1.1%
Catucci, 2, 2010	0.99	[0.89; 1.11]	1.2%
Chen, 2012	1.07	[0.80; 1.41]	0.8%
Christensen, 2010	0.93	[0.78; 1.11]	1.0%
Chu, 2014	1.09	[0.85; 1.41]	0.9%
Chu, 2012	1.14	[0.94; 1.37]	1.0%
Dai, 2016	0.73	[0.62; 0.86]	1.1%
Damodaran, 2018	1.37	[0.92; 2.06]	0.6%
Deng, 2015	1.00	[0.76; 1.32]	0.8%
Dikaiakos, 2015	1.09	[0.82; 1.45]	0.8%
Dikeakos, 2014	0.21	[0.15; 0.27]	0.8%
Dou, 2010	1.05	[0.90; 1.22]	1.1%
Doulah, 2018	0.86	[0.58; 1.28]	0.6%
George, 2011	1.23	[0.91; 1.68]	0.8%
Gu, 2016	1.51	[1.13; 2.01]	0.8%
Han, 2013	1.03	[0.91; 1.16]	1.1%
Hao, 2014	0.74	[0.58; 0.95]	0.9%
Hashemi, 2016	1.19	[0.87; 1.63]	0.8%
He, 2018	0.95	[0.80; 1.13]	1.1%
He, 2015	1.06	[0.88; 1.28]	1.0%
Hezova, 2012	1.05	[0.79; 1.40]	0.8%
Hoffman, 2009	0.74	[0.61; 0.90]	1.0%
Hong, 2011	0.88	[0.73; 1.07]	1.0%
Horikawa, 2008	0.87	[0.69; 1.11]	0.9%
Hu, 2013	0.80	[0.69; 0.93]	1.1%
Hu, 2009	0.85	[0.75; 0.96]	1.2%
Huang, 2017	0.98	[0.74; 1.29]	0.8%
Jedlinski, 2011	0.94	[0.70; 1.27]	0.8%
Kim, 2012	1.05	[0.78; 1.41]	0.8%
Kou, 2014	0.77	[0.62; 0.94]	1.0%
Kupcinskas, 2014	1.05	[0.84; 1.31]	1.0%
Kupcinskas, 2014	1.17	[0.91; 1.50]	0.9%
Li, 2016	0.68	[0.50; 0.93]	0.8%
Li, 2016	0.66	[0.45; 0.96]	0.6%
Li, 2015	0.71	[0.57; 0.89]	0.9%
Li, 2015	1.49	[1.16; 1.90]	0.9%
Li, 2014	1.13	[1.00; 1.27]	1.1%
Li, 2010	0.74	[0.58; 0.95]	0.9%
Linhares, 2012	1.32	[1.08; 1.61]	1.0%
Liu, 2015	1.23	[0.88; 1.72]	0.7%
Liu, 2013	1.18	[0.85; 1.64]	0.7%
Liu, 2010	1.04	[0.92; 1.17]	1.2%
Lv, 2013	1.99	[1.63; 2.42]	1.0%
Ma, 2013	1.00	[0.75; 1.33]	0.8%
Martin−Guerrero, 2015	1.50	[1.10; 2.05]	0.8%
Mashayekhi, 2018	1.02	[0.82; 1.27]	1.0%
Miao, 2016	0.85	[0.74; 0.97]	1.1%
Min, 2013	0.84	[0.70; 1.01]	1.0%
Minh, 2018	0.71	[0.50; 1.02]	0.7%
Mittal, 2011	1.11	[0.84; 1.46]	0.8%
Morales, 2016	0.95	[0.80; 1.13]	1.1%
Mulong Du, 2014	1.20	[1.05; 1.35]	1.1%
Ni, 2016	0.92	[0.70; 1.20]	0.9%
Nikolic, 2015	0.89	[0.71; 1.11]	0.9%
Okubo, 2010	0.94	[0.80; 1.10]	1.1%
Omrani, 2014	0.60	[0.32; 1.12]	0.4%
Parlayan, 2014	1.02	[0.88; 1.20]	1.1%
Pavlakis, 2013	1.23	[0.82; 1.85]	0.6%
Peckham−Gregory, 2016	0.86	[0.67; 1.11]	0.9%
Peng, 2010	0.77	[0.59; 1.01]	0.9%
Poltronieri−Oliveira, 2017	1.24	[0.92; 1.68]	0.8%
Pu, 2014	0.89	[0.69; 1.14]	0.9%
Qi, 2015	0.65	[0.50; 0.84]	0.9%
Qi, 2014	0.86	[0.70; 1.06]	1.0%
Qi, 2010	1.05	[0.86; 1.29]	1.0%
Qu, 2014	0.84	[0.69; 1.02]	1.0%
Rakmanee, 2017	0.47	[0.33; 0.68]	0.7%
Rogoveanu, 2017	0.96	[0.71; 1.29]	0.8%
Roy, 2014	1.20	[0.98; 1.47]	1.0%
Shen, 2016	0.91	[0.82; 1.00]	1.2%
Sodhi, 2015	1.42	[1.10; 1.84]	0.9%
Song, 2016	0.74	[0.61; 0.89]	1.0%
Srivastava, 2017	1.06	[0.78; 1.44]	0.8%
Srivastava, 2010	1.17	[0.87; 1.57]	0.8%
Su, 2016	1.13	[0.89; 1.44]	0.9%
Sun, 2016	0.79	[0.58; 1.07]	0.8%
Sushma, 2015	0.41	[0.25; 0.69]	0.5%
Tian, 2009	0.89	[0.79; 1.01]	1.2%
Tong, 2014	0.88	[0.75; 1.03]	1.1%
Toraih, 2016	1.59	[1.10; 2.29]	0.7%
Toraih, 2016	1.40	[0.98; 2.01]	0.7%
Umar, 2013	1.20	[0.93; 1.56]	0.9%
Vinci, 2013	1.24	[0.89; 1.71]	0.7%
Vinci, 2011	1.37	[0.93; 2.02]	0.6%
Wang, 2016	0.70	[0.56; 0.89]	0.9%
Wang, 2014	1.09	[0.93; 1.28]	1.1%
Wang, 2013	1.22	[1.11; 1.33]	1.2%
Wang, 2010	0.69	[0.57; 0.83]	1.0%
Wei, 2013	1.09	[0.89; 1.33]	1.0%
Yan, 2015	0.65	[0.51; 0.82]	0.9%
Yang, 2008	1.01	[0.87; 1.17]	1.1%
Yin, 2017	0.79	[0.69; 0.89]	1.1%
Yin, 2016	0.93	[0.79; 1.09]	1.1%
Yin, 2015	0.92	[0.72; 1.16]	0.9%
Zhan, 2011	0.76	[0.61; 0.93]	1.0%
Zhang, 2017	1.13	[0.91; 1.39]	1.0%
Zhang, 2016	0.93	[0.71; 1.21]	0.9%
Zhang, 2013	0.84	[0.74; 0.96]	1.1%
Zhang, 2012	1.23	[0.92; 1.64]	0.8%
Zhao, 2016	1.09	[0.76; 1.58]	0.7%
Zhou, 2014	0.69	[0.54; 0.88]	0.9%
Zhou, 2011	0.94	[0.74; 1.20]	0.9%
Zhu, 2012	0.81	[0.69; 0.96]	1.1%
Kim, 2010	0.84	[0.72; 0.98]	1.1%
**Random effects model**	**0.95**	**[0.90; 1.00]**	**100.0%**

Heterogeneity: $I^2 = 79\%$, $\tau^2 = 0.0397$, $p < 0.01$

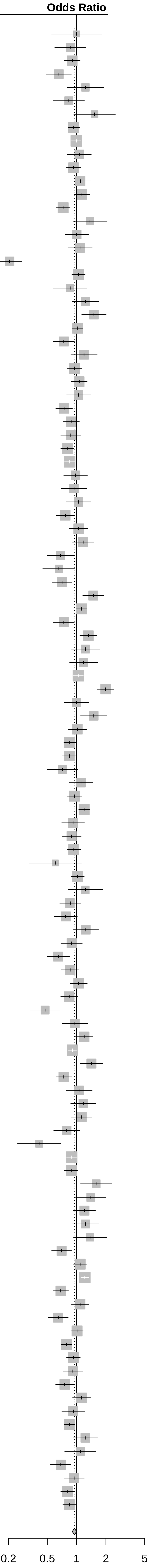

Supplement: Supplementary file 1 [file Data_Sheet_1.ZIP › Supplementary Figures/Suppl. Fig. S1.pdf]
