# Supplementary material for: Association of mir-196a-2 rs11614913 and mir-149 rs2292832 Polymorphisms With Risk of Cancer: An Updated Meta-Analysis
Source: Front Genet. 2019 Mar 15;10:186. doi: 10.3389/fgene.2019.00186 (PMC6429108; doi:10.3389/fgene.2019.00186)

Supplementary Figure S2. Forest plots for subgroup meta-analysis of miR-196a-2 rs11614913 according to the broad cancer category. **A)** Homozygote comparison; **B)** Heterozygote comparison; **C)** Dominant comparison; **D)** Recessive comparison; **E)** Allele contrast. **Abbreviations:** GI: cancers of digestive system; HNC: Head and neck carcinoma; GyC: Gynecologic cancers; HM: Hematological malignancies; UG: Urogenital cancers

A) Homozygote comparison

Study	OR	95%−CI	Weight (random)	Weight (fixed)
**GI**				
Abdel−Hamid, 2018	0.57	[0.13; 2.57]	0.6%	0.2%
Ahn, 2013	0.81	[0.55; 1.18]	2.5%	2.7%
Akkiz, 2011	0.41	[0.22; 0.77]	1.9%	1.4%
Chen, 2012	1.14	[0.64; 2.02]	2.0%	1.0%
Chu, 2014	1.13	[0.69; 1.85]	2.2%	1.4%
Dikaiakos, 2015	1.02	[0.54; 1.94]	1.8%	0.9%
Dikeakos, 2014	0.07	[0.04; 0.12]	1.9%	4.4%
Gu, 2016	2.40	[1.31; 4.40]	1.9%	0.6%
Han, 2013	1.07	[0.83; 1.37]	2.8%	5.6%
Hao, 2014	0.51	[0.29; 0.87]	2.0%	1.7%
Hezova, 2012	1.25	[0.66; 2.38]	1.8%	0.8%
Huang, 2017	0.99	[0.54; 1.82]	1.9%	1.0%
Kim, 2012	1.11	[0.60; 2.04]	1.9%	0.9%
Kou, 2014	0.53	[0.34; 0.85]	2.2%	2.3%
Kupcinskas, 2014	0.84	[0.51; 1.38]	2.2%	1.6%
Kupcinskas, 2014	1.26	[0.74; 2.14]	2.1%	1.1%
Li, 2016	0.37	[0.17; 0.81]	1.5%	1.0%
Li, 2016	0.41	[0.18; 0.93]	1.4%	0.8%
Li, 2015	2.29	[1.34; 3.89]	2.1%	0.8%
Li, 2010	0.57	[0.35; 0.92]	2.2%	2.0%
Lv, 2013	13.65	[6.76; 27.60]	1.7%	0.3%
Min, 2013	0.70	[0.49; 1.01]	2.5%	3.3%
Okubo, 2010	0.88	[0.63; 1.22]	2.6%	3.5%
Parlayan, 2014	0.86	[0.57; 1.30]	2.4%	2.2%
Pavlakis, 2013	1.12	[0.47; 2.66]	1.4%	0.4%
Peng, 2010	0.63	[0.37; 1.08]	2.1%	1.5%
Poltronieri−Oliveira, 2017	2.19	[1.15; 4.17]	1.8%	0.6%
Pu, 2014	0.75	[0.42; 1.34]	2.0%	1.2%
Qi, 2014	0.78	[0.48; 1.27]	2.2%	1.7%
Qi, 2010	1.10	[0.73; 1.65]	2.4%	2.0%
Qu, 2014	0.62	[0.40; 0.95]	2.3%	2.4%
Rogoveanu, 2017	0.92	[0.48; 1.73]	1.8%	0.9%
Shen, 2016	0.80	[0.66; 0.98]	2.9%	10.3%
Srivastava, 2010	0.96	[0.47; 1.96]	1.7%	0.7%
Su, 2016	1.28	[0.75; 2.20]	2.1%	1.1%
Toraih, 2016	2.48	[1.03; 5.93]	1.3%	0.3%
Toraih−2, 2016	0.56	[0.15; 2.09]	0.8%	0.3%
Umar, 2013	1.61	[0.82; 3.18]	1.7%	0.6%
Vinci, 2013	1.46	[0.60; 3.53]	1.3%	0.4%
Wang, 2014	1.19	[0.86; 1.65]	2.6%	3.1%
Wang, 2013	1.50	[1.24; 1.80]	2.9%	8.3%
Wang, 2010	0.37	[0.25; 0.57]	2.4%	3.5%
Wei, 2013	1.26	[0.83; 1.90]	2.4%	1.8%
Yan, 2015	0.35	[0.20; 0.61]	2.0%	2.0%
Zhan, 2011	0.57	[0.37; 0.88]	2.4%	2.5%
Zhang, 2016	0.90	[0.50; 1.60]	2.0%	1.1%
Zhang, 2013	0.69	[0.53; 0.89]	2.8%	6.4%
Zhou, 2014	0.44	[0.26; 0.75]	2.1%	1.9%
Zhu, 2012	0.65	[0.47; 0.91]	2.6%	3.9%
**Fixed effect model**	**0.89**	**[0.84; 0.95]**	**−−**	**100.0%**
**Random effects model**	**0.87**	**[0.71; 1.07]**	**100.0%**	**−−**
Heterogeneity: $I^2 = 84\%$, $\tau^2 = 0.2619$, $p < 0.01$				
Test for effect in subgroup (fixed effect): $z = -3.72$ ($p < 0.01$)				
Test for effect in subgroup (random effects): $t_{48} = -1.38$ ($p = 0.17$)				
**Others**				
Afsharzadeh, 2017	0.82	[0.36; 1.89]	2.1%	0.7%
Alshatwi, 2012	0.66	[0.11; 3.79]	0.7%	0.2%
Bansal, 2014	0.71	[0.33; 1.55]	2.2%	0.9%
Bodal, 2017			0.0%	0.0%
Catucci, 1, 2010	0.86	[0.64; 1.16]	4.0%	5.5%
Catucci, 2, 2010	0.98	[0.77; 1.25]	4.2%	7.7%
Dai, 2016	0.54	[0.38; 0.75]	3.8%	5.5%
Dou, 2010	1.17	[0.85; 1.61]	3.9%	4.0%
Doulah, 2018	0.82	[0.33; 2.04]	1.9%	0.6%
He, 2018	0.91	[0.65; 1.27]	3.8%	4.0%
He, 2015	1.17	[0.80; 1.71]	3.7%	2.8%
Hoffman, 2009	0.47	[0.30; 0.73]	3.4%	3.2%
Hong, 2011	0.80	[0.54; 1.18]	3.6%	3.2%
Hu, 2013	0.64	[0.48; 0.87]	4.0%	6.2%
Hu, 2009	0.73	[0.57; 0.93]	4.1%	8.9%
Jedlinski, 2011	0.91	[0.50; 1.66]	2.8%	1.3%
Linhares, 2012	1.65	[1.13; 2.41]	3.7%	2.4%
Ma, 2013	1.02	[0.59; 1.77]	3.0%	1.4%
Mashayekhi, 2018	0.96	[0.59; 1.54]	3.3%	2.0%
Minh, 2018	0.61	[0.31; 1.19]	2.6%	1.2%
Morales, 2016	0.89	[0.62; 1.28]	3.7%	3.5%
Omrani, 2014			0.0%	0.0%
Parlayan, 2014	0.97	[0.56; 1.67]	3.0%	1.5%
Qi, 2015	0.45	[0.24; 0.84]	2.8%	1.8%
Sodhi, 2015	3.43	[1.42; 8.27]	2.0%	0.3%
Tian, 2009	0.79	[0.62; 1.01]	4.1%	8.3%
Toraih, 2016	1.65	[0.70; 3.88]	2.0%	0.5%
Vinci, 2011	1.99	[0.78; 5.08]	1.8%	0.3%
Yin, 2017	0.60	[0.47; 0.78]	4.1%	8.6%
Yin, 2016	0.87	[0.63; 1.20]	3.9%	4.4%
Yin, 2015	0.87	[0.54; 1.41]	3.3%	2.0%
Zhang, 2012	1.72	[0.78; 3.80]	2.2%	0.5%
Zhao, 2016	1.19	[0.58; 2.47]	2.4%	0.8%
Kim, 2010	0.73	[0.54; 0.98]	4.0%	5.7%
**Fixed effect model**	**0.84**	**[0.78; 0.90]**	**−−**	**100.0%**
**Random effects model**	**0.87**	**[0.76; 0.99]**	**100.0%**	**−−**
Heterogeneity: $I^2 = 57\%$, $\tau^2 = 0.0553$, $p < 0.01$				
Test for effect in subgroup (fixed effect): $z = -5.05$ ($p < 0.01$)				
Test for effect in subgroup (random effects): $t_{48} = -2.24$ ($p = 0.03$)				
**HNC**				
Christensen, 2010	0.92	[0.63; 1.32]	12.0%	10.7%
Chu, 2012	1.57	[1.04; 2.37]	11.4%	6.5%
Li, 2014	1.24	[0.97; 1.60]	13.3%	19.8%
Liu, 2013	1.41	[0.76; 2.59]	9.0%	3.1%
Liu, 2010	1.05	[0.82; 1.34]	13.4%	22.4%
Miao, 2016	0.72	[0.55; 0.95]	13.1%	21.5%
Roy, 2014	1.34	[0.84; 2.14]	10.7%	5.4%
Sushma, 2015	0.23	[0.09; 0.60]	5.7%	3.6%
Zhang, 2017	1.28	[0.84; 1.94]	11.3%	6.9%
**Fixed effect model**	**1.05**	**[0.94; 1.18]**	**−−**	**100.0%**
**Random effects model**	**1.06**	**[0.76; 1.46]**	**100.0%**	**−−**
Heterogeneity: $I^2 = 69\%$, $\tau^2 = 0.0746$, $p < 0.01$				
Test for effect in subgroup (fixed effect): $z = 0.85$ ($p = 0.40$)				
Test for effect in subgroup (random effects): $t_{48} = 0.39$ ($p = 0.71$)				
**UG**				
Damodaran, 2018	1.47	[0.65; 3.30]	6.7%	2.5%
Deng, 2015	0.93	[0.55; 1.60]	9.4%	7.1%
George, 2011	0.58	[0.15; 2.19]	3.5%	1.6%
Hashemi, 2016	1.70	[0.76; 3.83]	6.6%	2.3%
Horikawa, 2008	0.73	[0.46; 1.18]	10.2%	10.3%
Mittal, 2011	0.51	[0.18; 1.48]	4.8%	2.7%
Mulong Du, 2014	1.52	[1.17; 1.97]	12.6%	23.8%
Nikolic, 2015	0.79	[0.48; 1.29]	9.9%	9.0%
Parlayan, 2014	1.04	[0.53; 2.04]	8.0%	4.3%
Toraih−1, 2016	2.25	[0.93; 5.47]	6.0%	1.5%
Wang, 2016	0.49	[0.31; 0.79]	10.1%	12.4%
Yang, 2008	1.02	[0.75; 1.37]	12.2%	22.3%
**Fixed effect model**	**1.04**	**[0.91; 1.20]**	**−−**	**100.0%**
**Random effects model**	**0.98**	**[0.74; 1.31]**	**100.0%**	**−−**
Heterogeneity: $I^2 = 62\%$, $\tau^2 = 0.1088$, $p < 0.01$				
Test for effect in subgroup (fixed effect): $z = 0.58$ ($p = 0.56$)				
Test for effect in subgroup (random effects): $t_{48} = -0.13$ ($p = 0.90$)				
**HM**				
Li, 2015	0.53	[0.33; 0.84]	18.5%	22.0%
Martin−Guerrero, 2015	2.32	[1.28; 4.18]	15.9%	6.2%
Parlayan, 2014	0.70	[0.36; 1.36]	14.5%	9.3%
Peckham−Gregory, 2016	0.68	[0.38; 1.20]	16.3%	13.5%
Rakmanee, 2017	0.25	[0.12; 0.52]	13.4%	14.0%
Tong, 2014	0.84	[0.61; 1.17]	21.4%	34.9%
**Fixed effect model**	**0.75**	**[0.61; 0.91]**	**−−**	**100.0%**
**Random effects model**	**0.72**	**[0.35; 1.50]**	**100.0%**	**−−**
Heterogeneity: $I^2 = 80\%$, $\tau^2 = 0.2894$, $p < 0.01$				
Test for effect in subgroup (fixed effect): $z = -2.86$ ($p < 0.01$)				
Test for effect in subgroup (random effects): $t_{48} = -1.15$ ($p = 0.30$)				
**GyC**				
Liu, 2015	1.91	[0.92; 3.93]	13.8%	5.7%
Ni, 2016	0.85	[0.48; 1.48]	17.1%	14.7%
Song, 2016	0.56	[0.38; 0.81]	21.2%	41.0%
Srivastava, 2017	1.20	[0.60; 2.42]	14.2%	7.8%
Sun, 2016	0.59	[0.32; 1.11]	15.6%	13.7%
Zhou, 2011	0.88	[0.52; 1.47]	18.0%	17.0%
**Fixed effect model**	**0.79**	**[0.63; 0.98]**	**−−**	**100.0%**
**Random effects model**	**0.86**	**[0.54; 1.36]**	**100.0%**	**−−**
Heterogeneity: $I^2 = 57\%$, $\tau^2 = 0.1039$, $p = 0.04$				
Test for effect in subgroup (fixed effect): $z = -2.16$ ($p = 0.03$)				
Test for effect in subgroup (random effects): $t_{48} = -0.86$ ($p = 0.43$)				

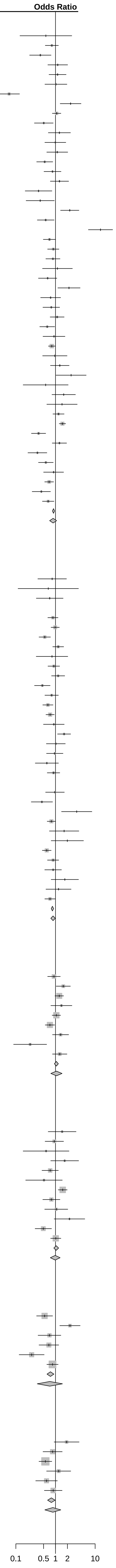

B) Heterozygote comparison

Study	OR	95%−CI	Weight (random)	Weight (fixed)
**GI**				
Abdel−Hamid, 2018	1.49	[0.65; 3.39]	1.0%	0.3%
Ahn, 2013	0.91	[0.65; 1.27]	2.4%	2.4%
Akkiz, 2011	0.74	[0.47; 1.17]	1.9%	1.5%
Chen, 2012	1.08	[0.65; 1.80]	1.7%	1.0%
Chu, 2014	0.83	[0.52; 1.32]	1.9%	1.3%
Dikaiakos, 2015	0.80	[0.43; 1.51]	1.4%	0.7%
Dikeakos, 2014	0.16	[0.10; 0.24]	2.0%	3.5%
Gu, 2016	1.43	[0.87; 2.35]	1.8%	0.9%
Han, 2013	1.11	[0.88; 1.39]	2.8%	4.8%
Hao, 2014	0.69	[0.46; 1.02]	2.1%	1.9%
Hezova, 2012	0.92	[0.61; 1.39]	2.1%	1.6%
Huang, 2017	1.07	[0.59; 1.93]	1.5%	0.7%
Kim, 2012	1.04	[0.61; 1.76]	1.7%	0.9%
Kou, 2014	0.73	[0.52; 1.03]	2.4%	2.6%
Kupcinskas, 2014	1.40	[1.02; 1.92]	2.5%	2.2%
Kupcinskas, 2014	1.26	[0.87; 1.82]	2.3%	1.7%
Li, 2016	0.48	[0.22; 1.05]	1.1%	0.7%
Li, 2016	0.89	[0.44; 1.80]	1.2%	0.6%
Li, 2015	1.43	[0.99; 2.08]	2.2%	1.6%
Li, 2010	0.79	[0.50; 1.24]	1.9%	1.4%
Lv, 2013	7.34	[3.76; 14.34]	1.3%	0.3%
Min, 2013	0.66	[0.48; 0.91]	2.4%	3.1%
Okubo, 2010	0.95	[0.70; 1.28]	2.5%	2.9%
Parlayan, 2014	0.78	[0.54; 1.13]	2.3%	2.1%
Pavlakis, 2013	0.66	[0.27; 1.60]	0.9%	0.4%
Peng, 2010	0.65	[0.42; 1.01]	2.0%	1.7%
Poltronieri−Oliveira, 2017	0.78	[0.50; 1.21]	2.0%	1.5%
Pu, 2014	0.76	[0.49; 1.17]	2.0%	1.5%
Qi, 2014	1.54	[1.01; 2.34]	2.1%	1.2%
Qi, 2010	1.02	[0.71; 1.46]	2.3%	2.0%
Qu, 2014	1.04	[0.76; 1.41]	2.5%	2.7%
Rogoveanu, 2017	0.98	[0.63; 1.50]	2.0%	1.4%
Shen, 2016	0.83	[0.69; 0.99]	3.0%	8.9%
Srivastava, 2010	1.45	[0.98; 2.14]	2.2%	1.4%
Su, 2016	1.16	[0.81; 1.67]	2.3%	1.8%
Toraih, 2016	1.26	[0.67; 2.36]	1.4%	0.6%
Toraih−2, 2016	1.93	[1.03; 3.62]	1.4%	0.5%
Umar, 2013	1.16	[0.83; 1.62]	2.4%	2.2%
Vinci, 2013	1.37	[0.88; 2.14]	2.0%	1.1%
Wang, 2014	1.17	[0.88; 1.55]	2.6%	2.9%
Wang, 2013	1.37	[1.16; 1.62]	3.0%	7.8%
Wang, 2010	0.91	[0.68; 1.22]	2.6%	3.2%
Wei, 2013	1.54	[1.05; 2.26]	2.2%	1.4%
Yan, 2015	0.52	[0.31; 0.88]	1.7%	1.3%
Zhan, 2011	0.80	[0.55; 1.15]	2.3%	2.1%
Zhang, 2016	1.03	[0.59; 1.82]	1.6%	0.8%
Zhang, 2013	0.75	[0.59; 0.95]	2.8%	5.3%
Zhou, 2014	0.62	[0.42; 0.91]	2.2%	2.2%
Zhu, 2012	0.89	[0.66; 1.19]	2.6%	3.3%
**Fixed effect model**	**0.98**	**[0.93; 1.03]**	**−−**	**100.0%**
**Random effects model**	**0.96**	**[0.84; 1.10]**	**100.0%**	**−−**
Heterogeneity: I^2 = 77%, τ^2 = 0.1153, p < 0.01				
Test for effect in subgroup (fixed effect): z = −0.77 (p = 0.44)				
Test for effect in subgroup (random effects): t_{48} = −0.55 (p = 0.58)				
**Others**				
Afsharzadeh, 2017	0.62	[0.35; 1.11]	2.1%	1.1%
Alshatwi, 2012	1.66	[0.93; 2.94]	2.1%	0.7%
Bansal, 2014	0.87	[0.52; 1.45]	2.4%	1.3%
Bodal, 2017	1.79	[1.01; 3.18]	2.1%	0.7%
Catucci, 1, 2010	0.96	[0.79; 1.16]	4.1%	8.3%
Catucci, 2, 2010	0.99	[0.84; 1.18]	4.2%	10.7%
Dai, 2016	0.73	[0.56; 0.96]	3.7%	4.9%
Dou, 2010	1.45	[1.08; 1.94]	3.6%	3.0%
Doulah, 2018	0.62	[0.33; 1.18]	1.9%	0.9%
He, 2018	0.94	[0.69; 1.27]	3.5%	3.4%
He, 2015	1.20	[0.85; 1.70]	3.2%	2.3%
Hoffman, 2009	0.84	[0.63; 1.11]	3.6%	4.2%
Hong, 2011	1.26	[0.89; 1.79]	3.2%	2.2%
Hu, 2013	0.68	[0.52; 0.90]	3.7%	5.1%
Hu, 2009	0.85	[0.68; 1.06]	3.9%	6.7%
Jedlinski, 2011	0.89	[0.56; 1.42]	2.6%	1.5%
Linhares, 2012	1.45	[1.03; 2.04]	3.3%	2.2%
Ma, 2013	1.30	[0.78; 2.15]	2.4%	1.0%
Mashayekhi, 2018	1.12	[0.82; 1.54]	3.4%	2.9%
Minh, 2018	0.35	[0.19; 0.65]	2.0%	1.4%
Morales, 2016	0.97	[0.76; 1.24]	3.8%	4.9%
Omrani, 2014	0.59	[0.31; 1.11]	1.9%	1.0%
Parlayan, 2014	1.12	[0.69; 1.80]	2.5%	1.3%
Qi, 2015	0.68	[0.36; 1.29]	1.9%	0.9%
Sodhi, 2015	1.59	[1.09; 2.32]	3.1%	1.7%
Tian, 2009	0.81	[0.65; 1.02]	4.0%	6.9%
Toraih, 2016	1.62	[0.93; 2.82]	2.2%	0.8%
Vinci, 2011	1.47	[0.84; 2.56]	2.2%	0.8%
Yin, 2017	0.98	[0.79; 1.22]	4.0%	6.5%
Yin, 2016	1.04	[0.78; 1.39]	3.6%	3.5%
Yin, 2015	1.18	[0.77; 1.83]	2.8%	1.5%
Zhang, 2012	1.48	[0.66; 3.33]	1.4%	0.4%
Zhao, 2016	0.74	[0.39; 1.39]	1.9%	0.9%
Kim, 2010	0.84	[0.65; 1.10]	3.7%	4.7%
**Fixed effect model**	**0.97**	**[0.92; 1.03]**	**−−**	**100.0%**
**Random effects model**	**0.99**	**[0.89; 1.10]**	**100.0%**	**−−**
Heterogeneity: I^2 = 57%, τ^2 = 0.0367, p < 0.01				
Test for effect in subgroup (fixed effect): z = −1.01 (p = 0.31)				
Test for effect in subgroup (random effects): t_{48} = −0.20 (p = 0.85)				
**HNC**				
Christensen, 2010	0.83	[0.63; 1.09]	13.1%	14.3%
Chu, 2012	2.05	[1.40; 3.00]	10.9%	4.6%
Li, 2014	0.98	[0.79; 1.23]	13.9%	18.6%
Liu, 2013	1.66	[0.93; 2.97]	7.4%	2.1%
Liu, 2010	1.13	[0.94; 1.37]	14.7%	25.5%
Miao, 2016	0.84	[0.66; 1.08]	13.5%	16.6%
Roy, 2014	1.24	[0.94; 1.63]	12.9%	11.1%
Sushma, 2015	0.18	[0.05; 0.61]	2.7%	1.5%
Zhang, 2017	1.35	[0.92; 1.98]	10.9%	5.6%
**Fixed effect model**	**1.08**	**[0.98; 1.18]**	**−−**	**100.0%**
**Random effects model**	**1.11**	**[0.79; 1.54]**	**100.0%**	**−−**
Heterogeneity: I^2 = 75%, τ^2 = 0.0688, p < 0.01				
Test for effect in subgroup (fixed effect): z = 1.52 (p = 0.13)				
Test for effect in subgroup (random effects): t_{48} = 0.69 (p = 0.51)				
**UG**				
Damodaran, 2018	2.08	[1.12; 3.87]	5.7%	2.2%
Deng, 2015	0.54	[0.33; 0.89]	7.3%	6.6%
George, 2011	1.71	[1.12; 2.60]	8.3%	5.3%
Hashemi, 2016	1.14	[0.73; 1.77]	8.0%	5.9%
Horikawa, 2008	1.04	[0.71; 1.50]	9.1%	8.7%
Mittal, 2011	1.48	[1.01; 2.17]	9.0%	6.9%
Mulong Du, 2014	1.46	[1.15; 1.87]	11.2%	17.1%
Nikolic, 2015	0.88	[0.64; 1.23]	9.8%	12.1%
Parlayan, 2014	1.13	[0.62; 2.05]	6.0%	3.3%
Toraih−1, 2016	2.03	[1.07; 3.86]	5.5%	2.1%
Wang, 2016	1.13	[0.75; 1.71]	8.5%	6.8%
Yang, 2008	1.03	[0.82; 1.29]	11.5%	23.0%
**Fixed effect model**	**1.18**	**[1.06; 1.31]**	**−−**	**100.0%**
**Random effects model**	**1.20**	**[0.96; 1.49]**	**100.0%**	**−−**
Heterogeneity: I^2 = 60%, τ^2 = 0.0567, p < 0.01				
Test for effect in subgroup (fixed effect): z = 3.09 (p < 0.01)				
Test for effect in subgroup (random effects): t_{48} = 1.80 (p = 0.10)				
**HM**				
Li, 2015	0.75	[0.47; 1.19]	16.6%	15.9%
Martin−Guerrero, 2015	0.98	[0.59; 1.64]	15.1%	11.2%
Parlayan, 2014	0.59	[0.32; 1.07]	12.8%	9.8%
Peckham−Gregory, 2016	0.93	[0.65; 1.34]	19.7%	22.5%
Rakmanee, 2017	0.56	[0.33; 0.97]	14.1%	12.8%
Tong, 2014	1.26	[0.93; 1.70]	21.8%	27.8%
**Fixed effect model**	**0.92**	**[0.77; 1.09]**	**−−**	**100.0%**
**Random effects model**	**0.86**	**[0.62; 1.19]**	**100.0%**	**−−**
Heterogeneity: I^2 = 51%, τ^2 = 0.0508, p = 0.07				
Test for effect in subgroup (fixed effect): z = −0.97 (p = 0.33)				
Test for effect in subgroup (random effects): t_{48} = −1.19 (p = 0.29)				
**GyC**				
Liu, 2015	2.50	[1.30; 4.80]	13.0%	5.4%
Ni, 2016	0.96	[0.58; 1.58]	17.4%	15.9%
Song, 2016	0.86	[0.62; 1.21]	23.4%	37.6%
Srivastava, 2017	1.21	[0.61; 2.38]	12.3%	7.6%
Sun, 2016	0.67	[0.37; 1.19]	14.9%	13.8%
Zhou, 2011	0.92	[0.58; 1.44]	19.0%	19.7%
**Fixed effect model**	**0.98**	**[0.80; 1.19]**	**−−**	**100.0%**
**Random effects model**	**1.02**	**[0.66; 1.58]**	**100.0%**	**−−**
Heterogeneity: I^2 = 52%, τ^2 = 0.0723, p = 0.06				
Test for effect in subgroup (fixed effect): z = −0.24 (p = 0.81)				
Test for effect in subgroup (random effects): t_{48} = 0.14 (p = 0.89)				

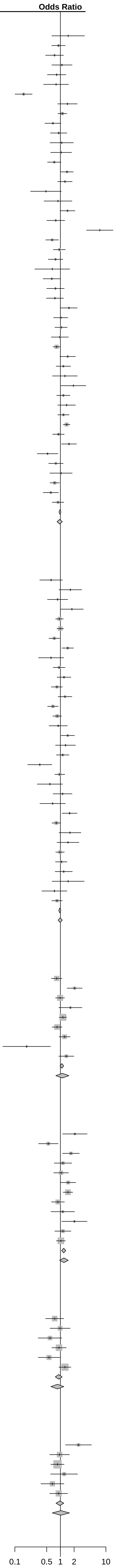

C) Dominant comparison

GI

Study	OR	95%−CI	Weight (random)	Weight (fixed)
Abdel−Hamid, 2018	1.27	[0.58; 2.81]	1.1%	0.3%
Ahn, 2013	0.87	[0.63; 1.20]	2.3%	2.4%
Akkiz, 2011	0.64	[0.42; 0.98]	2.0%	1.6%
Chen, 2012	1.10	[0.68; 1.79]	1.8%	0.9%
Chu, 2014	0.94	[0.61; 1.45]	2.0%	1.2%
Dikaiakos, 2015	0.90	[0.49; 1.64]	1.5%	0.7%
Dikeakos, 2014	0.12	[0.08; 0.18]	2.1%	3.8%
Gu, 2016	1.67	[1.04; 2.67]	1.8%	0.8%
Han, 2013	1.09	[0.88; 1.35]	2.7%	4.8%
Hao, 2014	0.64	[0.43; 0.94]	2.1%	1.9%
Hezova, 2012	0.98	[0.66; 1.45]	2.1%	1.5%
Huang, 2017	1.03	[0.59; 1.81]	1.6%	0.7%
Kim, 2012	1.06	[0.64; 1.75]	1.7%	0.9%
Kou, 2014	0.68	[0.49; 0.95]	2.3%	2.6%
Kupcinskas, 2014	1.27	[0.94; 1.70]	2.4%	2.3%
Kupcinskas, 2014	1.26	[0.89; 1.78]	2.3%	1.7%
Li, 2016	0.42	[0.20; 0.89]	1.2%	0.7%
Li, 2016	0.70	[0.35; 1.37]	1.3%	0.6%
Li, 2015	1.60	[1.12; 2.28]	2.2%	1.4%
Li, 2010	0.69	[0.45; 1.06]	2.0%	1.6%
Lv, 2013	8.70	[4.48; 16.90]	1.3%	0.3%
Min, 2013	0.68	[0.50; 0.91]	2.4%	3.0%
Okubo, 2010	0.92	[0.69; 1.23]	2.4%	2.9%
Parlayan, 2014	0.81	[0.57; 1.15]	2.2%	2.1%
Pavlakis, 2013	0.88	[0.38; 1.99]	1.0%	0.4%
Peng, 2010	0.64	[0.42; 0.97]	2.0%	1.7%
Poltronieri−Oliveira, 2017	0.99	[0.66; 1.49]	2.0%	1.4%
Pu, 2014	0.76	[0.50; 1.16]	2.0%	1.4%
Qi, 2014	1.27	[0.84; 1.90]	2.0%	1.3%
Qi, 2010	1.05	[0.75; 1.47]	2.3%	2.0%
Qu, 2014	0.92	[0.68; 1.23]	2.4%	2.7%
Rogoveanu, 2017	0.96	[0.64; 1.44]	2.0%	1.4%
Shen, 2016	0.82	[0.69; 0.97]	2.8%	8.8%
Srivastava, 2010	1.35	[0.93; 1.95]	2.2%	1.5%
Su, 2016	1.19	[0.84; 1.68]	2.2%	1.7%
Toraih, 2016	1.53	[0.86; 2.71]	1.6%	0.6%
Toraih−2, 2016	1.60	[0.87; 2.93]	1.5%	0.5%
Umar, 2013	1.21	[0.88; 1.67]	2.3%	2.0%
Vinci, 2013	1.38	[0.90; 2.13]	2.0%	1.0%
Wang, 2014	1.18	[0.90; 1.54]	2.5%	2.9%
Wang, 2013	1.41	[1.21; 1.66]	2.8%	7.7%
Wang, 2010	0.74	[0.56; 0.98]	2.5%	3.4%
Wei, 2013	1.43	[1.00; 2.05]	2.2%	1.5%
Yan, 2015	0.44	[0.27; 0.74]	1.7%	1.4%
Zhan, 2011	0.71	[0.50; 1.01]	2.2%	2.2%
Zhang, 2016	0.97	[0.57; 1.65]	1.7%	0.8%
Zhang, 2013	0.73	[0.58; 0.91]	2.6%	5.3%
Zhou, 2014	0.57	[0.39; 0.83]	2.2%	2.2%
Zhu, 2012	0.80	[0.61; 1.06]	2.5%	3.4%
**Fixed effect model**	**0.96**	**[0.91; 1.00]**	**−−**	**100.0%**
**Random effects model**	**0.94**	**[0.81; 1.09]**	**100.0%**	**−−**

Heterogeneity: I^2 = 83%, τ^2 = 0.1518, p < 0.01
Test for effect in subgroup (fixed effect): z = −1.82 (p = 0.07)
Test for effect in subgroup (random effects): t_{48} = −0.86 (p = 0.40)

Others

Study	OR	95%−CI	Weight (random)	Weight (fixed)
Afsharzadeh, 2017	0.66	[0.38; 1.15]	2.2%	1.0%
Alshatwi, 2012	1.58	[0.90; 2.79]	2.2%	0.7%
Bansal, 2014	0.83	[0.52; 1.33]	2.6%	1.3%
Bodal, 2017	1.79	[1.01; 3.18]	2.2%	0.6%
Catucci, 1, 2010	0.93	[0.78; 1.12]	3.9%	8.2%
Catucci, 2, 2010	0.99	[0.85; 1.16]	3.9%	10.5%
Dai, 2016	0.67	[0.52; 0.86]	3.6%	5.1%
Dou, 2010	1.34	[1.01; 1.76]	3.5%	3.0%
Doulah, 2018	0.66	[0.35; 1.22]	2.0%	0.9%
He, 2018	0.93	[0.70; 1.23]	3.4%	3.4%
He, 2015	1.19	[0.85; 1.65]	3.2%	2.2%
Hoffman, 2009	0.75	[0.57; 0.98]	3.5%	4.2%
Hong, 2011	1.08	[0.77; 1.50]	3.2%	2.4%
Hu, 2013	0.67	[0.52; 0.86]	3.6%	5.1%
Hu, 2009	0.80	[0.65; 0.99]	3.8%	6.9%
Jedlinski, 2011	0.90	[0.58; 1.39]	2.7%	1.5%
Linhares, 2012	1.52	[1.11; 2.08]	3.3%	2.2%
Ma, 2013	1.18	[0.74; 1.88]	2.6%	1.1%
Mashayekhi, 2018	1.09	[0.80; 1.46]	3.4%	2.8%
Minh, 2018	0.44	[0.25; 0.76]	2.3%	1.3%
Morales, 2016	0.95	[0.75; 1.20]	3.6%	4.9%
Omrani, 2014	0.59	[0.31; 1.11]	1.9%	0.9%
Parlayan, 2014	1.07	[0.67; 1.68]	2.6%	1.2%
Qi, 2015	0.53	[0.29; 0.96]	2.1%	1.0%
Sodhi, 2015	1.69	[1.16; 2.45]	3.0%	1.5%
Tian, 2009	0.81	[0.65; 0.99]	3.8%	6.9%
Toraih, 2016	1.63	[0.97; 2.73]	2.4%	0.8%
Vinci, 2011	1.54	[0.90; 2.64]	2.3%	0.7%
Yin, 2017	0.84	[0.69; 1.04]	3.8%	6.8%
Yin, 2016	0.98	[0.74; 1.29]	3.5%	3.5%
Yin, 2015	1.06	[0.70; 1.61]	2.8%	1.5%
Zhang, 2012	1.62	[0.74; 3.54]	1.5%	0.3%
Zhao, 2016	0.87	[0.48; 1.58]	2.1%	0.8%
Kim, 2010	0.80	[0.62; 1.02]	3.6%	4.8%
**Fixed effect model**	**0.93**	**[0.88; 0.98]**	**−−**	**100.0%**
**Random effects model**	**0.96**	**[0.86; 1.07]**	**100.0%**	**−−**

Heterogeneity: I^2 = 62%, τ^2 = 0.0415, p < 0.01
Test for effect in subgroup (fixed effect): z = −2.63 (p < 0.01)
Test for effect in subgroup (random effects): t_{48} = −0.84 (p = 0.41)

HNC

Study	OR	95%−CI	Weight (random)	Weight (fixed)
Christensen, 2010	0.85	[0.66; 1.10]	12.7%	14.0%
Chu, 2012	1.86	[1.30; 2.68]	10.9%	4.7%
Li, 2014	1.07	[0.87; 1.33]	13.3%	17.8%
Liu, 2013	1.54	[0.91; 2.63]	8.3%	2.3%
Liu, 2010	1.11	[0.93; 1.33]	13.8%	25.5%
Miao, 2016	0.80	[0.63; 1.01]	13.0%	16.8%
Roy, 2014	1.26	[0.97; 1.63]	12.6%	10.9%
Sushma, 2015	0.22	[0.09; 0.57]	4.2%	2.3%
Zhang, 2017	1.32	[0.93; 1.89]	11.0%	5.7%
**Fixed effect model**	**1.07**	**[0.98; 1.17]**	**−−**	**100.0%**
**Random effects model**	**1.08**	**[0.78; 1.50]**	**100.0%**	**−−**

Heterogeneity: I^2 = 76%, τ^2 = 0.0650, p < 0.01
Test for effect in subgroup (fixed effect): z = 1.43 (p = 0.15)
Test for effect in subgroup (random effects): t_{48} = 0.57 (p = 0.59)

UG

Study	OR	95%−CI	Weight (random)	Weight (fixed)
Damodaran, 2018	1.88	[1.06; 3.35]	6.3%	2.4%
Deng, 2015	0.67	[0.42; 1.05]	7.6%	6.1%
George, 2011	1.62	[1.07; 2.45]	8.2%	4.9%
Hashemi, 2016	1.20	[0.78; 1.85]	8.0%	5.4%
Horikawa, 2008	0.93	[0.66; 1.32]	9.1%	9.4%
Mittal, 2011	1.38	[0.95; 2.01]	8.7%	6.5%
Mulong Du, 2014	1.49	[1.18; 1.87]	10.6%	16.8%
Nikolic, 2015	0.86	[0.63; 1.18]	9.6%	12.0%
Parlayan, 2014	1.10	[0.62; 1.94]	6.3%	3.2%
Toraih−1, 2016	2.09	[1.14; 3.81]	6.0%	2.1%
Wang, 2016	0.86	[0.58; 1.26]	8.6%	7.9%
Yang, 2008	1.02	[0.83; 1.27]	10.8%	23.2%
**Fixed effect model**	**1.15**	**[1.04; 1.27]**	**−−**	**100.0%**
**Random effects model**	**1.16**	**[0.94; 1.42]**	**100.0%**	**−−**

Heterogeneity: I^2 = 61%, τ^2 = 0.0529, p < 0.01
Test for effect in subgroup (fixed effect): z = 2.66 (p < 0.01)
Test for effect in subgroup (random effects): t_{48} = 1.57 (p = 0.14)

HM

Study	OR	95%−CI	Weight (random)	Weight (fixed)
Li, 2015	0.64	[0.41; 0.98]	16.6%	16.8%
Martin−Guerrero, 2015	1.30	[0.82; 2.06]	15.8%	10.2%
Parlayan, 2014	0.63	[0.36; 1.09]	13.6%	9.2%
Peckham−Gregory, 2016	0.87	[0.62; 1.24]	18.9%	21.4%
Rakmanee, 2017	0.44	[0.26; 0.72]	14.6%	14.0%
Tong, 2014	1.08	[0.81; 1.43]	20.5%	28.4%
**Fixed effect model**	**0.85**	**[0.72; 1.00]**	**−−**	**100.0%**
**Random effects model**	**0.80**	**[0.53; 1.20]**	**100.0%**	**−−**

Heterogeneity: I^2 = 68%, τ^2 = 0.0902, p < 0.01
Test for effect in subgroup (fixed effect): z = −1.95 (p = 0.05)
Test for effect in subgroup (random effects): t_{48} = −1.43 (p = 0.21)

GyC

Study	OR	95%−CI	Weight (random)	Weight (fixed)
Liu, 2015	2.28	[1.22; 4.26]	13.5%	5.3%
Ni, 2016	0.92	[0.57; 1.47]	17.4%	15.4%
Song, 2016	0.74	[0.54; 1.01]	22.3%	39.9%
Srivastava, 2017	1.20	[0.63; 2.31]	12.9%	7.1%
Sun, 2016	0.64	[0.37; 1.10]	15.3%	13.5%
Zhou, 2011	0.90	[0.59; 1.39]	18.6%	18.8%
**Fixed effect model**	**0.90**	**[0.74; 1.08]**	**−−**	**100.0%**
**Random effects model**	**0.96**	**[0.62; 1.50]**	**100.0%**	**−−**

Heterogeneity: I^2 = 59%, τ^2 = 0.0868, p = 0.03
Test for effect in subgroup (fixed effect): z = −1.12 (p = 0.26)
Test for effect in subgroup (random effects): t_{48} = −0.21 (p = 0.84)

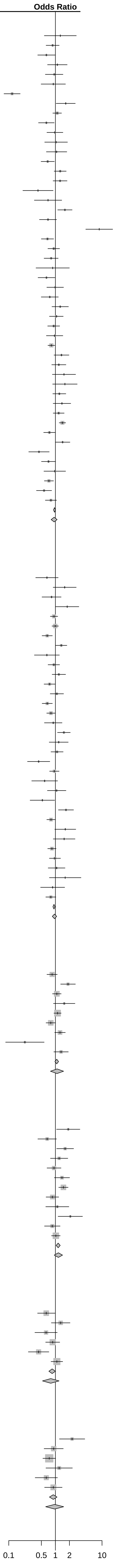

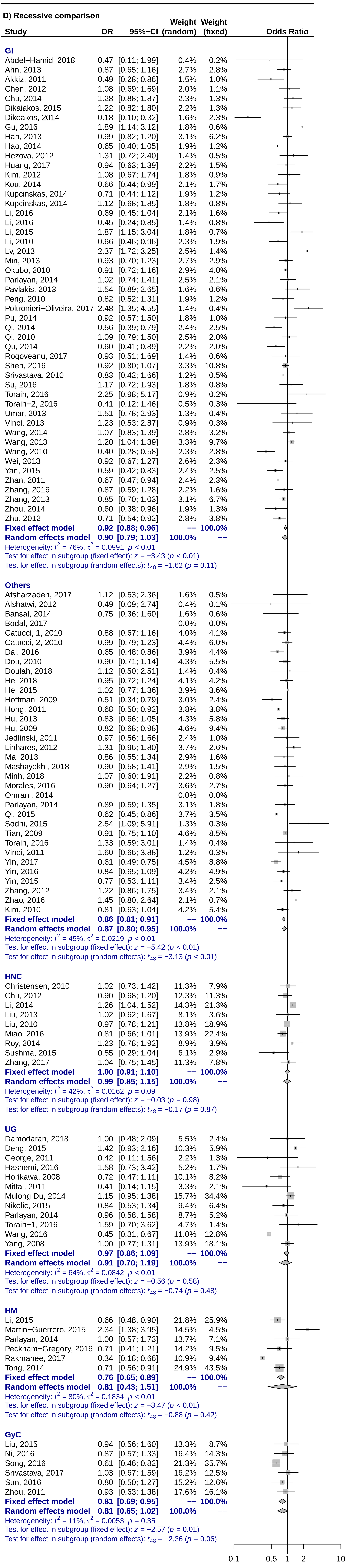
D) Recessive comparison
Study OR 95%−CI Weight (random) Weight (fixed) Odds Ratio
GI
Abdel−Hamid, 2018 0.47 [0.11; 1.99] 0.4% 0.2%
Ahn, 2013 0.87 [0.65; 1.16] 2.7% 2.8%
Akkiz, 2011 0.49 [0.28; 0.86] 1.5% 1.0%
Chen, 2012 1.08 [0.69; 1.69] 2.0% 1.1%
Chu, 2014 1.28 [0.88; 1.87] 2.3% 1.3%
Dikaiakos, 2015 1.22 [0.82; 1.80] 2.2% 1.3%
Dikeakos, 2014 0.18 [0.10; 0.32] 1.6% 2.3%
Gu, 2016 1.89 [1.14; 3.12] 1.8% 0.6%
Han, 2013 0.99 [0.82; 1.20] 3.1% 6.2%
Hao, 2014 0.65 [0.40; 1.05] 1.9% 1.2%
Hezova, 2012 1.31 [0.72; 2.40] 1.4% 0.5%
Huang, 2017 0.94 [0.63; 1.39] 2.2% 1.5%
Kim, 2012 1.08 [0.67; 1.74] 1.8% 0.9%
Kou, 2014 0.66 [0.44; 0.99] 2.1% 1.7%
Kupcinskas, 2014 0.71 [0.44; 1.12] 1.9% 1.2%
Kupcinskas, 2014 1.12 [0.68; 1.85] 1.8% 0.8%
Li, 2016 0.69 [0.45; 1.04] 2.1% 1.6%
Li, 2016 0.45 [0.24; 0.85] 1.4% 0.8%
Li, 2015 1.87 [1.15; 3.04] 1.8% 0.7%
Li, 2010 0.66 [0.46; 0.96] 2.3% 1.9%
Lv, 2013 2.37 [1.72; 3.25] 2.5% 1.4%
Min, 2013 0.93 [0.70; 1.23] 2.7% 2.9%
Okubo, 2010 0.91 [0.72; 1.16] 2.9% 4.0%
Parlayan, 2014 1.02 [0.74; 1.41] 2.5% 2.1%
Pavlakis, 2013 1.54 [0.89; 2.65] 1.6% 0.6%
Peng, 2010 0.82 [0.52; 1.31] 1.9% 1.2%
Poltronieri−Oliveira, 2017 2.48 [1.35; 4.55] 1.4% 0.4%
Pu, 2014 0.92 [0.57; 1.50] 1.8% 1.0%
Qi, 2014 0.56 [0.39; 0.79] 2.4% 2.5%
Qi, 2010 1.09 [0.79; 1.50] 2.5% 2.0%
Qu, 2014 0.60 [0.41; 0.89] 2.2% 2.0%
Rogoveanu, 2017 0.93 [0.51; 1.69] 1.4% 0.6%
Shen, 2016 0.92 [0.80; 1.07] 3.3% 10.8%
Srivastava, 2010 0.83 [0.42; 1.66] 1.2% 0.5%
Su, 2016 1.17 [0.72; 1.93] 1.8% 0.8%
Toraih, 2016 2.25 [0.98; 5.17] 0.9% 0.2%
Toraih−2, 2016 0.41 [0.12; 1.46] 0.5% 0.3%
Umar, 2013 1.51 [0.78; 2.93] 1.3% 0.4%
Vinci, 2013 1.23 [0.53; 2.87] 0.9% 0.3%
Wang, 2014 1.07 [0.83; 1.39] 2.8% 3.2%
Wang, 2013 1.20 [1.04; 1.39] 3.3% 9.7%
Wang, 2010 0.40 [0.28; 0.58] 2.3% 2.8%
Wei, 2013 0.92 [0.67; 1.27] 2.6% 2.3%
Yan, 2015 0.59 [0.42; 0.83] 2.4% 2.5%
Zhan, 2011 0.67 [0.47; 0.94] 2.4% 2.3%
Zhang, 2016 0.87 [0.59; 1.28] 2.2% 1.6%
Zhang, 2013 0.85 [0.70; 1.03] 3.1% 6.7%
Zhou, 2014 0.60 [0.38; 0.96] 1.9% 1.3%
Zhu, 2012 0.71 [0.54; 0.92] 2.8% 3.8%
Fixed effect model 0.92 [0.88; 0.96] −− 100.0%
Random effects model 0.90 [0.79; 1.03] 100.0% −−
Heterogeneity: I^2 = 76%, τ^2 = 0.0991, p < 0.01
Test for effect in subgroup (fixed effect): z = −3.43 (p < 0.01)
Test for effect in subgroup (random effects): t_{48} = −1.62 (p = 0.11)
Others
Afsharzadeh, 2017 1.12 [0.53; 2.36] 1.6% 0.5%
Alshatwi, 2012 0.49 [0.09; 2.74] 0.4% 0.1%
Bansal, 2014 0.75 [0.36; 1.60] 1.6% 0.6%
Bodal, 2017 0.0% 0.0%
Catucci, 1, 2010 0.88 [0.67; 1.16] 4.0% 4.1%
Catucci, 2, 2010 0.99 [0.79; 1.23] 4.4% 6.0%
Dai, 2016 0.65 [0.48; 0.86] 3.9% 4.4%
Dou, 2010 0.90 [0.71; 1.14] 4.3% 5.5%
Doulah, 2018 1.12 [0.50; 2.51] 1.4% 0.4%
He, 2018 0.95 [0.72; 1.24] 4.1% 4.2%
He, 2015 1.02 [0.77; 1.36] 3.9% 3.6%
Hoffman, 2009 0.51 [0.34; 0.79] 3.0% 2.4%
Hong, 2011 0.68 [0.50; 0.92] 3.8% 3.8%
Hu, 2013 0.83 [0.66; 1.05] 4.3% 5.8%
Hu, 2009 0.82 [0.68; 0.98] 4.6% 9.4%
Jedlinski, 2011 0.97 [0.56; 1.66] 2.4% 1.0%
Linhares, 2012 1.31 [0.96; 1.80] 3.7% 2.6%
Ma, 2013 0.86 [0.55; 1.34] 2.9% 1.6%
Mashayekhi, 2018 0.90 [0.58; 1.41] 2.9% 1.5%
Minh, 2018 1.07 [0.60; 1.91] 2.2% 0.8%
Morales, 2016 0.90 [0.64; 1.27] 3.6% 2.7%
Omrani, 2014 0.0% 0.0%
Parlayan, 2014 0.89 [0.59; 1.35] 3.1% 1.8%
Qi, 2015 0.62 [0.45; 0.86] 3.7% 3.5%
Sodhi, 2015 2.54 [1.09; 5.91] 1.3% 0.3%
Tian, 2009 0.91 [0.75; 1.10] 4.6% 8.5%
Toraih, 2016 1.33 [0.59; 3.01] 1.4% 0.4%
Vinci, 2011 1.60 [0.66; 3.88] 1.2% 0.3%
Yin, 2017 0.61 [0.49; 0.75] 4.5% 8.8%
Yin, 2016 0.84 [0.65; 1.09] 4.2% 4.9%
Yin, 2015 0.77 [0.53; 1.11] 3.4% 2.5%
Zhang, 2012 1.22 [0.86; 1.75] 3.4% 2.1%
Zhao, 2016 1.45 [0.80; 2.64] 2.1% 0.7%
Kim, 2010 0.81 [0.63; 1.04] 4.2% 5.4%
Fixed effect model 0.86 [0.81; 0.91] −− 100.0%
Random effects model 0.87 [0.80; 0.95] 100.0% −−
Heterogeneity: I^2 = 45%, τ^2 = 0.0219, p < 0.01
Test for effect in subgroup (fixed effect): z = −5.42 (p < 0.01)
Test for effect in subgroup (random effects): t_{48} = −3.13 (p < 0.01)
HNC
Christensen, 2010 1.02 [0.73; 1.42] 11.3% 7.9%
Chu, 2012 0.90 [0.68; 1.20] 12.3% 11.3%
Li, 2014 1.26 [1.04; 1.52] 14.3% 21.3%
Liu, 2013 1.02 [0.62; 1.67] 8.1% 3.6%
Liu, 2010 0.97 [0.78; 1.21] 13.8% 18.9%
Miao, 2016 0.81 [0.66; 1.01] 13.9% 22.4%
Roy, 2014 1.23 [0.78; 1.92] 8.9% 3.9%
Sushma, 2015 0.55 [0.29; 1.04] 6.1% 2.9%
Zhang, 2017 1.04 [0.75; 1.45] 11.3% 7.8%
Fixed effect model 1.00 [0.91; 1.10] −− 100.0%
Random effects model 0.99 [0.85; 1.15] 100.0% −−
Heterogeneity: I^2 = 42%, τ^2 = 0.0162, p = 0.09
Test for effect in subgroup (fixed effect): z = −0.03 (p = 0.98)
Test for effect in subgroup (random effects): t_{48} = −0.17 (p = 0.87)
UG
Damodaran, 2018 1.00 [0.48; 2.09] 5.5% 2.4%
Deng, 2015 1.42 [0.93; 2.16] 10.3% 5.9%
George, 2011 0.42 [0.11; 1.56] 2.2% 1.3%
Hashemi, 2016 1.58 [0.73; 3.42] 5.2% 1.7%
Horikawa, 2008 0.72 [0.47; 1.11] 10.1% 8.2%
Mittal, 2011 0.41 [0.14; 1.15] 3.3% 2.1%
Mulong Du, 2014 1.15 [0.95; 1.38] 15.7% 34.4%
Nikolic, 2015 0.84 [0.53; 1.34] 9.4% 6.4%
Parlayan, 2014 0.96 [0.58; 1.58] 8.7% 5.2%
Toraih−1, 2016 1.59 [0.70; 3.62] 4.7% 1.4%
Wang, 2016 0.45 [0.31; 0.67] 11.0% 12.8%
Yang, 2008 1.00 [0.77; 1.31] 13.9% 18.1%
Fixed effect model 0.97 [0.86; 1.09] −− 100.0%
Random effects model 0.91 [0.70; 1.19] 100.0% −−
Heterogeneity: I^2 = 64%, τ^2 = 0.0842, p < 0.01
Test for effect in subgroup (fixed effect): z = −0.56 (p = 0.58)
Test for effect in subgroup (random effects): t_{48} = −0.74 (p = 0.48)
HM
Li, 2015 0.66 [0.48; 0.90] 21.8% 25.9%
Martin−Guerrero, 2015 2.34 [1.38; 3.95] 14.5% 4.5%
Parlayan, 2014 1.00 [0.57; 1.73] 13.7% 7.1%
Peckham−Gregory, 2016 0.71 [0.41; 1.21] 14.2% 9.5%
Rakmanee, 2017 0.34 [0.18; 0.66] 10.9% 9.4%
Tong, 2014 0.71 [0.56; 0.91] 24.9% 43.5%
Fixed effect model 0.76 [0.65; 0.89] −− 100.0%
Random effects model 0.81 [0.43; 1.51] 100.0% −−
Heterogeneity: I^2 = 80%, τ^2 = 0.1834, p < 0.01
Test for effect in subgroup (fixed effect): z = −3.47 (p < 0.01)
Test for effect in subgroup (random effects): t_{48} = −0.88 (p = 0.42)
GyC
Liu, 2015 0.94 [0.56; 1.60] 13.3% 8.7%
Ni, 2016 0.87 [0.57; 1.33] 16.4% 14.3%
Song, 2016 0.61 [0.46; 0.82] 21.3% 35.7%
Srivastava, 2017 1.03 [0.67; 1.59] 16.2% 12.5%
Sun, 2016 0.80 [0.50; 1.27] 15.2% 12.6%
Zhou, 2011 0.93 [0.63; 1.38] 17.6% 16.1%
Fixed effect model 0.81 [0.69; 0.95] −− 100.0%
Random effects model 0.81 [0.65; 1.02] 100.0% −−
Heterogeneity: I^2 = 11%, τ^2 = 0.0053, p = 0.35
Test for effect in subgroup (fixed effect): z = −2.57 (p = 0.01)
Test for effect in subgroup (random effects): t_{48} = −2.36 (p = 0.06)
0.1 0.5 1 2 10

E) Allelic comparison

Study	OR	95%−CI	Weight (random)	Weight (fixed)
**GI**				
Abdel−Hamid, 2018	1.00	[0.55; 1.81]	0.9%	0.2%
Ahn, 2013	0.90	[0.75; 1.09]	2.4%	2.6%
Akkiz, 2011	0.66	[0.49; 0.89]	1.8%	1.2%
Chen, 2012	1.07	[0.80; 1.41]	1.9%	1.0%
Chu, 2014	1.09	[0.85; 1.41]	2.0%	1.3%
Dikaiakos, 2015	1.09	[0.82; 1.45]	1.9%	1.0%
Dikeakos, 2014	0.21	[0.15; 0.27]	1.9%	2.5%
Gu, 2016	1.51	[1.13; 2.01]	1.9%	0.8%
Han, 2013	1.03	[0.91; 1.16]	2.6%	5.5%
Hao, 2014	0.74	[0.58; 0.95]	2.1%	1.6%
Hezova, 2012	1.05	[0.79; 1.40]	1.9%	1.0%
Huang, 2017	0.98	[0.74; 1.29]	1.9%	1.1%
Kim, 2012	1.05	[0.78; 1.41]	1.8%	1.0%
Kou, 2014	0.77	[0.62; 0.94]	2.2%	2.2%
Kupcinskas, 2014	1.05	[0.84; 1.31]	2.2%	1.7%
Kupcinskas, 2014	1.17	[0.91; 1.50]	2.0%	1.2%
Li, 2016	0.68	[0.50; 0.93]	1.7%	1.0%
Li, 2016	0.66	[0.45; 0.96]	1.5%	0.7%
Li, 2015	1.49	[1.16; 1.90]	2.1%	1.1%
Li, 2010	0.74	[0.58; 0.95]	2.1%	1.6%
Lv, 2013	1.99	[1.63; 2.42]	2.3%	1.6%
Min, 2013	0.84	[0.70; 1.01]	2.4%	2.8%
Okubo, 2010	0.94	[0.80; 1.10]	2.5%	3.4%
Parlayan, 2014	0.94	[0.76; 1.15]	2.3%	2.1%
Pavlakis, 2013	1.23	[0.82; 1.85]	1.4%	0.5%
Peng, 2010	0.77	[0.59; 1.01]	1.9%	1.3%
Poltronieri−Oliveira, 2017	1.24	[0.92; 1.68]	1.8%	0.8%
Pu, 2014	0.89	[0.69; 1.14]	2.0%	1.4%
Qi, 2014	0.86	[0.70; 1.06]	2.2%	2.1%
Qi, 2010	1.05	[0.86; 1.29]	2.3%	2.0%
Qu, 2014	0.84	[0.69; 1.02]	2.3%	2.4%
Rogoveanu, 2017	0.96	[0.71; 1.29]	1.8%	1.0%
Shen, 2016	0.91	[0.82; 1.00]	2.7%	9.8%
Srivastava, 2010	1.17	[0.87; 1.57]	1.8%	0.9%
Su, 2016	1.13	[0.89; 1.44]	2.1%	1.4%
Toraih, 2016	1.60	[1.04; 2.47]	1.3%	0.4%
Toraih−2, 2016	1.13	[0.72; 1.79]	1.2%	0.4%
Umar, 2013	1.20	[0.93; 1.56]	2.0%	1.2%
Vinci, 2013	1.24	[0.89; 1.71]	1.7%	0.7%
Wang, 2014	1.09	[0.93; 1.28]	2.5%	3.1%
Wang, 2013	1.22	[1.11; 1.33]	2.7%	9.0%
Wang, 2010	0.69	[0.57; 0.83]	2.4%	3.1%
Wei, 2013	1.09	[0.89; 1.33]	2.3%	1.9%
Yan, 2015	0.65	[0.51; 0.82]	2.1%	1.9%
Zhan, 2011	0.76	[0.61; 0.93]	2.2%	2.2%
Zhang, 2016	0.93	[0.71; 1.21]	1.9%	1.2%
Zhang, 2013	0.84	[0.74; 0.96]	2.6%	5.9%
Zhou, 2014	0.69	[0.54; 0.88]	2.1%	1.8%
Zhu, 2012	0.81	[0.69; 0.96]	2.5%	3.5%
**Fixed effect model**	**0.95**	**[0.93; 0.98]**	**−−**	**100.0%**
**Random effects model**	**0.94**	**[0.86; 1.04]**	**100.0%**	**−−**

Heterogeneity: I^2 = 85%, τ^2 = 0.0648, p < 0.01
Test for effect in subgroup (fixed effect): z = −3.21 (p < 0.01)
Test for effect in subgroup (random effects): t_{48} = −1.26 (p = 0.21)

Study	OR	95%−CI	Weight (random)	Weight (fixed)
**Others**				
Afsharzadeh, 2017	0.86	[0.60; 1.24]	2.2%	0.9%
Alshatwi, 2012	1.23	[0.81; 1.88]	1.9%	0.5%
Bansal, 2014	0.83	[0.58; 1.20]	2.2%	0.9%
Bodal, 2017	1.53	[0.94; 2.50]	1.6%	0.4%
Catucci, 1, 2010	0.93	[0.82; 1.07]	3.7%	6.1%
Catucci, 2, 2010	0.99	[0.89; 1.11]	3.8%	8.3%
Dai, 2016	0.73	[0.62; 0.86]	3.5%	4.6%
Dou, 2010	1.05	[0.90; 1.22]	3.6%	4.4%
Doulah, 2018	0.86	[0.58; 1.28]	2.0%	0.7%
He, 2018	0.95	[0.80; 1.13]	3.5%	3.8%
He, 2015	1.06	[0.88; 1.28]	3.4%	3.0%
Hoffman, 2009	0.74	[0.61; 0.90]	3.3%	3.3%
Hong, 2011	0.88	[0.73; 1.07]	3.4%	3.1%
Hu, 2013	0.80	[0.69; 0.93]	3.6%	5.3%
Hu, 2009	0.85	[0.75; 0.96]	3.8%	7.9%
Jedlinski, 2011	0.94	[0.70; 1.27]	2.6%	1.2%
Linhares, 2012	1.32	[1.08; 1.61]	3.3%	2.3%
Ma, 2013	1.00	[0.75; 1.33]	2.7%	1.3%
Mashayekhi, 2018	1.02	[0.82; 1.27]	3.2%	2.2%
Minh, 2018	0.71	[0.50; 1.02]	2.2%	1.0%
Morales, 2016	0.95	[0.80; 1.13]	3.5%	3.7%
Omrani, 2014	0.60	[0.32; 1.12]	1.2%	0.4%
Parlayan, 2014	0.98	[0.75; 1.26]	2.9%	1.6%
Qi, 2015	0.65	[0.50; 0.84]	2.9%	2.0%
Sodhi, 2015	1.42	[1.10; 1.84]	2.9%	1.3%
Tian, 2009	0.89	[0.79; 1.01]	3.8%	7.7%
Toraih, 2016	1.42	[0.96; 2.11]	2.1%	0.6%
Vinci, 2011	1.37	[0.93; 2.02]	2.1%	0.6%
Yin, 2017	0.79	[0.69; 0.89]	3.8%	7.9%
Yin, 2016	0.93	[0.79; 1.09]	3.6%	4.3%
Yin, 2015	0.92	[0.72; 1.16]	3.1%	2.0%
Zhang, 2012	1.23	[0.92; 1.64]	2.7%	1.1%
Zhao, 2016	1.09	[0.76; 1.58]	2.2%	0.8%
Kim, 2010	0.84	[0.72; 0.98]	3.6%	4.9%
**Fixed effect model**	**0.92**	**[0.89; 0.95]**	**−−**	**100.0%**
**Random effects model**	**0.94**	**[0.88; 1.01]**	**100.0%**	**−−**

Heterogeneity: I^2 = 62%, τ^2 = 0.0167, p < 0.01
Test for effect in subgroup (fixed effect): z = −4.91 (p < 0.01)
Test for effect in subgroup (random effects): t_{48} = −1.87 (p = 0.07)

Study	OR	95%−CI	Weight (random)	Weight (fixed)
**HNC**				
Christensen, 2010	0.93	[0.78; 1.11]	12.1%	10.9%
Chu, 2012	1.14	[0.94; 1.37]	11.8%	8.7%
Li, 2014	1.13	[1.00; 1.27]	13.2%	20.0%
Liu, 2013	1.18	[0.85; 1.64]	8.5%	2.7%
Liu, 2010	1.04	[0.92; 1.17]	13.3%	22.7%
Miao, 2016	0.85	[0.74; 0.97]	13.0%	19.1%
Roy, 2014	1.20	[0.98; 1.47]	11.4%	7.2%
Sushma, 2015	0.41	[0.25; 0.69]	5.3%	2.0%
Zhang, 2017	1.13	[0.91; 1.39]	11.2%	6.8%
**Fixed effect model**	**1.03**	**[0.97; 1.09]**	**−−**	**100.0%**
**Random effects model**	**1.02**	**[0.85; 1.21]**	**100.0%**	**−−**

Heterogeneity: I^2 = 71%, τ^2 = 0.0197, p < 0.01
Test for effect in subgroup (fixed effect): z = 0.88 (p = 0.38)
Test for effect in subgroup (random effects): t_{48} = 0.22 (p = 0.83)

Study	OR	95%−CI	Weight (random)	Weight (fixed)
**UG**				
Damodaran, 2018	1.37	[0.92; 2.06]	6.0%	2.3%
Deng, 2015	1.00	[0.76; 1.32]	8.3%	5.8%
George, 2011	1.23	[0.91; 1.68]	7.6%	4.1%
Hashemi, 2016	1.19	[0.87; 1.63]	7.5%	4.1%
Horikawa, 2008	0.87	[0.69; 1.11]	9.0%	8.1%
Mittal, 2011	1.11	[0.84; 1.46]	8.2%	5.4%
Mulong Du, 2014	1.20	[1.05; 1.35]	11.2%	25.6%
Nikolic, 2015	0.89	[0.71; 1.11]	9.3%	9.1%
Parlayan, 2014	1.01	[0.74; 1.39]	7.4%	4.3%
Toraih−1, 2016	1.69	[1.10; 2.59]	5.6%	1.8%
Wang, 2016	0.70	[0.56; 0.89]	9.1%	9.5%
Yang, 2008	1.01	[0.87; 1.17]	10.8%	20.1%
**Fixed effect model**	**1.05**	**[0.98; 1.12]**	**−−**	**100.0%**
**Random effects model**	**1.05**	**[0.92; 1.20]**	**100.0%**	**−−**

Heterogeneity: I^2 = 61%, τ^2 = 0.0228, p < 0.01
Test for effect in subgroup (fixed effect): z = 1.41 (p = 0.16)
Test for effect in subgroup (random effects): t_{48} = 0.76 (p = 0.46)

Study	OR	95%−CI	Weight (random)	Weight (fixed)
**HM**				
Li, 2015	0.71	[0.57; 0.89]	18.6%	20.7%
Martin−Guerrero, 2015	1.50	[1.10; 2.05]	15.1%	7.4%
Parlayan, 2014	0.84	[0.59; 1.19]	13.8%	8.0%
Peckham−Gregory, 2016	0.86	[0.67; 1.11]	17.6%	15.7%
Rakmanee, 2017	0.47	[0.33; 0.68]	13.6%	10.5%
Tong, 2014	0.88	[0.75; 1.03]	21.3%	37.7%
**Fixed effect model**	**0.84**	**[0.76; 0.93]**	**−−**	**100.0%**
**Random effects model**	**0.83**	**[0.57; 1.21]**	**100.0%**	**−−**

Heterogeneity: I^2 = 81%, τ^2 = 0.0695, p < 0.01
Test for effect in subgroup (fixed effect): z = −3.40 (p < 0.01)
Test for effect in subgroup (random effects): t_{48} = −1.26 (p = 0.26)

Study	OR	95%−CI	Weight (random)	Weight (fixed)
**GyC**				
Liu, 2015	1.23	[0.88; 1.72]	14.2%	8.2%
Ni, 2016	0.92	[0.70; 1.20]	16.8%	15.0%
Song, 2016	0.74	[0.61; 0.89]	20.3%	35.5%
Srivastava, 2017	1.06	[0.78; 1.44]	15.3%	10.6%
Sun, 2016	0.79	[0.58; 1.07]	15.4%	12.6%
Zhou, 2011	0.94	[0.74; 1.20]	17.9%	18.1%
**Fixed effect model**	**0.88**	**[0.80; 0.98]**	**−−**	**100.0%**
**Random effects model**	**0.91**	**[0.75; 1.10]**	**100.0%**	**−−**

Heterogeneity: I^2 = 48%, τ^2 = 0.0165, p = 0.09
Test for effect in subgroup (fixed effect): z = −2.29 (p = 0.02)
Test for effect in subgroup (random effects): t_{48} = −1.25 (p = 0.27)

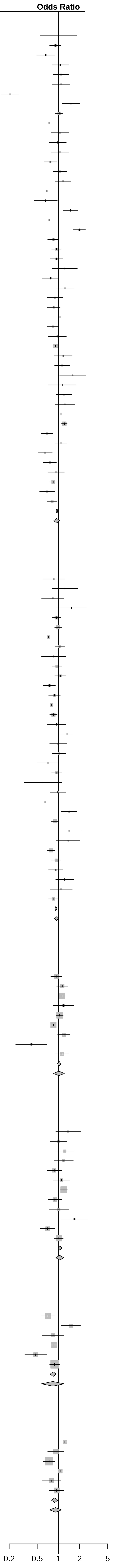

Supplement: Supplementary file 1 [file Data_Sheet_1.ZIP › Supplementary Figures/Suppl. Fig. S2.pdf]
