# Supplementary material for: Association of mir-196a-2 rs11614913 and mir-149 rs2292832 Polymorphisms With Risk of Cancer: An Updated Meta-Analysis
Source: Front Genet. 2019 Mar 15;10:186. doi: 10.3389/fgene.2019.00186 (PMC6429108; doi:10.3389/fgene.2019.00186)

Supplementary Figure S3. Forest plots for subgroup meta-analysis of miR-196a-2 rs11614913 according to cancer types. **A)** Homozygote comparison; **B)** Heterozygote comparison; **C)** Dominant comparison; **D)** Recessive comparison; **E)** Allele contrast. **Abbreviations:** BC: breast cancer; HCC: Hepatocellular cancer; GC: gastric cancer; CRC: colorectal cancer; LC: lung cancer; BIC: bladder cancer; PC: prostate cancer; OC: Oral cancer; OvC: ovarian cancer; ESCC: esophageal squamous cell carcinoma

A) Homozygote comparison

Study	OR	95%−CI	Weight (random)	Weight (fixed)
**HCC**				
Abdel−Hamid, 2018	0.57	[0.13; 2.57]	1.8%	0.6%
Akkiz, 2011	0.41	[0.22; 0.77]	5.2%	4.3%
Chu, 2014	1.13	[0.69; 1.85]	6.1%	4.1%
Han, 2013	1.07	[0.83; 1.37]	7.8%	16.6%
Hao, 2014	0.51	[0.29; 0.87]	5.8%	5.0%
Huang, 2017	0.99	[0.54; 1.82]	5.3%	2.9%
Kim, 2012	1.11	[0.60; 2.04]	5.3%	2.7%
Kou, 2014	0.53	[0.34; 0.85]	6.3%	6.8%
Li, 2016	0.41	[0.18; 0.93]	4.1%	2.4%
Li, 2015	2.29	[1.34; 3.89]	5.9%	2.5%
Li, 2010	0.57	[0.35; 0.92]	6.2%	6.0%
Qi, 2014	0.78	[0.48; 1.27]	6.2%	5.0%
Qi, 2010	1.10	[0.73; 1.65]	6.8%	6.1%
Toraih−2, 2016	0.56	[0.15; 2.09]	2.2%	0.9%
Yan, 2015	0.35	[0.20; 0.61]	5.7%	5.9%
Zhang, 2016	0.90	[0.50; 1.60]	5.5%	3.3%
Zhang, 2013	0.69	[0.53; 0.89]	7.8%	19.2%
Zhou, 2014	0.44	[0.26; 0.75]	5.9%	5.7%
**Fixed effect model**	**0.78**	**[0.70; 0.87]**	**−−**	**100.0%**
**Random effects model**	**0.74**	**[0.58; 0.94]**	**100.0%**	**−−**
Heterogeneity: $I^2 = 69\%$, $\tau^2 = 0.1368$, $p < 0.01$				
Test for effect in subgroup (fixed effect): $z = -4.37$ ($p < 0.01$)				
Test for effect in subgroup (random effects): $t_{17} = -2.65$ ($p = 0.02$)				
**BC**				
Afsharzadeh, 2017	0.82	[0.36; 1.89]	3.7%	1.4%
Alshatwi, 2012	0.66	[0.11; 3.79]	1.3%	0.4%
Bansal, 2014	0.71	[0.33; 1.55]	4.0%	1.7%
Bodal, 2017			0.0%	0.0%
Catucci, 1, 2010	0.86	[0.64; 1.16]	7.0%	10.9%
Catucci, 2, 2010	0.98	[0.77; 1.25]	7.3%	15.1%
Dai, 2016	0.54	[0.38; 0.75]	6.8%	10.7%
Doulah, 2018	0.82	[0.33; 2.04]	3.3%	1.1%
He, 2015	1.17	[0.80; 1.71]	6.5%	5.5%
Hoffman, 2009	0.47	[0.30; 0.73]	6.0%	6.4%
Hu, 2009	0.73	[0.57; 0.93]	7.3%	17.5%
Jedlinski, 2011	0.91	[0.50; 1.66]	5.0%	2.5%
Linhares, 2012	1.65	[1.13; 2.41]	6.5%	4.7%
Ma, 2013	1.02	[0.59; 1.77]	5.3%	2.8%
Mashayekhi, 2018	0.96	[0.59; 1.54]	5.8%	3.9%
Minh, 2018	0.61	[0.31; 1.19]	4.6%	2.4%
Morales, 2016	0.89	[0.62; 1.28]	6.6%	7.0%
Omrani, 2014			0.0%	0.0%
Qi, 2015	0.45	[0.24; 0.84]	4.9%	3.5%
Zhang, 2012	1.72	[0.78; 3.80]	3.9%	1.1%
Zhao, 2016	1.19	[0.58; 2.47]	4.2%	1.5%
**Fixed effect model**	**0.85**	**[0.77; 0.94]**	**−−**	**100.0%**
**Random effects model**	**0.85**	**[0.72; 1.02]**	**100.0%**	**−−**
Heterogeneity: $I^2 = 56\%$, $\tau^2 = 0.0640$, $p < 0.01$				
Test for effect in subgroup (fixed effect): $z = -3.27$ ($p < 0.01$)				
Test for effect in subgroup (random effects): $t_{17} = -1.92$ ($p = 0.07$)				
**GC**				
Ahn, 2013	0.81	[0.55; 1.18]	9.0%	9.3%
Dikeakos, 2014	0.07	[0.04; 0.12]	6.9%	15.0%
Gu, 2016	2.40	[1.31; 4.40]	6.9%	2.1%
Kupcinskas, 2014	0.84	[0.51; 1.38]	7.9%	5.4%
Li, 2016	0.37	[0.17; 0.81]	5.6%	3.4%
Okubo, 2010	0.88	[0.63; 1.22]	9.5%	11.9%
Parlayan, 2014	0.74	[0.45; 1.20]	8.0%	5.9%
Peng, 2010	0.63	[0.37; 1.08]	7.6%	5.3%
Poltronieri−Oliveira, 2017	2.19	[1.15; 4.17]	6.6%	1.9%
Pu, 2014	0.75	[0.42; 1.34]	7.2%	4.2%
Rogoveanu, 2017	0.92	[0.48; 1.73]	6.7%	3.1%
Su, 2016	1.28	[0.75; 2.20]	7.5%	3.6%
Wang, 2013	1.50	[1.24; 1.80]	10.6%	28.6%
**Fixed effect model**	**0.95**	**[0.85; 1.07]**	**−−**	**100.0%**
**Random effects model**	**0.80**	**[0.47; 1.37]**	**100.0%**	**−−**
Heterogeneity: $I^2 = 90\%$, $\tau^2 = 0.4751$, $p < 0.01$				
Test for effect in subgroup (fixed effect): $z = -0.82$ ($p = 0.41$)				
Test for effect in subgroup (random effects): $t_{17} = -0.90$ ($p = 0.39$)				
**CRC**				
Chen, 2012	1.14	[0.64; 2.02]	9.8%	6.7%
Dikaiakos, 2015	1.02	[0.54; 1.94]	9.1%	5.7%
Hezova, 2012	1.25	[0.66; 2.38]	9.0%	5.0%
Kupcinskas, 2014	1.26	[0.74; 2.14]	10.4%	7.2%
Lv, 2013	13.65	[6.76; 27.60]	8.3%	1.7%
Min, 2013	0.70	[0.49; 1.01]	12.6%	21.9%
Parlayan, 2014	1.09	[0.61; 1.96]	9.7%	6.6%
Vinci, 2013	1.46	[0.60; 3.53]	6.6%	2.5%
Zhan, 2011	0.57	[0.37; 0.88]	11.7%	16.8%
Zhu, 2012	0.65	[0.47; 0.91]	12.9%	26.0%
**Fixed effect model**	**1.05**	**[0.90; 1.22]**	**−−**	**100.0%**
**Random effects model**	**1.22**	**[0.65; 2.28]**	**100.0%**	**−−**
Heterogeneity: $I^2 = 88\%$, $\tau^2 = 0.4811$, $p < 0.01$				
Test for effect in subgroup (fixed effect): $z = 0.61$ ($p = 0.54$)				
Test for effect in subgroup (random effects): $t_{17} = 0.72$ ($p = 0.49$)				
**Others**				
Christensen, 2010	0.92	[0.63; 1.32]	5.4%	5.9%
Dou, 2010	1.17	[0.85; 1.61]	5.7%	7.0%
He, 2018	0.91	[0.65; 1.27]	5.6%	6.9%
Horikawa, 2008	0.73	[0.46; 1.18]	4.8%	3.9%
Hu, 2013	0.64	[0.48; 0.87]	5.8%	10.7%
Li, 2015	0.53	[0.33; 0.84]	4.9%	4.8%
Li, 2014	1.24	[0.97; 1.60]	6.0%	10.9%
Liu, 2015	1.56	[0.71; 3.41]	3.3%	1.0%
Liu, 2010	1.05	[0.82; 1.34]	6.0%	12.3%
Martin−Guerrero, 2015	2.32	[1.28; 4.18]	4.2%	1.3%
Miao, 2016	1.29	[0.73; 2.29]	4.3%	2.1%
Mulong Du, 2014	1.52	[1.17; 1.97]	6.0%	9.1%
Parlayan, 2014	0.70	[0.36; 1.36]	3.8%	2.0%
Pavlakis, 2013	1.12	[0.47; 2.66]	2.9%	1.0%
Peckham−Gregory, 2016	0.68	[0.38; 1.20]	4.3%	3.0%
Rakmanee, 2017	0.25	[0.12; 0.52]	3.5%	3.1%
Srivastava, 2017	1.20	[0.60; 2.42]	3.6%	1.4%
Srivastava, 2010	0.96	[0.47; 1.96]	3.6%	1.5%
Tong, 2014	0.84	[0.61; 1.17]	5.6%	7.6%
Toraih, 2016	2.10	[0.95; 4.60]	3.2%	0.9%
Toraih−1, 2016	2.25	[0.93; 5.47]	2.8%	0.6%
Zhou, 2011	0.88	[0.52; 1.47]	4.6%	3.0%
**Fixed effect model**	**1.00**	**[0.92; 1.10]**	**−−**	**100.0%**
**Random effects model**	**0.99**	**[0.81; 1.21]**	**100.0%**	**−−**
Heterogeneity: $I^2 = 68\%$, $\tau^2 = 0.0989$, $p < 0.01$				
Test for effect in subgroup (fixed effect): $z = 0.11$ ($p = 0.91$)				
Test for effect in subgroup (random effects): $t_{17} = -0.13$ ($p = 0.90$)				
**OC**				
Chu, 2012	1.57	[1.04; 2.37]	18.7%	14.5%
Liu, 2013	1.41	[0.76; 2.59]	14.8%	6.8%
Miao, 2016	0.63	[0.47; 0.85]	21.0%	43.4%
Roy, 2014	1.34	[0.84; 2.14]	17.6%	12.1%
Sushma, 2015	0.23	[0.09; 0.60]	9.4%	8.0%
Zhang, 2017	1.28	[0.84; 1.94]	18.5%	15.3%
**Fixed effect model**	**0.97**	**[0.82; 1.16]**	**−−**	**100.0%**
**Random effects model**	**0.98**	**[0.49; 1.95]**	**100.0%**	**−−**
Heterogeneity: $I^2 = 82\%$, $\tau^2 = 0.2376$, $p < 0.01$				
Test for effect in subgroup (fixed effect): $z = -0.30$ ($p = 0.77$)				
Test for effect in subgroup (random effects): $t_{17} = -0.07$ ($p = 0.95$)				
**PC**				
Damodaran, 2018	1.47	[0.65; 3.30]	19.2%	12.5%
George, 2011	0.58	[0.15; 2.19]	10.1%	8.2%
Hashemi, 2016	1.70	[0.76; 3.83]	19.2%	11.8%
Nikolic, 2015	0.79	[0.48; 1.29]	28.5%	45.5%
Parlayan, 2014	1.04	[0.53; 2.04]	23.0%	21.9%
**Fixed effect model**	**1.02**	**[0.74; 1.40]**	**−−**	**100.0%**
**Random effects model**	**1.02**	**[0.65; 1.62]**	**100.0%**	**−−**
Heterogeneity: $I^2 = 2\%$, $\tau^2 = 0.0027$, $p = 0.40$				
Test for effect in subgroup (fixed effect): $z = 0.12$ ($p = 0.90$)				
Test for effect in subgroup (random effects): $t_{17} = 0.15$ ($p = 0.89$)				
**BIC**				
Deng, 2015	0.93	[0.55; 1.60]	25.8%	16.0%
Mittal, 2011	0.51	[0.18; 1.48]	13.2%	6.0%
Wang, 2016	0.49	[0.31; 0.79]	27.7%	27.9%
Yang, 2008	1.02	[0.75; 1.37]	33.3%	50.1%
**Fixed effect model**	**0.83**	**[0.66; 1.03]**	**−−**	**100.0%**
**Random effects model**	**0.76**	**[0.41; 1.39]**	**100.0%**	**−−**
Heterogeneity: $I^2 = 59\%$, $\tau^2 = 0.0939$, $p = 0.06$				
Test for effect in subgroup (fixed effect): $z = -1.69$ ($p = 0.09$)				
Test for effect in subgroup (random effects): $t_{17} = -1.46$ ($p = 0.24$)				
**LC**				
Hong, 2011	0.80	[0.54; 1.18]	12.1%	9.3%
Parlayan, 2014	0.97	[0.56; 1.67]	10.2%	4.4%
Sodhi, 2015	3.43	[1.42; 8.27]	6.6%	0.9%
Tian, 2009	0.79	[0.62; 1.01]	13.9%	24.2%
Vinci, 2011	1.99	[0.78; 5.08]	6.2%	1.0%
Yin, 2017	0.60	[0.47; 0.78]	13.8%	25.0%
Yin, 2016	0.87	[0.63; 1.20]	13.0%	12.8%
Yin, 2015	0.87	[0.54; 1.41]	11.0%	5.8%
Kim, 2010	0.73	[0.54; 0.98]	13.3%	16.6%
**Fixed effect model**	**0.79**	**[0.70; 0.89]**	**−−**	**100.0%**
**Random effects model**	**0.86**	**[0.64; 1.17]**	**100.0%**	**−−**
Heterogeneity: $I^2 = 60\%$, $\tau^2 = 0.0547$, $p < 0.01$				
Test for effect in subgroup (fixed effect): $z = -3.82$ ($p < 0.01$)				
Test for effect in subgroup (random effects): $t_{17} = -1.11$ ($p = 0.30$)				
**OvC**				
Liu, 2015	3.01	[1.05; 8.67]	14.3%	3.3%
Ni, 2016	0.85	[0.48; 1.48]	27.2%	20.5%
Song, 2016	0.56	[0.38; 0.81]	33.6%	57.2%
Sun, 2016	0.59	[0.32; 1.11]	24.9%	19.1%
**Fixed effect model**	**0.70**	**[0.54; 0.91]**	**−−**	**100.0%**
**Random effects model**	**0.82**	**[0.28; 2.37]**	**100.0%**	**−−**
Heterogeneity: $I^2 = 68\%$, $\tau^2 = 0.1913$, $p = 0.02$				
Test for effect in subgroup (fixed effect): $z = -2.62$ ($p < 0.01$)				
Test for effect in subgroup (random effects): $t_{17} = -0.59$ ($p = 0.59$)				
**ESCC**				
Qu, 2014	0.62	[0.40; 0.95]	16.3%	11.2%
Shen, 2016	0.80	[0.66; 0.98]	20.2%	47.5%
Umar, 2013	1.61	[0.82; 3.18]	12.0%	2.8%
Wang, 2014	1.19	[0.86; 1.65]	18.2%	14.2%
Wang, 2010	0.37	[0.25; 0.57]	16.7%	16.0%
Wei, 2013	1.26	[0.83; 1.90]	16.6%	8.4%
**Fixed effect model**	**0.83**	**[0.73; 0.95]**	**−−**	**100.0%**
**Random effects model**	**0.85**	**[0.49; 1.47]**	**100.0%**	**−−**
Heterogeneity: $I^2 = 82\%$, $\tau^2 = 0.1567$, $p < 0.01$				
Test for effect in subgroup (fixed effect): $z = -2.74$ ($p < 0.01$)				
Test for effect in subgroup (random effects): $t_{17} = -0.75$ ($p = 0.49$)				

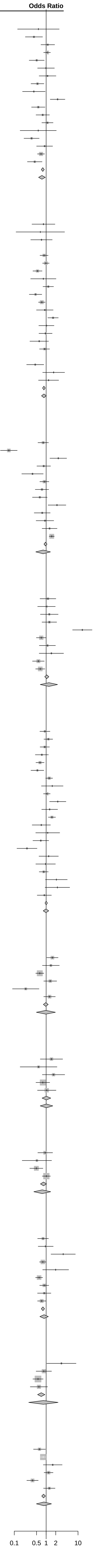

B) Heterozygote comparison

Study	OR	95%−CI	Weight (random)	Weight (fixed)
**HCC**				
Abdel−Hamid, 2018	1.49	[0.65; 3.39]	2.8%	1.0%
Akkiz, 2011	0.74	[0.47; 1.17]	5.6%	4.8%
Chu, 2014	0.83	[0.52; 1.32]	5.4%	4.2%
Han, 2013	1.11	[0.88; 1.39]	8.1%	15.5%
Hao, 2014	0.69	[0.46; 1.02]	6.1%	6.3%
Huang, 2017	1.07	[0.59; 1.93]	4.3%	2.3%
Kim, 2012	1.04	[0.61; 1.76]	4.9%	2.9%
Kou, 2014	0.73	[0.52; 1.03]	6.8%	8.4%
Li, 2016	0.89	[0.44; 1.80]	3.5%	1.8%
Li, 2015	1.43	[0.99; 2.08]	6.4%	5.0%
Li, 2010	0.79	[0.50; 1.24]	5.6%	4.7%
Qi, 2014	1.54	[1.01; 2.34]	5.9%	3.9%
Qi, 2010	1.02	[0.71; 1.46]	6.6%	6.4%
Toraih−2, 2016	1.93	[1.03; 3.62]	4.0%	1.5%
Yan, 2015	0.52	[0.31; 0.88]	4.9%	4.3%
Zhang, 2016	1.03	[0.59; 1.82]	4.5%	2.6%
Zhang, 2013	0.75	[0.59; 0.95]	8.0%	17.2%
Zhou, 2014	0.62	[0.42; 0.91]	6.3%	7.1%
**Fixed effect model**	**0.92**	**[0.84; 1.01]**	**−−**	**100.0%**
**Random effects model**	**0.93**	**[0.79; 1.09]**	**100.0%**	**−−**
Heterogeneity: $I^2 = 55\%$, $\tau^2 = 0.0530$, $p < 0.01$				
Test for effect in subgroup (fixed effect): $z = -1.76$ ($p = 0.08$)				
Test for effect in subgroup (random effects): $t_{17} = -0.95$ ($p = 0.36$)				
**BC**				
Afsharzadeh, 2017	0.62	[0.35; 1.11]	3.7%	2.0%
Alshatwi, 2012	1.66	[0.93; 2.94]	3.7%	1.2%
Bansal, 2014	0.87	[0.52; 1.45]	4.2%	2.1%
Bodal, 2017	1.79	[1.01; 3.18]	3.7%	1.2%
Catucci, 1, 2010	0.96	[0.79; 1.16]	7.0%	14.1%
Catucci, 2, 2010	0.99	[0.84; 1.18]	7.2%	18.2%
Dai, 2016	0.73	[0.56; 0.96]	6.3%	8.3%
Doulah, 2018	0.62	[0.33; 1.18]	3.3%	1.6%
He, 2015	1.20	[0.85; 1.70]	5.6%	3.8%
Hoffman, 2009	0.84	[0.63; 1.11]	6.2%	7.1%
Hu, 2009	0.85	[0.68; 1.06]	6.8%	11.4%
Jedlinski, 2011	0.89	[0.56; 1.42]	4.5%	2.5%
Linhares, 2012	1.45	[1.03; 2.04]	5.6%	3.7%
Ma, 2013	1.30	[0.78; 2.15]	4.2%	1.8%
Mashayekhi, 2018	1.12	[0.82; 1.54]	5.9%	4.9%
Minh, 2018	0.35	[0.19; 0.65]	3.4%	2.3%
Morales, 2016	0.97	[0.76; 1.24]	6.5%	8.4%
Omrani, 2014	0.59	[0.31; 1.11]	3.3%	1.7%
Qi, 2015	0.68	[0.36; 1.29]	3.2%	1.6%
Zhang, 2012	1.48	[0.66; 3.33]	2.4%	0.7%
Zhao, 2016	0.74	[0.39; 1.39]	3.3%	1.5%
**Fixed effect model**	**0.95**	**[0.88; 1.02]**	**−−**	**100.0%**
**Random effects model**	**0.94**	**[0.81; 1.09]**	**100.0%**	**−−**
Heterogeneity: $I^2 = 53\%$, $\tau^2 = 0.0352$, $p < 0.01$				
Test for effect in subgroup (fixed effect): $z = -1.50$ ($p = 0.13$)				
Test for effect in subgroup (random effects): $t_{17} = -0.89$ ($p = 0.39$)				
**GC**				
Ahn, 2013	0.91	[0.65; 1.27]	8.6%	7.9%
Dikeakos, 2014	0.16	[0.10; 0.24]	7.3%	11.6%
Gu, 2016	1.43	[0.87; 2.35]	6.5%	3.0%
Kupcinskas, 2014	1.40	[1.02; 1.92]	9.0%	7.5%
Li, 2016	0.48	[0.22; 1.05]	3.9%	2.2%
Okubo, 2010	0.95	[0.70; 1.28]	9.1%	9.7%
Parlayan, 2014	0.65	[0.42; 1.01]	7.3%	5.4%
Peng, 2010	0.65	[0.42; 1.01]	7.2%	5.5%
Poltronieri−Oliveira, 2017	0.78	[0.50; 1.21]	7.2%	5.0%
Pu, 2014	0.76	[0.49; 1.17]	7.3%	5.1%
Rogoveanu, 2017	0.98	[0.63; 1.50]	7.3%	4.7%
Su, 2016	1.16	[0.81; 1.67]	8.3%	6.1%
Wang, 2013	1.37	[1.16; 1.62]	11.0%	26.2%
**Fixed effect model**	**0.96**	**[0.88; 1.06]**	**−−**	**100.0%**
**Random effects model**	**0.81**	**[0.57; 1.16]**	**100.0%**	**−−**
Heterogeneity: $I^2 = 89\%$, $\tau^2 = 0.2600$, $p < 0.01$				
Test for effect in subgroup (fixed effect): $z = -0.75$ ($p = 0.45$)				
Test for effect in subgroup (random effects): $t_{17} = -1.28$ ($p = 0.22$)				
**CRC**				
Chen, 2012	1.08	[0.65; 1.80]	8.8%	6.1%
Dikaiakos, 2015	0.80	[0.43; 1.51]	7.1%	4.5%
Hezova, 2012	0.92	[0.61; 1.39]	10.6%	10.0%
Kupcinskas, 2014	1.26	[0.87; 1.82]	11.5%	10.9%
Lv, 2013	7.34	[3.76; 14.34]	6.6%	2.1%
Min, 2013	0.66	[0.48; 0.91]	12.4%	19.4%
Parlayan, 2014	1.03	[0.60; 1.74]	8.5%	5.8%
Vinci, 2013	1.37	[0.88; 2.14]	10.0%	7.1%
Zhan, 2011	0.80	[0.55; 1.15]	11.5%	13.5%
Zhu, 2012	0.89	[0.66; 1.19]	13.0%	20.6%
**Fixed effect model**	**1.06**	**[0.94; 1.20]**	**−−**	**100.0%**
**Random effects model**	**1.12**	**[0.72; 1.75]**	**100.0%**	**−−**
Heterogeneity: $I^2 = 81\%$, $\tau^2 = 0.1937$, $p < 0.01$				
Test for effect in subgroup (fixed effect): $z = 0.92$ ($p = 0.36$)				
Test for effect in subgroup (random effects): $t_{17} = 0.57$ ($p = 0.58$)				
**Others**				
Christensen, 2010	0.83	[0.63; 1.09]	5.9%	8.5%
Dou, 2010	1.45	[1.08; 1.94]	5.7%	5.5%
He, 2018	0.94	[0.69; 1.27]	5.6%	6.3%
Horikawa, 2008	1.04	[0.71; 1.50]	5.0%	4.0%
Hu, 2013	0.68	[0.52; 0.90]	5.9%	9.4%
Li, 2015	0.75	[0.47; 1.19]	4.3%	3.1%
Li, 2014	0.98	[0.79; 1.23]	6.3%	11.0%
Liu, 2015	2.12	[1.05; 4.29]	2.7%	0.8%
Liu, 2010	1.13	[0.94; 1.37]	6.6%	15.1%
Martin−Guerrero, 2015	0.98	[0.59; 1.64]	3.9%	2.2%
Miao, 2016	1.20	[0.70; 2.08]	3.6%	1.8%
Mulong Du, 2014	1.46	[1.15; 1.87]	6.1%	7.9%
Parlayan, 2014	0.59	[0.32; 1.07]	3.3%	1.9%
Pavlakis, 2013	0.66	[0.27; 1.60]	2.0%	0.9%
Peckham−Gregory, 2016	0.93	[0.65; 1.34]	5.1%	4.4%
Rakmanee, 2017	0.56	[0.33; 0.97]	3.6%	2.5%
Srivastava, 2017	1.21	[0.61; 2.38]	2.8%	1.1%
Srivastava, 2010	1.45	[0.98; 2.14]	4.8%	3.1%
Tong, 2014	1.26	[0.93; 1.70]	5.6%	5.4%
Toraih, 2016	1.74	[1.04; 2.92]	3.8%	1.6%
Toraih−1, 2016	2.03	[1.07; 3.86]	3.0%	0.9%
Zhou, 2011	0.92	[0.58; 1.44]	4.3%	2.9%
**Fixed effect model**	**1.06**	**[0.99; 1.14]**	**−−**	**100.0%**
**Random effects model**	**1.06**	**[0.91; 1.22]**	**100.0%**	**−−**
Heterogeneity: $I^2 = 61\%$, $\tau^2 = 0.0517$, $p < 0.01$				
Test for effect in subgroup (fixed effect): $z = 1.59$ ($p = 0.11$)				
Test for effect in subgroup (random effects): $t_{17} = 0.79$ ($p = 0.44$)				
**OC**				
Chu, 2012	2.05	[1.40; 3.00]	18.8%	11.6%
Liu, 2013	1.66	[0.93; 2.97]	12.9%	5.2%
Miao, 2016	0.79	[0.60; 1.02]	22.7%	37.6%
Roy, 2014	1.24	[0.94; 1.63]	22.2%	27.7%
Sushma, 2015	0.18	[0.05; 0.61]	4.7%	3.8%
Zhang, 2017	1.35	[0.92; 1.98]	18.7%	14.1%
**Fixed effect model**	**1.16**	**[1.00; 1.35]**	**−−**	**100.0%**
**Random effects model**	**1.15**	**[0.57; 2.29]**	**100.0%**	**−−**
Heterogeneity: $I^2 = 82\%$, $\tau^2 = 0.1779$, $p < 0.01$				
Test for effect in subgroup (fixed effect): $z = 1.95$ ($p = 0.05$)				
Test for effect in subgroup (random effects): $t_{17} = 0.51$ ($p = 0.63$)				
**PC**				
Damodaran, 2018	2.08	[1.12; 3.87]	15.2%	7.7%
George, 2011	1.71	[1.12; 2.60]	21.9%	18.4%
Hashemi, 2016	1.14	[0.73; 1.77]	21.2%	20.4%
Nikolic, 2015	0.88	[0.64; 1.23]	25.8%	42.1%
Parlayan, 2014	1.13	[0.62; 2.05]	15.9%	11.4%
**Fixed effect model**	**1.21**	**[0.99; 1.47]**	**−−**	**100.0%**
**Random effects model**	**1.27**	**[0.83; 1.95]**	**100.0%**	**−−**
Heterogeneity: $I^2 = 56\%$, $\tau^2 = 0.0699$, $p = 0.06$				
Test for effect in subgroup (fixed effect): $z = 1.87$ ($p = 0.06$)				
Test for effect in subgroup (random effects): $t_{17} = 1.57$ ($p = 0.19$)				
**BIC**				
Deng, 2015	0.54	[0.33; 0.89]	20.2%	15.1%
Mittal, 2011	1.48	[1.01; 2.17]	24.7%	16.0%
Wang, 2016	1.13	[0.75; 1.71]	23.6%	15.8%
Yang, 2008	1.03	[0.82; 1.29]	31.5%	53.1%
**Fixed effect model**	**1.04**	**[0.88; 1.23]**	**−−**	**100.0%**
**Random effects model**	**1.01**	**[0.54; 1.90]**	**100.0%**	**−−**
Heterogeneity: $I^2 = 70\%$, $\tau^2 = 0.0800$, $p = 0.02$				
Test for effect in subgroup (fixed effect): $z = 0.49$ ($p = 0.63$)				
Test for effect in subgroup (random effects): $t_{17} = 0.05$ ($p = 0.96$)				
**LC**				
Hong, 2011	1.26	[0.89; 1.79]	11.2%	7.7%
Parlayan, 2014	1.12	[0.69; 1.80]	8.8%	4.4%
Sodhi, 2015	1.59	[1.09; 2.32]	10.6%	5.8%
Tian, 2009	0.81	[0.65; 1.02]	13.6%	23.9%
Vinci, 2011	1.47	[0.84; 2.56]	7.6%	2.8%
Yin, 2017	0.98	[0.79; 1.22]	13.6%	22.3%
Yin, 2016	1.04	[0.78; 1.39]	12.3%	12.1%
Yin, 2015	1.18	[0.77; 1.83]	9.5%	5.0%
Kim, 2010	0.84	[0.65; 1.10]	12.7%	16.1%
**Fixed effect model**	**1.01**	**[0.92; 1.12]**	**−−**	**100.0%**
**Random effects model**	**1.06**	**[0.89; 1.26]**	**100.0%**	**−−**
Heterogeneity: $I^2 = 47\%$, $\tau^2 = 0.0227$, $p = 0.06$				
Test for effect in subgroup (fixed effect): $z = 0.26$ ($p = 0.80$)				
Test for effect in subgroup (random effects): $t_{17} = 0.75$ ($p = 0.48$)				
**OvC**				
Liu, 2015	3.68	[1.38; 9.83]	11.8%	3.4%
Ni, 2016	0.96	[0.58; 1.58]	27.6%	22.8%
Song, 2016	0.86	[0.62; 1.21]	37.0%	54.0%
Sun, 2016	0.67	[0.37; 1.19]	23.6%	19.8%
**Fixed effect model**	**0.94**	**[0.74; 1.20]**	**−−**	**100.0%**
**Random effects model**	**1.03**	**[0.38; 2.79]**	**100.0%**	**−−**
Heterogeneity: $I^2 = 67\%$, $\tau^2 = 0.1447$, $p = 0.03$				
Test for effect in subgroup (fixed effect): $z = -0.48$ ($p = 0.63$)				
Test for effect in subgroup (random effects): $t_{17} = 0.09$ ($p = 0.93$)				
**ESCC**				
Qu, 2014	1.04	[0.76; 1.41]	16.4%	12.5%
Shen, 2016	0.83	[0.69; 0.99]	19.7%	41.9%
Umar, 2013	1.16	[0.83; 1.62]	15.7%	10.1%
Wang, 2014	1.17	[0.88; 1.55]	17.0%	13.8%
Wang, 2010	0.91	[0.68; 1.22]	16.8%	14.9%
Wei, 2013	1.54	[1.05; 2.26]	14.5%	6.8%
**Fixed effect model**	**0.99**	**[0.89; 1.11]**	**−−**	**100.0%**
**Random effects model**	**1.05**	**[0.84; 1.32]**	**100.0%**	**−−**
Heterogeneity: $I^2 = 57\%$, $\tau^2 = 0.0276$, $p = 0.04$				
Test for effect in subgroup (fixed effect): $z = -0.10$ ($p = 0.92$)				
Test for effect in subgroup (random effects): $t_{17} = 0.55$ ($p = 0.61$)				

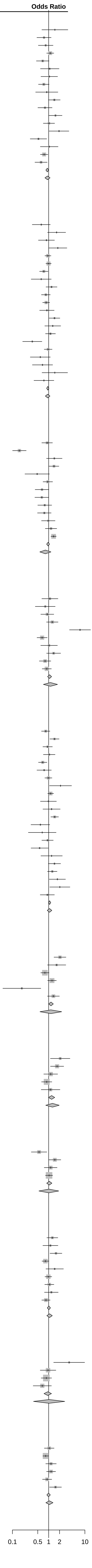

C) Dominant comparison

Study	OR	95%−CI	Weight (random)	Weight (fixed)
**HCC**				
Abdel−Hamid, 2018	1.27	[0.58; 2.81]	3.1%	1.1%
Akkiz, 2011	0.64	[0.42; 0.98]	5.7%	5.1%
Chu, 2014	0.94	[0.61; 1.45]	5.6%	4.0%
Han, 2013	1.09	[0.88; 1.35]	7.6%	15.5%
Hao, 2014	0.64	[0.43; 0.94]	6.0%	6.2%
Huang, 2017	1.03	[0.59; 1.81]	4.5%	2.3%
Kim, 2012	1.06	[0.64; 1.75]	5.0%	2.8%
Kou, 2014	0.68	[0.49; 0.95]	6.6%	8.2%
Li, 2016	0.70	[0.35; 1.37]	3.8%	2.0%
Li, 2015	1.60	[1.12; 2.28]	6.3%	4.6%
Li, 2010	0.69	[0.45; 1.06]	5.7%	5.1%
Qi, 2014	1.27	[0.84; 1.90]	5.9%	4.0%
Qi, 2010	1.05	[0.75; 1.47]	6.5%	6.3%
Toraih−2, 2016	1.60	[0.87; 2.93]	4.2%	1.6%
Yan, 2015	0.44	[0.27; 0.74]	5.0%	4.4%
Zhang, 2016	0.97	[0.57; 1.65]	4.8%	2.6%
Zhang, 2013	0.73	[0.58; 0.91]	7.5%	17.2%
Zhou, 2014	0.57	[0.39; 0.83]	6.2%	7.0%
**Fixed effect model**	**0.88**	**[0.80; 0.96]**	**−−**	**100.0%**
**Random effects model**	**0.88**	**[0.74; 1.04]**	**100.0%**	**−−**
Heterogeneity: $I^2 = 65\%$, $\tau^2 = 0.0713$, $p < 0.01$				
Test for effect in subgroup (fixed effect): $z = -2.88$ ($p < 0.01$)				
Test for effect in subgroup (random effects): $t_{17} = -1.59$ ($p = 0.13$)				
**BC**				
Afsharzadeh, 2017	0.66	[0.38; 1.15]	3.8%	1.8%
Alshatwi, 2012	1.58	[0.90; 2.79]	3.8%	1.1%
Bansal, 2014	0.83	[0.52; 1.33]	4.4%	2.2%
Bodal, 2017	1.79	[1.01; 3.18]	3.7%	1.0%
Catucci, 1, 2010	0.93	[0.78; 1.12]	6.6%	14.1%
Catucci, 2, 2010	0.99	[0.85; 1.16]	6.7%	17.9%
Dai, 2016	0.67	[0.52; 0.86]	6.1%	8.7%
Doulah, 2018	0.66	[0.35; 1.22]	3.5%	1.5%
He, 2015	1.19	[0.85; 1.65]	5.5%	3.8%
Hoffman, 2009	0.75	[0.57; 0.98]	6.0%	7.2%
Hu, 2009	0.80	[0.65; 0.99]	6.4%	11.7%
Jedlinski, 2011	0.90	[0.58; 1.39]	4.7%	2.5%
Linhares, 2012	1.52	[1.11; 2.08]	5.6%	3.7%
Ma, 2013	1.18	[0.74; 1.88]	4.4%	1.9%
Mashayekhi, 2018	1.09	[0.80; 1.46]	5.7%	4.8%
Minh, 2018	0.44	[0.25; 0.76]	3.9%	2.3%
Morales, 2016	0.95	[0.75; 1.20]	6.2%	8.4%
Omrani, 2014	0.59	[0.31; 1.11]	3.3%	1.5%
Qi, 2015	0.53	[0.29; 0.96]	3.5%	1.8%
Zhang, 2012	1.62	[0.74; 3.54]	2.6%	0.6%
Zhao, 2016	0.87	[0.48; 1.58]	3.6%	1.4%
**Fixed effect model**	**0.92**	**[0.86; 0.99]**	**−−**	**100.0%**
**Random effects model**	**0.92**	**[0.79; 1.07]**	**100.0%**	**−−**
Heterogeneity: $I^2 = 61\%$, $\tau^2 = 0.0425$, $p < 0.01$				
Test for effect in subgroup (fixed effect): $z = -2.36$ ($p = 0.02$)				
Test for effect in subgroup (random effects): $t_{17} = -1.20$ ($p = 0.24$)				
**GC**				
Ahn, 2013	0.87	[0.63; 1.20]	8.5%	8.0%
Dikeakos, 2014	0.12	[0.08; 0.18]	7.6%	12.8%
Gu, 2016	1.67	[1.04; 2.67]	6.7%	2.7%
Kupcinskas, 2014	1.27	[0.94; 1.70]	8.8%	7.8%
Li, 2016	0.42	[0.20; 0.89]	4.3%	2.3%
Okubo, 2010	0.92	[0.69; 1.23]	8.9%	9.7%
Parlayan, 2014	0.68	[0.46; 1.03]	7.5%	5.4%
Peng, 2010	0.64	[0.42; 0.97]	7.4%	5.6%
Poltronieri−Oliveira, 2017	0.99	[0.66; 1.49]	7.4%	4.6%
Pu, 2014	0.76	[0.50; 1.16]	7.3%	4.8%
Rogoveanu, 2017	0.96	[0.64; 1.44]	7.5%	4.8%
Su, 2016	1.19	[0.84; 1.68]	8.1%	5.8%
Wang, 2013	1.41	[1.21; 1.66]	10.2%	25.8%
**Fixed effect model**	**0.96**	**[0.88; 1.05]**	**−−**	**100.0%**
**Random effects model**	**0.80**	**[0.53; 1.20]**	**100.0%**	**−−**
Heterogeneity: $I^2 = 92\%$, $\tau^2 = 0.3476$, $p < 0.01$				
Test for effect in subgroup (fixed effect): $z = -0.86$ ($p = 0.39$)				
Test for effect in subgroup (random effects): $t_{17} = -1.19$ ($p = 0.26$)				
**CRC**				
Chen, 2012	1.10	[0.68; 1.79]	9.1%	6.0%
Dikaiakos, 2015	0.90	[0.49; 1.64]	7.5%	4.2%
Hezova, 2012	0.98	[0.66; 1.45]	10.6%	9.6%
Kupcinskas, 2014	1.26	[0.89; 1.78]	11.4%	11.1%
Lv, 2013	8.70	[4.48; 16.90]	6.8%	1.8%
Min, 2013	0.68	[0.50; 0.91]	12.1%	19.4%
Parlayan, 2014	1.05	[0.63; 1.74]	8.9%	5.7%
Vinci, 2013	1.38	[0.90; 2.13]	9.9%	6.6%
Zhan, 2011	0.71	[0.50; 1.01]	11.3%	14.0%
Zhu, 2012	0.80	[0.61; 1.06]	12.5%	21.5%
**Fixed effect model**	**1.05**	**[0.93; 1.19]**	**−−**	**100.0%**
**Random effects model**	**1.14**	**[0.70; 1.85]**	**100.0%**	**−−**
Heterogeneity: $I^2 = 85\%$, $\tau^2 = 0.2256$, $p < 0.01$				
Test for effect in subgroup (fixed effect): $z = 0.83$ ($p = 0.41$)				
Test for effect in subgroup (random effects): $t_{17} = 0.61$ ($p = 0.56$)				
**Others**				
Christensen, 2010	0.85	[0.66; 1.10]	5.6%	8.2%
Dou, 2010	1.34	[1.01; 1.76]	5.5%	5.6%
He, 2018	0.93	[0.70; 1.23]	5.4%	6.3%
Horikawa, 2008	0.93	[0.66; 1.32]	5.0%	4.3%
Hu, 2013	0.67	[0.52; 0.86]	5.7%	9.5%
Li, 2015	0.64	[0.41; 0.98]	4.4%	3.4%
Li, 2014	1.07	[0.87; 1.33]	5.9%	10.4%
Liu, 2015	1.92	[0.98; 3.75]	2.9%	0.8%
Liu, 2010	1.11	[0.93; 1.33]	6.1%	15.0%
Martin−Guerrero, 2015	1.30	[0.82; 2.06]	4.2%	2.1%
Miao, 2016	1.24	[0.74; 2.08]	3.8%	1.7%
Mulong Du, 2014	1.49	[1.18; 1.87]	5.8%	7.7%
Parlayan, 2014	0.63	[0.36; 1.09]	3.6%	1.9%
Pavlakis, 2013	0.88	[0.38; 1.99]	2.3%	0.8%
Peckham−Gregory, 2016	0.87	[0.62; 1.24]	5.0%	4.3%
Rakmanee, 2017	0.44	[0.26; 0.72]	3.9%	2.8%
Srivastava, 2017	1.20	[0.63; 2.31]	3.0%	1.1%
Srivastava, 2010	1.35	[0.93; 1.95]	4.8%	3.1%
Tong, 2014	1.08	[0.81; 1.43]	5.4%	5.8%
Toraih, 2016	1.82	[1.12; 2.94]	4.0%	1.6%
Toraih−1, 2016	2.09	[1.14; 3.81]	3.3%	1.0%
Zhou, 2011	0.90	[0.59; 1.39]	4.4%	2.8%
**Fixed effect model**	**1.04**	**[0.97; 1.12]**	**−−**	**100.0%**
**Random effects model**	**1.04**	**[0.89; 1.21]**	**100.0%**	**−−**
Heterogeneity: $I^2 = 68\%$, $\tau^2 = 0.0621$, $p < 0.01$				
Test for effect in subgroup (fixed effect): $z = 1.18$ ($p = 0.24$)				
Test for effect in subgroup (random effects): $t_{17} = 0.48$ ($p = 0.64$)				
**OC**				
Chu, 2012	1.86	[1.30; 2.68]	18.2%	11.4%
Liu, 2013	1.54	[0.91; 2.63]	14.0%	5.5%
Miao, 2016	0.73	[0.57; 0.93]	21.3%	37.1%
Roy, 2014	1.26	[0.97; 1.63]	21.0%	26.5%
Sushma, 2015	0.22	[0.09; 0.57]	7.1%	5.5%
Zhang, 2017	1.32	[0.93; 1.89]	18.4%	13.9%
**Fixed effect model**	**1.10**	**[0.95; 1.26]**	**−−**	**100.0%**
**Random effects model**	**1.07**	**[0.54; 2.12]**	**100.0%**	**−−**
Heterogeneity: $I^2 = 85\%$, $\tau^2 = 0.1900$, $p < 0.01$				
Test for effect in subgroup (fixed effect): $z = 1.29$ ($p = 0.20$)				
Test for effect in subgroup (random effects): $t_{17} = 0.25$ ($p = 0.81$)				
**PC**				
Damodaran, 2018	1.88	[1.06; 3.35]	16.4%	8.5%
George, 2011	1.62	[1.07; 2.45]	21.3%	17.6%
Hashemi, 2016	1.20	[0.78; 1.85]	20.9%	19.3%
Nikolic, 2015	0.86	[0.63; 1.18]	24.9%	43.0%
Parlayan, 2014	1.10	[0.62; 1.94]	16.6%	11.6%
**Fixed effect model**	**1.18**	**[0.97; 1.42]**	**−−**	**100.0%**
**Random effects model**	**1.24**	**[0.84; 1.83]**	**100.0%**	**−−**
Heterogeneity: $I^2 = 54\%$, $\tau^2 = 0.0586$, $p = 0.07$				
Test for effect in subgroup (fixed effect): $z = 1.68$ ($p = 0.09$)				
Test for effect in subgroup (random effects): $t_{17} = 1.52$ ($p = 0.20$)				
**BIC**				
Deng, 2015	0.67	[0.42; 1.05]	21.4%	14.0%
Mittal, 2011	1.38	[0.95; 2.01]	24.4%	14.9%
Wang, 2016	0.86	[0.58; 1.26]	24.1%	18.1%
Yang, 2008	1.02	[0.83; 1.27]	30.2%	53.0%
**Fixed effect model**	**1.00**	**[0.85; 1.17]**	**−−**	**100.0%**
**Random effects model**	**0.97**	**[0.62; 1.53]**	**100.0%**	**−−**
Heterogeneity: $I^2 = 54\%$, $\tau^2 = 0.0358$, $p = 0.09$				
Test for effect in subgroup (fixed effect): $z = -0.04$ ($p = 0.97$)				
Test for effect in subgroup (random effects): $t_{17} = -0.19$ ($p = 0.86$)				
**LC**				
Hong, 2011	1.08	[0.77; 1.50]	11.3%	8.0%
Parlayan, 2014	1.07	[0.67; 1.68]	9.2%	4.2%
Sodhi, 2015	1.69	[1.16; 2.45]	10.6%	5.0%
Tian, 2009	0.81	[0.65; 0.99]	13.1%	23.5%
Vinci, 2011	1.54	[0.90; 2.64]	8.1%	2.5%
Yin, 2017	0.84	[0.69; 1.04]	13.1%	23.1%
Yin, 2016	0.98	[0.74; 1.29]	12.1%	12.1%
Yin, 2015	1.06	[0.70; 1.61]	9.9%	5.1%
Kim, 2010	0.80	[0.62; 1.02]	12.5%	16.5%
**Fixed effect model**	**0.94**	**[0.86; 1.04]**	**−−**	**100.0%**
**Random effects model**	**1.00**	**[0.82; 1.22]**	**100.0%**	**−−**
Heterogeneity: $I^2 = 58\%$, $\tau^2 = 0.0315$, $p = 0.02$				
Test for effect in subgroup (fixed effect): $z = -1.21$ ($p = 0.23$)				
Test for effect in subgroup (random effects): $t_{17} = 0.02$ ($p = 0.99$)				
**OvC**				
Liu, 2015	3.44	[1.32; 8.93]	12.6%	3.2%
Ni, 2016	0.92	[0.57; 1.47]	27.7%	21.7%
Song, 2016	0.74	[0.54; 1.01]	35.3%	56.1%
Sun, 2016	0.64	[0.37; 1.10]	24.4%	19.0%
**Fixed effect model**	**0.85**	**[0.67; 1.06]**	**−−**	**100.0%**
**Random effects model**	**0.95**	**[0.34; 2.64]**	**100.0%**	**−−**
Heterogeneity: $I^2 = 70\%$, $\tau^2 = 0.1555$, $p = 0.02$				
Test for effect in subgroup (fixed effect): $z = -1.46$ ($p = 0.14$)				
Test for effect in subgroup (random effects): $t_{17} = -0.16$ ($p = 0.88$)				
**ESCC**				
Qu, 2014	0.92	[0.68; 1.23]	16.4%	12.8%
Shen, 2016	0.82	[0.69; 0.97]	19.0%	41.3%
Umar, 2013	1.21	[0.88; 1.67]	15.9%	9.4%
Wang, 2014	1.18	[0.90; 1.54]	17.0%	13.6%
Wang, 2010	0.74	[0.56; 0.98]	16.8%	15.8%
Wei, 2013	1.43	[1.00; 2.05]	15.0%	7.0%
**Fixed effect model**	**0.95**	**[0.85; 1.05]**	**−−**	**100.0%**
**Random effects model**	**1.00**	**[0.77; 1.30]**	**100.0%**	**−−**
Heterogeneity: $I^2 = 68\%$, $\tau^2 = 0.0393$, $p < 0.01$				
Test for effect in subgroup (fixed effect): $z = -1.00$ ($p = 0.32$)				
Test for effect in subgroup (random effects): $t_{17} = -0.00$ ($p = 1.00$)				

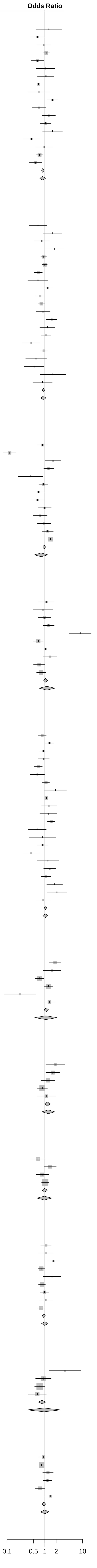

D) Recessive comparison

Study	OR	95%−CI	Weight (random)	Weight (fixed)
**HCC**				
Abdel−Hamid, 2018	0.47	[0.11; 1.99]	1.0%	0.5%
Akkiz, 2011	0.49	[0.28; 0.86]	4.3%	3.0%
Chu, 2014	1.28	[0.88; 1.87]	6.3%	3.9%
Han, 2013	0.99	[0.82; 1.20]	8.8%	17.9%
Hao, 2014	0.65	[0.40; 1.05]	5.2%	3.6%
Huang, 2017	0.94	[0.63; 1.39]	6.1%	4.3%
Kim, 2012	1.08	[0.67; 1.74]	5.1%	2.7%
Kou, 2014	0.66	[0.44; 0.99]	5.9%	5.0%
Li, 2016	0.45	[0.24; 0.85]	3.8%	2.4%
Li, 2015	1.87	[1.15; 3.04]	5.1%	2.0%
Li, 2010	0.66	[0.46; 0.96]	6.4%	5.6%
Qi, 2014	0.56	[0.39; 0.79]	6.6%	7.1%
Qi, 2010	1.09	[0.79; 1.50]	7.0%	5.9%
Toraih−2, 2016	0.41	[0.12; 1.46]	1.3%	0.8%
Yan, 2015	0.59	[0.42; 0.83]	6.8%	7.3%
Zhang, 2016	0.87	[0.59; 1.28]	6.2%	4.7%
Zhang, 2013	0.85	[0.70; 1.03]	8.8%	19.4%
Zhou, 2014	0.60	[0.38; 0.96]	5.3%	3.9%
**Fixed effect model**	**0.84**	**[0.77; 0.91]**	**−−**	**100.0%**
**Random effects model**	**0.80**	**[0.67; 0.96]**	**100.0%**	**−−**
Heterogeneity: $I^2 = 63\%$, $\tau^2 = 0.0612$, $p < 0.01$				
Test for effect in subgroup (fixed effect): $z = -4.18$ ($p < 0.01$)				
Test for effect in subgroup (random effects): $t_{17} = -2.62$ ($p = 0.02$)				
**BC**				
Afsharzadeh, 2017	1.12	[0.53; 2.36]	2.9%	1.0%
Alshatwi, 2012	0.49	[0.09; 2.74]	0.7%	0.3%
Bansal, 2014	0.75	[0.36; 1.60]	2.8%	1.3%
Bodal, 2017			0.0%	0.0%
Catucci, 1, 2010	0.88	[0.67; 1.16]	7.2%	8.5%
Catucci, 2, 2010	0.99	[0.79; 1.23]	7.9%	12.5%
Dai, 2016	0.65	[0.48; 0.86]	7.1%	9.2%
Doulah, 2018	1.12	[0.50; 2.51]	2.5%	0.9%
He, 2015	1.02	[0.77; 1.36]	7.1%	7.4%
Hoffman, 2009	0.51	[0.34; 0.79]	5.4%	4.9%
Hu, 2009	0.82	[0.68; 0.98]	8.3%	19.5%
Jedlinski, 2011	0.97	[0.56; 1.66]	4.3%	2.1%
Linhares, 2012	1.31	[0.96; 1.80]	6.7%	5.3%
Ma, 2013	0.86	[0.55; 1.34]	5.2%	3.4%
Mashayekhi, 2018	0.90	[0.58; 1.41]	5.2%	3.2%
Minh, 2018	1.07	[0.60; 1.91]	3.9%	1.8%
Morales, 2016	0.90	[0.64; 1.27]	6.4%	5.6%
Omrani, 2014			0.0%	0.0%
Qi, 2015	0.62	[0.45; 0.86]	6.6%	7.4%
Zhang, 2012	1.22	[0.86; 1.75]	6.2%	4.3%
Zhao, 2016	1.45	[0.80; 2.64]	3.8%	1.4%
**Fixed effect model**	**0.89**	**[0.82; 0.96]**	**−−**	**100.0%**
**Random effects model**	**0.90**	**[0.79; 1.02]**	**100.0%**	**−−**
Heterogeneity: $I^2 = 43\%$, $\tau^2 = 0.0255$, $p = 0.02$				
Test for effect in subgroup (fixed effect): $z = -2.91$ ($p < 0.01$)				
Test for effect in subgroup (random effects): $t_{17} = -1.82$ ($p = 0.09$)				
**GC**				
Ahn, 2013	0.87	[0.65; 1.16]	10.0%	10.1%
Dikeakos, 2014	0.18	[0.10; 0.32]	5.8%	8.3%
Gu, 2016	1.89	[1.14; 3.12]	6.6%	2.3%
Kupcinskas, 2014	0.71	[0.44; 1.12]	7.1%	4.4%
Li, 2016	0.69	[0.45; 1.04]	7.9%	5.6%
Okubo, 2010	0.91	[0.72; 1.16]	10.9%	14.4%
Parlayan, 2014	0.98	[0.66; 1.46]	8.2%	5.2%
Peng, 2010	0.82	[0.52; 1.31]	7.1%	4.2%
Poltronieri−Oliveira, 2017	2.48	[1.35; 4.55]	5.3%	1.3%
Pu, 2014	0.92	[0.57; 1.50]	6.8%	3.6%
Rogoveanu, 2017	0.93	[0.51; 1.69]	5.4%	2.3%
Su, 2016	1.17	[0.72; 1.93]	6.6%	3.0%
Wang, 2013	1.20	[1.04; 1.39]	12.5%	35.2%
**Fixed effect model**	**0.98**	**[0.90; 1.07]**	**−−**	**100.0%**
**Random effects model**	**0.92**	**[0.65; 1.29]**	**100.0%**	**−−**
Heterogeneity: $I^2 = 81\%$, $\tau^2 = 0.1448$, $p < 0.01$				
Test for effect in subgroup (fixed effect): $z = -0.42$ ($p = 0.67$)				
Test for effect in subgroup (random effects): $t_{17} = -0.54$ ($p = 0.60$)				
**CRC**				
Chen, 2012	1.08	[0.69; 1.69]	9.5%	6.8%
Dikaiakos, 2015	1.22	[0.82; 1.80]	10.6%	8.4%
Hezova, 2012	1.31	[0.72; 2.40]	6.9%	3.4%
Kupcinskas, 2014	1.12	[0.68; 1.85]	8.6%	5.4%
Lv, 2013	2.37	[1.72; 3.25]	12.3%	9.0%
Min, 2013	0.93	[0.70; 1.23]	13.1%	18.7%
Parlayan, 2014	1.07	[0.69; 1.67]	9.6%	7.0%
Vinci, 2013	1.23	[0.53; 2.87]	4.3%	1.8%
Zhan, 2011	0.67	[0.47; 0.94]	11.6%	15.0%
Zhu, 2012	0.71	[0.54; 0.92]	13.5%	24.5%
**Fixed effect model**	**1.04**	**[0.92; 1.17]**	**−−**	**100.0%**
**Random effects model**	**1.09**	**[0.83; 1.42]**	**100.0%**	**−−**
Heterogeneity: $I^2 = 79\%$, $\tau^2 = 0.1421$, $p < 0.01$				
Test for effect in subgroup (fixed effect): $z = 0.64$ ($p = 0.52$)				
Test for effect in subgroup (random effects): $t_{17} = 0.71$ ($p = 0.49$)				
**Others**				
Christensen, 2010	1.02	[0.73; 1.42]	5.3%	4.1%
Dou, 2010	0.90	[0.71; 1.14]	6.3%	8.7%
He, 2018	0.95	[0.72; 1.24]	5.9%	6.6%
Horikawa, 2008	0.72	[0.47; 1.11]	4.4%	3.0%
Hu, 2013	0.83	[0.66; 1.05]	6.3%	9.2%
Li, 2015	0.66	[0.48; 0.90]	5.4%	5.6%
Li, 2014	1.26	[1.04; 1.52]	6.7%	11.2%
Liu, 2015	0.88	[0.49; 1.57]	3.2%	1.5%
Liu, 2010	0.97	[0.78; 1.21]	6.5%	9.9%
Martin−Guerrero, 2015	2.34	[1.38; 3.95]	3.6%	1.0%
Miao, 2016	1.13	[0.75; 1.68]	4.6%	2.7%
Mulong Du, 2014	1.15	[0.95; 1.38]	6.8%	12.4%
Parlayan, 2014	1.00	[0.57; 1.73]	3.4%	1.5%
Pavlakis, 2013	1.54	[0.89; 2.65]	3.4%	1.3%
Peckham−Gregory, 2016	0.71	[0.41; 1.21]	3.5%	2.1%
Rakmanee, 2017	0.34	[0.18; 0.66]	2.7%	2.0%
Srivastava, 2017	1.03	[0.67; 1.59]	4.3%	2.4%
Srivastava, 2010	0.83	[0.42; 1.66]	2.6%	1.1%
Tong, 2014	0.71	[0.56; 0.91]	6.2%	9.4%
Toraih, 2016	1.63	[0.77; 3.46]	2.3%	0.7%
Toraih−1, 2016	1.59	[0.70; 3.62]	2.0%	0.5%
Zhou, 2011	0.93	[0.63; 1.38]	4.7%	3.1%
**Fixed effect model**	**0.97**	**[0.90; 1.04]**	**−−**	**100.0%**
**Random effects model**	**0.96**	**[0.83; 1.11]**	**100.0%**	**−−**
Heterogeneity: $I^2 = 62\%$, $\tau^2 = 0.0454$, $p < 0.01$				
Test for effect in subgroup (fixed effect): $z = -0.92$ ($p = 0.36$)				
Test for effect in subgroup (random effects): $t_{17} = -0.55$ ($p = 0.59$)				
**OC**				
Chu, 2012	0.90	[0.68; 1.20]	20.4%	23.0%
Liu, 2013	1.02	[0.62; 1.67]	13.5%	7.3%
Miao, 2016	0.75	[0.59; 0.94]	22.4%	39.7%
Roy, 2014	1.23	[0.78; 1.92]	14.8%	8.0%
Sushma, 2015	0.55	[0.29; 1.04]	10.1%	6.0%
Zhang, 2017	1.04	[0.75; 1.45]	18.8%	16.0%
**Fixed effect model**	**0.88**	**[0.76; 1.01]**	**−−**	**100.0%**
**Random effects model**	**0.89**	**[0.70; 1.14]**	**100.0%**	**−−**
Heterogeneity: $I^2 = 33\%$, $\tau^2 = 0.0162$, $p = 0.19$				
Test for effect in subgroup (fixed effect): $z = -1.86$ ($p = 0.06$)				
Test for effect in subgroup (random effects): $t_{17} = -1.19$ ($p = 0.29$)				
**PC**				
Damodaran, 2018	1.00	[0.48; 2.09]	17.7%	13.8%
George, 2011	0.42	[0.11; 1.56]	7.1%	7.9%
Hashemi, 2016	1.58	[0.73; 3.42]	16.6%	10.2%
Nikolic, 2015	0.84	[0.53; 1.34]	30.5%	37.8%
Parlayan, 2014	0.96	[0.58; 1.58]	28.1%	30.3%
**Fixed effect model**	**0.94**	**[0.71; 1.24]**	**−−**	**100.0%**
**Random effects model**	**0.95**	**[0.65; 1.37]**	**100.0%**	**−−**
Heterogeneity: $I^2 = 0\%$, $\tau^2 = 0$, $p = 0.49$				
Test for effect in subgroup (fixed effect): $z = -0.43$ ($p = 0.67$)				
Test for effect in subgroup (random effects): $t_{17} = -0.42$ ($p = 0.70$)				
**BlC**				
Deng, 2015	1.42	[0.93; 2.16]	26.8%	15.2%
Mittal, 2011	0.41	[0.14; 1.15]	8.5%	5.4%
Wang, 2016	0.45	[0.31; 0.67]	28.6%	32.9%
Yang, 2008	1.00	[0.77; 1.31]	36.1%	46.5%
**Fixed effect model**	**0.85**	**[0.71; 1.03]**	**−−**	**100.0%**
**Random effects model**	**0.77**	**[0.30; 1.97]**	**100.0%**	**−−**
Heterogeneity: $I^2 = 84\%$, $\tau^2 = 0.2443$, $p < 0.01$				
Test for effect in subgroup (fixed effect): $z = -1.65$ ($p = 0.10$)				
Test for effect in subgroup (random effects): $t_{17} = -0.88$ ($p = 0.44$)				
**LC**				
Hong, 2011	0.68	[0.50; 0.92]	12.5%	10.5%
Parlayan, 2014	0.89	[0.59; 1.35]	10.1%	5.0%
Sodhi, 2015	2.54	[1.09; 5.91]	4.4%	0.8%
Tian, 2009	0.91	[0.75; 1.10]	15.2%	23.6%
Vinci, 2011	1.60	[0.66; 3.88]	4.1%	0.8%
Yin, 2017	0.61	[0.49; 0.75]	14.8%	24.2%
Yin, 2016	0.84	[0.65; 1.09]	13.7%	13.5%
Yin, 2015	0.77	[0.53; 1.11]	11.2%	6.9%
Kim, 2010	0.81	[0.63; 1.04]	13.9%	14.8%
**Fixed effect model**	**0.80**	**[0.72; 0.88]**	**−−**	**100.0%**
**Random effects model**	**0.83**	**[0.66; 1.03]**	**100.0%**	**−−**
Heterogeneity: $I^2 = 59\%$, $\tau^2 = 0.0330$, $p = 0.01$				
Test for effect in subgroup (fixed effect): $z = -4.67$ ($p < 0.01$)				
Test for effect in subgroup (random effects): $t_{17} = -1.96$ ($p = 0.09$)				
**OvC**				
Liu, 2015	1.07	[0.55; 2.07]	16.1%	7.8%
Ni, 2016	0.87	[0.57; 1.33]	26.0%	21.0%
Song, 2016	0.61	[0.46; 0.82]	33.8%	52.6%
Sun, 2016	0.80	[0.50; 1.27]	24.1%	18.6%
**Fixed effect model**	**0.74**	**[0.60; 0.90]**	**−−**	**100.0%**
**Random effects model**	**0.75**	**[0.52; 1.07]**	**100.0%**	**−−**
Heterogeneity: $I^2 = 12\%$, $\tau^2 = 0.0066$, $p = 0.33$				
Test for effect in subgroup (fixed effect): $z = -2.93$ ($p < 0.01$)				
Test for effect in subgroup (random effects): $t_{17} = -2.60$ ($p = 0.08$)				
**ESCC**				
Qu, 2014	0.60	[0.41; 0.89]	15.3%	9.1%
Shen, 2016	0.92	[0.80; 1.07]	22.9%	50.1%
Umar, 2013	1.51	[0.78; 2.93]	8.7%	1.9%
Wang, 2014	1.07	[0.83; 1.39]	19.5%	15.1%
Wang, 2010	0.40	[0.28; 0.58]	16.0%	12.9%
Wei, 2013	0.92	[0.67; 1.27]	17.6%	10.8%
**Fixed effect model**	**0.86**	**[0.77; 0.96]**	**−−**	**100.0%**
**Random effects model**	**0.81**	**[0.52; 1.28]**	**100.0%**	**−−**
Heterogeneity: $I^2 = 81\%$, $\tau^2 = 0.0967$, $p < 0.01$				
Test for effect in subgroup (fixed effect): $z = -2.80$ ($p < 0.01$)				
Test for effect in subgroup (random effects): $t_{17} = -1.17$ ($p = 0.30$)				

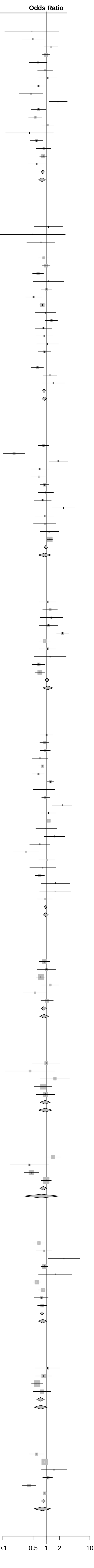

E) Allelic comparison

Study	OR	95%−CI	Weight (random)	Weight (fixed)
**HCC**				
Abdel−Hamid, 2018	1.00	[0.55; 1.81]	2.4%	0.7%
Akkiz, 2011	0.66	[0.49; 0.89]	5.2%	3.6%
Chu, 2014	1.09	[0.85; 1.41]	5.7%	3.8%
Han, 2013	1.03	[0.91; 1.16]	7.4%	16.7%
Hao, 2014	0.74	[0.58; 0.95]	5.8%	4.9%
Huang, 2017	0.98	[0.74; 1.29]	5.4%	3.3%
Kim, 2012	1.05	[0.78; 1.41]	5.2%	2.9%
Kou, 2014	0.77	[0.62; 0.94]	6.3%	6.8%
Li, 2016	0.66	[0.45; 0.96]	4.2%	2.2%
Li, 2015	1.49	[1.16; 1.90]	5.8%	3.5%
Li, 2010	0.74	[0.58; 0.95]	5.8%	5.0%
Qi, 2014	0.86	[0.70; 1.06]	6.3%	6.4%
Qi, 2010	1.05	[0.86; 1.29]	6.4%	6.2%
Toraih−2, 2016	1.13	[0.72; 1.79]	3.4%	1.1%
Yan, 2015	0.65	[0.51; 0.82]	6.0%	5.8%
Zhang, 2016	0.93	[0.71; 1.21]	5.5%	3.6%
Zhang, 2013	0.84	[0.74; 0.96]	7.4%	17.9%
Zhou, 2014	0.69	[0.54; 0.88]	5.9%	5.4%
**Fixed effect model**	**0.89**	**[0.85; 0.94]**	**−−**	**100.0%**
**Random effects model**	**0.88**	**[0.79; 0.99]**	**100.0%**	**−−**
Heterogeneity: $I^2 = 69\%$, $\tau^2 = 0.0297$, $p < 0.01$				
Test for effect in subgroup (fixed effect): $z = -4.31$ ($p < 0.01$)				
Test for effect in subgroup (random effects): $t_{17} = -2.36$ ($p = 0.03$)				
**BC**				
Afsharzadeh, 2017	0.86	[0.60; 1.24]	3.8%	1.7%
Alshatwi, 2012	1.23	[0.81; 1.88]	3.3%	1.0%
Bansal, 2014	0.83	[0.58; 1.20]	3.8%	1.7%
Bodal, 2017	1.53	[0.94; 2.50]	2.8%	0.7%
Catucci, 1, 2010	0.93	[0.82; 1.07]	6.4%	11.6%
Catucci, 2, 2010	0.99	[0.89; 1.11]	6.6%	15.8%
Dai, 2016	0.73	[0.62; 0.86]	6.1%	8.7%
Doulah, 2018	0.86	[0.58; 1.28]	3.5%	1.4%
He, 2015	1.06	[0.88; 1.28]	5.8%	5.7%
Hoffman, 2009	0.74	[0.61; 0.90]	5.8%	6.3%
Hu, 2009	0.85	[0.75; 0.96]	6.5%	14.9%
Jedlinski, 2011	0.94	[0.70; 1.27]	4.5%	2.4%
Linhares, 2012	1.32	[1.08; 1.61]	5.7%	4.5%
Ma, 2013	1.00	[0.75; 1.33]	4.7%	2.5%
Mashayekhi, 2018	1.02	[0.82; 1.27]	5.5%	4.3%
Minh, 2018	0.71	[0.50; 1.02]	3.9%	1.9%
Morales, 2016	0.95	[0.80; 1.13]	6.0%	7.1%
Omrani, 2014	0.60	[0.32; 1.12]	2.0%	0.7%
Qi, 2015	0.65	[0.50; 0.84]	4.9%	3.7%
Zhang, 2012	1.23	[0.92; 1.64]	4.6%	2.2%
Zhao, 2016	1.09	[0.76; 1.58]	3.8%	1.4%
**Fixed effect model**	**0.93**	**[0.88; 0.97]**	**−−**	**100.0%**
**Random effects model**	**0.93**	**[0.85; 1.02]**	**100.0%**	**−−**
Heterogeneity: $I^2 = 63\%$, $\tau^2 = 0.0204$, $p < 0.01$				
Test for effect in subgroup (fixed effect): $z = -3.25$ ($p < 0.01$)				
Test for effect in subgroup (random effects): $t_{17} = -1.54$ ($p = 0.14$)				
**GC**				
Ahn, 2013	0.90	[0.75; 1.09]	8.7%	9.2%
Dikeakos, 2014	0.21	[0.15; 0.27]	6.9%	8.6%
Gu, 2016	1.51	[1.13; 2.01]	6.9%	2.9%
Kupcinskas, 2014	1.05	[0.84; 1.31]	8.1%	6.1%
Li, 2016	0.68	[0.50; 0.93]	6.5%	3.7%
Okubo, 2010	0.94	[0.80; 1.10]	9.2%	12.1%
Parlayan, 2014	0.86	[0.67; 1.11]	7.6%	5.1%
Peng, 2010	0.77	[0.59; 1.01]	7.2%	4.6%
Poltronieri−Oliveira, 2017	1.24	[0.92; 1.68]	6.7%	2.9%
Pu, 2014	0.89	[0.69; 1.14]	7.5%	5.0%
Rogoveanu, 2017	0.96	[0.71; 1.29]	6.8%	3.4%
Su, 2016	1.13	[0.89; 1.44]	7.7%	4.8%
Wang, 2013	1.22	[1.11; 1.33]	10.1%	31.6%
**Fixed effect model**	**0.98**	**[0.93; 1.03]**	**−−**	**100.0%**
**Random effects model**	**0.88**	**[0.66; 1.18]**	**100.0%**	**−−**
Heterogeneity: $I^2 = 92\%$, $\tau^2 = 0.1406$, $p < 0.01$				
Test for effect in subgroup (fixed effect): $z = -0.79$ ($p = 0.43$)				
Test for effect in subgroup (random effects): $t_{17} = -0.94$ ($p = 0.36$)				
**CRC**				
Chen, 2012	1.07	[0.80; 1.41]	9.2%	6.4%
Dikaiakos, 2015	1.09	[0.82; 1.45]	9.1%	6.2%
Hezova, 2012	1.05	[0.79; 1.40]	9.1%	6.2%
Kupcinskas, 2014	1.17	[0.91; 1.50]	9.9%	7.6%
Lv, 2013	1.99	[1.63; 2.42]	11.2%	9.7%
Min, 2013	0.84	[0.70; 1.01]	11.5%	17.5%
Parlayan, 2014	1.05	[0.79; 1.39]	9.1%	6.3%
Vinci, 2013	1.24	[0.89; 1.71]	8.3%	4.5%
Zhan, 2011	0.76	[0.61; 0.93]	10.8%	13.5%
Zhu, 2012	0.81	[0.69; 0.96]	11.9%	22.0%
**Fixed effect model**	**1.03**	**[0.96; 1.11]**	**−−**	**100.0%**
**Random effects model**	**1.06**	**[0.87; 1.30]**	**100.0%**	**−−**
Heterogeneity: $I^2 = 86\%$, $\tau^2 = 0.0889$, $p < 0.01$				
Test for effect in subgroup (fixed effect): $z = 0.89$ ($p = 0.37$)				
Test for effect in subgroup (random effects): $t_{17} = 0.68$ ($p = 0.52$)				
**Others**				
Christensen, 2010	0.93	[0.78; 1.11]	5.4%	6.1%
Dou, 2010	1.05	[0.90; 1.22]	5.6%	7.4%
He, 2018	0.95	[0.80; 1.13]	5.4%	6.4%
Horikawa, 2008	0.87	[0.69; 1.11]	4.7%	3.4%
Hu, 2013	0.80	[0.69; 0.93]	5.6%	9.0%
Li, 2015	0.71	[0.57; 0.89]	4.8%	4.2%
Li, 2014	1.13	[1.00; 1.27]	5.9%	11.2%
Liu, 2015	1.15	[0.80; 1.66]	3.4%	1.3%
Liu, 2010	1.04	[0.92; 1.17]	5.9%	12.6%
Martin−Guerrero, 2015	1.50	[1.10; 2.05]	3.9%	1.5%
Miao, 2016	1.12	[0.85; 1.48]	4.3%	2.3%
Mulong Du, 2014	1.20	[1.05; 1.35]	5.8%	10.6%
Parlayan, 2014	0.84	[0.59; 1.19]	3.6%	1.6%
Pavlakis, 2013	1.23	[0.82; 1.85]	3.1%	1.0%
Peckham−Gregory, 2016	0.86	[0.67; 1.11]	4.6%	3.2%
Rakmanee, 2017	0.47	[0.33; 0.68]	3.5%	2.1%
Srivastava, 2017	1.06	[0.78; 1.44]	4.0%	1.8%
Srivastava, 2010	1.17	[0.87; 1.57]	4.1%	1.9%
Tong, 2014	0.88	[0.75; 1.03]	5.5%	7.6%
Toraih, 2016	1.59	[1.10; 2.29]	3.4%	1.1%
Toraih−1, 2016	1.69	[1.10; 2.59]	2.9%	0.7%
Zhou, 2011	0.94	[0.74; 1.20]	4.6%	3.2%
**Fixed effect model**	**1.00**	**[0.96; 1.05]**	**−−**	**100.0%**
**Random effects model**	**1.00**	**[0.90; 1.12]**	**100.0%**	**−−**
Heterogeneity: $I^2 = 72\%$, $\tau^2 = 0.0276$, $p < 0.01$				
Test for effect in subgroup (fixed effect): $z = 0.15$ ($p = 0.88$)				
Test for effect in subgroup (random effects): $t_{17} = 0.02$ ($p = 0.98$)				
**OC**				
Chu, 2012	1.14	[0.94; 1.37]	19.4%	19.7%
Liu, 2013	1.18	[0.85; 1.64]	13.9%	6.2%
Miao, 2016	0.79	[0.69; 0.92]	20.9%	38.0%
Roy, 2014	1.20	[0.98; 1.47]	18.7%	16.2%
Sushma, 2015	0.41	[0.25; 0.69]	8.8%	4.5%
Zhang, 2017	1.13	[0.91; 1.39]	18.4%	15.3%
**Fixed effect model**	**0.98**	**[0.90; 1.07]**	**−−**	**100.0%**
**Random effects model**	**0.97**	**[0.67; 1.40]**	**100.0%**	**−−**
Heterogeneity: $I^2 = 82\%$, $\tau^2 = 0.0575$, $p < 0.01$				
Test for effect in subgroup (fixed effect): $z = -0.36$ ($p = 0.72$)				
Test for effect in subgroup (random effects): $t_{17} = -0.22$ ($p = 0.84$)				
**PC**				
Damodaran, 2018	1.37	[0.92; 2.06]	15.8%	9.6%
George, 2011	1.23	[0.91; 1.68]	20.2%	17.3%
Hashemi, 2016	1.19	[0.87; 1.63]	19.9%	17.1%
Nikolic, 2015	0.89	[0.71; 1.11]	24.5%	38.1%
Parlayan, 2014	1.01	[0.74; 1.39]	19.6%	17.9%
**Fixed effect model**	**1.07**	**[0.94; 1.22]**	**−−**	**100.0%**
**Random effects model**	**1.09**	**[0.87; 1.35]**	**100.0%**	**−−**
Heterogeneity: $I^2 = 27\%$, $\tau^2 = 0.0089$, $p = 0.24$				
Test for effect in subgroup (fixed effect): $z = 0.98$ ($p = 0.33$)				
Test for effect in subgroup (random effects): $t_{17} = 1.05$ ($p = 0.35$)				
**BIC**				
Deng, 2015	1.00	[0.76; 1.32]	22.8%	14.3%
Mittal, 2011	1.11	[0.84; 1.46]	22.5%	13.2%
Wang, 2016	0.70	[0.56; 0.89]	25.0%	23.3%
Yang, 2008	1.01	[0.87; 1.17]	29.7%	49.2%
**Fixed effect model**	**0.95**	**[0.86; 1.06]**	**−−**	**100.0%**
**Random effects model**	**0.94**	**[0.69; 1.29]**	**100.0%**	**−−**
Heterogeneity: $I^2 = 65\%$, $\tau^2 = 0.0238$, $p = 0.04$				
Test for effect in subgroup (fixed effect): $z = -0.94$ ($p = 0.35$)				
Test for effect in subgroup (random effects): $t_{17} = -0.61$ ($p = 0.58$)				
**LC**				
Hong, 2011	0.88	[0.73; 1.07]	11.5%	9.2%
Parlayan, 2014	0.98	[0.75; 1.26]	10.0%	4.9%
Sodhi, 2015	1.42	[1.10; 1.84]	10.0%	4.0%
Tian, 2009	0.89	[0.79; 1.01]	13.1%	23.0%
Vinci, 2011	1.37	[0.93; 2.02]	7.2%	1.8%
Yin, 2017	0.79	[0.69; 0.89]	13.0%	23.5%
Yin, 2016	0.93	[0.79; 1.09]	12.2%	12.8%
Yin, 2015	0.92	[0.72; 1.16]	10.5%	6.1%
Kim, 2010	0.84	[0.72; 0.98]	12.4%	14.7%
**Fixed effect model**	**0.90**	**[0.85; 0.95]**	**−−**	**100.0%**
**Random effects model**	**0.94**	**[0.81; 1.08]**	**100.0%**	**−−**
Heterogeneity: $I^2 = 64\%$, $\tau^2 = 0.0152$, $p < 0.01$				
Test for effect in subgroup (fixed effect): $z = -3.60$ ($p < 0.01$)				
Test for effect in subgroup (random effects): $t_{17} = -1.03$ ($p = 0.33$)				
**OvC**				
Liu, 2015	1.40	[0.91; 2.14]	17.6%	7.1%
Ni, 2016	0.92	[0.70; 1.20]	26.4%	22.1%
Song, 2016	0.74	[0.61; 0.89]	31.8%	52.3%
Sun, 2016	0.79	[0.58; 1.07]	24.2%	18.5%
**Fixed effect model**	**0.83**	**[0.73; 0.95]**	**−−**	**100.0%**
**Random effects model**	**0.89**	**[0.59; 1.33]**	**100.0%**	**−−**
Heterogeneity: $I^2 = 62\%$, $\tau^2 = 0.0320$, $p = 0.05$				
Test for effect in subgroup (fixed effect): $z = -2.73$ ($p < 0.01$)				
Test for effect in subgroup (random effects): $t_{17} = -0.95$ ($p = 0.41$)				
**ESCC**				
Qu, 2014	0.84	[0.69; 1.02]	16.2%	11.0%
Shen, 2016	0.91	[0.82; 1.00]	19.3%	45.5%
Umar, 2013	1.20	[0.93; 1.56]	14.3%	5.5%
Wang, 2014	1.09	[0.93; 1.28]	17.5%	14.7%
Wang, 2010	0.69	[0.57; 0.83]	16.8%	14.3%
Wei, 2013	1.09	[0.89; 1.33]	16.0%	9.0%
**Fixed effect model**	**0.93**	**[0.87; 0.99]**	**−−**	**100.0%**
**Random effects model**	**0.94**	**[0.76; 1.17]**	**100.0%**	**−−**
Heterogeneity: $I^2 = 77\%$, $\tau^2 = 0.0242$, $p < 0.01$				
Test for effect in subgroup (fixed effect): $z = -2.31$ ($p = 0.02$)				
Test for effect in subgroup (random effects): $t_{17} = -0.70$ ($p = 0.51$)				

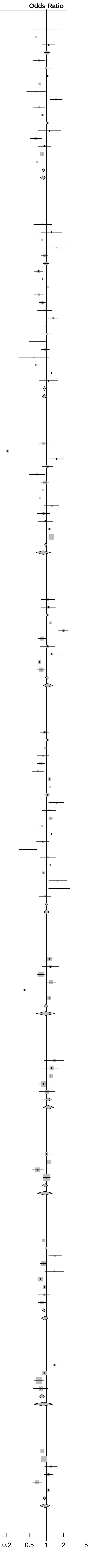

Supplement: Supplementary file 1 [file Data_Sheet_1.ZIP › Supplementary Figures/Suppl. Fig. S3.pdf]
