# Supplementary material for: Association of mir-196a-2 rs11614913 and mir-149 rs2292832 Polymorphisms With Risk of Cancer: An Updated Meta-Analysis
Source: Front Genet. 2019 Mar 15;10:186. doi: 10.3389/fgene.2019.00186 (PMC6429108; doi:10.3389/fgene.2019.00186)

**Supplementary Figure S4.** Forest plots for association of miR-149 rs2292832 and cancer risk under different genetic models. **A)** Homozygote comparison with trim & fill" approach; **B)** Homozygote comparison using original studies; **C)** Heterozygote comparison; **D)** Dominant comparison; **E)** Recessive comparison with "trim & fill" approach; **F)** Recessive comparison using original studies; **G)** Allele contrast with "trim & fill" approach; H) Allele contrast using original studies

**A) Homozygote comparison - trim & fill**

| Studies | OR | 95% − CI | Weights |
|---|---|---|---|
| Ahn, 2012 | 1.00 | [0.63; 1.59] | 2.2% |
| Chu, 2012 | 0.77 | [0.46; 1.30] | 2.0% |
| Chu, 2014 | 1.17 | [0.59; 2.35] | 1.6% |
| Cîmpeanu, 2017 | 0.99 | [0.55; 1.77] | 1.8% |
| Dikeakos, 2014 | 0.44 | [0.24; 0.80] | 1.8% |
| Dong, 2015 | 0.59 | [0.40; 0.86] | 2.5% |
| Du, 2014 | 0.64 | [0.40; 1.05] | 2.1% |
| Gu, 2016 | 1.17 | [0.67; 2.08] | 1.9% |
| Hashemi, 2016 | 1.31 | [0.69; 2.48] | 1.7% |
| He, 2015 | 1.91 | [1.21; 3.00] | 2.2% |
| He, 2018 | 0.94 | [0.60; 1.48] | 2.2% |
| Hu, 2009 | 0.98 | [0.73; 1.33] | 2.7% |
| Hu, 2013 | 1.13 | [0.79; 1.61] | 2.5% |
| Huang, 2013 | 1.36 | [0.74; 2.50] | 1.8% |
| Jiang, 2016 | 0.95 | [0.66; 1.36] | 2.5% |
| Kim, 2012 | 1.46 | [0.70; 3.08] | 1.5% |
| Kontorovich, 2010 | 0.62 | [0.36; 1.09] | 1.9% |
| Kou, 2014 | 0.81 | [0.51; 1.29] | 2.2% |
| Li, 2016 | 1.23 | [0.78; 1.94] | 2.2% |
| Liu, 2010 | 0.90 | [0.66; 1.22] | 2.7% |
| Liu, 2014 | 0.50 | [0.33; 0.77] | 2.3% |
| Liu, 2015 | 1.57 | [0.93; 2.66] | 2.0% |
| Lv, 2013 | 1.31 | [0.81; 2.14] | 2.1% |
| Miao, 2016 | 1.12 | [0.81; 1.54] | 2.6% |
| Min, 2012 | 1.01 | [0.66; 1.56] | 2.3% |
| Mw Zhang−a, 2012 | 1.00 | [0.64; 1.56] | 2.2% |
| Mw Zhang−b, 2012 | 0.99 | [0.59; 1.66] | 2.0% |
| Ni, 2016 | 1.09 | [0.61; 1.94] | 1.9% |
| Pratedrat, 2015 | 0.87 | [0.34; 2.24] | 1.1% |
| Pu, 2014 | 0.95 | [0.55; 1.64] | 2.0% |
| Ranjbar, 2018 | 0.61 | [0.17; 2.16] | 0.7% |
| Su, 2016 | 1.33 | [0.73; 2.42] | 1.8% |
| Sushma, 2015 | 16.23 | [0.90; 292.86] | 0.2% |
| Tandon, 2017 | 2.86 | [1.32; 6.21] | 1.4% |
| Tian, 2009 | 0.90 | [0.67; 1.19] | 2.7% |
| Tu, 2012 | 2.66 | [1.32; 5.33] | 1.6% |
| Vinci, 2011 | 2.15 | [0.91; 5.07] | 1.2% |
| Vinci, 2013 | 1.47 | [0.73; 2.96] | 1.6% |
| Wang, 2014 | 0.71 | [0.49; 1.03] | 2.5% |
| Wang, 2014 | 1.96 | [0.98; 3.91] | 1.6% |
| Wei, 2014 | 0.63 | [0.46; 0.86] | 2.7% |
| Yan, 2015 | 0.82 | [0.52; 1.28] | 2.2% |
| Yang, 2016 | 0.82 | [0.19; 3.58] | 0.6% |
| Zhang, 2012 | 1.36 | [0.72; 2.56] | 1.7% |
| Filled: Zhang, 2012 | 0.58 | [0.31; 1.09] | 1.7% |
| Filled: Kim, 2012 | 0.54 | [0.26; 1.13] | 1.5% |
| Filled: Vinci, 2013 | 0.54 | [0.27; 1.08] | 1.6% |
| Filled: Liu, 2015 | 0.50 | [0.30; 0.85] | 2.0% |
| Filled: He, 2015 | 0.41 | [0.26; 0.65] | 2.2% |
| Filled: Wang, 2014 | 0.40 | [0.20; 0.80] | 1.6% |
| Filled: Vinci, 2011 | 0.37 | [0.16; 0.87] | 1.2% |
| Filled: Tu, 2012 | 0.30 | [0.15; 0.60] | 1.6% |
| Filled: Tandon, 2017 | 0.28 | [0.13; 0.60] | 1.4% |
| Filled: Sushma, 2015 | 0.05 | [0.00; 0.88] | 0.2% |
| **Random effects model** | **0.90** | **[0.78; 1.03]** | **100.0%** |

Heterogeneity: $I^2 = 64\%$, $\tau^2 = 0.1189$, $p < 0.01$

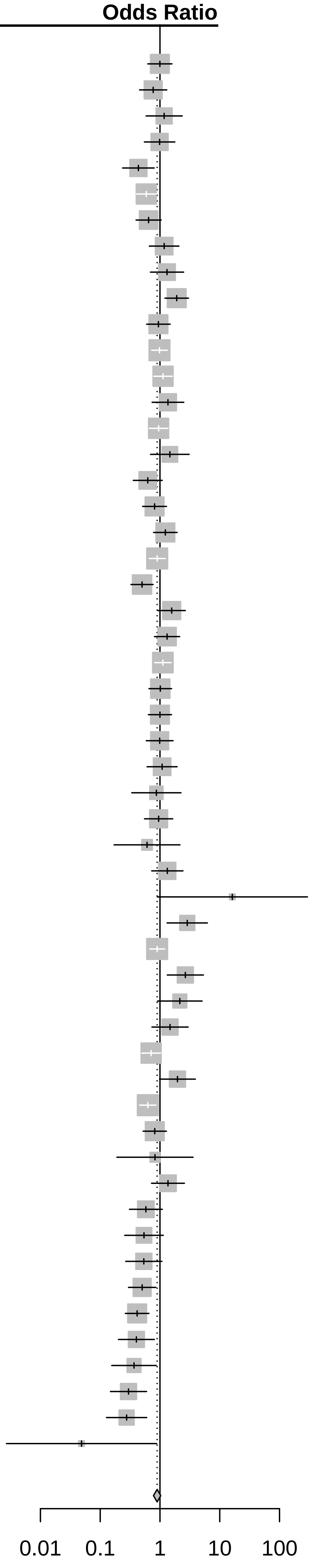

**B) Homozygote comparison using original studies**

| Studies | OR | 95% − CI | Weights |
|---|---|---|---|
| Ahn, 2012 | 1.00 | [0.63; 1.59] | 2.6% |
| Chu, 2012 | 0.77 | [0.46; 1.30] | 2.3% |
| Chu, 2014 | 1.17 | [0.59; 2.35] | 1.7% |
| Cîmpeanu, 2017 | 0.99 | [0.55; 1.77] | 2.1% |
| Dikeakos, 2014 | 0.44 | [0.24; 0.80] | 2.0% |
| Dong, 2015 | 0.59 | [0.40; 0.86] | 3.1% |
| Du, 2014 | 0.64 | [0.40; 1.05] | 2.5% |
| Gu, 2016 | 1.17 | [0.67; 2.08] | 2.1% |
| Hashemi, 2016 | 1.31 | [0.69; 2.48] | 1.9% |
| He, 2015 | 1.91 | [1.21; 3.00] | 2.7% |
| He, 2018 | 0.94 | [0.60; 1.48] | 2.7% |
| Hu, 2009 | 0.98 | [0.73; 1.33] | 3.5% |
| Hu, 2013 | 1.13 | [0.79; 1.61] | 3.2% |
| Huang, 2013 | 1.36 | [0.74; 2.50] | 2.0% |
| Jiang, 2016 | 0.95 | [0.66; 1.36] | 3.2% |
| Kim, 2012 | 1.46 | [0.70; 3.08] | 1.5% |
| Kontorovich, 2010 | 0.62 | [0.36; 1.09] | 2.2% |
| Kou, 2014 | 0.81 | [0.51; 1.29] | 2.6% |
| Li, 2016 | 1.23 | [0.78; 1.94] | 2.7% |
| Liu, 2010 | 0.90 | [0.66; 1.22] | 3.5% |
| Liu, 2014 | 0.50 | [0.33; 0.77] | 2.8% |
| Liu, 2015 | 1.57 | [0.93; 2.66] | 2.3% |
| Lv, 2013 | 1.31 | [0.81; 2.14] | 2.5% |
| Miao, 2016 | 1.12 | [0.81; 1.54] | 3.4% |
| Min, 2012 | 1.01 | [0.66; 1.56] | 2.8% |
| Mw Zhang−a, 2012 | 1.00 | [0.64; 1.56] | 2.7% |
| Mw Zhang−b, 2012 | 0.99 | [0.59; 1.66] | 2.4% |
| Ni, 2016 | 1.09 | [0.61; 1.94] | 2.1% |
| Pratedrat, 2015 | 0.87 | [0.34; 2.24] | 1.1% |
| Pu, 2014 | 0.95 | [0.55; 1.64] | 2.2% |
| Ranjbar, 2018 | 0.61 | [0.17; 2.16] | 0.7% |
| Su, 2016 | 1.33 | [0.73; 2.42] | 2.0% |
| Sushma, 2015 | 16.23 | [0.90; 292.86] | 0.1% |
| Tandon, 2017 | 2.86 | [1.32; 6.21] | 1.4% |
| Tian, 2009 | 0.90 | [0.67; 1.19] | 3.6% |
| Tu, 2012 | 2.66 | [1.32; 5.33] | 1.7% |
| Vinci, 2011 | 2.15 | [0.91; 5.07] | 1.2% |
| Vinci, 2013 | 1.47 | [0.73; 2.96] | 1.7% |
| Wang, 2014 | 0.71 | [0.49; 1.03] | 3.1% |
| Wang, 2014 | 1.96 | [0.98; 3.91] | 1.7% |
| Wei, 2014 | 0.63 | [0.46; 0.86] | 3.5% |
| Yan, 2015 | 0.82 | [0.52; 1.28] | 2.7% |
| Yang, 2016 | 0.82 | [0.19; 3.58] | 0.5% |
| Zhang, 2012 | 1.36 | [0.72; 2.56] | 1.9% |
| **Random effects model** | **1.01** | **[0.90; 1.14]** | **100.0%** |

Heterogeneity: $I^2 = 53\%$, $\tau^2 = 0.0670$, $p < 0.01$

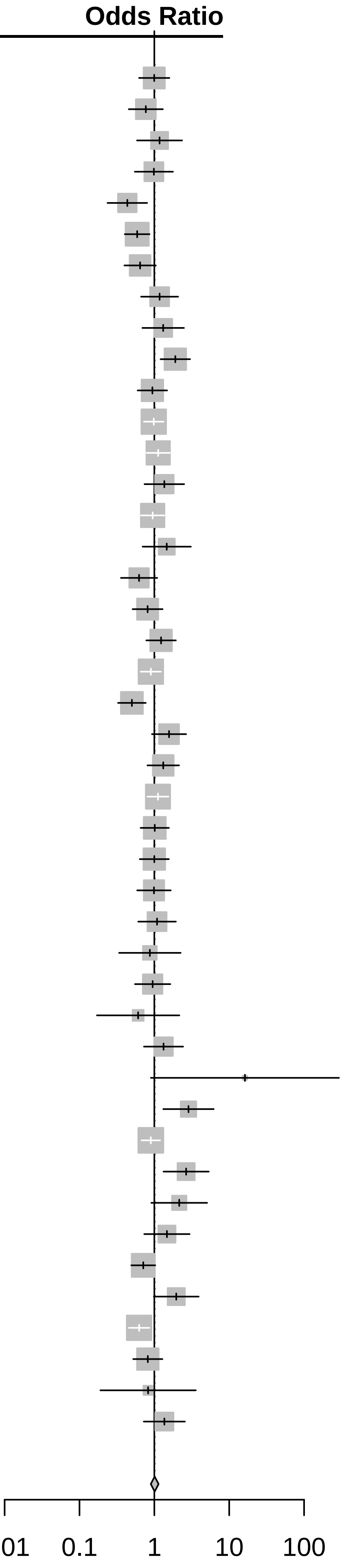

**C) Heterozygote comparison**

| Studies | OR | 95% − CI | Weights |
|---|---|---|---|
| Ahn, 2012 | 0.86 | [0.53; 1.38] | 2.2% |
| Chu, 2012 | 0.74 | [0.41; 1.32] | 1.6% |
| Chu, 2014 | 1.17 | [0.54; 2.54] | 1.0% |
| Cîmpeanu, 2017 | 0.74 | [0.47; 1.14] | 2.4% |
| Dikeakos, 2014 | 0.58 | [0.31; 1.07] | 1.5% |
| Dong, 2015 | 0.75 | [0.52; 1.08] | 3.0% |
| Du, 2014 | 0.87 | [0.53; 1.41] | 2.2% |
| Gu, 2016 | 1.12 | [0.70; 1.79] | 2.3% |
| Hashemi, 2016 | 1.56 | [0.99; 2.48] | 2.3% |
| He, 2015 | 1.62 | [1.02; 2.57] | 2.3% |
| He, 2018 | 0.66 | [0.40; 1.12] | 2.0% |
| Hu, 2009 | 0.98 | [0.82; 1.17] | 5.4% |
| Hu, 2013 | 1.10 | [0.76; 1.57] | 3.1% |
| Huang, 2013 | 1.05 | [0.57; 1.92] | 1.6% |
| Jiang, 2016 | 0.82 | [0.55; 1.22] | 2.8% |
| Kim, 2012 | 0.99 | [0.47; 2.09] | 1.1% |
| Kontorovich, 2010 | 0.81 | [0.45; 1.46] | 1.6% |
| Kou, 2014 | 0.86 | [0.63; 1.18] | 3.6% |
| Li, 2016 | 1.23 | [0.78; 1.93] | 2.4% |
| Liu, 2010 | 1.00 | [0.84; 1.19] | 5.5% |
| Liu, 2014 | 0.69 | [0.46; 1.04] | 2.7% |
| Liu, 2015 | 1.24 | [0.86; 1.80] | 3.0% |
| Lv, 2013 | 0.99 | [0.57; 1.73] | 1.8% |
| Miao, 2016 | 1.02 | [0.74; 1.42] | 3.5% |
| Min, 2012 | 0.86 | [0.55; 1.33] | 2.5% |
| Mw Zhang−a, 2012 | 0.87 | [0.55; 1.35] | 2.4% |
| Mw Zhang−b, 2012 | 0.72 | [0.43; 1.21] | 1.9% |
| Ni, 2016 | 1.05 | [0.68; 1.62] | 2.5% |
| Pratedrat, 2015 | 0.92 | [0.33; 2.60] | 0.6% |
| Pu, 2014 | 0.66 | [0.34; 1.25] | 1.4% |
| Ranjbar, 2018 | 0.29 | [0.07; 1.15] | 0.4% |
| Su, 2016 | 1.10 | [0.78; 1.57] | 3.2% |
| Sushma, 2015 | 2.29 | [1.06; 4.94] | 1.1% |
| Tandon, 2017 | 2.87 | [1.53; 5.38] | 1.5% |
| Tian, 2009 | 0.95 | [0.71; 1.26] | 3.9% |
| Tu, 2012 | 2.60 | [1.30; 5.20] | 1.3% |
| Vinci, 2011 | 1.14 | [0.65; 2.00] | 1.8% |
| Vinci, 2013 | 0.84 | [0.53; 1.33] | 2.3% |
| Wang, 2014 | 0.69 | [0.48; 1.01] | 3.0% |
| Wang, 2014 | 1.61 | [0.81; 3.18] | 1.3% |
| Wei, 2014 | 0.68 | [0.50; 0.94] | 3.6% |
| Yan, 2015 | 0.93 | [0.62; 1.40] | 2.7% |
| Yang, 2016 | 0.98 | [0.24; 4.07] | 0.3% |
| Zhang, 2012 | 0.94 | [0.50; 1.77] | 1.5% |
| **Random effects model** | **0.96** | **[0.88; 1.06]** | **100.0%** |

Heterogeneity: $I^2 = 37\%$, $\tau^2 = 0.0269$, $p < 0.01$

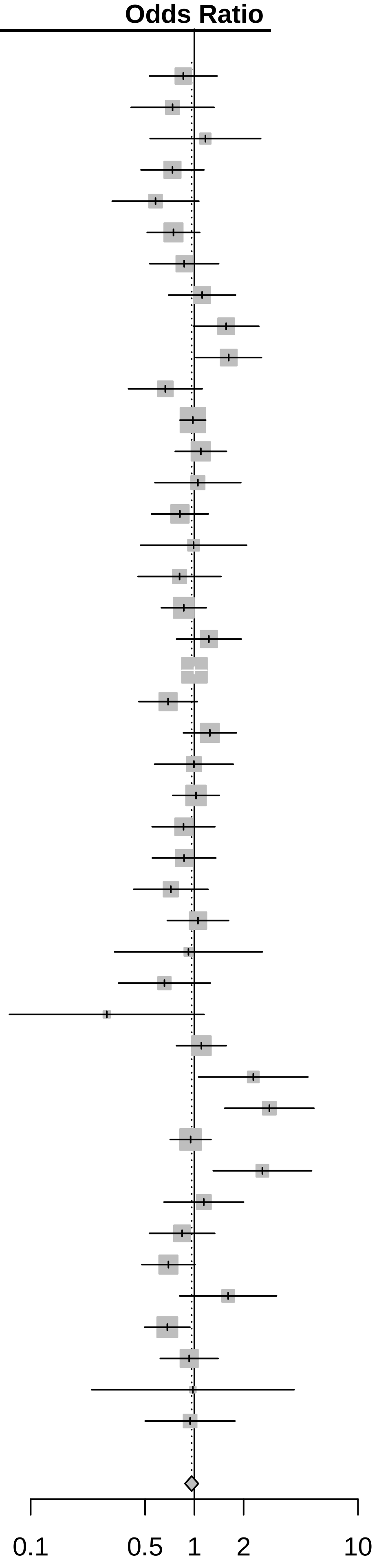

D) **Dominant model**

| Studies | OR | 95% − CI | Weights |
|---|---|---|---|
| Ahn, 2012 | 0.93 | [0.59; 1.46] | 2.3% |
| Chu, 2012 | 0.76 | [0.45; 1.28] | 1.9% |
| Chu, 2014 | 1.17 | [0.59; 2.33] | 1.3% |
| Cîmpeanu, 2017 | 0.80 | [0.53; 1.20] | 2.5% |
| Dikeakos, 2014 | 0.50 | [0.28; 0.89] | 1.7% |
| Dong, 2015 | 0.67 | [0.47; 0.95] | 2.9% |
| Du, 2014 | 0.75 | [0.47; 1.18] | 2.2% |
| Gu, 2016 | 1.13 | [0.73; 1.76] | 2.3% |
| Hashemi, 2016 | 1.49 | [0.98; 2.27] | 2.4% |
| He, 2015 | 1.77 | [1.14; 2.74] | 2.3% |
| He, 2018 | 0.88 | [0.56; 1.37] | 2.3% |
| Hu, 2009 | 0.98 | [0.82; 1.16] | 4.2% |
| Hu, 2013 | 1.11 | [0.79; 1.56] | 2.9% |
| Huang, 2013 | 1.19 | [0.67; 2.09] | 1.7% |
| Jiang, 2016 | 0.92 | [0.64; 1.31] | 2.8% |
| Kim, 2012 | 1.21 | [0.59; 2.46] | 1.3% |
| Kontorovich, 2010 | 0.71 | [0.44; 1.13] | 2.2% |
| Kou, 2014 | 0.85 | [0.63; 1.15] | 3.2% |
| Li, 2016 | 1.23 | [0.80; 1.90] | 2.4% |
| Liu, 2010 | 0.98 | [0.83; 1.16] | 4.2% |
| Liu, 2014 | 0.60 | [0.41; 0.87] | 2.7% |
| Liu, 2015 | 1.31 | [0.92; 1.87] | 2.9% |
| Lv, 2013 | 1.23 | [0.76; 1.99] | 2.1% |
| Miao, 2016 | 1.07 | [0.79; 1.46] | 3.2% |
| Min, 2012 | 0.94 | [0.62; 1.42] | 2.5% |
| Mw Zhang−a, 2012 | 0.93 | [0.61; 1.42] | 2.4% |
| Mw Zhang−b, 2012 | 0.85 | [0.52; 1.38] | 2.1% |
| Ni, 2016 | 1.06 | [0.70; 1.60] | 2.5% |
| Pratedrat, 2015 | 0.88 | [0.35; 2.24] | 0.8% |
| Pu, 2014 | 0.88 | [0.51; 1.50] | 1.9% |
| Ranjbar, 2018 | 0.49 | [0.14; 1.72] | 0.5% |
| Su, 2016 | 1.14 | [0.81; 1.59] | 3.0% |
| Sushma, 2015 | 2.92 | [1.39; 6.14] | 1.2% |
| Tandon, 2017 | 2.87 | [1.72; 4.78] | 2.0% |
| Tian, 2009 | 0.92 | [0.70; 1.21] | 3.4% |
| Tu, 2012 | 2.63 | [1.37; 5.06] | 1.4% |
| Vinci, 2011 | 1.32 | [0.78; 2.22] | 1.9% |
| Vinci, 2013 | 0.96 | [0.63; 1.47] | 2.4% |
| Wang, 2014 | 0.70 | [0.49; 1.00] | 2.8% |
| Wang, 2014 | 1.76 | [0.92; 3.39] | 1.4% |
| Wei, 2014 | 0.65 | [0.48; 0.88] | 3.2% |
| Yan, 2015 | 0.89 | [0.61; 1.30] | 2.7% |
| Yang, 2016 | 0.91 | [0.23; 3.60] | 0.4% |
| Zhang, 2012 | 1.13 | [0.62; 2.06] | 1.6% |
| **Random effects model** | **1.00** | **[0.90; 1.11]** | **100.0%** |

Heterogeneity: $I^2 = 54\%$, $\tau^2 = 0.0459$, $p < 0.01$

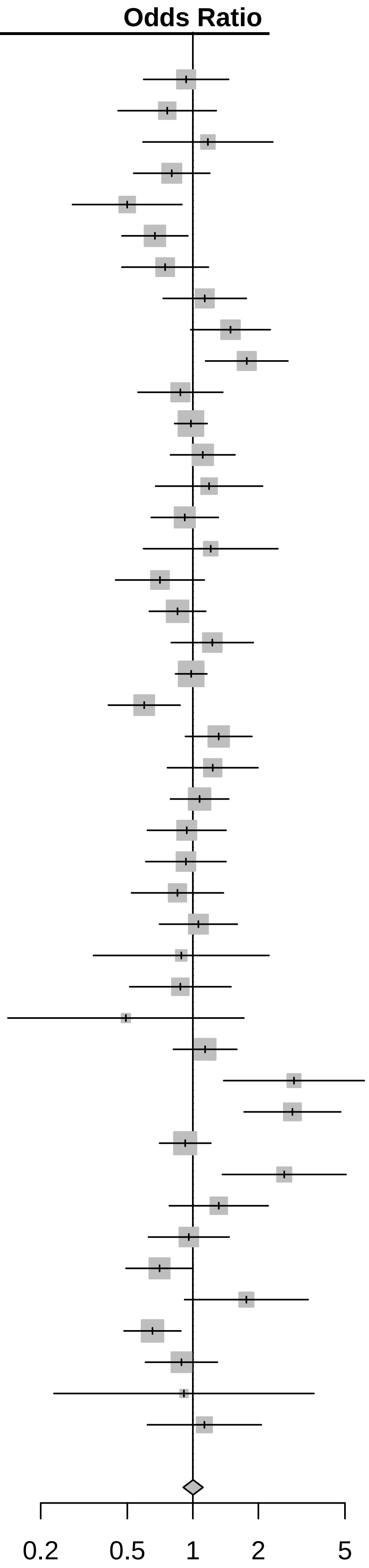

E) **Recessive model - trim & fill**

| Studies | OR | 95% − CI | Weights |
|---|---|---|---|
| Ahn, 2012 | 1.13 | [0.87; 1.47] | 2.8% |
| Chu, 2012 | 0.96 | [0.72; 1.30] | 2.5% |
| Chu, 2014 | 1.05 | [0.70; 1.57] | 1.8% |
| Cîmpeanu, 2017 | 1.13 | [0.65; 1.97] | 1.2% |
| Dikeakos, 2014 | 0.68 | [0.48; 0.97] | 2.1% |
| Dong, 2015 | 0.74 | [0.57; 0.95] | 2.8% |
| Du, 2014 | 0.72 | [0.54; 0.97] | 2.5% |
| Gu, 2016 | 1.10 | [0.67; 1.80] | 1.4% |
| Hashemi, 2016 | 1.09 | [0.59; 2.00] | 1.0% |
| He, 2015 | 1.29 | [1.00; 1.68] | 2.8% |
| He, 2018 | 1.26 | [0.95; 1.66] | 2.7% |
| Hu, 2009 | 0.99 | [0.74; 1.32] | 2.6% |
| Hu, 2013 | 1.05 | [0.85; 1.29] | 3.2% |
| Huang, 2013 | 1.31 | [0.87; 1.97] | 1.8% |
| Jiang, 2016 | 1.10 | [0.90; 1.35] | 3.3% |
| Kim, 2012 | 1.48 | [0.97; 2.24] | 1.7% |
| Kontorovich, 2010 | 0.67 | [0.40; 1.13] | 1.3% |
| Kou, 2014 | 0.88 | [0.57; 1.35] | 1.7% |
| Li, 2016 | 1.04 | [0.80; 1.35] | 2.8% |
| Liu, 2010 | 0.90 | [0.67; 1.21] | 2.5% |
| Liu, 2014 | 0.64 | [0.47; 0.89] | 2.3% |
| Liu, 2015 | 1.39 | [0.86; 2.25] | 1.5% |
| Lv, 2013 | 1.32 | [0.97; 1.79] | 2.5% |
| Miao, 2016 | 1.10 | [0.91; 1.33] | 3.4% |
| Min, 2012 | 1.14 | [0.89; 1.48] | 2.9% |
| Mw Zhang−a, 2012 | 1.12 | [0.86; 1.46] | 2.8% |
| Mw Zhang−b, 2012 | 1.26 | [0.90; 1.77] | 2.2% |
| Ni, 2016 | 1.05 | [0.63; 1.75] | 1.3% |
| Pratedrat, 2015 | 0.92 | [0.52; 1.65] | 1.1% |
| Pu, 2014 | 1.24 | [0.85; 1.80] | 2.0% |
| Ranjbar, 2018 | 1.55 | [0.77; 3.09] | 0.8% |
| Su, 2016 | 1.26 | [0.71; 2.25] | 1.1% |
| Sushma, 2015 | 14.10 | [0.78; 253.71] | 0.1% |
| Tandon, 2017 | 2.47 | [1.14; 5.33] | 0.7% |
| Tian, 2009 | 0.94 | [0.79; 1.11] | 3.6% |
| Tu, 2012 | 1.24 | [0.80; 1.91] | 1.7% |
| Vinci, 2011 | 2.02 | [0.89; 4.57] | 0.6% |
| Vinci, 2013 | 1.59 | [0.82; 3.10] | 0.9% |
| Wang, 2014 | 0.96 | [0.78; 1.18] | 3.3% |
| Wang, 2014 | 1.33 | [0.90; 1.98] | 1.9% |
| Wei, 2014 | 0.85 | [0.71; 1.02] | 3.5% |
| Yan, 2015 | 0.86 | [0.60; 1.23] | 2.1% |
| Yang, 2016 | 0.84 | [0.36; 1.95] | 0.6% |
| Zhang, 2012 | 1.43 | [0.99; 2.06] | 2.1% |
| Filled: Wang, 2014 | 0.75 | [0.51; 1.12] | 1.9% |
| Filled: Liu, 2015 | 0.72 | [0.45; 1.17] | 1.5% |
| Filled: Zhang, 2012 | 0.70 | [0.49; 1.01] | 2.1% |
| Filled: Kim, 2012 | 0.68 | [0.45; 1.03] | 1.7% |
| Filled: Ranjbar, 2018 | 0.65 | [0.33; 1.30] | 0.8% |
| Filled: Vinci, 2013 | 0.63 | [0.32; 1.23] | 0.9% |
| Filled: Vinci, 2011 | 0.50 | [0.22; 1.13] | 0.6% |
| Filled: Tandon, 2017 | 0.41 | [0.19; 0.88] | 0.7% |
| Filled: Sushma, 2015 | 0.07 | [0.00; 1.28] | 0.1% |
| **Random effects model** | **1.00** | **[0.93; 1.09]** | **100.0%** |

Heterogeneity: $I^2 = 49\%$, $\tau^2 = 0.0282$, $p < 0.01$

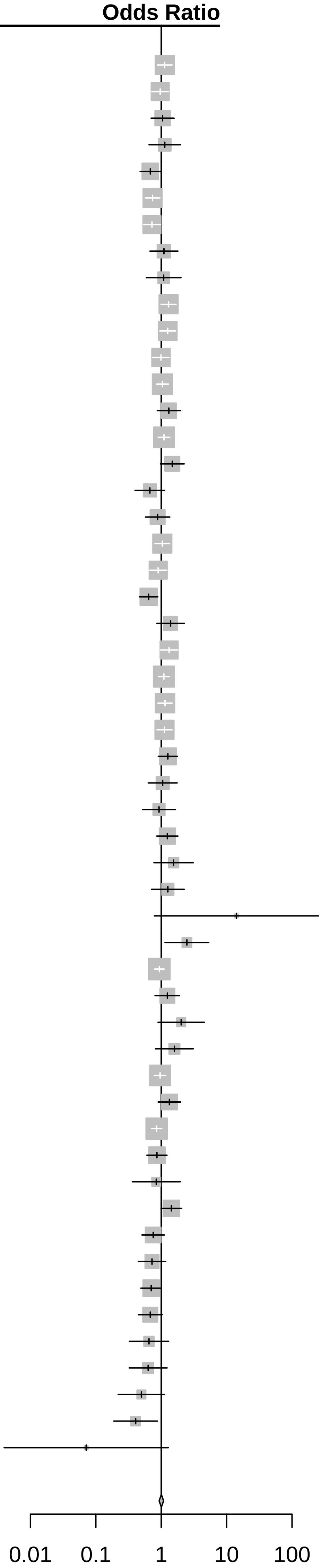

F) **Reccesive model using original studies**

| Studies | OR | 95% − CI | Weights |
|---|---|---|---|
| Ahn, 2012 | 1.13 | [0.87; 1.47] | 3.2% |
| Chu, 2012 | 0.96 | [0.72; 1.30] | 2.8% |
| Chu, 2014 | 1.05 | [0.70; 1.57] | 1.9% |
| Cîmpeanu, 2017 | 1.13 | [0.65; 1.97] | 1.2% |
| Dikeakos, 2014 | 0.68 | [0.48; 0.97] | 2.3% |
| Dong, 2015 | 0.74 | [0.57; 0.95] | 3.3% |
| Du, 2014 | 0.72 | [0.54; 0.97] | 2.8% |
| Gu, 2016 | 1.10 | [0.67; 1.80] | 1.4% |
| Hashemi, 2016 | 1.09 | [0.59; 2.00] | 1.0% |
| He, 2015 | 1.29 | [1.00; 1.68] | 3.2% |
| He, 2018 | 1.26 | [0.95; 1.66] | 3.0% |
| Hu, 2009 | 0.99 | [0.74; 1.32] | 2.9% |
| Hu, 2013 | 1.05 | [0.85; 1.29] | 3.8% |
| Huang, 2013 | 1.31 | [0.87; 1.97] | 1.9% |
| Jiang, 2016 | 1.10 | [0.90; 1.35] | 3.9% |
| Kim, 2012 | 1.48 | [0.97; 2.24] | 1.8% |
| Kontorovich, 2010 | 0.67 | [0.40; 1.13] | 1.3% |
| Kou, 2014 | 0.88 | [0.57; 1.35] | 1.8% |
| Li, 2016 | 1.04 | [0.80; 1.35] | 3.2% |
| Liu, 2010 | 0.90 | [0.67; 1.21] | 2.8% |
| Liu, 2014 | 0.64 | [0.47; 0.89] | 2.6% |
| Liu, 2015 | 1.39 | [0.86; 2.25] | 1.5% |
| Lv, 2013 | 1.32 | [0.97; 1.79] | 2.7% |
| Miao, 2016 | 1.10 | [0.91; 1.33] | 4.1% |
| Min, 2012 | 1.14 | [0.89; 1.48] | 3.3% |
| Mw Zhang−a, 2012 | 1.12 | [0.86; 1.46] | 3.2% |
| Mw Zhang−b, 2012 | 1.26 | [0.90; 1.77] | 2.4% |
| Ni, 2016 | 1.05 | [0.63; 1.75] | 1.4% |
| Pratedrat, 2015 | 0.92 | [0.52; 1.65] | 1.1% |
| Pu, 2014 | 1.24 | [0.85; 1.80] | 2.2% |
| Ranjbar, 2018 | 1.55 | [0.77; 3.09] | 0.8% |
| Su, 2016 | 1.26 | [0.71; 2.25] | 1.1% |
| Sushma, 2015 | 14.10 | [0.78; 253.71] | 0.1% |
| Tandon, 2017 | 2.47 | [1.14; 5.33] | 0.7% |
| Tian, 2009 | 0.94 | [0.79; 1.11] | 4.4% |
| Tu, 2012 | 1.24 | [0.80; 1.91] | 1.8% |
| Vinci, 2011 | 2.02 | [0.89; 4.57] | 0.6% |
| Vinci, 2013 | 1.59 | [0.82; 3.10] | 0.9% |
| Wang, 2014 | 0.96 | [0.78; 1.18] | 4.0% |
| Wang, 2014 | 1.33 | [0.90; 1.98] | 2.0% |
| Wei, 2014 | 0.85 | [0.71; 1.02] | 4.2% |
| Yan, 2015 | 0.86 | [0.60; 1.23] | 2.3% |
| Yang, 2016 | 0.84 | [0.36; 1.95] | 0.6% |
| Zhang, 2012 | 1.43 | [0.99; 2.06] | 2.2% |
| **Random effects model** | **1.05** | **[0.98; 1.13]** | **100.0%** |

Heterogeneity: $I^2 = 42\%$, $\tau^2 = 0.0193$, $p < 0.01$

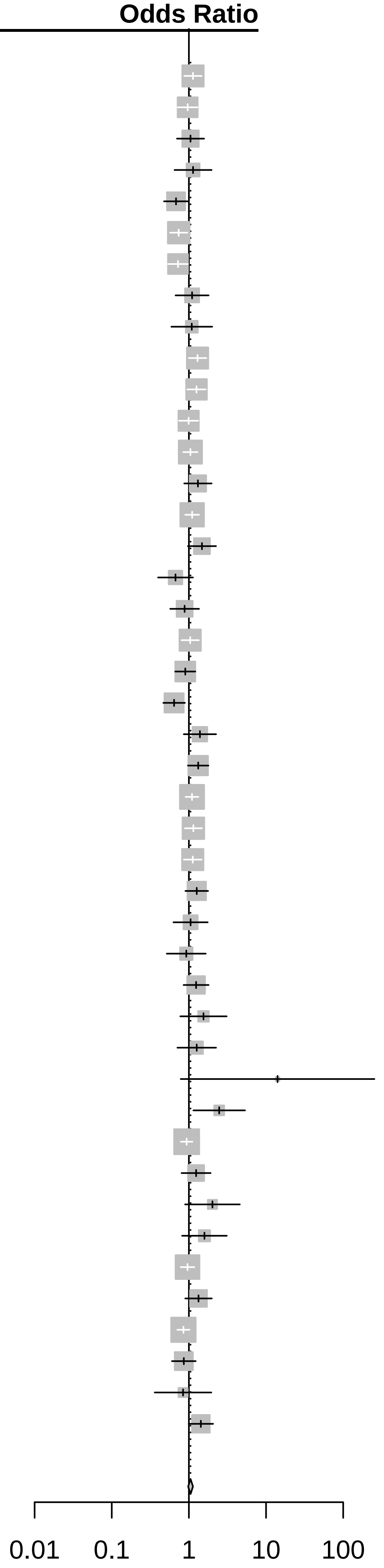

G) Allele contrast - trim & fill

| Studies | OR | 95% − CI | Weights |
|---|---|---|---|
| Ahn, 2012 | 1.06 | [0.87; 1.30] | 2.2% |
| Chu, 2012 | 0.91 | [0.71; 1.17] | 1.9% |
| Chu, 2014 | 1.07 | [0.77; 1.51] | 1.6% |
| Cîmpeanu, 2017 | 0.92 | [0.68; 1.24] | 1.7% |
| Dikeakos, 2014 | 0.70 | [0.53; 0.91] | 1.9% |
| Dong, 2015 | 0.77 | [0.64; 0.92] | 2.2% |
| Du, 2014 | 0.78 | [0.63; 0.97] | 2.1% |
| Gu, 2016 | 1.09 | [0.82; 1.46] | 1.8% |
| Hashemi, 2016 | 1.29 | [0.94; 1.77] | 1.6% |
| He, 2015 | 1.32 | [1.08; 1.61] | 2.2% |
| He, 2018 | 1.13 | [0.90; 1.42] | 2.0% |
| Hu, 2009 | 0.99 | [0.87; 1.12] | 2.5% |
| Hu, 2013 | 1.05 | [0.89; 1.23] | 2.3% |
| Huang, 2013 | 1.20 | [0.90; 1.62] | 1.7% |
| Jiang, 2016 | 1.05 | [0.88; 1.25] | 2.3% |
| Kim, 2012 | 1.30 | [0.94; 1.78] | 1.6% |
| Kontorovich, 2010 | 0.71 | [0.50; 0.99] | 1.6% |
| Kou, 2014 | 0.89 | [0.72; 1.10] | 2.1% |
| Li, 2016 | 1.07 | [0.88; 1.29] | 2.2% |
| Liu, 2010 | 0.97 | [0.85; 1.10] | 2.5% |
| Liu, 2014 | 0.68 | [0.55; 0.85] | 2.1% |
| Liu, 2015 | 1.25 | [0.98; 1.60] | 1.9% |
| Lv, 2013 | 1.27 | [0.99; 1.63] | 1.9% |
| Miao, 2016 | 1.07 | [0.93; 1.24] | 2.4% |
| Min, 2012 | 1.07 | [0.88; 1.29] | 2.2% |
| Mw Zhang−a, 2012 | 1.05 | [0.86; 1.28] | 2.2% |
| Mw Zhang−b, 2012 | 1.09 | [0.85; 1.40] | 1.9% |
| Ni, 2016 | 1.04 | [0.79; 1.36] | 1.8% |
| Pratedrat, 2015 | 0.92 | [0.58; 1.47] | 1.1% |
| Pu, 2014 | 1.10 | [0.82; 1.49] | 1.7% |
| Ranjbar, 2018 | 1.15 | [0.65; 2.01] | 0.9% |
| Su, 2016 | 1.13 | [0.87; 1.45] | 1.9% |
| Sushma, 2015 | 3.28 | [1.64; 6.53] | 0.7% |
| Tandon, 2017 | 2.65 | [1.74; 4.03] | 1.3% |
| Tian, 2009 | 0.95 | [0.83; 1.08] | 2.5% |
| Tu, 2012 | 1.40 | [1.02; 1.92] | 1.6% |
| Vinci, 2011 | 1.38 | [0.93; 2.05] | 1.3% |
| Vinci, 2013 | 1.09 | [0.79; 1.51] | 1.6% |
| Wang, 2014 | 0.91 | [0.78; 1.06] | 2.4% |
| Wang, 2014 | 1.32 | [0.98; 1.76] | 1.8% |
| Wei, 2014 | 0.83 | [0.72; 0.95] | 2.4% |
| Yan, 2015 | 0.90 | [0.72; 1.13] | 2.0% |
| Yang, 2016 | 0.90 | [0.49; 1.64] | 0.8% |
| Zhang, 2012 | 1.25 | [0.95; 1.64] | 1.8% |
| Filled: Liu, 2015 | 0.74 | [0.58; 0.95] | 1.9% |
| Filled: Zhang, 2012 | 0.74 | [0.56; 0.97] | 1.8% |
| Filled: Lv, 2013 | 0.73 | [0.57; 0.93] | 1.9% |
| Filled: Hashemi, 2016 | 0.72 | [0.52; 0.99] | 1.6% |
| Filled: Kim, 2012 | 0.71 | [0.52; 0.98] | 1.6% |
| Filled: Wang, 2014 | 0.70 | [0.53; 0.94] | 1.8% |
| Filled: He, 2015 | 0.70 | [0.57; 0.86] | 2.2% |
| Filled: Vinci, 2011 | 0.67 | [0.45; 0.99] | 1.3% |
| Filled: Tu, 2012 | 0.66 | [0.48; 0.91] | 1.6% |
| Filled: Tandon, 2017 | 0.35 | [0.23; 0.53] | 1.3% |
| Filled: Sushma, 2015 | 0.28 | [0.14; 0.56] | 0.7% |
| **Random effects model** | **0.96** | **[0.89; 1.04]** | **100.0%** |

Heterogeneity: $I^2 = 74\%$, $\tau^2 = 0.0389$, $p < 0.01$

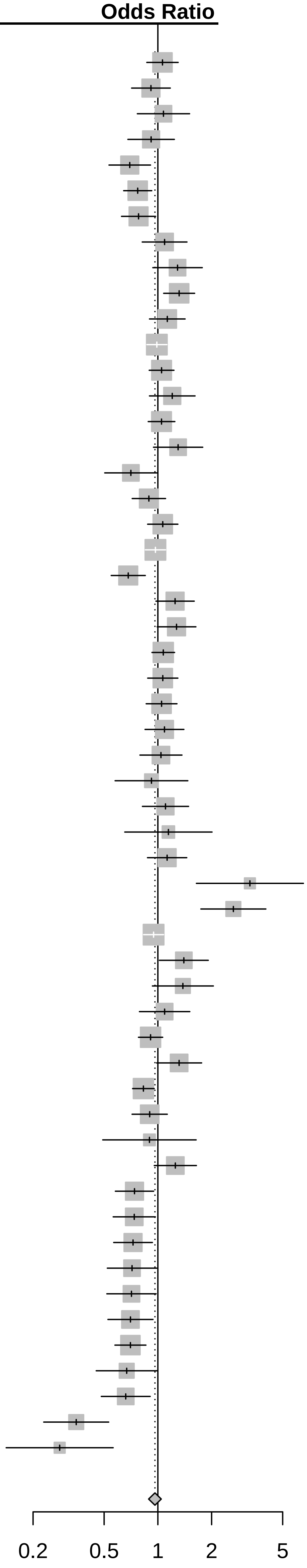

**H) Allele contrast using original studies**

| Studies | OR | 95% − CI | Weights |
|---|---|---|---|
| Ahn, 2012 | 1.06 | [0.87; 1.30] | 2.7% |
| Chu, 2012 | 0.91 | [0.71; 1.17] | 2.3% |
| Chu, 2014 | 1.07 | [0.77; 1.51] | 1.7% |
| Cîmpeanu, 2017 | 0.92 | [0.68; 1.24] | 2.0% |
| Dikeakos, 2014 | 0.70 | [0.53; 0.91] | 2.2% |
| Dong, 2015 | 0.77 | [0.64; 0.92] | 2.9% |
| Du, 2014 | 0.78 | [0.63; 0.97] | 2.6% |
| Gu, 2016 | 1.09 | [0.82; 1.46] | 2.0% |
| Hashemi, 2016 | 1.29 | [0.94; 1.77] | 1.8% |
| He, 2015 | 1.32 | [1.08; 1.61] | 2.7% |
| He, 2018 | 1.13 | [0.90; 1.42] | 2.5% |
| Hu, 2009 | 0.99 | [0.87; 1.12] | 3.3% |
| Hu, 2013 | 1.05 | [0.89; 1.23] | 3.1% |
| Huang, 2013 | 1.20 | [0.90; 1.62] | 2.0% |
| Jiang, 2016 | 1.05 | [0.88; 1.25] | 3.0% |
| Kim, 2012 | 1.30 | [0.94; 1.78] | 1.8% |
| Kontorovich, 2010 | 0.71 | [0.50; 0.99] | 1.7% |
| Kou, 2014 | 0.89 | [0.72; 1.10] | 2.6% |
| Li, 2016 | 1.07 | [0.88; 1.29] | 2.8% |
| Liu, 2010 | 0.97 | [0.85; 1.10] | 3.3% |
| Liu, 2014 | 0.68 | [0.55; 0.85] | 2.6% |
| Liu, 2015 | 1.25 | [0.98; 1.60] | 2.3% |
| Lv, 2013 | 1.27 | [0.99; 1.63] | 2.3% |
| Miao, 2016 | 1.07 | [0.93; 1.24] | 3.2% |
| Min, 2012 | 1.07 | [0.88; 1.29] | 2.8% |
| Mw Zhang−a, 2012 | 1.05 | [0.86; 1.28] | 2.7% |
| Mw Zhang−b, 2012 | 1.09 | [0.85; 1.40] | 2.3% |
| Ni, 2016 | 1.04 | [0.79; 1.36] | 2.2% |
| Pratedrat, 2015 | 0.92 | [0.58; 1.47] | 1.1% |
| Pu, 2014 | 1.10 | [0.82; 1.49] | 2.0% |
| Ranjbar, 2018 | 1.15 | [0.65; 2.01] | 0.9% |
| Su, 2016 | 1.13 | [0.87; 1.45] | 2.3% |
| Sushma, 2015 | 3.28 | [1.64; 6.53] | 0.6% |
| Tandon, 2017 | 2.65 | [1.74; 4.03] | 1.3% |
| Tian, 2009 | 0.95 | [0.83; 1.08] | 3.3% |
| Tu, 2012 | 1.40 | [1.02; 1.92] | 1.9% |
| Vinci, 2011 | 1.38 | [0.93; 2.05] | 1.4% |
| Vinci, 2013 | 1.09 | [0.79; 1.51] | 1.8% |
| Wang, 2014 | 0.91 | [0.78; 1.06] | 3.1% |
| Wang, 2014 | 1.32 | [0.98; 1.76] | 2.0% |
| Wei, 2014 | 0.83 | [0.72; 0.95] | 3.3% |
| Yan, 2015 | 0.90 | [0.72; 1.13] | 2.5% |
| Yang, 2016 | 0.90 | [0.49; 1.64] | 0.8% |
| Zhang, 2012 | 1.25 | [0.95; 1.64] | 2.2% |
| **Random effects model** | **1.04** | **[0.97; 1.12]** | **100.0%** |

Heterogeneity: $I^2 = 64\%$, $\tau^2 = 0.0227$, $p < 0.01$

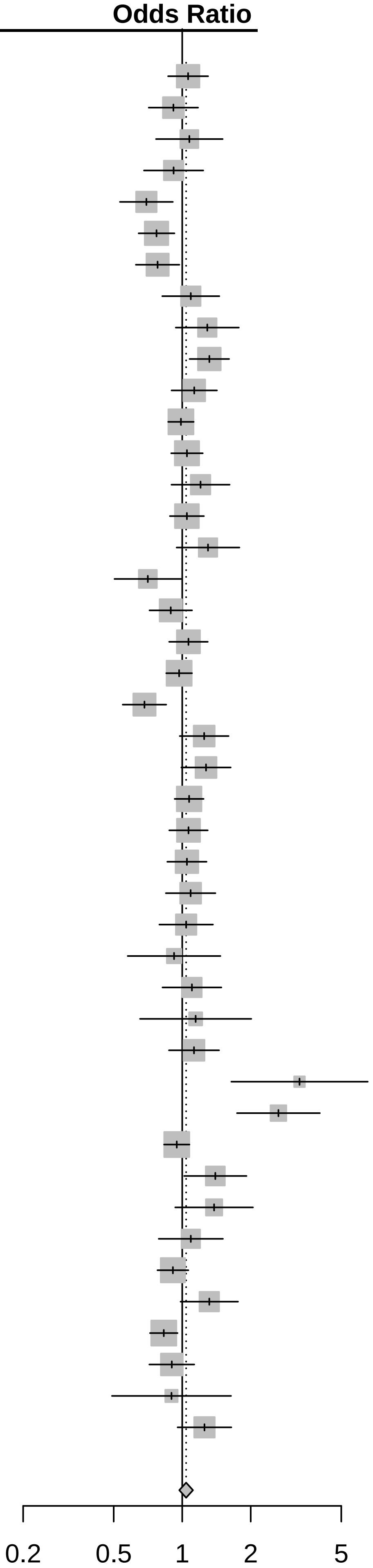

Supplement: Supplementary file 1 [file Data_Sheet_1.ZIP › Supplementary Figures/Suppl. Fig. S4.pdf]
