# Supplementary material for: Association of mir-196a-2 rs11614913 and mir-149 rs2292832 Polymorphisms With Risk of Cancer: An Updated Meta-Analysis
Source: Front Genet. 2019 Mar 15;10:186. doi: 10.3389/fgene.2019.00186 (PMC6429108; doi:10.3389/fgene.2019.00186)

Supplementary Figure S5. Forest plots for meta-analysis of miR-149 rs2292832 sub-grouped according to the broad cancer category. **A)** Homozygote comparison; **B)** Heterozygote comparison; **C)** Dominant model; **D)** Recessive model; **E)** Allele contrast. **Abbreviations:** GI: Gastrointestinal tract cancers; HNC: Head and neck cancers

A) Homozygote comparison

Study	OR	95%−CI	Weight (random)	Weight (fixed)
**GI**				
Ahn, 2012	1.00	[0.63; 1.59]	5.4%	5.5%
Chu, 2014	1.17	[0.59; 2.35]	3.5%	2.3%
Cîmpeanu, 2017	0.99	[0.55; 1.77]	4.2%	3.5%
Dikeakos, 2014	0.44	[0.24; 0.80]	4.1%	4.3%
Gu, 2016	1.17	[0.67; 2.08]	4.4%	3.4%
Jiang, 2016	0.95	[0.66; 1.36]	6.6%	9.4%
Kim, 2012	1.46	[0.70; 3.08]	3.2%	1.8%
Kou, 2014	0.81	[0.51; 1.29]	5.4%	6.3%
Liu, 2014	0.50	[0.33; 0.77]	5.8%	9.2%
Liu, 2015	1.57	[0.93; 2.66]	4.8%	3.4%
Lv, 2013	1.31	[0.81; 2.14]	5.2%	4.4%
Min, 2012	1.01	[0.66; 1.56]	5.7%	6.2%
Mw Zhang−a, 2012	1.00	[0.64; 1.56]	5.6%	5.9%
Mw Zhang−b, 2012	0.99	[0.59; 1.66]	4.9%	4.5%
Pratedrat, 2015	0.87	[0.34; 2.24]	2.2%	1.4%
Pu, 2014	0.95	[0.55; 1.64]	4.6%	4.1%
Ranjbar, 2018	0.61	[0.17; 2.16]	1.4%	1.0%
Su, 2016	1.33	[0.73; 2.42]	4.1%	2.8%
Vinci, 2013	1.47	[0.73; 2.96]	3.4%	2.0%
Wang, 2014	0.71	[0.49; 1.03]	6.5%	10.4%
Wang, 2014	1.96	[0.98; 3.91]	3.5%	1.9%
Yan, 2015	0.82	[0.52; 1.28]	5.6%	6.5%
**Fixed effect model**	**0.95**	**[0.85; 1.06]**	**−−**	**100.0%**
**Random effects model**	**0.97**	**[0.83; 1.13]**	**100.0%**	**−−**
Heterogeneity: $I^2 = 37\%$, $\tau^2 = 0.0416$, $p = 0.04$				
Test for effect in subgroup (fixed effect): $z = -0.88$ ($p = 0.38$)				
Test for effect in subgroup (random effects): $t_{21} = -0.44$ ($p = 0.67$)				
**HNC**				
Chu, 2012	0.77	[0.46; 1.30]	16.1%	14.4%
Huang, 2013	1.36	[0.74; 2.50]	13.6%	8.0%
Liu, 2010	0.90	[0.66; 1.22]	24.2%	37.9%
Miao, 2016	1.12	[0.81; 1.54]	23.7%	31.8%
Sushma, 2015	16.23	[0.90; 292.86]	1.0%	0.2%
Tandon, 2017	2.86	[1.32; 6.21]	10.0%	3.6%
Tu, 2012	2.66	[1.32; 5.33]	11.5%	4.1%
**Fixed effect model**	**1.16**	**[0.97; 1.39]**	**−−**	**100.0%**
**Random effects model**	**1.35**	**[0.77; 2.38]**	**100.0%**	**−−**
Heterogeneity: $I^2 = 69\%$, $\tau^2 = 0.1507$, $p < 0.01$				
Test for effect in subgroup (fixed effect): $z = 1.61$ ($p = 0.11$)				
Test for effect in subgroup (random effects): $t_{21} = 1.31$ ($p = 0.24$)				
**Others**				
Dong, 2015	0.59	[0.40; 0.86]	8.3%	10.4%
Du, 2014	0.64	[0.40; 1.05]	6.8%	6.4%
Hashemi, 2016	1.31	[0.69; 2.48]	5.0%	2.5%
He, 2015	1.91	[1.21; 3.00]	7.2%	4.3%
He, 2018	0.94	[0.60; 1.48]	7.2%	6.0%
Hu, 2009	0.98	[0.73; 1.33]	9.5%	13.3%
Hu, 2013	1.13	[0.79; 1.61]	8.6%	8.8%
Kontorovich, 2010	0.62	[0.36; 1.09]	5.8%	4.8%
Li, 2016	1.23	[0.78; 1.94]	7.2%	5.2%
Ni, 2016	1.09	[0.61; 1.94]	5.6%	3.4%
Tian, 2009	0.90	[0.67; 1.19]	9.8%	15.4%
Vinci, 2011	2.15	[0.91; 5.07]	3.3%	1.1%
Wei, 2014	0.63	[0.46; 0.86]	9.3%	15.2%
Yang, 2016	0.82	[0.19; 3.58]	1.4%	0.6%
Zhang, 2012	1.36	[0.72; 2.56]	5.0%	2.5%
**Fixed effect model**	**0.93**	**[0.83; 1.04]**	**−−**	**100.0%**
**Random effects model**	**0.97**	**[0.79; 1.19]**	**100.0%**	**−−**
Heterogeneity: $I^2 = 59\%$, $\tau^2 = 0.0740$, $p < 0.01$				
Test for effect in subgroup (fixed effect): $z = -1.25$ ($p = 0.21$)				
Test for effect in subgroup (random effects): $t_{21} = -0.32$ ($p = 0.75$)				

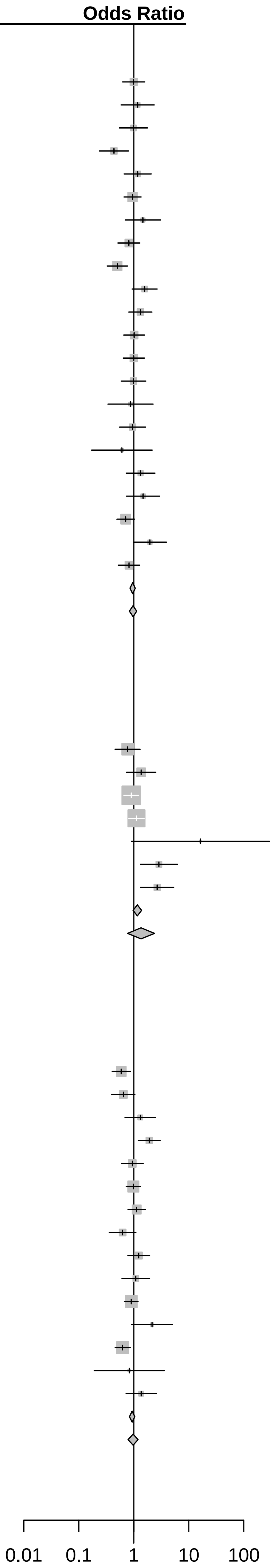

B) Heterozygote comparison

Study	OR	95%−CI	Weight (random)	Weight (fixed)
**GI**				
Ahn, 2012	0.86	[0.53; 1.38]	4.8%	4.5%
Chu, 2014	1.17	[0.54; 2.54]	2.2%	1.5%
Cîmpeanu, 2017	0.74	[0.47; 1.14]	5.3%	5.6%
Dikeakos, 2014	0.58	[0.31; 1.07]	3.3%	3.1%
Gu, 2016	1.12	[0.70; 1.79]	4.9%	4.0%
Jiang, 2016	0.82	[0.55; 1.22]	6.0%	6.5%
Kim, 2012	0.99	[0.47; 2.09]	2.4%	1.7%
Kou, 2014	0.86	[0.63; 1.18]	7.8%	10.2%
Liu, 2014	0.69	[0.46; 1.04]	5.8%	6.8%
Liu, 2015	1.24	[0.86; 1.80]	6.5%	6.1%
Lv, 2013	0.99	[0.57; 1.73]	3.9%	3.1%
Min, 2012	0.86	[0.55; 1.33]	5.3%	5.2%
Mw Zhang−a, 2012	0.87	[0.55; 1.35]	5.2%	5.1%
Mw Zhang−b, 2012	0.72	[0.43; 1.21]	4.2%	4.1%
Pratedrat, 2015	0.92	[0.33; 2.60]	1.3%	0.9%
Pu, 2014	0.66	[0.34; 1.25]	3.0%	2.7%
Ranjbar, 2018	0.29	[0.07; 1.15]	0.8%	0.9%
Su, 2016	1.10	[0.78; 1.57]	7.0%	7.3%
Vinci, 2013	0.84	[0.53; 1.33]	5.0%	4.9%
Wang, 2014	0.69	[0.48; 1.01]	6.5%	8.2%
Wang, 2014	1.61	[0.81; 3.18]	2.8%	1.7%
Yan, 2015	0.93	[0.62; 1.40]	5.9%	5.9%
**Fixed effect model**	**0.88**	**[0.80; 0.97]**	**−−**	**100.0%**
**Random effects model**	**0.88**	**[0.79; 0.97]**	**100.0%**	**−−**
Heterogeneity: $I^2 = 0\%$, $\tau^2 = 0$, $p = 0.57$				
Test for effect in subgroup (fixed effect): $z = -2.53$ ($p = 0.01$)				
Test for effect in subgroup (random effects): $t_{21} = -2.66$ ($p = 0.01$)				
**HNC**				
Chu, 2012	0.74	[0.41; 1.32]	10.3%	6.6%
Huang, 2013	1.05	[0.57; 1.92]	9.8%	5.2%
Liu, 2010	1.00	[0.84; 1.19]	34.3%	62.9%
Miao, 2016	1.02	[0.74; 1.42]	21.8%	17.7%
Sushma, 2015	2.29	[1.06; 4.94]	6.6%	2.2%
Tandon, 2017	2.87	[1.53; 5.38]	9.2%	3.1%
Tu, 2012	2.60	[1.30; 5.20]	7.9%	2.3%
**Fixed effect model**	**1.11**	**[0.97; 1.27]**	**−−**	**100.0%**
**Random effects model**	**1.34**	**[0.82; 2.19]**	**100.0%**	**−−**
Heterogeneity: $I^2 = 72\%$, $\tau^2 = 0.1266$, $p < 0.01$				
Test for effect in subgroup (fixed effect): $z = 1.56$ ($p = 0.12$)				
Test for effect in subgroup (random effects): $t_{21} = 1.48$ ($p = 0.19$)				
**Others**				
Dong, 2015	0.75	[0.52; 1.08]	8.0%	7.8%
Du, 2014	0.87	[0.53; 1.41]	5.7%	4.3%
Hashemi, 2016	1.56	[0.99; 2.48]	6.1%	3.5%
He, 2015	1.62	[1.02; 2.57]	6.1%	3.5%
He, 2018	0.66	[0.40; 1.12]	5.2%	4.1%
Hu, 2009	0.98	[0.82; 1.17]	14.2%	29.1%
Hu, 2013	1.10	[0.76; 1.57]	8.3%	6.9%
Kontorovich, 2010	0.81	[0.45; 1.46]	4.3%	3.0%
Li, 2016	1.23	[0.78; 1.93]	6.2%	4.1%
Ni, 2016	1.05	[0.68; 1.62]	6.7%	4.9%
Tian, 2009	0.95	[0.71; 1.26]	10.4%	11.7%
Vinci, 2011	1.14	[0.65; 2.00]	4.6%	2.8%
Wei, 2014	0.68	[0.50; 0.94]	9.4%	11.2%
Yang, 2016	0.98	[0.24; 4.07]	0.9%	0.5%
Zhang, 2012	0.94	[0.50; 1.77]	3.8%	2.4%
**Fixed effect model**	**0.97**	**[0.88; 1.07]**	**−−**	**100.0%**
**Random effects model**	**0.98**	**[0.85; 1.12]**	**100.0%**	**−−**
Heterogeneity: $I^2 = 30\%$, $\tau^2 = 0.0175$, $p = 0.13$				
Test for effect in subgroup (fixed effect): $z = -0.60$ ($p = 0.55$)				
Test for effect in subgroup (random effects): $t_{21} = -0.37$ ($p = 0.72$)				

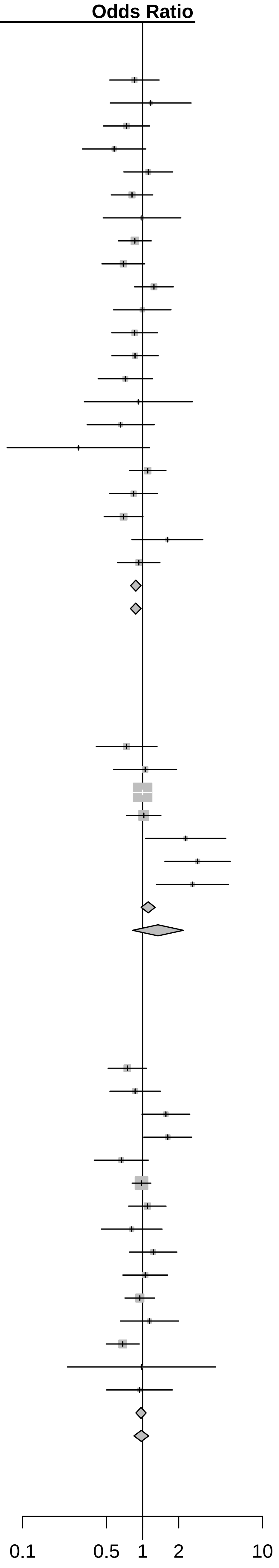

C) **Dominant model**

Study	OR	95%−CI	Weight (random)	Weight (fixed)
**GI**				
Ahn, 2012	0.93	[0.59; 1.46]	4.8%	4.3%
Chu, 2014	1.17	[0.59; 2.33]	2.8%	1.7%
Cîmpeanu, 2017	0.80	[0.53; 1.20]	5.3%	5.7%
Dikeakos, 2014	0.50	[0.28; 0.89]	3.5%	3.2%
Gu, 2016	1.13	[0.73; 1.76]	4.9%	4.0%
Jiang, 2016	0.92	[0.64; 1.31]	6.0%	6.9%
Kim, 2012	1.21	[0.59; 2.46]	2.7%	1.5%
Kou, 2014	0.85	[0.63; 1.15]	6.8%	10.1%
Liu, 2014	0.60	[0.41; 0.87]	5.6%	7.6%
Liu, 2015	1.31	[0.92; 1.87]	6.0%	5.9%
Lv, 2013	1.23	[0.76; 1.99]	4.5%	3.3%
Min, 2012	0.94	[0.62; 1.42]	5.2%	5.0%
Mw Zhang−a, 2012	0.93	[0.61; 1.42]	5.1%	4.8%
Mw Zhang−b, 2012	0.85	[0.52; 1.38]	4.4%	3.8%
Pratedrat, 2015	0.88	[0.35; 2.24]	1.7%	1.0%
Pu, 2014	0.88	[0.51; 1.50]	3.9%	3.0%
Ranjbar, 2018	0.49	[0.14; 1.72]	1.0%	0.8%
Su, 2016	1.14	[0.81; 1.59]	6.3%	7.0%
Vinci, 2013	0.96	[0.63; 1.47]	5.0%	4.7%
Wang, 2014	0.70	[0.49; 1.00]	6.0%	8.0%
Wang, 2014	1.76	[0.92; 3.39]	3.0%	1.6%
Yan, 2015	0.89	[0.61; 1.30]	5.6%	6.1%
**Fixed effect model**	**0.92**	**[0.84; 1.01]**	**--**	**100.0%**
**Random effects model**	**0.92**	**[0.82; 1.03]**	**100.0%**	**--**
Heterogeneity: $I^2 = 19\%$, $\tau^2 = 0.0121$, $p = 0.21$				
Test for effect in subgroup (fixed effect): $z = -1.66$ ($p = 0.10$)				
Test for effect in subgroup (random effects): $t_{21} = -1.47$ ($p = 0.16$)				
**HNC**				
Chu, 2012	0.76	[0.45; 1.28]	12.3%	7.3%
Huang, 2013	1.19	[0.67; 2.09]	11.1%	4.9%
Liu, 2010	0.98	[0.83; 1.16]	27.0%	62.2%
Miao, 2016	1.07	[0.79; 1.46]	20.3%	17.4%
Sushma, 2015	2.92	[1.39; 6.14]	7.6%	1.9%
Tandon, 2017	2.87	[1.72; 4.78]	12.6%	4.0%
Tu, 2012	2.63	[1.37; 5.06]	9.1%	2.3%
**Fixed effect model**	**1.14**	**[1.01; 1.30]**	**--**	**100.0%**
**Random effects model**	**1.45**	**[0.87; 2.43]**	**100.0%**	**--**
Heterogeneity: $I^2 = 80\%$, $\tau^2 = 0.1691$, $p < 0.01$				
Test for effect in subgroup (fixed effect): $z = 2.06$ ($p = 0.04$)				
Test for effect in subgroup (random effects): $t_{21} = 1.76$ ($p = 0.13$)				
**Others**				
Dong, 2015	0.67	[0.47; 0.95]	7.8%	8.0%
Du, 2014	0.75	[0.47; 1.18]	6.0%	4.6%
Hashemi, 2016	1.49	[0.98; 2.27]	6.6%	3.8%
He, 2015	1.77	[1.14; 2.74]	6.4%	3.3%
He, 2018	0.88	[0.56; 1.37]	6.2%	4.3%
Hu, 2009	0.98	[0.82; 1.16]	11.3%	28.1%
Hu, 2013	1.11	[0.79; 1.56]	8.0%	6.7%
Kontorovich, 2010	0.71	[0.44; 1.13]	5.9%	4.5%
Li, 2016	1.23	[0.80; 1.90]	6.4%	3.9%
Ni, 2016	1.06	[0.70; 1.60]	6.7%	4.7%
Tian, 2009	0.92	[0.70; 1.21]	9.3%	11.6%
Vinci, 2011	1.32	[0.78; 2.22]	5.2%	2.6%
Wei, 2014	0.65	[0.48; 0.88]	8.7%	11.2%
Yang, 2016	0.91	[0.23; 3.60]	1.1%	0.4%
Zhang, 2012	1.13	[0.62; 2.06]	4.3%	2.1%
**Fixed effect model**	**0.96**	**[0.88; 1.06]**	**--**	**100.0%**
**Random effects model**	**0.98**	**[0.83; 1.15]**	**100.0%**	**--**
Heterogeneity: $I^2 = 52\%$, $\tau^2 = 0.0381$, $p = 0.01$				
Test for effect in subgroup (fixed effect): $z = -0.78$ ($p = 0.44$)				
Test for effect in subgroup (random effects): $t_{21} = -0.26$ ($p = 0.80$)				

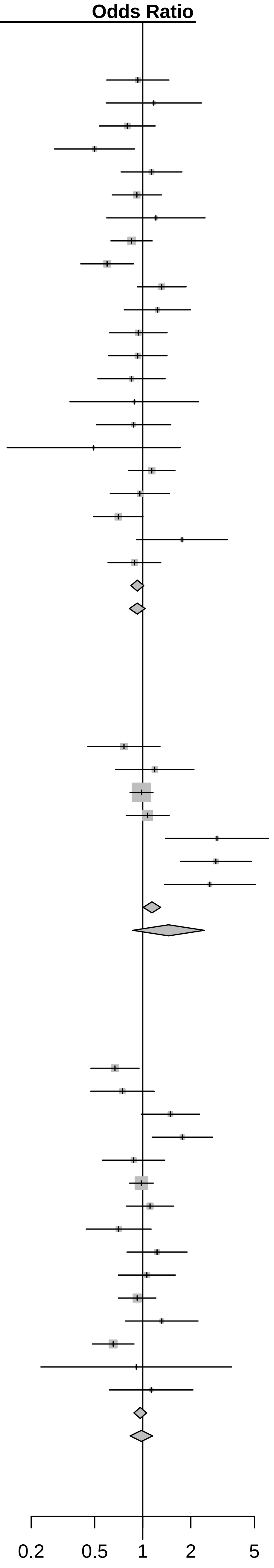

D) recessive model

Study	OR	95%−CI	Weight (random)	Weight (fixed)
**GI**				
Ahn, 2012	1.13	[0.87; 1.47]	6.8%	7.5%
Chu, 2014	1.05	[0.70; 1.57]	4.0%	3.2%
Cîmpeanu, 2017	1.13	[0.65; 1.97]	2.5%	1.6%
Dikeakos, 2014	0.68	[0.48; 0.97]	4.7%	5.1%
Gu, 2016	1.10	[0.67; 1.80]	3.0%	2.1%
Jiang, 2016	1.10	[0.90; 1.35]	8.2%	12.2%
Kim, 2012	1.48	[0.97; 2.24]	3.9%	2.5%
Kou, 2014	0.88	[0.57; 1.35]	3.7%	3.2%
Liu, 2014	0.64	[0.47; 0.89]	5.4%	6.5%
Liu, 2015	1.39	[0.86; 2.25]	3.1%	2.0%
Lv, 2013	1.32	[0.97; 1.79]	5.7%	5.1%
Min, 2012	1.14	[0.89; 1.48]	6.9%	7.8%
Mw Zhang−a, 2012	1.12	[0.86; 1.46]	6.6%	7.2%
Mw Zhang−b, 2012	1.26	[0.90; 1.77]	5.1%	4.2%
Pratedrat, 2015	0.92	[0.52; 1.65]	2.3%	1.7%
Pu, 2014	1.24	[0.85; 1.80]	4.5%	3.6%
Ranjbar, 2018	1.55	[0.77; 3.09]	1.7%	0.9%
Su, 2016	1.26	[0.71; 2.25]	2.4%	1.4%
Vinci, 2013	1.59	[0.82; 3.10]	1.9%	1.0%
Wang, 2014	0.96	[0.78; 1.18]	8.3%	13.4%
Wang, 2014	1.33	[0.90; 1.98]	4.2%	3.0%
Yan, 2015	0.86	[0.60; 1.23]	4.8%	4.7%
**Fixed effect model**	**1.07**	**[1.00; 1.15]**	**--**	**100.0%**
**Random effects model**	**1.08**	**[0.98; 1.19]**	**100.0%**	**--**
Heterogeneity: $I^2 = 31\%$, $\tau^2 = 0.0143$, $p = 0.08$				
Test for effect in subgroup (fixed effect): $z = 1.86$ ($p = 0.06$)				
Test for effect in subgroup (random effects): $t_{21} = 1.60$ ($p = 0.12$)				
**HNC**				
Chu, 2012	0.96	[0.72; 1.30]	19.9%	19.0%
Huang, 2013	1.31	[0.87; 1.97]	13.5%	8.6%
Liu, 2010	0.90	[0.67; 1.21]	19.8%	19.4%
Miao, 2016	1.10	[0.91; 1.33]	29.3%	43.0%
Sushma, 2015	14.10	[0.78; 253.71]	0.4%	0.1%
Tandon, 2017	2.47	[1.14; 5.33]	4.9%	1.9%
Tu, 2012	1.24	[0.80; 1.91]	12.4%	8.0%
**Fixed effect model**	**1.10**	**[0.97; 1.25]**	**--**	**100.0%**
**Random effects model**	**1.13**	**[0.85; 1.51]**	**100.0%**	**--**
Heterogeneity: $I^2 = 44\%$, $\tau^2 = 0.0270$, $p = 0.10$				
Test for effect in subgroup (fixed effect): $z = 1.52$ ($p = 0.13$)				
Test for effect in subgroup (random effects): $t_{21} = 1.06$ ($p = 0.33$)				
**Others**				
Dong, 2015	0.74	[0.57; 0.95]	8.6%	9.3%
Du, 2014	0.72	[0.54; 0.97]	7.4%	7.1%
Hashemi, 2016	1.09	[0.59; 2.00]	2.7%	1.4%
He, 2015	1.29	[1.00; 1.68]	8.4%	6.7%
He, 2018	1.26	[0.95; 1.66]	7.9%	6.1%
Hu, 2009	0.99	[0.74; 1.32]	7.7%	6.4%
Hu, 2013	1.05	[0.85; 1.29]	10.1%	11.3%
Kontorovich, 2010	0.67	[0.40; 1.13]	3.5%	2.3%
Li, 2016	1.04	[0.80; 1.35]	8.5%	7.7%
Ni, 2016	1.05	[0.63; 1.75]	3.6%	1.9%
Tian, 2009	0.94	[0.79; 1.11]	11.6%	18.2%
Vinci, 2011	2.02	[0.89; 4.57]	1.6%	0.6%
Wei, 2014	0.85	[0.71; 1.02]	11.1%	16.9%
Yang, 2016	0.84	[0.36; 1.95]	1.5%	0.8%
Zhang, 2012	1.43	[0.99; 2.06]	5.8%	3.3%
**Fixed effect model**	**0.97**	**[0.91; 1.05]**	**--**	**100.0%**
**Random effects model**	**0.99**	**[0.87; 1.13]**	**100.0%**	**--**
Heterogeneity: $I^2 = 51\%$, $\tau^2 = 0.0231$, $p = 0.01$				
Test for effect in subgroup (fixed effect): $z = -0.69$ ($p = 0.49$)				
Test for effect in subgroup (random effects): $t_{21} = -0.14$ ($p = 0.89$)				

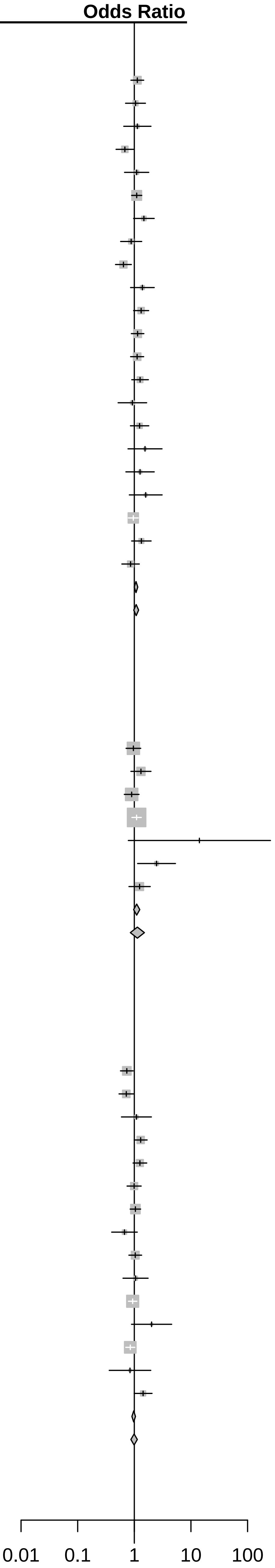

E) Allele contrast

Study	OR	95%−CI	Weight (random)	Weight (fixed)
**GI**				
Ahn, 2012	1.06	[0.87; 1.30]	5.6%	6.4%
Chu, 2014	1.07	[0.77; 1.51]	3.5%	2.3%
Cîmpeanu, 2017	0.92	[0.68; 1.24]	4.0%	3.1%
Dikeakos, 2014	0.70	[0.53; 0.91]	4.5%	4.4%
Gu, 2016	1.09	[0.82; 1.46]	4.2%	3.1%
Jiang, 2016	1.05	[0.88; 1.25]	6.1%	8.9%
Kim, 2012	1.30	[0.94; 1.78]	3.8%	2.4%
Kou, 2014	0.89	[0.72; 1.10]	5.3%	6.2%
Liu, 2014	0.68	[0.55; 0.85]	5.2%	6.7%
Liu, 2015	1.25	[0.98; 1.60]	4.8%	3.9%
Lv, 2013	1.27	[0.99; 1.63]	4.8%	3.9%
Min, 2012	1.07	[0.88; 1.29]	5.7%	6.9%
Mw Zhang−a, 2012	1.05	[0.86; 1.28]	5.6%	6.7%
Mw Zhang−b, 2012	1.09	[0.85; 1.40]	4.7%	4.1%
Pratedrat, 2015	0.92	[0.58; 1.47]	2.3%	1.3%
Pu, 2014	1.10	[0.82; 1.49]	4.0%	2.9%
Ranjbar, 2018	1.15	[0.65; 2.01]	1.8%	0.8%
Su, 2016	1.13	[0.87; 1.45]	4.7%	3.9%
Vinci, 2013	1.09	[0.79; 1.51]	3.7%	2.4%
Wang, 2014	0.91	[0.78; 1.06]	6.4%	11.5%
Wang, 2014	1.32	[0.98; 1.76]	4.1%	2.8%
Yan, 2015	0.90	[0.72; 1.13]	5.1%	5.5%
**Fixed effect model**	**1.01**	**[0.96; 1.06]**	**−−**	**100.0%**
**Random effects model**	**1.02**	**[0.94; 1.10]**	**100.0%**	**−−**
Heterogeneity: $I^2 = 46\%$, $\tau^2 = 0.0133$, $p = 0.01$				
Test for effect in subgroup (fixed effect): $z = 0.41$ ($p = 0.68$)				
Test for effect in subgroup (random effects): $t_{21} = 0.52$ ($p = 0.61$)				
**HNC**				
Chu, 2012	0.91	[0.71; 1.17]	15.9%	11.5%
Huang, 2013	1.20	[0.90; 1.62]	13.6%	7.2%
Liu, 2010	0.97	[0.85; 1.10]	22.7%	41.0%
Miao, 2016	1.07	[0.93; 1.24]	21.8%	31.1%
Sushma, 2015	3.28	[1.64; 6.53]	4.2%	0.9%
Tandon, 2017	2.65	[1.74; 4.03]	9.0%	2.5%
Tu, 2012	1.40	[1.02; 1.92]	12.7%	5.7%
**Fixed effect model**	**1.10**	**[1.01; 1.19]**	**−−**	**100.0%**
**Random effects model**	**1.30**	**[0.87; 1.95]**	**100.0%**	**−−**
Heterogeneity: $I^2 = 83\%$, $\tau^2 = 0.0683$, $p < 0.01$				
Test for effect in subgroup (fixed effect): $z = 2.30$ ($p = 0.02$)				
Test for effect in subgroup (random effects): $t_{21} = 1.61$ ($p = 0.16$)				
**Others**				
Dong, 2015	0.77	[0.64; 0.92]	7.9%	8.7%
Du, 2014	0.78	[0.63; 0.97]	7.0%	6.0%
Hashemi, 2016	1.29	[0.94; 1.77]	5.0%	2.2%
He, 2015	1.32	[1.08; 1.61]	7.5%	5.6%
He, 2018	1.13	[0.90; 1.42]	6.8%	4.6%
Hu, 2009	0.99	[0.87; 1.12]	9.1%	15.4%
Hu, 2013	1.05	[0.89; 1.23]	8.4%	9.7%
Kontorovich, 2010	0.71	[0.50; 0.99]	4.7%	2.6%
Li, 2016	1.07	[0.88; 1.29]	7.6%	6.5%
Ni, 2016	1.04	[0.79; 1.36]	5.9%	3.4%
Tian, 2009	0.95	[0.83; 1.08]	9.2%	15.8%
Vinci, 2011	1.38	[0.93; 2.05]	3.9%	1.4%
Wei, 2014	0.83	[0.72; 0.95]	8.9%	14.3%
Yang, 2016	0.90	[0.49; 1.64]	2.1%	0.7%
Zhang, 2012	1.25	[0.95; 1.64]	5.9%	3.1%
**Fixed effect model**	**0.98**	**[0.93; 1.03]**	**−−**	**100.0%**
**Random effects model**	**1.00**	**[0.89; 1.11]**	**100.0%**	**−−**
Heterogeneity: $I^2 = 66\%$, $\tau^2 = 0.0199$, $p < 0.01$				
Test for effect in subgroup (fixed effect): $z = −0.91$ ($p = 0.36$)				
Test for effect in subgroup (random effects): $t_{21} = −0.04$ ($p = 0.97$)				

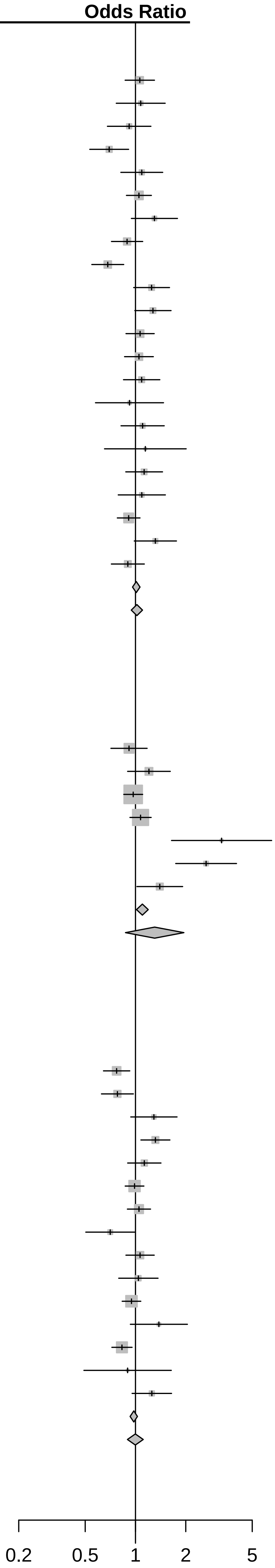

Supplement: Supplementary file 1 [file Data_Sheet_1.ZIP › Supplementary Figures/Suppl. Fig. S5.pdf]
