# Supplementary material for: Association of mir-196a-2 rs11614913 and mir-149 rs2292832 Polymorphisms With Risk of Cancer: An Updated Meta-Analysis
Source: Front Genet. 2019 Mar 15;10:186. doi: 10.3389/fgene.2019.00186 (PMC6429108; doi:10.3389/fgene.2019.00186)

**Supplementary Figure S6.** Forest plots for meta-analysis of miR-149 rs2292832 sub-grouped according to cancer types. **A)** Homozygote comparison; **B)** Heterozygote comparison; **C)** Dominant comparison; **D)** Recessive comparison; **E)** Allele contrast.
**Abbreviations:** BC: Breast cancer; HCC: Hepatocellular cancers; GC: Gastric cancer; CRC: Colorectal cancer; LC: Lung cancer

**A) Homozygote comparison**

| Study | OR | 95%−CI | Weight (random) | Weight (fixed) |
|---|---|---|---|---|
| **Others** | | | | |
| He, 2018 | 0.94 | [0.60; 1.48] | 7.9% | 6.8% |
| Tandon, 2017 | 2.86 | [1.32; 6.21] | 4.3% | 1.4% |
| Hashemi, 2016 | 1.31 | [0.69; 2.48] | 5.5% | 2.9% |
| Miao, 2016 | 1.12 | [0.81; 1.54] | 10.1% | 12.6% |
| Ni, 2016 | 1.09 | [0.61; 1.94] | 6.2% | 3.9% |
| Yang, 2016 | 0.82 | [0.19; 3.58] | 1.5% | 0.7% |
| Dong, 2015 | 0.59 | [0.40; 0.86] | 9.1% | 11.8% |
| Sushma, 2015 | 16.23 | [0.90; 292.86] | 0.4% | 0.1% |
| Du, 2014 | 0.64 | [0.40; 1.05] | 7.4% | 7.2% |
| Wei, 2014 | 0.63 | [0.46; 0.86] | 10.2% | 17.3% |
| Hu, 2013 | 1.13 | [0.79; 1.61] | 9.5% | 9.9% |
| Huang, 2013 | 1.36 | [0.74; 2.50] | 5.8% | 3.2% |
| Chu, 2012 | 0.77 | [0.46; 1.30] | 6.9% | 5.7% |
| Tu, 2012 | 2.66 | [1.32; 5.33] | 4.9% | 1.6% |
| Liu, 2010 | 0.90 | [0.66; 1.22] | 10.3% | 15.0% |
| **Fixed effect model** | **0.94** | **[0.84; 1.06]** | **--** | **100.0%** |
| **Random effects model** | **1.02** | **[0.78; 1.34]** | **100.0%** | **--** |
| Heterogeneity: $I^2 = 65\%$, $\tau^2 = 0.1082$, $p < 0.01$ | | | | |
| Test for effect in subgroup (fixed effect): $z = -0.96$ ($p = 0.34$) | | | | |
| Test for effect in subgroup (random effects): $t_{14} = 0.16$ ($p = 0.88$) | | | | |
| **CRC** | | | | |
| Ranjbar, 2018 | 0.61 | [0.17; 2.16] | 6.4% | 5.0% |
| Lv, 2013 | 1.31 | [0.81; 2.14] | 24.3% | 22.7% |
| Vinci, 2013 | 1.47 | [0.73; 2.96] | 16.1% | 10.3% |
| Min, 2012 | 1.01 | [0.66; 1.56] | 26.9% | 31.7% |
| Mw Zhang−a, 2012 | 1.00 | [0.64; 1.56] | 26.2% | 30.2% |
| **Fixed effect model** | **1.10** | **[0.87; 1.40]** | **--** | **100.0%** |
| **Random effects model** | **1.10** | **[0.85; 1.43]** | **100.0%** | **--** |
| Heterogeneity: $I^2 = 0\%$, $\tau^2 = 0$, $p = 0.67$ | | | | |
| Test for effect in subgroup (fixed effect): $z = 0.81$ ($p = 0.42$) | | | | |
| Test for effect in subgroup (random effects): $t_{14} = 1.05$ ($p = 0.35$) | | | | |
| **GC** | | | | |
| Cîmpeanu, 2017 | 0.99 | [0.55; 1.77] | 11.1% | 9.3% |
| Gu, 2016 | 1.17 | [0.67; 2.08] | 11.5% | 9.0% |
| Jiang, 2016 | 0.95 | [0.66; 1.36] | 17.3% | 25.2% |
| Su, 2016 | 1.33 | [0.73; 2.42] | 10.7% | 7.5% |
| Dikeakos, 2014 | 0.44 | [0.24; 0.80] | 10.6% | 11.6% |
| Pu, 2014 | 0.95 | [0.55; 1.64] | 12.0% | 10.9% |
| Ahn, 2012 | 1.00 | [0.63; 1.59] | 14.1% | 14.6% |
| Mw Zhang−b, 2012 | 0.99 | [0.59; 1.66] | 12.7% | 11.9% |
| **Fixed effect model** | **0.95** | **[0.80; 1.14]** | **--** | **100.0%** |
| **Random effects model** | **0.95** | **[0.74; 1.20]** | **100.0%** | **--** |
| Heterogeneity: $I^2 = 14\%$, $\tau^2 = 0.0109$, $p = 0.32$ | | | | |
| Test for effect in subgroup (fixed effect): $z = -0.52$ ($p = 0.60$) | | | | |
| Test for effect in subgroup (random effects): $t_{14} = -0.54$ ($p = 0.61$) | | | | |
| **LC** | | | | |
| Li, 2016 | 1.23 | [0.78; 1.94] | 35.3% | 23.8% |
| Vinci, 2011 | 2.15 | [0.91; 5.07] | 16.5% | 5.1% |
| Tian, 2009 | 0.90 | [0.67; 1.19] | 48.2% | 71.0% |
| **Fixed effect model** | **1.04** | **[0.82; 1.31]** | **--** | **100.0%** |
| **Random effects model** | **1.16** | **[0.45; 2.97]** | **100.0%** | **--** |
| Heterogeneity: $I^2 = 53\%$, $\tau^2 = 0.0670$, $p = 0.12$ | | | | |
| Test for effect in subgroup (fixed effect): $z = 0.33$ ($p = 0.74$) | | | | |
| Test for effect in subgroup (random effects): $t_{14} = 0.67$ ($p = 0.57$) | | | | |
| **BC** | | | | |
| He, 2015 | 1.91 | [1.21; 3.00] | 26.0% | 17.2% |
| Zhang, 2012 | 1.36 | [0.72; 2.56] | 18.3% | 10.2% |
| Kontorovich, 2010 | 0.62 | [0.36; 1.09] | 21.2% | 19.3% |
| Hu, 2009 | 0.98 | [0.73; 1.33] | 34.6% | 53.3% |
| **Fixed effect model** | **1.11** | **[0.90; 1.37]** | **--** | **100.0%** |
| **Random effects model** | **1.13** | **[0.53; 2.37]** | **100.0%** | **--** |
| Heterogeneity: $I^2 = 72\%$, $\tau^2 = 0.1396$, $p = 0.01$ | | | | |
| Test for effect in subgroup (fixed effect): $z = 0.96$ ($p = 0.34$) | | | | |
| Test for effect in subgroup (random effects): $t_{14} = 0.51$ ($p = 0.65$) | | | | |
| **HCC** | | | | |
| Liu, 2015 | 1.57 | [0.93; 2.66] | 11.8% | 7.9% |
| Pratedrat, 2015 | 0.87 | [0.34; 2.24] | 5.5% | 3.3% |
| Yan, 2015 | 0.82 | [0.52; 1.28] | 13.8% | 15.0% |
| Chu, 2014 | 1.17 | [0.59; 2.35] | 8.6% | 5.4% |
| Kou, 2014 | 0.81 | [0.51; 1.29] | 13.4% | 14.5% |
| Liu, 2014 | 0.50 | [0.33; 0.77] | 14.4% | 21.3% |
| Wang, 2014 | 0.71 | [0.49; 1.03] | 16.1% | 24.1% |
| Wang, 2014 | 1.96 | [0.98; 3.91] | 8.6% | 4.4% |
| Kim, 2012 | 1.46 | [0.70; 3.08] | 7.8% | 4.2% |
| **Fixed effect model** | **0.88** | **[0.74; 1.05]** | **--** | **100.0%** |
| **Random effects model** | **0.96** | **[0.68; 1.34]** | **100.0%** | **--** |
| Heterogeneity: $I^2 = 61\%$, $\tau^2 = 0.1143$, $p < 0.01$ | | | | |
| Test for effect in subgroup (fixed effect): $z = -1.45$ ($p = 0.15$) | | | | |
| Test for effect in subgroup (random effects): $t_{14} = -0.30$ ($p = 0.77$) | | | | |

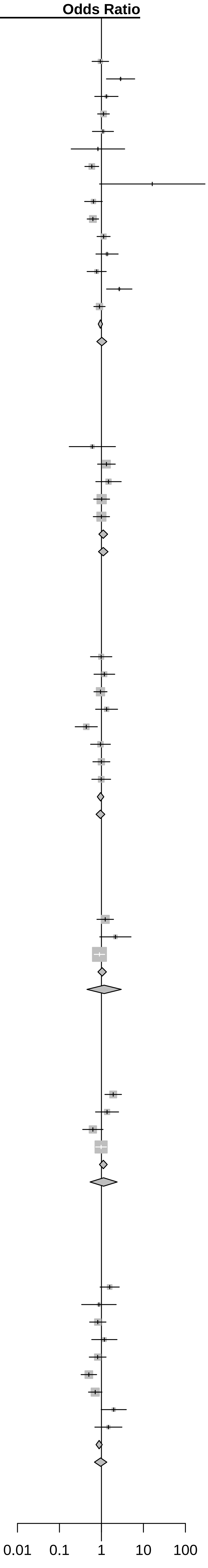

**B) Heterozygote comparison**

| Study | OR | 95%−CI | Weight (random) | Weight (fixed) |
|---|---|---|---|---|
| **Others** | | | | |
| He, 2018 | 0.66 | [0.40; 1.12] | 5.6% | 4.5% |
| Tandon, 2017 | 2.87 | [1.53; 5.38] | 4.2% | 1.6% |
| Hashemi, 2016 | 1.56 | [0.99; 2.48] | 6.6% | 3.8% |
| Miao, 2016 | 1.02 | [0.74; 1.42] | 9.9% | 9.4% |
| Ni, 2016 | 1.05 | [0.68; 1.62] | 7.2% | 5.4% |
| Yang, 2016 | 0.98 | [0.24; 4.07] | 1.0% | 0.5% |
| Dong, 2015 | 0.75 | [0.52; 1.08] | 8.7% | 8.5% |
| Sushma, 2015 | 2.29 | [1.06; 4.94] | 3.0% | 1.2% |
| Du, 2014 | 0.87 | [0.53; 1.41] | 6.2% | 4.7% |
| Wei, 2014 | 0.68 | [0.50; 0.94] | 10.2% | 12.2% |
| Hu, 2013 | 1.10 | [0.76; 1.57] | 9.0% | 7.5% |
| Huang, 2013 | 1.05 | [0.57; 1.92] | 4.5% | 2.7% |
| Chu, 2012 | 0.74 | [0.41; 1.32] | 4.7% | 3.5% |
| Tu, 2012 | 2.60 | [1.30; 5.20] | 3.6% | 1.2% |
| Liu, 2010 | 1.00 | [0.84; 1.19] | 15.6% | 33.3% |
| **Fixed effect model** | **1.01** | **[0.91; 1.12]** | **--** | **100.0%** |
| **Random effects model** | **1.07** | **[0.84; 1.36]** | **100.0%** | **--** |
| Heterogeneity: $I^2 = 64\%$, $\tau^2 = 0.0770$, $p < 0.01$ | | | | |
| Test for effect in subgroup (fixed effect): $z = 0.20$ ($p = 0.84$) | | | | |
| Test for effect in subgroup (random effects): $t_{14} = 0.62$ ($p = 0.55$) | | | | |
| **CRC** | | | | |
| Ranjbar, 2018 | 0.29 | [0.07; 1.15] | 4.0% | 4.9% |
| Lv, 2013 | 0.99 | [0.57; 1.73] | 19.1% | 16.1% |
| Vinci, 2013 | 0.84 | [0.53; 1.33] | 24.9% | 25.4% |
| Min, 2012 | 0.86 | [0.55; 1.33] | 26.2% | 27.1% |
| Mw Zhang−a, 2012 | 0.87 | [0.55; 1.35] | 25.8% | 26.4% |
| **Fixed effect model** | **0.85** | **[0.68; 1.07]** | **--** | **100.0%** |
| **Random effects model** | **0.85** | **[0.65; 1.11]** | **100.0%** | **--** |
| Heterogeneity: $I^2 = 0\%$, $\tau^2 = 0$, $p = 0.62$ | | | | |
| Test for effect in subgroup (fixed effect): $z = -1.38$ ($p = 0.17$) | | | | |
| Test for effect in subgroup (random effects): $t_{14} = -1.67$ ($p = 0.17$) | | | | |
| **GC** | | | | |
| Cîmpeanu, 2017 | 0.74 | [0.47; 1.14] | 13.7% | 14.9% |
| Gu, 2016 | 1.12 | [0.70; 1.79] | 12.6% | 10.6% |
| Jiang, 2016 | 0.82 | [0.55; 1.22] | 15.6% | 17.2% |
| Su, 2016 | 1.10 | [0.78; 1.57] | 18.1% | 19.2% |
| Dikeakos, 2014 | 0.58 | [0.31; 1.07] | 8.6% | 8.3% |
| Pu, 2014 | 0.66 | [0.34; 1.25] | 7.9% | 7.2% |
| Ahn, 2012 | 0.86 | [0.53; 1.38] | 12.5% | 11.9% |
| Mw Zhang−b, 2012 | 0.72 | [0.43; 1.21] | 10.9% | 10.7% |
| **Fixed effect model** | **0.85** | **[0.73; 1.01]** | **--** | **100.0%** |
| **Random effects model** | **0.85** | **[0.71; 1.03]** | **100.0%** | **--** |
| Heterogeneity: $I^2 = 0\%$, $\tau^2 = 0$, $p = 0.49$ | | | | |
| Test for effect in subgroup (fixed effect): $z = -1.88$ ($p = 0.06$) | | | | |
| Test for effect in subgroup (random effects): $t_{14} = -1.99$ ($p = 0.09$) | | | | |
| **LC** | | | | |
| Li, 2016 | 1.23 | [0.78; 1.93] | 29.3% | 21.8% |
| Vinci, 2011 | 1.14 | [0.65; 2.00] | 21.8% | 15.1% |
| Tian, 2009 | 0.95 | [0.71; 1.26] | 48.9% | 63.1% |
| **Fixed effect model** | **1.04** | **[0.83; 1.30]** | **--** | **100.0%** |
| **Random effects model** | **1.04** | **[0.73; 1.47]** | **100.0%** | **--** |
| Heterogeneity: $I^2 = 0\%$, $\tau^2 = 0$, $p = 0.60$ | | | | |
| Test for effect in subgroup (fixed effect): $z = 0.34$ ($p = 0.74$) | | | | |
| Test for effect in subgroup (random effects): $t_{14} = 0.48$ ($p = 0.68$) | | | | |
| **BC** | | | | |
| He, 2015 | 1.62 | [1.02; 2.57] | 21.5% | 9.3% |
| Zhang, 2012 | 0.94 | [0.50; 1.77] | 13.5% | 6.3% |
| Kontorovich, 2010 | 0.81 | [0.45; 1.46] | 15.2% | 8.0% |
| Hu, 2009 | 0.98 | [0.82; 1.17] | 49.8% | 76.4% |
| **Fixed effect model** | **1.02** | **[0.88; 1.20]** | **--** | **100.0%** |
| **Random effects model** | **1.06** | **[0.69; 1.62]** | **100.0%** | **--** |
| Heterogeneity: $I^2 = 37\%$, $\tau^2 = 0.0268$, $p = 0.19$ | | | | |
| Test for effect in subgroup (fixed effect): $z = 0.29$ ($p = 0.77$) | | | | |
| Test for effect in subgroup (random effects): $t_{14} = 0.40$ ($p = 0.72$) | | | | |
| **HCC** | | | | |
| Liu, 2015 | 1.24 | [0.86; 1.80] | 15.8% | 14.2% |
| Pratedrat, 2015 | 0.92 | [0.33; 2.60] | 3.2% | 2.1% |
| Yan, 2015 | 0.93 | [0.62; 1.40] | 14.3% | 13.8% |
| Chu, 2014 | 1.17 | [0.54; 2.54] | 5.4% | 3.4% |
| Kou, 2014 | 0.86 | [0.63; 1.18] | 18.9% | 23.7% |
| Liu, 2014 | 0.69 | [0.46; 1.04] | 14.1% | 15.7% |
| Wang, 2014 | 0.69 | [0.48; 1.01] | 15.8% | 19.1% |
| Wang, 2014 | 1.61 | [0.81; 3.18] | 6.7% | 4.0% |
| Kim, 2012 | 0.99 | [0.47; 2.09] | 5.8% | 4.0% |
| **Fixed effect model** | **0.91** | **[0.78; 1.06]** | **--** | **100.0%** |
| **Random effects model** | **0.92** | **[0.75; 1.13]** | **100.0%** | **--** |
| Heterogeneity: $I^2 = 18\%$, $\tau^2 = 0.0123$, $p = 0.29$ | | | | |
| Test for effect in subgroup (fixed effect): $z = -1.17$ ($p = 0.24$) | | | | |
| Test for effect in subgroup (random effects): $t_{14} = -0.95$ ($p = 0.37$) | | | | |

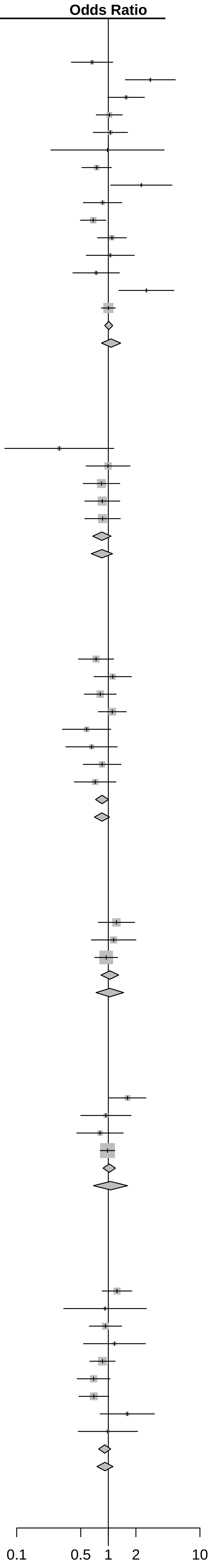

**C) Dominant comparison**

| Study | OR | 95%−CI | Weight (random) | Weight (fixed) |
|---|---|---|---|---|
| **Others** | | | | |
| He, 2018 | 0.88 | [0.56; 1.37] | 6.6% | 4.6% |
| Tandon, 2017 | 2.87 | [1.72; 4.78] | 5.7% | 2.1% |
| Hashemi, 2016 | 1.49 | [0.98; 2.27] | 7.1% | 4.1% |
| Miao, 2016 | 1.07 | [0.79; 1.46] | 9.2% | 9.2% |
| Ni, 2016 | 1.06 | [0.70; 1.60] | 7.2% | 5.1% |
| Yang, 2016 | 0.91 | [0.23; 3.60] | 1.2% | 0.5% |
| Dong, 2015 | 0.67 | [0.47; 0.95] | 8.4% | 8.6% |
| Sushma, 2015 | 2.92 | [1.39; 6.14] | 3.4% | 1.0% |
| Du, 2014 | 0.75 | [0.47; 1.18] | 6.5% | 4.9% |
| Wei, 2014 | 0.65 | [0.48; 0.88] | 9.3% | 12.2% |
| Hu, 2013 | 1.11 | [0.79; 1.56] | 8.5% | 7.3% |
| Huang, 2013 | 1.19 | [0.67; 2.09] | 5.0% | 2.6% |
| Chu, 2012 | 0.76 | [0.45; 1.28] | 5.6% | 3.8% |
| Tu, 2012 | 2.63 | [1.37; 5.06] | 4.1% | 1.2% |
| Liu, 2010 | 0.98 | [0.83; 1.16] | 12.2% | 32.7% |
| **Fixed effect model** | **1.02** | **[0.93; 1.12]** | **--** | **100.0%** |
| **Random effects model** | **1.11** | **[0.85; 1.44]** | **100.0%** | **--** |
| Heterogeneity: $I^2 = 74\%$, $\tau^2 = 0.1070$, $p < 0.01$ | | | | |
| Test for effect in subgroup (fixed effect): $z = 0.34$ ($p = 0.73$) | | | | |
| Test for effect in subgroup (random effects): $t_{14} = 0.83$ ($p = 0.42$) | | | | |
| **CRC** | | | | |
| Ranjbar, 2018 | 0.49 | [0.14; 1.72] | 5.0% | 4.3% |
| Lv, 2013 | 1.23 | [0.76; 1.99] | 21.4% | 17.9% |
| Vinci, 2013 | 0.96 | [0.63; 1.47] | 24.3% | 25.2% |
| Min, 2012 | 0.94 | [0.62; 1.42] | 24.9% | 26.7% |
| Mw Zhang−a, 2012 | 0.93 | [0.61; 1.42] | 24.4% | 25.9% |
| **Fixed effect model** | **0.97** | **[0.79; 1.21]** | **--** | **100.0%** |
| **Random effects model** | **0.98** | **[0.78; 1.22]** | **100.0%** | **--** |
| Heterogeneity: $I^2 = 0\%$, $\tau^2 = 0$, $p = 0.70$ | | | | |
| Test for effect in subgroup (fixed effect): $z = -0.24$ ($p = 0.81$) | | | | |
| Test for effect in subgroup (random effects): $t_{14} = -0.31$ ($p = 0.77$) | | | | |
| **GC** | | | | |
| Cîmpeanu, 2017 | 0.80 | [0.53; 1.20] | 13.7% | 15.1% |
| Gu, 2016 | 1.13 | [0.73; 1.76] | 12.5% | 10.7% |
| Jiang, 2016 | 0.92 | [0.64; 1.31] | 15.3% | 18.1% |
| Su, 2016 | 1.14 | [0.81; 1.59] | 16.0% | 18.3% |
| Dikeakos, 2014 | 0.50 | [0.28; 0.89] | 9.1% | 8.4% |
| Pu, 2014 | 0.88 | [0.51; 1.50] | 10.0% | 8.0% |
| Ahn, 2012 | 0.93 | [0.59; 1.46] | 12.2% | 11.3% |
| Mw Zhang−b, 2012 | 0.85 | [0.52; 1.38] | 11.2% | 10.1% |
| **Fixed effect model** | **0.92** | **[0.79; 1.07]** | **--** | **100.0%** |
| **Random effects model** | **0.91** | **[0.76; 1.10]** | **100.0%** | **--** |
| Heterogeneity: $I^2 = 4\%$, $\tau^2 = 0.0021$, $p = 0.40$ | | | | |
| Test for effect in subgroup (fixed effect): $z = -1.08$ ($p = 0.28$) | | | | |
| Test for effect in subgroup (random effects): $t_{14} = -1.13$ ($p = 0.30$) | | | | |
| **LC** | | | | |
| Li, 2016 | 1.23 | [0.80; 1.90] | 30.6% | 21.5% |
| Vinci, 2011 | 1.32 | [0.78; 2.22] | 24.8% | 14.5% |
| Tian, 2009 | 0.92 | [0.70; 1.21] | 44.6% | 64.0% |
| **Fixed effect model** | **1.05** | **[0.85; 1.29]** | **--** | **100.0%** |
| **Random effects model** | **1.05** | **[0.65; 1.69]** | **100.0%** | **--** |
| Heterogeneity: $I^2 = 4\%$, $\tau^2 = 0.0019$, $p = 0.35$ | | | | |
| Test for effect in subgroup (fixed effect): $z = 0.41$ ($p = 0.68$) | | | | |
| Test for effect in subgroup (random effects): $t_{14} = 0.44$ ($p = 0.70$) | | | | |
| **BC** | | | | |
| He, 2015 | 1.77 | [1.14; 2.74] | 22.8% | 8.8% |
| Zhang, 2012 | 1.13 | [0.62; 2.06] | 15.5% | 5.6% |
| Kontorovich, 2010 | 0.71 | [0.44; 1.13] | 21.1% | 11.7% |
| Hu, 2009 | 0.98 | [0.82; 1.16] | 40.6% | 73.8% |
| **Fixed effect model** | **1.03** | **[0.89; 1.19]** | **--** | **100.0%** |
| **Random effects model** | **1.08** | **[0.60; 1.94]** | **100.0%** | **--** |
| Heterogeneity: $I^2 = 66\%$, $\tau^2 = 0.0738$, $p = 0.03$ | | | | |
| Test for effect in subgroup (fixed effect): $z = 0.34$ ($p = 0.73$) | | | | |
| Test for effect in subgroup (random effects): $t_{14} = 0.39$ ($p = 0.72$) | | | | |
| **HCC** | | | | |
| Liu, 2015 | 1.31 | [0.92; 1.87] | 15.0% | 13.5% |
| Pratedrat, 2015 | 0.88 | [0.35; 2.24] | 4.3% | 2.4% |
| Yan, 2015 | 0.89 | [0.61; 1.30] | 14.0% | 14.0% |
| Chu, 2014 | 1.17 | [0.59; 2.33] | 6.9% | 3.8% |
| Kou, 2014 | 0.85 | [0.63; 1.15] | 16.9% | 23.2% |
| Liu, 2014 | 0.60 | [0.41; 0.87] | 14.0% | 17.4% |
| Wang, 2014 | 0.70 | [0.49; 1.00] | 14.8% | 18.4% |
| Wang, 2014 | 1.76 | [0.92; 3.39] | 7.4% | 3.7% |
| Kim, 2012 | 1.21 | [0.59; 2.46] | 6.6% | 3.5% |
| **Fixed effect model** | **0.91** | **[0.79; 1.04]** | **--** | **100.0%** |
| **Random effects model** | **0.94** | **[0.73; 1.21]** | **100.0%** | **--** |
| Heterogeneity: $I^2 = 51\%$, $\tau^2 = 0.0518$, $p = 0.04$ | | | | |
| Test for effect in subgroup (fixed effect): $z = -1.35$ ($p = 0.18$) | | | | |
| Test for effect in subgroup (random effects): $t_{14} = -0.59$ ($p = 0.57$) | | | | |

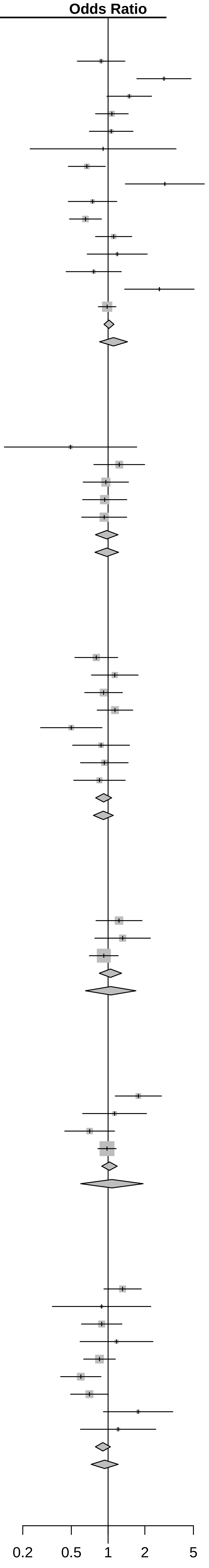

**D) Recessive comparison**

| Study | OR | 95%−CI | Weight (random) | Weight (fixed) |
|---|---|---|---|---|
| **Others** | | | | |
| He, 2018 | 1.26 | [0.95; 1.66] | 8.8% | 7.1% |
| Tandon, 2017 | 2.47 | [1.14; 5.33] | 2.0% | 0.7% |
| Hashemi, 2016 | 1.09 | [0.59; 2.00] | 3.0% | 1.6% |
| Miao, 2016 | 1.10 | [0.91; 1.33] | 12.0% | 15.8% |
| Ni, 2016 | 1.05 | [0.63; 1.75] | 4.0% | 2.3% |
| Yang, 2016 | 0.84 | [0.36; 1.95] | 1.7% | 0.9% |
| Dong, 2015 | 0.74 | [0.57; 0.95] | 9.5% | 10.8% |
| Sushma, 2015 | 14.10 | [0.78; 253.71] | 0.2% | 0.0% |
| Du, 2014 | 0.72 | [0.54; 0.97] | 8.3% | 8.2% |
| Wei, 2014 | 0.85 | [0.71; 1.02] | 12.4% | 19.5% |
| Hu, 2013 | 1.05 | [0.85; 1.29] | 11.2% | 13.1% |
| Huang, 2013 | 1.31 | [0.87; 1.97] | 5.5% | 3.2% |
| Chu, 2012 | 0.96 | [0.72; 1.30] | 8.2% | 6.9% |
| Tu, 2012 | 1.24 | [0.80; 1.91] | 5.1% | 2.9% |
| Liu, 2010 | 0.90 | [0.67; 1.21] | 8.1% | 7.1% |
| **Fixed effect model** | **0.98** | **[0.91; 1.06]** | **−−** | **100.0%** |
| **Random effects model** | **1.00** | **[0.87; 1.16]** | **100.0%** | **−−** |

Heterogeneity: $I^2$ = 51%, $\tau^2$ = 0.0261, $p$ = 0.01
Test for effect in subgroup (fixed effect): $z$ = −0.46 ($p$ = 0.65)
Test for effect in subgroup (random effects): $t_{14}$ = 0.04 ($p$ = 0.97)

| Study | OR | 95%−CI | Weight (random) | Weight (fixed) |
|---|---|---|---|---|
| **CRC** | | | | |
| Ranjbar, 2018 | 1.55 | [0.77; 3.09] | 7.6% | 4.2% |
| Lv, 2013 | 1.32 | [0.97; 1.79] | 25.0% | 23.1% |
| Vinci, 2013 | 1.59 | [0.82; 3.10] | 8.1% | 4.4% |
| Min, 2012 | 1.14 | [0.89; 1.48] | 30.2% | 35.4% |
| Mw Zhang−a, 2012 | 1.12 | [0.86; 1.46] | 29.0% | 32.9% |
| **Fixed effect model** | **1.21** | **[1.04; 1.41]** | **−−** | **100.0%** |
| **Random effects model** | **1.21** | **[1.05; 1.41]** | **100.0%** | **−−** |

Heterogeneity: $I^2$ = 0%, $\tau^2$ = 0, $p$ = 0.75
Test for effect in subgroup (fixed effect): $z$ = 2.53 ($p$ = 0.01)
Test for effect in subgroup (random effects): $t_{14}$ = 3.63 ($p$ = 0.02)

| Study | OR | 95%−CI | Weight (random) | Weight (fixed) |
|---|---|---|---|---|
| **GC** | | | | |
| Cîmpeanu, 2017 | 1.13 | [0.65; 1.97] | 6.8% | 4.3% |
| Gu, 2016 | 1.10 | [0.67; 1.80] | 8.1% | 5.6% |
| Jiang, 2016 | 1.10 | [0.90; 1.35] | 22.1% | 32.1% |
| Su, 2016 | 1.26 | [0.71; 2.25] | 6.4% | 3.8% |
| Dikeakos, 2014 | 0.68 | [0.48; 0.97] | 12.7% | 13.6% |
| Pu, 2014 | 1.24 | [0.85; 1.80] | 12.1% | 9.4% |
| Ahn, 2012 | 1.13 | [0.87; 1.47] | 18.2% | 19.9% |
| Mw Zhang−b, 2012 | 1.26 | [0.90; 1.77] | 13.7% | 11.1% |
| **Fixed effect model** | **1.09** | **[0.97; 1.22]** | **−−** | **100.0%** |
| **Random effects model** | **1.09** | **[0.93; 1.28]** | **100.0%** | **−−** |

Heterogeneity: $I^2$ = 14%, $\tau^2$ = 0.0051, $p$ = 0.32
Test for effect in subgroup (fixed effect): $z$ = 1.42 ($p$ = 0.16)
Test for effect in subgroup (random effects): $t_{14}$ = 1.25 ($p$ = 0.25)

| Study | OR | 95%−CI | Weight (random) | Weight (fixed) |
|---|---|---|---|---|
| **LC** | | | | |
| Li, 2016 | 1.04 | [0.80; 1.35] | 39.2% | 28.9% |
| Vinci, 2011 | 2.02 | [0.89; 4.57] | 7.5% | 2.1% |
| Tian, 2009 | 0.94 | [0.79; 1.11] | 53.3% | 69.0% |
| **Fixed effect model** | **0.99** | **[0.86; 1.14]** | **−−** | **100.0%** |
| **Random effects model** | **1.03** | **[0.57; 1.84]** | **100.0%** | **−−** |

Heterogeneity: $I^2$ = 42%, $\tau^2$ = 0.0168, $p$ = 0.18
Test for effect in subgroup (fixed effect): $z$ = −0.16 ($p$ = 0.87)
Test for effect in subgroup (random effects): $t_{14}$ = 0.21 ($p$ = 0.86)

| Study | OR | 95%−CI | Weight (random) | Weight (fixed) |
|---|---|---|---|---|
| **BC** | | | | |
| He, 2015 | 1.29 | [1.00; 1.68] | 33.1% | 35.8% |
| Zhang, 2012 | 1.43 | [0.99; 2.06] | 22.9% | 17.7% |
| Kontorovich, 2010 | 0.67 | [0.40; 1.13] | 13.7% | 12.5% |
| Hu, 2009 | 0.99 | [0.74; 1.32] | 30.2% | 34.1% |
| **Fixed effect model** | **1.14** | **[0.97; 1.34]** | **−−** | **100.0%** |
| **Random effects model** | **1.10** | **[0.68; 1.78]** | **100.0%** | **−−** |

Heterogeneity: $I^2$ = 59%, $\tau^2$ = 0.0420, $p$ = 0.06
Test for effect in subgroup (fixed effect): $z$ = 1.56 ($p$ = 0.12)
Test for effect in subgroup (random effects): $t_{14}$ = 0.65 ($p$ = 0.56)

| Study | OR | 95%−CI | Weight (random) | Weight (fixed) |
|---|---|---|---|---|
| **HCC** | | | | |
| Liu, 2015 | 1.39 | [0.86; 2.25] | 7.9% | 5.0% |
| Pratedrat, 2015 | 0.92 | [0.52; 1.65] | 5.9% | 4.2% |
| Yan, 2015 | 0.86 | [0.60; 1.23] | 12.1% | 11.6% |
| Chu, 2014 | 1.05 | [0.70; 1.57] | 10.2% | 8.1% |
| Kou, 2014 | 0.88 | [0.57; 1.35] | 9.3% | 7.9% |
| Liu, 2014 | 0.64 | [0.47; 0.89] | 13.6% | 16.2% |
| Wang, 2014 | 0.96 | [0.78; 1.18] | 20.9% | 33.3% |
| Wang, 2014 | 1.33 | [0.90; 1.98] | 10.5% | 7.4% |
| Kim, 2012 | 1.48 | [0.97; 2.24] | 9.7% | 6.3% |
| **Fixed effect model** | **0.98** | **[0.87; 1.10]** | **−−** | **100.0%** |
| **Random effects model** | **1.00** | **[0.81; 1.23]** | **100.0%** | **−−** |

Heterogeneity: $I^2$ = 49%, $\tau^2$ = 0.0327, $p$ = 0.05
Test for effect in subgroup (fixed effect): $z$ = −0.38 ($p$ = 0.70)
Test for effect in subgroup (random effects): $t_{14}$ = 0.01 ($p$ = 0.99)

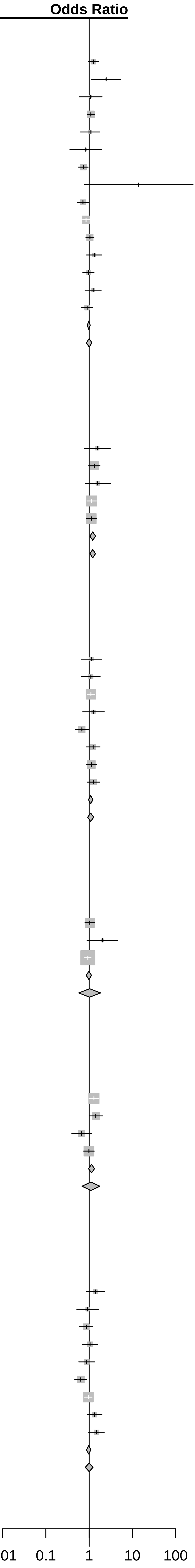

**E) Allelic comparison**

| Study | OR | 95%−CI | Weight (random) | Weight (fixed) |
|---|---|---|---|---|
| **Others** | | | | |
| He, 2018 | 1.13 | [0.90; 1.42] | 7.4% | 5.3% |
| Tandon, 2017 | 2.65 | [1.74; 4.03] | 3.9% | 1.1% |
| Hashemi, 2016 | 1.29 | [0.94; 1.77] | 5.5% | 2.5% |
| Miao, 2016 | 1.07 | [0.93; 1.24] | 9.5% | 13.3% |
| Ni, 2016 | 1.04 | [0.79; 1.36] | 6.4% | 3.9% |
| Yang, 2016 | 0.90 | [0.49; 1.64] | 2.3% | 0.8% |
| Dong, 2015 | 0.77 | [0.64; 0.92] | 8.6% | 10.1% |
| Sushma, 2015 | 3.28 | [1.64; 6.53] | 1.8% | 0.4% |
| Du, 2014 | 0.78 | [0.63; 0.97] | 7.6% | 6.9% |
| Wei, 2014 | 0.83 | [0.72; 0.95] | 9.7% | 16.5% |
| Hu, 2013 | 1.05 | [0.89; 1.23] | 9.1% | 11.2% |
| Huang, 2013 | 1.20 | [0.90; 1.62] | 5.9% | 3.1% |
| Chu, 2012 | 0.91 | [0.71; 1.17] | 6.9% | 4.9% |
| Tu, 2012 | 1.40 | [1.02; 1.92] | 5.5% | 2.4% |
| Liu, 2010 | 0.97 | [0.85; 1.10] | 9.9% | 17.5% |
| **Fixed effect model** | **1.00** | **[0.94; 1.05]** | **--** | **100.0%** |
| **Random effects model** | **1.08** | **[0.90; 1.30]** | **100.0%** | **--** |
| Heterogeneity: $I^2$ = 78%, $\tau^2$ = 0.0426, $p$ < 0.01 | | | | |
| Test for effect in subgroup (fixed effect): $z$ = −0.12 ($p$ = 0.91) | | | | |
| Test for effect in subgroup (random effects): $t_{14}$ = 0.89 ($p$ = 0.39) | | | | |
| **CRC** | | | | |
| Ranjbar, 2018 | 1.15 | [0.65; 2.01] | 8.2% | 3.8% |
| Lv, 2013 | 1.27 | [0.99; 1.63] | 22.1% | 18.8% |
| Vinci, 2013 | 1.09 | [0.79; 1.51] | 17.2% | 11.8% |
| Min, 2012 | 1.07 | [0.88; 1.29] | 26.4% | 33.3% |
| Mw Zhang−a, 2012 | 1.05 | [0.86; 1.28] | 26.1% | 32.3% |
| **Fixed effect model** | **1.11** | **[0.99; 1.24]** | **--** | **100.0%** |
| **Random effects model** | **1.10** | **[1.00; 1.22]** | **100.0%** | **--** |
| Heterogeneity: $I^2$ = 0%, $\tau^2$ = 0, $p$ = 0.80 | | | | |
| Test for effect in subgroup (fixed effect): $z$ = 1.76 ($p$ = 0.08) | | | | |
| Test for effect in subgroup (random effects): $t_{14}$ = 2.73 ($p$ = 0.05) | | | | |
| **GC** | | | | |
| Cîmpeanu, 2017 | 0.92 | [0.68; 1.24] | 10.6% | 8.5% |
| Gu, 2016 | 1.09 | [0.82; 1.46] | 11.0% | 8.4% |
| Jiang, 2016 | 1.05 | [0.88; 1.25] | 16.1% | 24.1% |
| Su, 2016 | 1.13 | [0.87; 1.45] | 12.4% | 10.7% |
| Dikeakos, 2014 | 0.70 | [0.53; 0.91] | 11.8% | 11.8% |
| Pu, 2014 | 1.10 | [0.82; 1.49] | 10.7% | 7.9% |
| Ahn, 2012 | 1.06 | [0.87; 1.30] | 14.7% | 17.3% |
| Mw Zhang−b, 2012 | 1.09 | [0.85; 1.40] | 12.6% | 11.2% |
| **Fixed effect model** | **1.02** | **[0.94; 1.11]** | **--** | **100.0%** |
| **Random effects model** | **1.01** | **[0.89; 1.15]** | **100.0%** | **--** |
| Heterogeneity: $I^2$ = 29%, $\tau^2$ = 0.0064, $p$ = 0.19 | | | | |
| Test for effect in subgroup (fixed effect): $z$ = 0.42 ($p$ = 0.67) | | | | |
| Test for effect in subgroup (random effects): $t_{14}$ = 0.25 ($p$ = 0.81) | | | | |
| **LC** | | | | |
| Li, 2016 | 1.07 | [0.88; 1.29] | 36.7% | 27.3% |
| Vinci, 2011 | 1.38 | [0.93; 2.05] | 19.0% | 5.9% |
| Tian, 2009 | 0.95 | [0.83; 1.08] | 44.3% | 66.8% |
| **Fixed effect model** | **1.00** | **[0.91; 1.11]** | **--** | **100.0%** |
| **Random effects model** | **1.04** | **[0.71; 1.53]** | **100.0%** | **--** |
| Heterogeneity: $I^2$ = 46%, $\tau^2$ = 0.0095, $p$ = 0.16 | | | | |
| Test for effect in subgroup (fixed effect): $z$ = 0.08 ($p$ = 0.94) | | | | |
| Test for effect in subgroup (random effects): $t_{14}$ = 0.45 ($p$ = 0.70) | | | | |
| **BC** | | | | |
| He, 2015 | 1.32 | [1.08; 1.61] | 27.4% | 20.8% |
| Zhang, 2012 | 1.25 | [0.95; 1.64] | 21.6% | 11.5% |
| Kontorovich, 2010 | 0.71 | [0.50; 0.99] | 17.4% | 9.9% |
| Hu, 2009 | 0.99 | [0.87; 1.12] | 33.6% | 57.7% |
| **Fixed effect model** | **1.06** | **[0.96; 1.17]** | **--** | **100.0%** |
| **Random effects model** | **1.06** | **[0.69; 1.61]** | **100.0%** | **--** |
| Heterogeneity: $I^2$ = 76%, $\tau^2$ = 0.0385, $p$ < 0.01 | | | | |
| Test for effect in subgroup (fixed effect): $z$ = 1.15 ($p$ = 0.25) | | | | |
| Test for effect in subgroup (random effects): $t_{14}$ = 0.41 ($p$ = 0.71) | | | | |
| **HCC** | | | | |
| Liu, 2015 | 1.25 | [0.98; 1.60] | 11.8% | 9.3% |
| Pratedrat, 2015 | 0.92 | [0.58; 1.47] | 5.7% | 3.0% |
| Yan, 2015 | 0.90 | [0.72; 1.13] | 12.6% | 12.9% |
| Chu, 2014 | 1.07 | [0.77; 1.51] | 8.7% | 5.4% |
| Kou, 2014 | 0.89 | [0.72; 1.10] | 13.1% | 14.5% |
| Liu, 2014 | 0.68 | [0.55; 0.85] | 12.9% | 15.8% |
| Wang, 2014 | 0.91 | [0.78; 1.06] | 15.7% | 27.1% |
| Wang, 2014 | 1.32 | [0.98; 1.76] | 10.2% | 6.6% |
| Kim, 2012 | 1.30 | [0.94; 1.78] | 9.3% | 5.5% |
| **Fixed effect model** | **0.96** | **[0.88; 1.04]** | **--** | **100.0%** |
| **Random effects model** | **0.99** | **[0.84; 1.18]** | **100.0%** | **--** |
| Heterogeneity: $I^2$ = 66%, $\tau^2$ = 0.0303, $p$ < 0.01 | | | | |
| Test for effect in subgroup (fixed effect): $z$ = −1.03 ($p$ = 0.30) | | | | |
| Test for effect in subgroup (random effects): $t_{14}$ = −0.12 ($p$ = 0.90) | | | | |

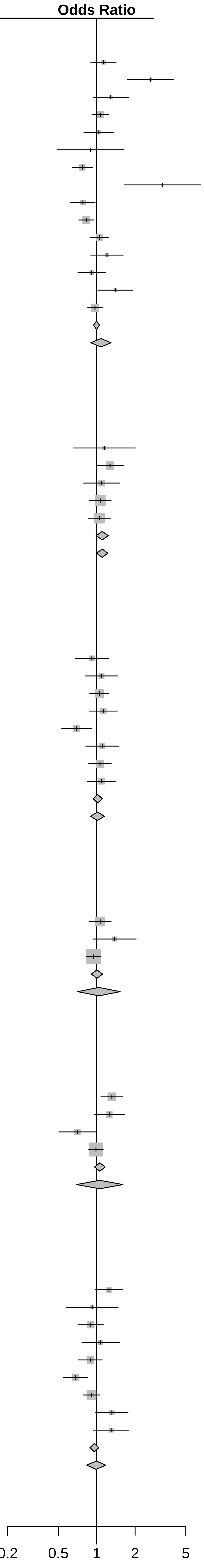

Supplement: Supplementary file 1 [file Data_Sheet_1.ZIP › Supplementary Figures/Suppl. Fig. S6.pdf]
