# Supplementary material for: Association of mir-196a-2 rs11614913 and mir-149 rs2292832 Polymorphisms With Risk of Cancer: An Updated Meta-Analysis
Source: Front Genet. 2019 Mar 15;10:186. doi: 10.3389/fgene.2019.00186 (PMC6429108; doi:10.3389/fgene.2019.00186)
Supplement: Supplementary file 1 [file Data_Sheet_1.ZIP › Supplementary Figures/Suppl. Fig. S8.pdf]

Supplementary Figure S8. Sensitivity analyses for meta-analysis of miR-196a-2 rs11614913 and cancer risk under different genetic models in order to detect potential influential studies using leave-one-out approach. **A)** Homozygote; **B)** Heterozygote; **C)** Dominant; **D)** Recessive **E)** allele contrast. Each line represents the overall estimate while omitting one study

A)

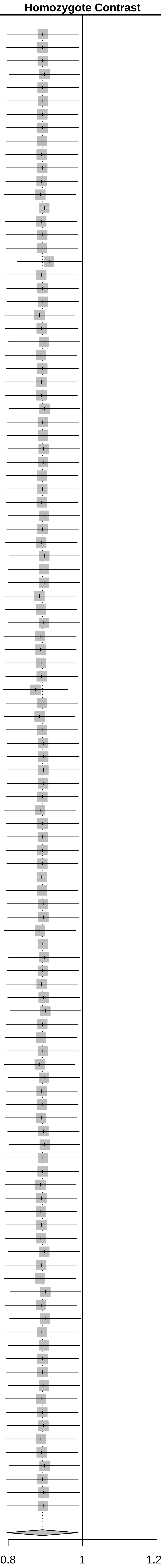

Study	OR	95%−CI
Omitting Abdel−Hamid, 2018	0.888	[0.798; 0.988]
Omitting Afsharzadeh, 2017	0.887	[0.797; 0.987]
Omitting Ahn, 2013	0.887	[0.797; 0.988]
Omitting Akkiz, 2011	0.892	[0.802; 0.992]
Omitting Alshatwi, 2012	0.887	[0.797; 0.987]
Omitting Bansal, 2014	0.888	[0.798; 0.988]
Omitting Bodal, 2017	0.887	[0.797; 0.986]
Omitting Catucci, 1, 2010	0.887	[0.796; 0.988]
Omitting Catucci, 2, 2010	0.886	[0.795; 0.986]
Omitting Chen, 2012	0.885	[0.795; 0.985]
Omitting Christensen, 2010	0.886	[0.796; 0.987]
Omitting Chu, 2014	0.885	[0.794; 0.985]
Omitting Chu, 2012	0.881	[0.792; 0.981]
Omitting Dai, 2016	0.892	[0.801; 0.992]
Omitting Damodaran, 2018	0.884	[0.794; 0.983]
Omitting Deng, 2015	0.886	[0.796; 0.987]
Omitting Dikaiakos, 2015	0.886	[0.795; 0.986]
Omitting Dikeakos, 2014	0.905	[0.822; 0.996]
Omitting Dou, 2010	0.884	[0.794; 0.984]
Omitting Doulah, 2018	0.887	[0.797; 0.987]
Omitting George, 2011	0.888	[0.798; 0.988]
Omitting Gu, 2016	0.879	[0.791; 0.977]
Omitting Han, 2013	0.885	[0.794; 0.985]
Omitting Hao, 2014	0.891	[0.801; 0.992]
Omitting Hashemi, 2016	0.883	[0.793; 0.982]
Omitting He, 2018	0.886	[0.796; 0.987]
Omitting He, 2015	0.884	[0.794; 0.984]
Omitting Hezova, 2012	0.884	[0.794; 0.984]
Omitting Hoffman, 2009	0.892	[0.802; 0.993]
Omitting Hong, 2011	0.888	[0.797; 0.988]
Omitting Horikawa, 2008	0.888	[0.798; 0.989]
Omitting Hu, 2013	0.890	[0.799; 0.991]
Omitting Hu, 2009	0.889	[0.798; 0.990]
Omitting Huang, 2017	0.886	[0.795; 0.986]
Omitting Jedlinski, 2011	0.886	[0.796; 0.987]
Omitting Kim, 2012	0.885	[0.795; 0.985]
Omitting Kou, 2014	0.891	[0.801; 0.992]
Omitting Kupcinskas, 2014	0.887	[0.797; 0.988]
Omitting Kupcinskas, 2014	0.884	[0.794; 0.984]
Omitting Li, 2016	0.892	[0.802; 0.992]
Omitting Li, 2016	0.891	[0.801; 0.991]
Omitting Li, 2015	0.891	[0.801; 0.992]
Omitting Li, 2015	0.879	[0.790; 0.977]
Omitting Li, 2014	0.883	[0.793; 0.983]
Omitting Li, 2010	0.890	[0.800; 0.991]
Omitting Linhares, 2012	0.880	[0.791; 0.980]
Omitting Liu, 2015	0.882	[0.792; 0.981]
Omitting Liu, 2013	0.883	[0.793; 0.983]
Omitting Liu, 2010	0.885	[0.794; 0.985]
Omitting Lv, 2013	0.869	[0.789; 0.957]
Omitting Ma, 2013	0.885	[0.795; 0.986]
Omitting Martin−Guerrero, 2015	0.879	[0.791; 0.977]
Omitting Mashayekhi, 2018	0.886	[0.796; 0.986]
Omitting Miao, 2016	0.889	[0.798; 0.990]
Omitting Min, 2013	0.889	[0.798; 0.990]
Omitting Minh, 2018	0.889	[0.799; 0.990]
Omitting Mittal, 2011	0.889	[0.799; 0.989]
Omitting Morales, 2016	0.887	[0.796; 0.987]
Omitting Mulong Du, 2014	0.881	[0.791; 0.980]
Omitting Ni, 2016	0.887	[0.797; 0.988]
Omitting Nikolic, 2015	0.888	[0.797; 0.988]
Omitting Okubo, 2010	0.887	[0.796; 0.988]
Omitting Omrani, 2014	0.887	[0.797; 0.986]
Omitting Parlayan, 2014	0.885	[0.795; 0.986]
Omitting Pavlakis, 2013	0.885	[0.795; 0.986]
Omitting Peckham−Gregory, 2016	0.889	[0.798; 0.989]
Omitting Peng, 2010	0.889	[0.799; 0.990]
Omitting Poltronieri−Oliveira, 2017	0.880	[0.791; 0.979]
Omitting Pu, 2014	0.888	[0.797; 0.989]
Omitting Qi, 2015	0.891	[0.801; 0.992]
Omitting Qi, 2014	0.888	[0.797; 0.988]
Omitting Qi, 2010	0.885	[0.794; 0.985]
Omitting Qu, 2014	0.890	[0.799; 0.991]
Omitting Rakmanee, 2017	0.895	[0.805; 0.994]
Omitting Rogoveanu, 2017	0.886	[0.796; 0.987]
Omitting Roy, 2014	0.883	[0.793; 0.983]
Omitting Shen, 2016	0.888	[0.797; 0.989]
Omitting Sodhi, 2015	0.879	[0.792; 0.977]
Omitting Song, 2016	0.891	[0.801; 0.992]
Omitting Srivastava, 2017	0.885	[0.795; 0.985]
Omitting Srivastava, 2010	0.886	[0.796; 0.986]
Omitting Su, 2016	0.884	[0.794; 0.984]
Omitting Sun, 2016	0.889	[0.799; 0.990]
Omitting Sushma, 2015	0.893	[0.803; 0.992]
Omitting Tian, 2009	0.888	[0.797; 0.989]
Omitting Tong, 2014	0.887	[0.797; 0.988]
Omitting Toraih, 2016	0.881	[0.792; 0.980]
Omitting Toraih, 2016	0.884	[0.794; 0.984]
Omitting Umar, 2013	0.882	[0.793; 0.982]
Omitting Vinci, 2013	0.884	[0.794; 0.984]
Omitting Vinci, 2011	0.883	[0.793; 0.982]
Omitting Wang, 2016	0.892	[0.801; 0.992]
Omitting Wang, 2014	0.884	[0.793; 0.984]
Omitting Wang, 2013	0.880	[0.791; 0.980]
Omitting Wang, 2010	0.895	[0.805; 0.995]
Omitting Wei, 2013	0.883	[0.793; 0.983]
Omitting Yan, 2015	0.894	[0.804; 0.994]
Omitting Yang, 2008	0.885	[0.795; 0.986]
Omitting Yin, 2017	0.891	[0.800; 0.992]
Omitting Yin, 2016	0.887	[0.796; 0.988]
Omitting Yin, 2015	0.887	[0.796; 0.987]
Omitting Zhan, 2011	0.891	[0.800; 0.991]
Omitting Zhang, 2017	0.883	[0.793; 0.983]
Omitting Zhang, 2016	0.886	[0.796; 0.987]
Omitting Zhang, 2013	0.889	[0.799; 0.990]
Omitting Zhang, 2012	0.883	[0.793; 0.982]
Omitting Zhao, 2016	0.885	[0.795; 0.985]
Omitting Zhou, 2014	0.892	[0.802; 0.993]
Omitting Zhou, 2011	0.887	[0.796; 0.987]
Omitting Zhu, 2012	0.890	[0.799; 0.991]
Omitting Kim, 2010	0.889	[0.798; 0.990]
**Random effects model**	**0.887**	**[0.797; 0.986]**

B)

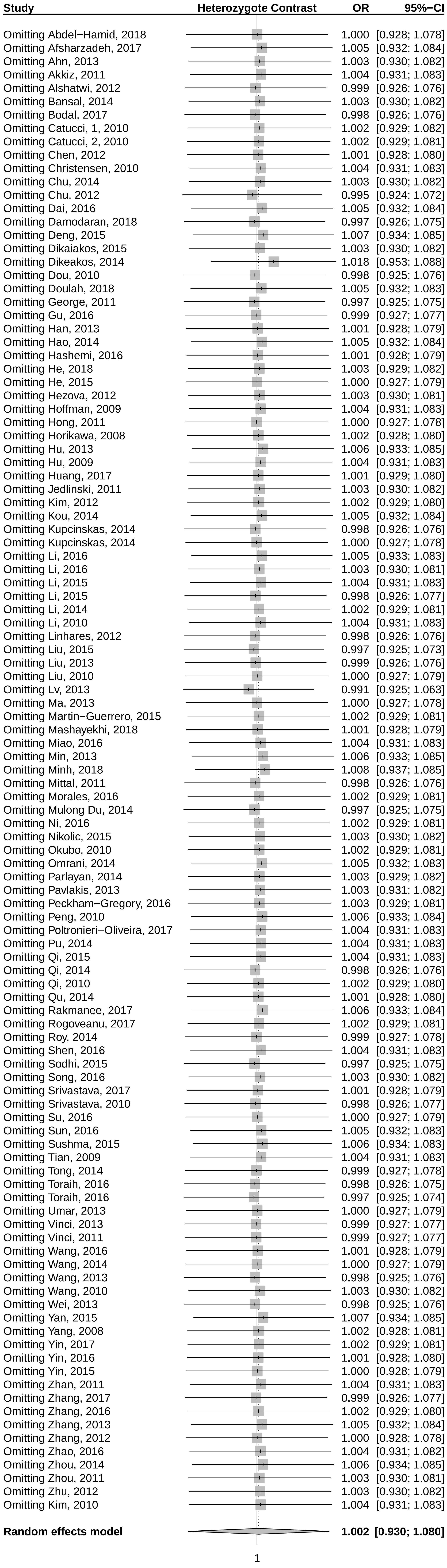
Study
Heterozygote Contrast
OR
95%−CI
Omitting Abdel−Hamid, 2018 1.000 [0.928; 1.078]
Omitting Afsharzadeh, 2017 1.005 [0.932; 1.084]
Omitting Ahn, 2013 1.003 [0.930; 1.082]
Omitting Akkiz, 2011 1.004 [0.931; 1.083]
Omitting Alshatwi, 2012 0.999 [0.926; 1.076]
Omitting Bansal, 2014 1.003 [0.930; 1.082]
Omitting Bodal, 2017 0.998 [0.926; 1.076]
Omitting Catucci, 1, 2010 1.002 [0.929; 1.082]
Omitting Catucci, 2, 2010 1.002 [0.929; 1.081]
Omitting Chen, 2012 1.001 [0.928; 1.080]
Omitting Christensen, 2010 1.004 [0.931; 1.083]
Omitting Chu, 2014 1.003 [0.930; 1.082]
Omitting Chu, 2012 0.995 [0.924; 1.072]
Omitting Dai, 2016 1.005 [0.932; 1.084]
Omitting Damodaran, 2018 0.997 [0.926; 1.075]
Omitting Deng, 2015 1.007 [0.934; 1.085]
Omitting Dikaiakos, 2015 1.003 [0.930; 1.082]
Omitting Dikeakos, 2014 1.018 [0.953; 1.088]
Omitting Dou, 2010 0.998 [0.925; 1.076]
Omitting Doulah, 2018 1.005 [0.932; 1.083]
Omitting George, 2011 0.997 [0.925; 1.075]
Omitting Gu, 2016 0.999 [0.927; 1.077]
Omitting Han, 2013 1.001 [0.928; 1.079]
Omitting Hao, 2014 1.005 [0.932; 1.084]
Omitting Hashemi, 2016 1.001 [0.928; 1.079]
Omitting He, 2018 1.003 [0.929; 1.082]
Omitting He, 2015 1.000 [0.927; 1.079]
Omitting Hezova, 2012 1.003 [0.930; 1.081]
Omitting Hoffman, 2009 1.004 [0.931; 1.083]
Omitting Hong, 2011 1.000 [0.927; 1.078]
Omitting Horikawa, 2008 1.002 [0.928; 1.080]
Omitting Hu, 2013 1.006 [0.933; 1.085]
Omitting Hu, 2009 1.004 [0.931; 1.083]
Omitting Huang, 2017 1.001 [0.929; 1.080]
Omitting Jedlinski, 2011 1.003 [0.930; 1.082]
Omitting Kim, 2012 1.002 [0.929; 1.080]
Omitting Kou, 2014 1.005 [0.932; 1.084]
Omitting Kupcinskas, 2014 0.998 [0.926; 1.076]
Omitting Kupcinskas, 2014 1.000 [0.927; 1.078]
Omitting Li, 2016 1.005 [0.933; 1.083]
Omitting Li, 2016 1.003 [0.930; 1.081]
Omitting Li, 2015 1.004 [0.931; 1.083]
Omitting Li, 2015 0.998 [0.926; 1.077]
Omitting Li, 2014 1.002 [0.929; 1.081]
Omitting Li, 2010 1.004 [0.931; 1.083]
Omitting Linhares, 2012 0.998 [0.926; 1.076]
Omitting Liu, 2015 0.997 [0.925; 1.073]
Omitting Liu, 2013 0.999 [0.926; 1.076]
Omitting Liu, 2010 1.000 [0.927; 1.079]
Omitting Lv, 2013 0.991 [0.925; 1.063]
Omitting Ma, 2013 1.000 [0.927; 1.078]
Omitting Martin−Guerrero, 2015 1.002 [0.929; 1.081]
Omitting Mashayekhi, 2018 1.001 [0.928; 1.079]
Omitting Miao, 2016 1.004 [0.931; 1.083]
Omitting Min, 2013 1.006 [0.933; 1.085]
Omitting Minh, 2018 1.008 [0.937; 1.085]
Omitting Mittal, 2011 0.998 [0.926; 1.076]
Omitting Morales, 2016 1.002 [0.929; 1.081]
Omitting Mulong Du, 2014 0.997 [0.925; 1.075]
Omitting Ni, 2016 1.002 [0.929; 1.081]
Omitting Nikolic, 2015 1.003 [0.930; 1.082]
Omitting Okubo, 2010 1.002 [0.929; 1.081]
Omitting Omrani, 2014 1.005 [0.932; 1.083]
Omitting Parlayan, 2014 1.003 [0.929; 1.082]
Omitting Pavlakis, 2013 1.003 [0.931; 1.082]
Omitting Peckham−Gregory, 2016 1.003 [0.929; 1.081]
Omitting Peng, 2010 1.006 [0.933; 1.084]
Omitting Poltronieri−Oliveira, 2017 1.004 [0.931; 1.083]
Omitting Pu, 2014 1.004 [0.931; 1.083]
Omitting Qi, 2015 1.004 [0.931; 1.083]
Omitting Qi, 2014 0.998 [0.926; 1.076]
Omitting Qi, 2010 1.002 [0.929; 1.080]
Omitting Qu, 2014 1.001 [0.928; 1.080]
Omitting Rakmanee, 2017 1.006 [0.933; 1.084]
Omitting Rogoveanu, 2017 1.002 [0.929; 1.081]
Omitting Roy, 2014 0.999 [0.927; 1.078]
Omitting Shen, 2016 1.004 [0.931; 1.083]
Omitting Sodhi, 2015 0.997 [0.925; 1.075]
Omitting Song, 2016 1.003 [0.930; 1.082]
Omitting Srivastava, 2017 1.001 [0.928; 1.079]
Omitting Srivastava, 2010 0.998 [0.926; 1.077]
Omitting Su, 2016 1.000 [0.927; 1.079]
Omitting Sun, 2016 1.005 [0.932; 1.083]
Omitting Sushma, 2015 1.006 [0.934; 1.083]
Omitting Tian, 2009 1.004 [0.931; 1.083]
Omitting Tong, 2014 0.999 [0.927; 1.078]
Omitting Toraih, 2016 0.998 [0.926; 1.075]
Omitting Toraih, 2016 0.997 [0.925; 1.074]
Omitting Umar, 2013 1.000 [0.927; 1.079]
Omitting Vinci, 2013 0.999 [0.927; 1.077]
Omitting Vinci, 2011 0.999 [0.927; 1.077]
Omitting Wang, 2016 1.001 [0.928; 1.079]
Omitting Wang, 2014 1.000 [0.927; 1.079]
Omitting Wang, 2013 0.998 [0.925; 1.076]
Omitting Wang, 2010 1.003 [0.930; 1.082]
Omitting Wei, 2013 0.998 [0.925; 1.076]
Omitting Yan, 2015 1.007 [0.934; 1.085]
Omitting Yang, 2008 1.002 [0.928; 1.081]
Omitting Yin, 2017 1.002 [0.929; 1.081]
Omitting Yin, 2016 1.001 [0.928; 1.080]
Omitting Yin, 2015 1.000 [0.928; 1.079]
Omitting Zhan, 2011 1.004 [0.931; 1.083]
Omitting Zhang, 2017 0.999 [0.926; 1.077]
Omitting Zhang, 2016 1.002 [0.929; 1.080]
Omitting Zhang, 2013 1.005 [0.932; 1.084]
Omitting Zhang, 2012 1.000 [0.928; 1.078]
Omitting Zhao, 2016 1.004 [0.931; 1.082]
Omitting Zhou, 2014 1.006 [0.934; 1.085]
Omitting Zhou, 2011 1.003 [0.930; 1.081]
Omitting Zhu, 2012 1.003 [0.930; 1.082]
Omitting Kim, 2010 1.004 [0.931; 1.083]
Random effects model 1.002 [0.930; 1.080]
1

C)

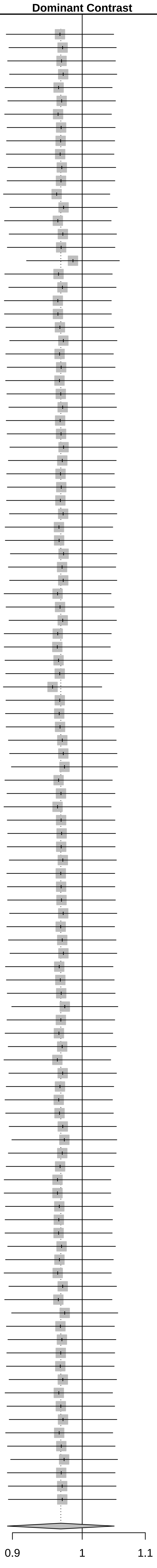

Study	OR	95%−CI
Omitting Abdel−Hamid, 2018	0.967	[0.892; 1.049]
Omitting Afsharzadeh, 2017	0.971	[0.895; 1.053]
Omitting Ahn, 2013	0.969	[0.893; 1.052]
Omitting Akkiz, 2011	0.972	[0.896; 1.054]
Omitting Alshatwi, 2012	0.965	[0.890; 1.046]
Omitting Bansal, 2014	0.969	[0.894; 1.052]
Omitting Bodal, 2017	0.964	[0.889; 1.045]
Omitting Catucci, 1, 2010	0.969	[0.893; 1.051]
Omitting Catucci, 2, 2010	0.968	[0.892; 1.050]
Omitting Chen, 2012	0.967	[0.892; 1.049]
Omitting Christensen, 2010	0.970	[0.894; 1.052]
Omitting Chu, 2014	0.968	[0.893; 1.051]
Omitting Chu, 2012	0.962	[0.888; 1.043]
Omitting Dai, 2016	0.972	[0.897; 1.054]
Omitting Damodaran, 2018	0.964	[0.889; 1.045]
Omitting Deng, 2015	0.971	[0.896; 1.053]
Omitting Dikaiakos, 2015	0.969	[0.893; 1.051]
Omitting Dikeakos, 2014	0.986	[0.919; 1.058]
Omitting Dou, 2010	0.965	[0.890; 1.046]
Omitting Doulah, 2018	0.971	[0.895; 1.053]
Omitting George, 2011	0.964	[0.889; 1.045]
Omitting Gu, 2016	0.964	[0.889; 1.045]
Omitting Han, 2013	0.967	[0.891; 1.049]
Omitting Hao, 2014	0.972	[0.896; 1.054]
Omitting Hashemi, 2016	0.966	[0.891; 1.048]
Omitting He, 2018	0.969	[0.893; 1.051]
Omitting He, 2015	0.966	[0.891; 1.048]
Omitting Hezova, 2012	0.968	[0.892; 1.050]
Omitting Hoffman, 2009	0.971	[0.895; 1.053]
Omitting Hong, 2011	0.967	[0.892; 1.049]
Omitting Horikawa, 2008	0.969	[0.893; 1.051]
Omitting Hu, 2013	0.972	[0.897; 1.054]
Omitting Hu, 2009	0.970	[0.894; 1.053]
Omitting Huang, 2017	0.968	[0.892; 1.050]
Omitting Jedlinski, 2011	0.969	[0.893; 1.051]
Omitting Kim, 2012	0.968	[0.892; 1.050]
Omitting Kou, 2014	0.972	[0.896; 1.054]
Omitting Kupcinskas, 2014	0.966	[0.890; 1.047]
Omitting Kupcinskas, 2014	0.966	[0.890; 1.048]
Omitting Li, 2016	0.972	[0.897; 1.054]
Omitting Li, 2016	0.970	[0.894; 1.052]
Omitting Li, 2015	0.972	[0.896; 1.054]
Omitting Li, 2015	0.964	[0.889; 1.045]
Omitting Li, 2014	0.967	[0.891; 1.049]
Omitting Li, 2010	0.971	[0.895; 1.053]
Omitting Linhares, 2012	0.964	[0.889; 1.045]
Omitting Liu, 2015	0.963	[0.889; 1.043]
Omitting Liu, 2013	0.965	[0.890; 1.046]
Omitting Liu, 2010	0.967	[0.891; 1.049]
Omitting Lv, 2013	0.956	[0.888; 1.030]
Omitting Ma, 2013	0.967	[0.891; 1.049]
Omitting Martin−Guerrero, 2015	0.966	[0.891; 1.048]
Omitting Mashayekhi, 2018	0.967	[0.891; 1.049]
Omitting Miao, 2016	0.970	[0.895; 1.053]
Omitting Min, 2013	0.972	[0.896; 1.054]
Omitting Minh, 2018	0.974	[0.899; 1.055]
Omitting Mittal, 2011	0.965	[0.890; 1.047]
Omitting Morales, 2016	0.968	[0.893; 1.051]
Omitting Mulong Du, 2014	0.963	[0.889; 1.045]
Omitting Ni, 2016	0.969	[0.893; 1.051]
Omitting Nikolic, 2015	0.969	[0.894; 1.052]
Omitting Okubo, 2010	0.969	[0.893; 1.051]
Omitting Omrani, 2014	0.971	[0.896; 1.053]
Omitting Parlayan, 2014	0.968	[0.892; 1.050]
Omitting Pavlakis, 2013	0.969	[0.893; 1.051]
Omitting Peckham−Gregory, 2016	0.969	[0.893; 1.051]
Omitting Peng, 2010	0.972	[0.896; 1.054]
Omitting Poltronieri−Oliveira, 2017	0.968	[0.892; 1.050]
Omitting Pu, 2014	0.970	[0.895; 1.052]
Omitting Qi, 2015	0.972	[0.897; 1.054]
Omitting Qi, 2014	0.966	[0.891; 1.048]
Omitting Qi, 2010	0.967	[0.892; 1.050]
Omitting Qu, 2014	0.969	[0.893; 1.051]
Omitting Rakmanee, 2017	0.974	[0.899; 1.055]
Omitting Rogoveanu, 2017	0.968	[0.893; 1.050]
Omitting Roy, 2014	0.965	[0.890; 1.047]
Omitting Shen, 2016	0.970	[0.894; 1.053]
Omitting Sodhi, 2015	0.963	[0.889; 1.044]
Omitting Song, 2016	0.971	[0.895; 1.053]
Omitting Srivastava, 2017	0.967	[0.892; 1.049]
Omitting Srivastava, 2010	0.965	[0.890; 1.047]
Omitting Su, 2016	0.966	[0.891; 1.048]
Omitting Sun, 2016	0.971	[0.896; 1.053]
Omitting Sushma, 2015	0.973	[0.899; 1.054]
Omitting Tian, 2009	0.970	[0.894; 1.053]
Omitting Tong, 2014	0.967	[0.891; 1.049]
Omitting Toraih, 2016	0.963	[0.889; 1.044]
Omitting Toraih, 2016	0.963	[0.889; 1.044]
Omitting Umar, 2013	0.966	[0.891; 1.048]
Omitting Vinci, 2013	0.965	[0.890; 1.047]
Omitting Vinci, 2011	0.965	[0.890; 1.046]
Omitting Wang, 2016	0.969	[0.894; 1.052]
Omitting Wang, 2014	0.966	[0.891; 1.048]
Omitting Wang, 2013	0.964	[0.889; 1.045]
Omitting Wang, 2010	0.971	[0.895; 1.053]
Omitting Wei, 2013	0.965	[0.890; 1.046]
Omitting Yan, 2015	0.974	[0.899; 1.055]
Omitting Yang, 2008	0.968	[0.892; 1.050]
Omitting Yin, 2017	0.970	[0.894; 1.052]
Omitting Yin, 2016	0.968	[0.892; 1.050]
Omitting Yin, 2015	0.967	[0.892; 1.049]
Omitting Zhan, 2011	0.971	[0.895; 1.053]
Omitting Zhang, 2017	0.965	[0.890; 1.047]
Omitting Zhang, 2016	0.968	[0.893; 1.050]
Omitting Zhang, 2013	0.971	[0.896; 1.054]
Omitting Zhang, 2012	0.966	[0.891; 1.047]
Omitting Zhao, 2016	0.969	[0.893; 1.051]
Omitting Zhou, 2014	0.973	[0.898; 1.055]
Omitting Zhou, 2011	0.969	[0.893; 1.051]
Omitting Zhu, 2012	0.970	[0.894; 1.053]
Omitting Kim, 2010	0.970	[0.894; 1.053]
**Random effects model**	**0.968**	**[0.893; 1.050]**

D)

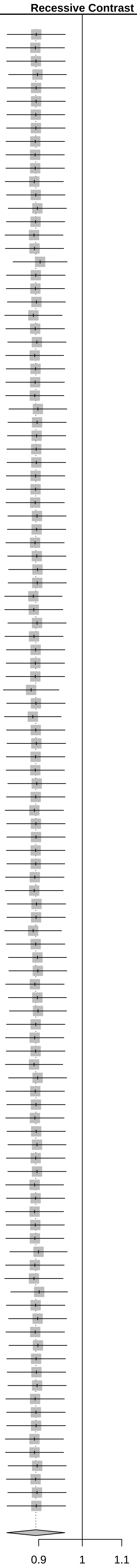

Study	OR	95%−CI
Omitting Abdel−Hamid, 2018	0.895	[0.834; 0.960]
Omitting Afsharzadeh, 2017	0.893	[0.832; 0.958]
Omitting Ahn, 2013	0.894	[0.833; 0.960]
Omitting Akkiz, 2011	0.897	[0.837; 0.962]
Omitting Alshatwi, 2012	0.894	[0.834; 0.959]
Omitting Bansal, 2014	0.894	[0.834; 0.960]
Omitting Bodal, 2017	0.894	[0.833; 0.959]
Omitting Catucci, 1, 2010	0.894	[0.833; 0.960]
Omitting Catucci, 2, 2010	0.893	[0.832; 0.958]
Omitting Chen, 2012	0.892	[0.832; 0.958]
Omitting Christensen, 2010	0.892	[0.832; 0.958]
Omitting Chu, 2014	0.891	[0.830; 0.955]
Omitting Chu, 2012	0.894	[0.833; 0.959]
Omitting Dai, 2016	0.897	[0.836; 0.963]
Omitting Damodaran, 2018	0.893	[0.833; 0.959]
Omitting Deng, 2015	0.890	[0.830; 0.955]
Omitting Dikaiakos, 2015	0.891	[0.831; 0.956]
Omitting Dikeakos, 2014	0.903	[0.846; 0.964]
Omitting Dou, 2010	0.894	[0.833; 0.959]
Omitting Doulah, 2018	0.893	[0.832; 0.958]
Omitting George, 2011	0.895	[0.835; 0.960]
Omitting Gu, 2016	0.889	[0.829; 0.952]
Omitting Han, 2013	0.893	[0.831; 0.958]
Omitting Hao, 2014	0.896	[0.835; 0.961]
Omitting Hashemi, 2016	0.891	[0.831; 0.956]
Omitting He, 2018	0.893	[0.832; 0.959]
Omitting He, 2015	0.892	[0.831; 0.958]
Omitting Hezova, 2012	0.892	[0.831; 0.956]
Omitting Hoffman, 2009	0.898	[0.838; 0.963]
Omitting Hong, 2011	0.897	[0.836; 0.962]
Omitting Horikawa, 2008	0.896	[0.835; 0.961]
Omitting Hu, 2013	0.895	[0.834; 0.960]
Omitting Hu, 2009	0.895	[0.834; 0.961]
Omitting Huang, 2017	0.893	[0.832; 0.959]
Omitting Jedlinski, 2011	0.893	[0.832; 0.959]
Omitting Kim, 2012	0.892	[0.832; 0.958]
Omitting Kou, 2014	0.896	[0.835; 0.962]
Omitting Kupcinskas, 2014	0.896	[0.835; 0.961]
Omitting Kupcinskas, 2014	0.892	[0.831; 0.957]
Omitting Li, 2016	0.896	[0.835; 0.961]
Omitting Li, 2016	0.897	[0.837; 0.962]
Omitting Li, 2015	0.897	[0.836; 0.962]
Omitting Li, 2015	0.888	[0.829; 0.952]
Omitting Li, 2014	0.889	[0.829; 0.954]
Omitting Li, 2010	0.896	[0.836; 0.962]
Omitting Linhares, 2012	0.890	[0.830; 0.955]
Omitting Liu, 2015	0.893	[0.833; 0.959]
Omitting Liu, 2013	0.893	[0.832; 0.958]
Omitting Liu, 2010	0.893	[0.832; 0.958]
Omitting Lv, 2013	0.884	[0.826; 0.945]
Omitting Ma, 2013	0.894	[0.833; 0.960]
Omitting Martin−Guerrero, 2015	0.887	[0.829; 0.950]
Omitting Mashayekhi, 2018	0.894	[0.833; 0.959]
Omitting Miao, 2016	0.895	[0.834; 0.961]
Omitting Min, 2013	0.893	[0.832; 0.959]
Omitting Minh, 2018	0.893	[0.832; 0.958]
Omitting Mittal, 2011	0.896	[0.835; 0.961]
Omitting Morales, 2016	0.894	[0.833; 0.959]
Omitting Mulong Du, 2014	0.891	[0.830; 0.956]
Omitting Ni, 2016	0.894	[0.833; 0.959]
Omitting Nikolic, 2015	0.894	[0.833; 0.960]
Omitting Okubo, 2010	0.894	[0.832; 0.959]
Omitting Omrani, 2014	0.894	[0.833; 0.959]
Omitting Parlayan, 2014	0.892	[0.831; 0.957]
Omitting Pavlakis, 2013	0.890	[0.830; 0.955]
Omitting Peckham−Gregory, 2016	0.895	[0.834; 0.961]
Omitting Peng, 2010	0.894	[0.833; 0.960]
Omitting Poltronieri−Oliveira, 2017	0.888	[0.829; 0.951]
Omitting Pu, 2014	0.894	[0.833; 0.959]
Omitting Qi, 2015	0.897	[0.836; 0.963]
Omitting Qi, 2014	0.898	[0.838; 0.963]
Omitting Qi, 2010	0.892	[0.831; 0.957]
Omitting Qu, 2014	0.897	[0.836; 0.962]
Omitting Rakmanee, 2017	0.899	[0.839; 0.963]
Omitting Rogoveanu, 2017	0.894	[0.833; 0.959]
Omitting Roy, 2014	0.891	[0.831; 0.956]
Omitting Shen, 2016	0.893	[0.832; 0.959]
Omitting Sodhi, 2015	0.890	[0.831; 0.954]
Omitting Song, 2016	0.898	[0.837; 0.963]
Omitting Srivastava, 2017	0.893	[0.832; 0.958]
Omitting Srivastava, 2010	0.894	[0.833; 0.959]
Omitting Su, 2016	0.892	[0.831; 0.957]
Omitting Sun, 2016	0.895	[0.834; 0.960]
Omitting Sushma, 2015	0.896	[0.836; 0.961]
Omitting Tian, 2009	0.894	[0.832; 0.959]
Omitting Tong, 2014	0.896	[0.835; 0.962]
Omitting Toraih, 2016	0.891	[0.831; 0.956]
Omitting Toraih, 2016	0.893	[0.833; 0.959]
Omitting Umar, 2013	0.891	[0.831; 0.956]
Omitting Vinci, 2013	0.893	[0.832; 0.958]
Omitting Vinci, 2011	0.892	[0.831; 0.957]
Omitting Wang, 2016	0.900	[0.839; 0.964]
Omitting Wang, 2014	0.892	[0.831; 0.957]
Omitting Wang, 2013	0.890	[0.829; 0.955]
Omitting Wang, 2010	0.901	[0.841; 0.965]
Omitting Wei, 2013	0.893	[0.832; 0.959]
Omitting Yan, 2015	0.898	[0.837; 0.963]
Omitting Yang, 2008	0.893	[0.832; 0.958]
Omitting Yin, 2017	0.898	[0.838; 0.964]
Omitting Yin, 2016	0.894	[0.833; 0.960]
Omitting Yin, 2015	0.895	[0.834; 0.961]
Omitting Zhan, 2011	0.897	[0.836; 0.962]
Omitting Zhang, 2017	0.892	[0.831; 0.958]
Omitting Zhang, 2016	0.894	[0.833; 0.959]
Omitting Zhang, 2013	0.894	[0.833; 0.960]
Omitting Zhang, 2012	0.891	[0.830; 0.956]
Omitting Zhao, 2016	0.891	[0.831; 0.956]
Omitting Zhou, 2014	0.897	[0.836; 0.962]
Omitting Zhou, 2011	0.893	[0.832; 0.959]
Omitting Zhu, 2012	0.896	[0.835; 0.962]
Omitting Kim, 2010	0.895	[0.834; 0.961]
**Random effects model**	**0.894**	**[0.833; 0.959]**

E)

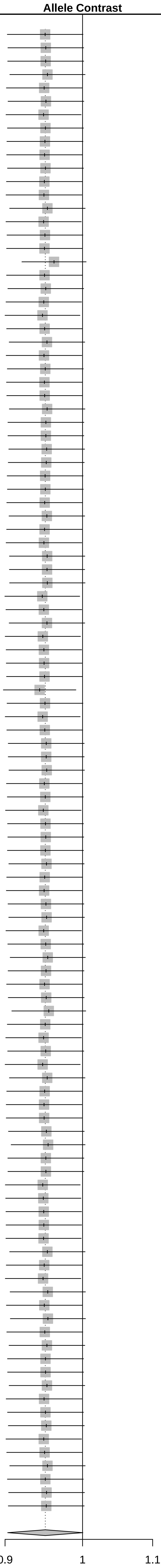

Study	OR	95%−CI
Omitting Abdel−Hamid, 2018	0.950	[0.903; 1.001]
Omitting Afsharzadeh, 2017	0.951	[0.904; 1.002]
Omitting Ahn, 2013	0.951	[0.903; 1.001]
Omitting Akkiz, 2011	0.953	[0.906; 1.003]
Omitting Alshatwi, 2012	0.949	[0.902; 0.999]
Omitting Bansal, 2014	0.951	[0.904; 1.002]
Omitting Bodal, 2017	0.948	[0.901; 0.998]
Omitting Catucci, 1, 2010	0.951	[0.903; 1.001]
Omitting Catucci, 2, 2010	0.950	[0.902; 1.001]
Omitting Chen, 2012	0.950	[0.902; 1.000]
Omitting Christensen, 2010	0.951	[0.903; 1.001]
Omitting Chu, 2014	0.949	[0.902; 1.000]
Omitting Chu, 2012	0.949	[0.901; 0.999]
Omitting Dai, 2016	0.953	[0.906; 1.004]
Omitting Damodaran, 2018	0.948	[0.901; 0.998]
Omitting Deng, 2015	0.950	[0.902; 1.000]
Omitting Dikaiakos, 2015	0.950	[0.902; 1.000]
Omitting Dikeakos, 2014	0.962	[0.921; 1.005]
Omitting Dou, 2010	0.950	[0.902; 1.000]
Omitting Doulah, 2018	0.951	[0.903; 1.001]
Omitting George, 2011	0.949	[0.901; 0.999]
Omitting Gu, 2016	0.947	[0.900; 0.996]
Omitting Han, 2013	0.950	[0.902; 1.000]
Omitting Hao, 2014	0.953	[0.905; 1.003]
Omitting Hashemi, 2016	0.949	[0.901; 0.999]
Omitting He, 2018	0.951	[0.903; 1.001]
Omitting He, 2015	0.949	[0.902; 1.000]
Omitting Hezova, 2012	0.950	[0.902; 1.000]
Omitting Hoffman, 2009	0.953	[0.905; 1.003]
Omitting Hong, 2011	0.951	[0.904; 1.002]
Omitting Horikawa, 2008	0.951	[0.904; 1.002]
Omitting Hu, 2013	0.952	[0.905; 1.003]
Omitting Hu, 2009	0.952	[0.904; 1.002]
Omitting Huang, 2017	0.950	[0.903; 1.001]
Omitting Jedlinski, 2011	0.951	[0.903; 1.001]
Omitting Kim, 2012	0.950	[0.902; 1.000]
Omitting Kou, 2014	0.953	[0.905; 1.003]
Omitting Kupcinskas, 2014	0.950	[0.902; 1.000]
Omitting Kupcinskas, 2014	0.949	[0.901; 0.999]
Omitting Li, 2016	0.953	[0.905; 1.003]
Omitting Li, 2016	0.953	[0.905; 1.003]
Omitting Li, 2015	0.953	[0.906; 1.003]
Omitting Li, 2015	0.947	[0.900; 0.996]
Omitting Li, 2014	0.949	[0.901; 0.999]
Omitting Li, 2010	0.953	[0.905; 1.003]
Omitting Linhares, 2012	0.947	[0.900; 0.997]
Omitting Liu, 2015	0.949	[0.901; 0.999]
Omitting Liu, 2013	0.949	[0.901; 0.999]
Omitting Liu, 2010	0.950	[0.902; 1.000]
Omitting Lv, 2013	0.943	[0.898; 0.991]
Omitting Ma, 2013	0.950	[0.902; 1.000]
Omitting Martin−Guerrero, 2015	0.947	[0.900; 0.997]
Omitting Mashayekhi, 2018	0.950	[0.902; 1.000]
Omitting Miao, 2016	0.952	[0.904; 1.002]
Omitting Min, 2013	0.952	[0.904; 1.002]
Omitting Minh, 2018	0.952	[0.905; 1.003]
Omitting Mittal, 2011	0.949	[0.902; 1.000]
Omitting Morales, 2016	0.951	[0.903; 1.001]
Omitting Mulong Du, 2014	0.948	[0.901; 0.998]
Omitting Ni, 2016	0.951	[0.903; 1.001]
Omitting Nikolic, 2015	0.951	[0.903; 1.002]
Omitting Okubo, 2010	0.951	[0.903; 1.001]
Omitting Omrani, 2014	0.952	[0.905; 1.002]
Omitting Parlayan, 2014	0.950	[0.902; 1.000]
Omitting Pavlakis, 2013	0.949	[0.902; 0.999]
Omitting Peckham−Gregory, 2016	0.951	[0.904; 1.002]
Omitting Peng, 2010	0.952	[0.905; 1.003]
Omitting Poltronieri−Oliveira, 2017	0.949	[0.901; 0.999]
Omitting Pu, 2014	0.951	[0.903; 1.002]
Omitting Qi, 2015	0.954	[0.906; 1.004]
Omitting Qi, 2014	0.952	[0.904; 1.002]
Omitting Qi, 2010	0.950	[0.902; 1.000]
Omitting Qu, 2014	0.952	[0.904; 1.002]
Omitting Rakmanee, 2017	0.955	[0.908; 1.004]
Omitting Rogoveanu, 2017	0.951	[0.903; 1.001]
Omitting Roy, 2014	0.948	[0.901; 0.998]
Omitting Shen, 2016	0.951	[0.903; 1.002]
Omitting Sodhi, 2015	0.947	[0.900; 0.997]
Omitting Song, 2016	0.953	[0.905; 1.003]
Omitting Srivastava, 2017	0.950	[0.902; 1.000]
Omitting Srivastava, 2010	0.949	[0.901; 0.999]
Omitting Su, 2016	0.949	[0.901; 0.999]
Omitting Sun, 2016	0.952	[0.904; 1.002]
Omitting Sushma, 2015	0.954	[0.907; 1.003]
Omitting Tian, 2009	0.951	[0.903; 1.002]
Omitting Tong, 2014	0.951	[0.904; 1.002]
Omitting Toraih, 2016	0.947	[0.900; 0.997]
Omitting Toraih, 2016	0.948	[0.901; 0.998]
Omitting Umar, 2013	0.949	[0.901; 0.999]
Omitting Vinci, 2013	0.949	[0.901; 0.999]
Omitting Vinci, 2011	0.948	[0.901; 0.998]
Omitting Wang, 2016	0.953	[0.906; 1.003]
Omitting Wang, 2014	0.949	[0.902; 0.999]
Omitting Wang, 2013	0.948	[0.900; 0.998]
Omitting Wang, 2010	0.954	[0.906; 1.004]
Omitting Wei, 2013	0.949	[0.902; 1.000]
Omitting Yan, 2015	0.954	[0.907; 1.004]
Omitting Yang, 2008	0.950	[0.902; 1.000]
Omitting Yin, 2017	0.953	[0.905; 1.003]
Omitting Yin, 2016	0.951	[0.903; 1.001]
Omitting Yin, 2015	0.951	[0.903; 1.001]
Omitting Zhan, 2011	0.953	[0.905; 1.003]
Omitting Zhang, 2017	0.949	[0.901; 0.999]
Omitting Zhang, 2016	0.951	[0.903; 1.001]
Omitting Zhang, 2013	0.952	[0.904; 1.002]
Omitting Zhang, 2012	0.949	[0.901; 0.999]
Omitting Zhao, 2016	0.950	[0.902; 1.000]
Omitting Zhou, 2014	0.953	[0.906; 1.004]
Omitting Zhou, 2011	0.951	[0.903; 1.001]
Omitting Zhu, 2012	0.952	[0.904; 1.003]
Omitting Kim, 2010	0.952	[0.904; 1.002]
**Random effects model**	**0.951**	**[0.903; 1.000]**
