# Supplementary material for: Association of mir-196a-2 rs11614913 and mir-149 rs2292832 Polymorphisms With Risk of Cancer: An Updated Meta-Analysis
Source: Front Genet. 2019 Mar 15;10:186. doi: 10.3389/fgene.2019.00186 (PMC6429108; doi:10.3389/fgene.2019.00186)
Supplement: Supplementary file 1 [file Data_Sheet_1.ZIP › Supplementary Figures/Suppl. fig. S9.pdf]

Supplementary Figure S9. Sensitivity analyses for meta-analysis of miR-149 rs2292832 and cancer risk under different genetic models in order to detect potential influential studies using leave-one-out approach. **A)** Homozygote contrast using trim & fill (TAF) approach; **B)** Homozygote contrast using original studies; **C)** Heterozygote; **D)** Dominant model; **E)** Recessive model using trim & fill approach **F)** Recessive model using original studies; **G)** allele contrast using trim & fill approach; **H)** Allele contrast using original studies. Each line represents the overall estimate while omitting one study

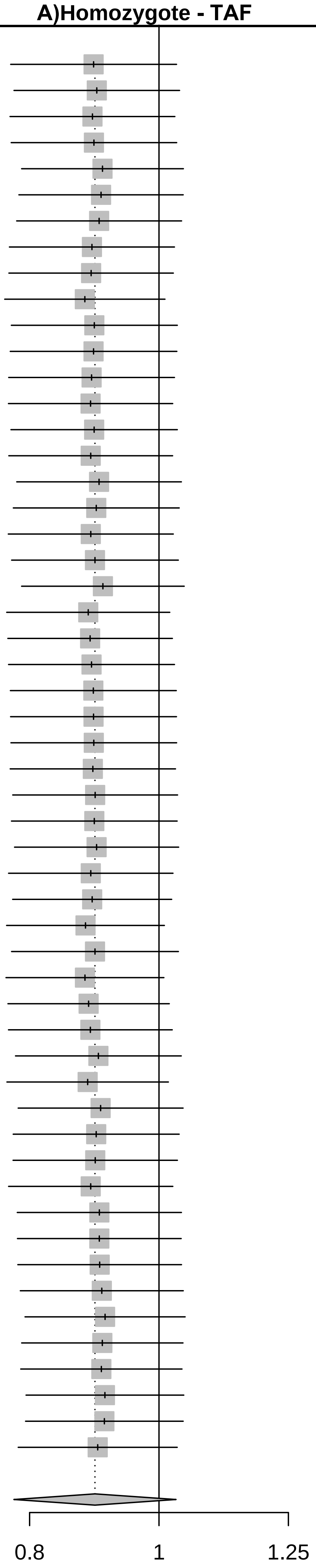

Study	OR	95%−CI
Omitting Ahn, 2012	0.893	[0.774; 1.031]
Omitting Chu, 2012	0.898	[0.779; 1.036]
Omitting Chu, 2014	0.892	[0.773; 1.028]
Omitting Cîmpeanu, 2017	0.894	[0.775; 1.031]
Omitting Dikeakos, 2014	0.907	[0.789; 1.043]
Omitting Dong, 2015	0.905	[0.785; 1.043]
Omitting Du, 2014	0.902	[0.782; 1.040]
Omitting Gu, 2016	0.891	[0.773; 1.027]
Omitting Hashemi, 2016	0.890	[0.772; 1.025]
Omitting He, 2015	0.880	[0.766; 1.010]
Omitting He, 2018	0.894	[0.775; 1.032]
Omitting Hu, 2009	0.893	[0.774; 1.031]
Omitting Hu, 2013	0.890	[0.772; 1.027]
Omitting Huang, 2013	0.889	[0.771; 1.024]
Omitting Jiang, 2016	0.894	[0.775; 1.032]
Omitting Kim, 2012	0.889	[0.772; 1.024]
Omitting Kontorovich, 2010	0.902	[0.782; 1.039]
Omitting Kou, 2014	0.897	[0.778; 1.035]
Omitting Li, 2016	0.889	[0.771; 1.025]
Omitting Liu, 2010	0.895	[0.776; 1.034]
Omitting Liu, 2014	0.908	[0.789; 1.044]
Omitting Liu, 2015	0.885	[0.769; 1.019]
Omitting Lv, 2013	0.888	[0.771; 1.023]
Omitting Miao, 2016	0.890	[0.772; 1.027]
Omitting Min, 2012	0.893	[0.774; 1.030]
Omitting Mw Zhang−a, 2012	0.893	[0.774; 1.031]
Omitting Mw Zhang−b, 2012	0.894	[0.775; 1.031]
Omitting Ni, 2016	0.892	[0.774; 1.029]
Omitting Pratedrat, 2015	0.896	[0.777; 1.033]
Omitting Pu, 2014	0.894	[0.775; 1.032]
Omitting Ranjbar, 2018	0.898	[0.780; 1.034]
Omitting Su, 2016	0.889	[0.772; 1.024]
Omitting Sushma, 2015	0.891	[0.777; 1.022]
Omitting Tandon, 2017	0.881	[0.769; 1.009]
Omitting Tian, 2009	0.896	[0.776; 1.034]
Omitting Tu, 2012	0.880	[0.768; 1.008]
Omitting Vinci, 2011	0.886	[0.771; 1.018]
Omitting Vinci, 2013	0.888	[0.771; 1.023]
Omitting Wang, 2014	0.901	[0.781; 1.039]
Omitting Wang, 2014	0.884	[0.769; 1.016]
Omitting Wei, 2014	0.904	[0.784; 1.042]
Omitting Yan, 2015	0.897	[0.778; 1.035]
Omitting Yang, 2016	0.896	[0.778; 1.032]
Omitting Zhang, 2012	0.889	[0.772; 1.024]
Omitting Filled: Zhang, 2012	0.902	[0.783; 1.039]
Omitting Filled: Kim, 2012	0.902	[0.783; 1.039]
Omitting Filled: Vinci, 2013	0.903	[0.784; 1.040]
Omitting Filled: Liu, 2015	0.906	[0.787; 1.043]
Omitting Filled: He, 2015	0.911	[0.794; 1.046]
Omitting Filled: Wang, 2014	0.907	[0.789; 1.042]
Omitting Filled: Vinci, 2011	0.905	[0.788; 1.040]
Omitting Filled: Tu, 2012	0.911	[0.795; 1.044]
Omitting Filled: Tandon, 2017	0.910	[0.794; 1.043]
Omitting Filled: Sushma, 2015	0.900	[0.784; 1.032]
**Random effects model**	**0.895**	**[0.779; 1.030]**

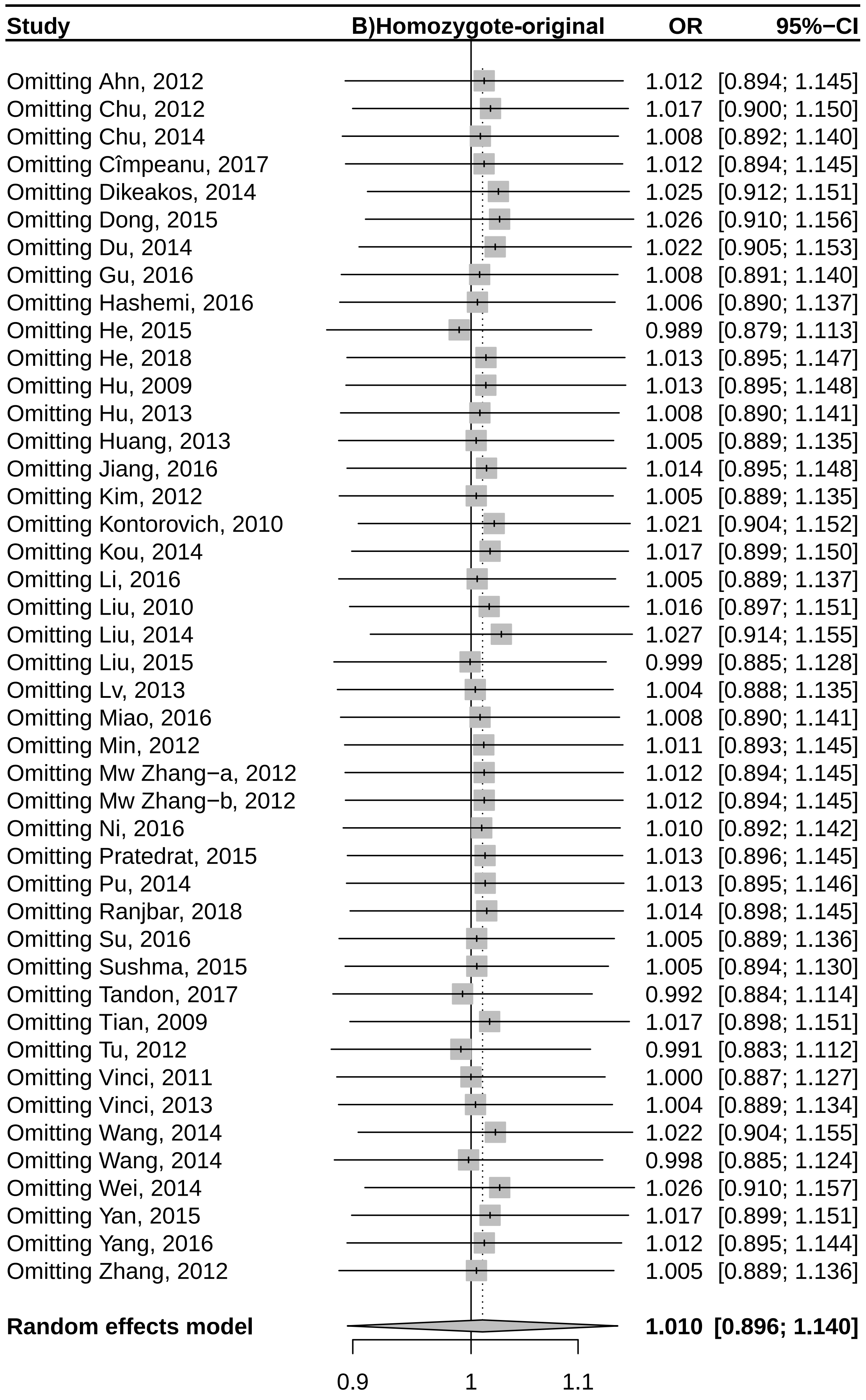
Study
B)Homozygote-original
OR
95%−CI
Omitting Ahn, 2012 1.012 [0.894; 1.145]
Omitting Chu, 2012 1.017 [0.900; 1.150]
Omitting Chu, 2014 1.008 [0.892; 1.140]
Omitting Cîmpeanu, 2017 1.012 [0.894; 1.145]
Omitting Dikeakos, 2014 1.025 [0.912; 1.151]
Omitting Dong, 2015 1.026 [0.910; 1.156]
Omitting Du, 2014 1.022 [0.905; 1.153]
Omitting Gu, 2016 1.008 [0.891; 1.140]
Omitting Hashemi, 2016 1.006 [0.890; 1.137]
Omitting He, 2015 0.989 [0.879; 1.113]
Omitting He, 2018 1.013 [0.895; 1.147]
Omitting Hu, 2009 1.013 [0.895; 1.148]
Omitting Hu, 2013 1.008 [0.890; 1.141]
Omitting Huang, 2013 1.005 [0.889; 1.135]
Omitting Jiang, 2016 1.014 [0.895; 1.148]
Omitting Kim, 2012 1.005 [0.889; 1.135]
Omitting Kontorovich, 2010 1.021 [0.904; 1.152]
Omitting Kou, 2014 1.017 [0.899; 1.150]
Omitting Li, 2016 1.005 [0.889; 1.137]
Omitting Liu, 2010 1.016 [0.897; 1.151]
Omitting Liu, 2014 1.027 [0.914; 1.155]
Omitting Liu, 2015 0.999 [0.885; 1.128]
Omitting Lv, 2013 1.004 [0.888; 1.135]
Omitting Miao, 2016 1.008 [0.890; 1.141]
Omitting Min, 2012 1.011 [0.893; 1.145]
Omitting Mw Zhang−a, 2012 1.012 [0.894; 1.145]
Omitting Mw Zhang−b, 2012 1.012 [0.894; 1.145]
Omitting Ni, 2016 1.010 [0.892; 1.142]
Omitting Pratedrat, 2015 1.013 [0.896; 1.145]
Omitting Pu, 2014 1.013 [0.895; 1.146]
Omitting Ranjbar, 2018 1.014 [0.898; 1.145]
Omitting Su, 2016 1.005 [0.889; 1.136]
Omitting Sushma, 2015 1.005 [0.894; 1.130]
Omitting Tandon, 2017 0.992 [0.884; 1.114]
Omitting Tian, 2009 1.017 [0.898; 1.151]
Omitting Tu, 2012 0.991 [0.883; 1.112]
Omitting Vinci, 2011 1.000 [0.887; 1.127]
Omitting Vinci, 2013 1.004 [0.889; 1.134]
Omitting Wang, 2014 1.022 [0.904; 1.155]
Omitting Wang, 2014 0.998 [0.885; 1.124]
Omitting Wei, 2014 1.026 [0.910; 1.157]
Omitting Yan, 2015 1.017 [0.899; 1.151]
Omitting Yang, 2016 1.012 [0.895; 1.144]
Omitting Zhang, 2012 1.005 [0.889; 1.136]
Random effects model 1.010 [0.896; 1.140]
0.9
1
1.1

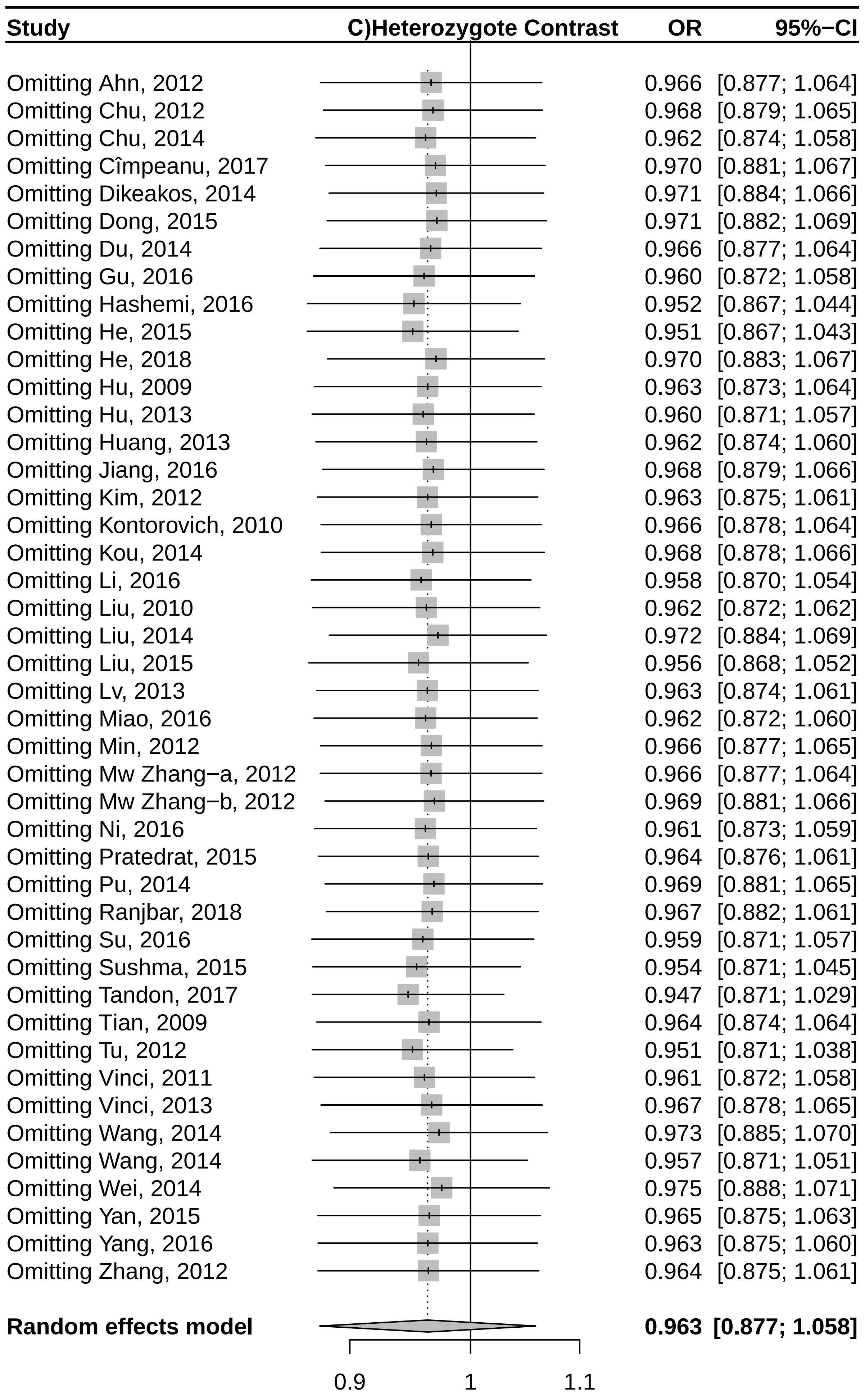
Study
C)Heterozygote Contrast
OR
95%−CI
Omitting Ahn, 2012 0.966 [0.877; 1.064]
Omitting Chu, 2012 0.968 [0.879; 1.065]
Omitting Chu, 2014 0.962 [0.874; 1.058]
Omitting Cîmpeanu, 2017 0.970 [0.881; 1.067]
Omitting Dikeakos, 2014 0.971 [0.884; 1.066]
Omitting Dong, 2015 0.971 [0.882; 1.069]
Omitting Du, 2014 0.966 [0.877; 1.064]
Omitting Gu, 2016 0.960 [0.872; 1.058]
Omitting Hashemi, 2016 0.952 [0.867; 1.044]
Omitting He, 2015 0.951 [0.867; 1.043]
Omitting He, 2018 0.970 [0.883; 1.067]
Omitting Hu, 2009 0.963 [0.873; 1.064]
Omitting Hu, 2013 0.960 [0.871; 1.057]
Omitting Huang, 2013 0.962 [0.874; 1.060]
Omitting Jiang, 2016 0.968 [0.879; 1.066]
Omitting Kim, 2012 0.963 [0.875; 1.061]
Omitting Kontorovich, 2010 0.966 [0.878; 1.064]
Omitting Kou, 2014 0.968 [0.878; 1.066]
Omitting Li, 2016 0.958 [0.870; 1.054]
Omitting Liu, 2010 0.962 [0.872; 1.062]
Omitting Liu, 2014 0.972 [0.884; 1.069]
Omitting Liu, 2015 0.956 [0.868; 1.052]
Omitting Lv, 2013 0.963 [0.874; 1.061]
Omitting Miao, 2016 0.962 [0.872; 1.060]
Omitting Min, 2012 0.966 [0.877; 1.065]
Omitting Mw Zhang−a, 2012 0.966 [0.877; 1.064]
Omitting Mw Zhang−b, 2012 0.969 [0.881; 1.066]
Omitting Ni, 2016 0.961 [0.873; 1.059]
Omitting Pratedrat, 2015 0.964 [0.876; 1.061]
Omitting Pu, 2014 0.969 [0.881; 1.065]
Omitting Ranjbar, 2018 0.967 [0.882; 1.061]
Omitting Su, 2016 0.959 [0.871; 1.057]
Omitting Sushma, 2015 0.954 [0.871; 1.045]
Omitting Tandon, 2017 0.947 [0.871; 1.029]
Omitting Tian, 2009 0.964 [0.874; 1.064]
Omitting Tu, 2012 0.951 [0.871; 1.038]
Omitting Vinci, 2011 0.961 [0.872; 1.058]
Omitting Vinci, 2013 0.967 [0.878; 1.065]
Omitting Wang, 2014 0.973 [0.885; 1.070]
Omitting Wang, 2014 0.957 [0.871; 1.051]
Omitting Wei, 2014 0.975 [0.888; 1.071]
Omitting Yan, 2015 0.965 [0.875; 1.063]
Omitting Yang, 2016 0.963 [0.875; 1.060]
Omitting Zhang, 2012 0.964 [0.875; 1.061]
Random effects model 0.963 [0.877; 1.058]
0.9
1
1.1

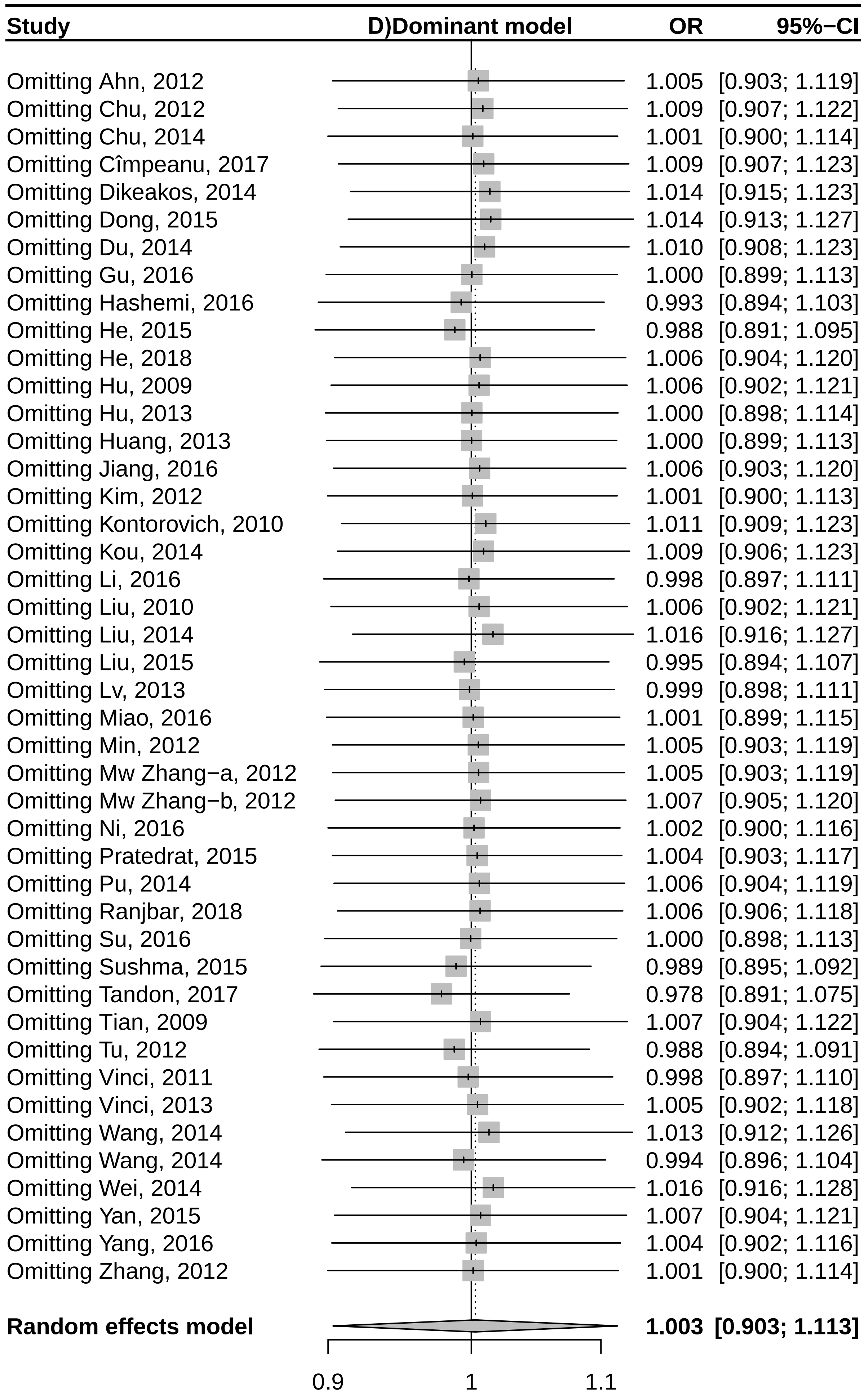
Study
D)Dominant model
OR
95%−CI
Omitting Ahn, 2012 1.005 [0.903; 1.119]
Omitting Chu, 2012 1.009 [0.907; 1.122]
Omitting Chu, 2014 1.001 [0.900; 1.114]
Omitting Cîmpeanu, 2017 1.009 [0.907; 1.123]
Omitting Dikeakos, 2014 1.014 [0.915; 1.123]
Omitting Dong, 2015 1.014 [0.913; 1.127]
Omitting Du, 2014 1.010 [0.908; 1.123]
Omitting Gu, 2016 1.000 [0.899; 1.113]
Omitting Hashemi, 2016 0.993 [0.894; 1.103]
Omitting He, 2015 0.988 [0.891; 1.095]
Omitting He, 2018 1.006 [0.904; 1.120]
Omitting Hu, 2009 1.006 [0.902; 1.121]
Omitting Hu, 2013 1.000 [0.898; 1.114]
Omitting Huang, 2013 1.000 [0.899; 1.113]
Omitting Jiang, 2016 1.006 [0.903; 1.120]
Omitting Kim, 2012 1.001 [0.900; 1.113]
Omitting Kontorovich, 2010 1.011 [0.909; 1.123]
Omitting Kou, 2014 1.009 [0.906; 1.123]
Omitting Li, 2016 0.998 [0.897; 1.111]
Omitting Liu, 2010 1.006 [0.902; 1.121]
Omitting Liu, 2014 1.016 [0.916; 1.127]
Omitting Liu, 2015 0.995 [0.894; 1.107]
Omitting Lv, 2013 0.999 [0.898; 1.111]
Omitting Miao, 2016 1.001 [0.899; 1.115]
Omitting Min, 2012 1.005 [0.903; 1.119]
Omitting Mw Zhang−a, 2012 1.005 [0.903; 1.119]
Omitting Mw Zhang−b, 2012 1.007 [0.905; 1.120]
Omitting Ni, 2016 1.002 [0.900; 1.116]
Omitting Pratedrat, 2015 1.004 [0.903; 1.117]
Omitting Pu, 2014 1.006 [0.904; 1.119]
Omitting Ranjbar, 2018 1.006 [0.906; 1.118]
Omitting Su, 2016 1.000 [0.898; 1.113]
Omitting Sushma, 2015 0.989 [0.895; 1.092]
Omitting Tandon, 2017 0.978 [0.891; 1.075]
Omitting Tian, 2009 1.007 [0.904; 1.122]
Omitting Tu, 2012 0.988 [0.894; 1.091]
Omitting Vinci, 2011 0.998 [0.897; 1.110]
Omitting Vinci, 2013 1.005 [0.902; 1.118]
Omitting Wang, 2014 1.013 [0.912; 1.126]
Omitting Wang, 2014 0.994 [0.896; 1.104]
Omitting Wei, 2014 1.016 [0.916; 1.128]
Omitting Yan, 2015 1.007 [0.904; 1.121]
Omitting Yang, 2016 1.004 [0.902; 1.116]
Omitting Zhang, 2012 1.001 [0.900; 1.114]
Random effects model 1.003 [0.903; 1.113]
0.9
1
1.1

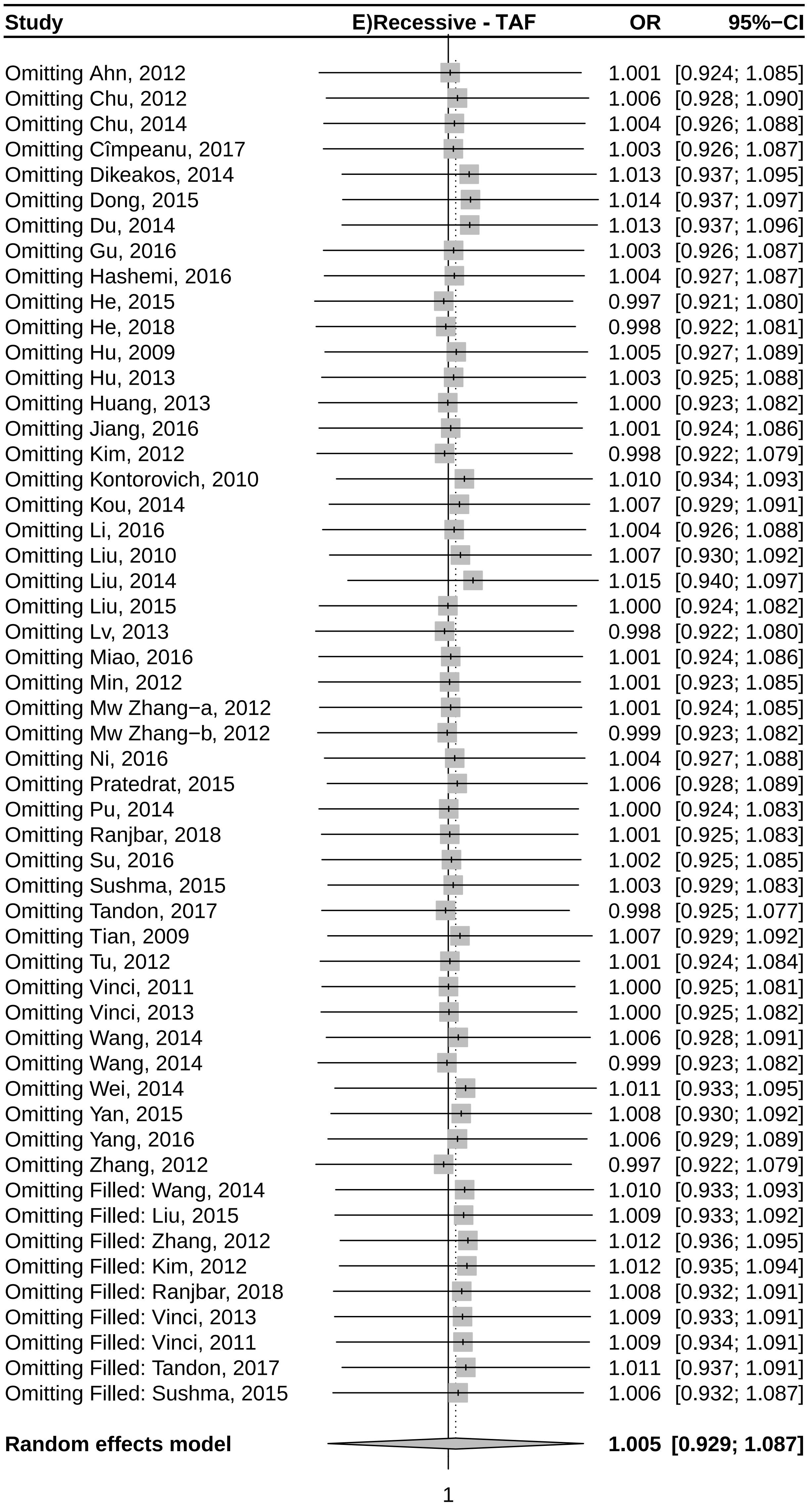

Study
E)Recessive - TAF
OR
95%−CI
Omitting Ahn, 2012 1.001 [0.924; 1.085]
Omitting Chu, 2012 1.006 [0.928; 1.090]
Omitting Chu, 2014 1.004 [0.926; 1.088]
Omitting Cîmpeanu, 2017 1.003 [0.926; 1.087]
Omitting Dikeakos, 2014 1.013 [0.937; 1.095]
Omitting Dong, 2015 1.014 [0.937; 1.097]
Omitting Du, 2014 1.013 [0.937; 1.096]
Omitting Gu, 2016 1.003 [0.926; 1.087]
Omitting Hashemi, 2016 1.004 [0.927; 1.087]
Omitting He, 2015 0.997 [0.921; 1.080]
Omitting He, 2018 0.998 [0.922; 1.081]
Omitting Hu, 2009 1.005 [0.927; 1.089]
Omitting Hu, 2013 1.003 [0.925; 1.088]
Omitting Huang, 2013 1.000 [0.923; 1.082]
Omitting Jiang, 2016 1.001 [0.924; 1.086]
Omitting Kim, 2012 0.998 [0.922; 1.079]
Omitting Kontorovich, 2010 1.010 [0.934; 1.093]
Omitting Kou, 2014 1.007 [0.929; 1.091]
Omitting Li, 2016 1.004 [0.926; 1.088]
Omitting Liu, 2010 1.007 [0.930; 1.092]
Omitting Liu, 2014 1.015 [0.940; 1.097]
Omitting Liu, 2015 1.000 [0.924; 1.082]
Omitting Lv, 2013 0.998 [0.922; 1.080]
Omitting Miao, 2016 1.001 [0.924; 1.086]
Omitting Min, 2012 1.001 [0.923; 1.085]
Omitting Mw Zhang−a, 2012 1.001 [0.924; 1.085]
Omitting Mw Zhang−b, 2012 0.999 [0.923; 1.082]
Omitting Ni, 2016 1.004 [0.927; 1.088]
Omitting Pratedrat, 2015 1.006 [0.928; 1.089]
Omitting Pu, 2014 1.000 [0.924; 1.083]
Omitting Ranjbar, 2018 1.001 [0.925; 1.083]
Omitting Su, 2016 1.002 [0.925; 1.085]
Omitting Sushma, 2015 1.003 [0.929; 1.083]
Omitting Tandon, 2017 0.998 [0.925; 1.077]
Omitting Tian, 2009 1.007 [0.929; 1.092]
Omitting Tu, 2012 1.001 [0.924; 1.084]
Omitting Vinci, 2011 1.000 [0.925; 1.081]
Omitting Vinci, 2013 1.000 [0.925; 1.082]
Omitting Wang, 2014 1.006 [0.928; 1.091]
Omitting Wang, 2014 0.999 [0.923; 1.082]
Omitting Wei, 2014 1.011 [0.933; 1.095]
Omitting Yan, 2015 1.008 [0.930; 1.092]
Omitting Yang, 2016 1.006 [0.929; 1.089]
Omitting Zhang, 2012 0.997 [0.922; 1.079]
Omitting Filled: Wang, 2014 1.010 [0.933; 1.093]
Omitting Filled: Liu, 2015 1.009 [0.933; 1.092]
Omitting Filled: Zhang, 2012 1.012 [0.936; 1.095]
Omitting Filled: Kim, 2012 1.012 [0.935; 1.094]
Omitting Filled: Ranjbar, 2018 1.008 [0.932; 1.091]
Omitting Filled: Vinci, 2013 1.009 [0.933; 1.091]
Omitting Filled: Vinci, 2011 1.009 [0.934; 1.091]
Omitting Filled: Tandon, 2017 1.011 [0.937; 1.091]
Omitting Filled: Sushma, 2015 1.006 [0.932; 1.087]
Random effects model 1.005 [0.929; 1.087]
1

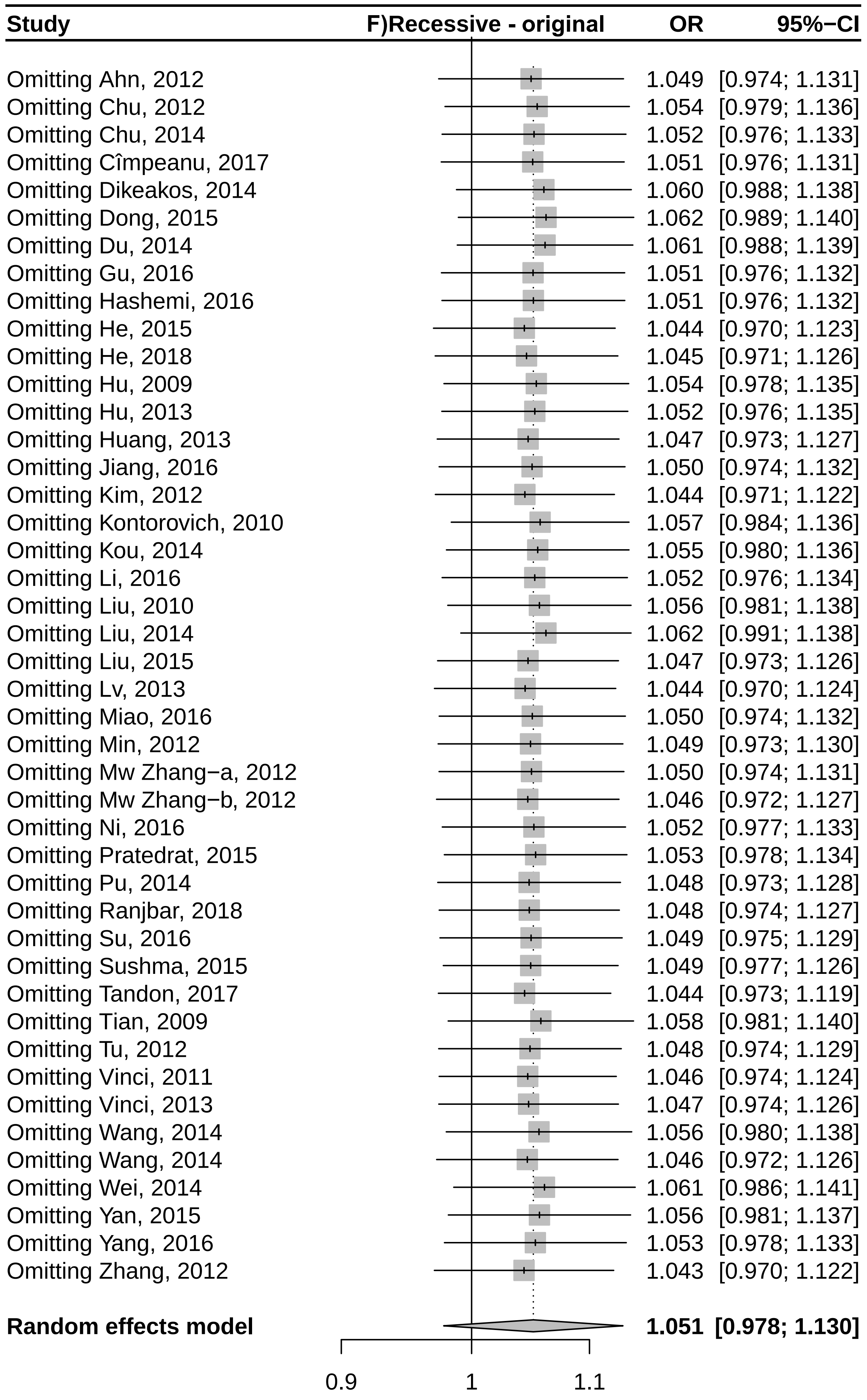
Study
F)Recessive - original
OR
95%−CI
Omitting Ahn, 2012 1.049 [0.974; 1.131]
Omitting Chu, 2012 1.054 [0.979; 1.136]
Omitting Chu, 2014 1.052 [0.976; 1.133]
Omitting Cîmpeanu, 2017 1.051 [0.976; 1.131]
Omitting Dikeakos, 2014 1.060 [0.988; 1.138]
Omitting Dong, 2015 1.062 [0.989; 1.140]
Omitting Du, 2014 1.061 [0.988; 1.139]
Omitting Gu, 2016 1.051 [0.976; 1.132]
Omitting Hashemi, 2016 1.051 [0.976; 1.132]
Omitting He, 2015 1.044 [0.970; 1.123]
Omitting He, 2018 1.045 [0.971; 1.126]
Omitting Hu, 2009 1.054 [0.978; 1.135]
Omitting Hu, 2013 1.052 [0.976; 1.135]
Omitting Huang, 2013 1.047 [0.973; 1.127]
Omitting Jiang, 2016 1.050 [0.974; 1.132]
Omitting Kim, 2012 1.044 [0.971; 1.122]
Omitting Kontorovich, 2010 1.057 [0.984; 1.136]
Omitting Kou, 2014 1.055 [0.980; 1.136]
Omitting Li, 2016 1.052 [0.976; 1.134]
Omitting Liu, 2010 1.056 [0.981; 1.138]
Omitting Liu, 2014 1.062 [0.991; 1.138]
Omitting Liu, 2015 1.047 [0.973; 1.126]
Omitting Lv, 2013 1.044 [0.970; 1.124]
Omitting Miao, 2016 1.050 [0.974; 1.132]
Omitting Min, 2012 1.049 [0.973; 1.130]
Omitting Mw Zhang−a, 2012 1.050 [0.974; 1.131]
Omitting Mw Zhang−b, 2012 1.046 [0.972; 1.127]
Omitting Ni, 2016 1.052 [0.977; 1.133]
Omitting Pratedrat, 2015 1.053 [0.978; 1.134]
Omitting Pu, 2014 1.048 [0.973; 1.128]
Omitting Ranjbar, 2018 1.048 [0.974; 1.127]
Omitting Su, 2016 1.049 [0.975; 1.129]
Omitting Sushma, 2015 1.049 [0.977; 1.126]
Omitting Tandon, 2017 1.044 [0.973; 1.119]
Omitting Tian, 2009 1.058 [0.981; 1.140]
Omitting Tu, 2012 1.048 [0.974; 1.129]
Omitting Vinci, 2011 1.046 [0.974; 1.124]
Omitting Vinci, 2013 1.047 [0.974; 1.126]
Omitting Wang, 2014 1.056 [0.980; 1.138]
Omitting Wang, 2014 1.046 [0.972; 1.126]
Omitting Wei, 2014 1.061 [0.986; 1.141]
Omitting Yan, 2015 1.056 [0.981; 1.137]
Omitting Yang, 2016 1.053 [0.978; 1.133]
Omitting Zhang, 2012 1.043 [0.970; 1.122]
Random effects model 1.051 [0.978; 1.130]
0.9
1
1.1

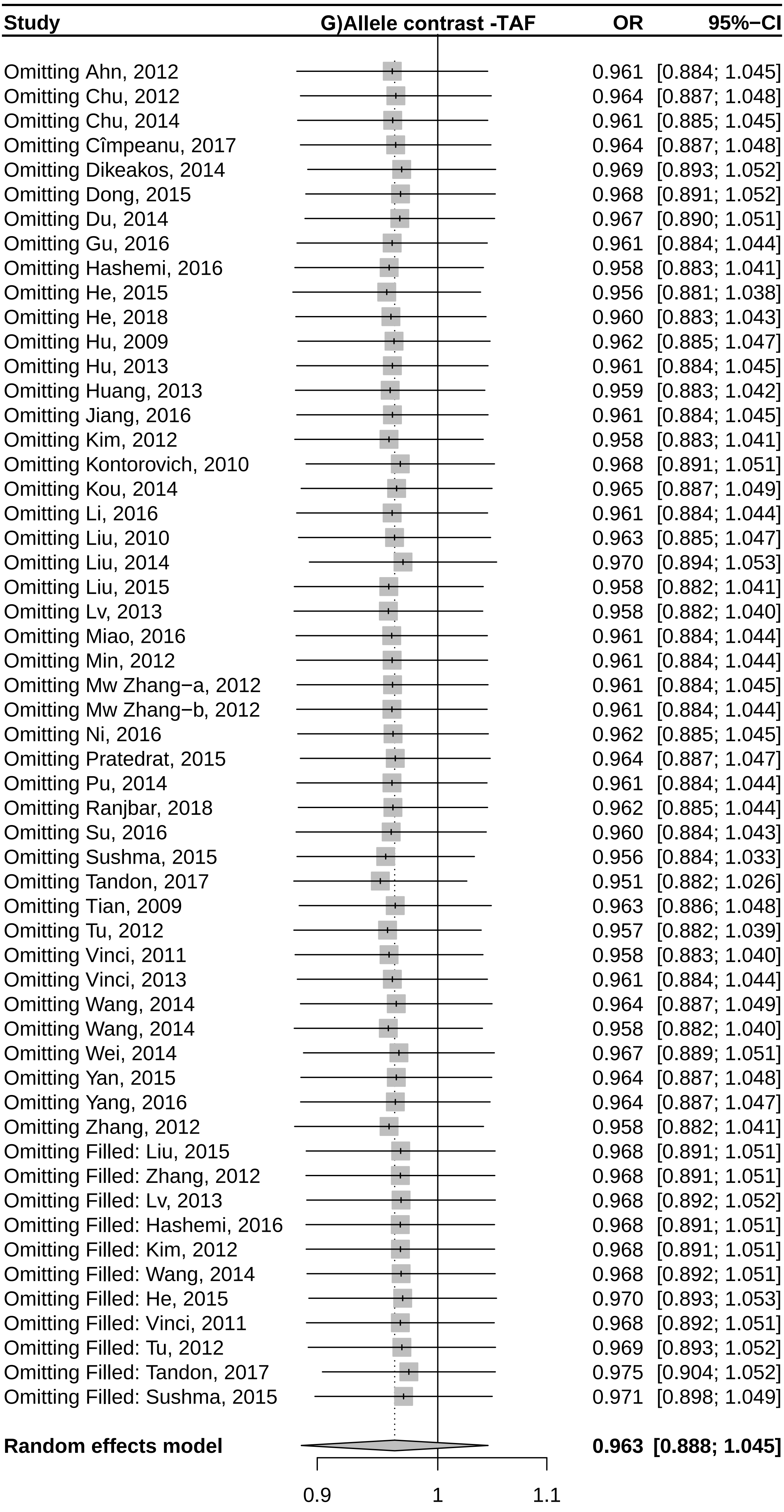
Study
G)Allele contrast -TAF
OR
95%−CI
Omitting Ahn, 2012 0.961 [0.884; 1.045]
Omitting Chu, 2012 0.964 [0.887; 1.048]
Omitting Chu, 2014 0.961 [0.885; 1.045]
Omitting Cîmpeanu, 2017 0.964 [0.887; 1.048]
Omitting Dikeakos, 2014 0.969 [0.893; 1.052]
Omitting Dong, 2015 0.968 [0.891; 1.052]
Omitting Du, 2014 0.967 [0.890; 1.051]
Omitting Gu, 2016 0.961 [0.884; 1.044]
Omitting Hashemi, 2016 0.958 [0.883; 1.041]
Omitting He, 2015 0.956 [0.881; 1.038]
Omitting He, 2018 0.960 [0.883; 1.043]
Omitting Hu, 2009 0.962 [0.885; 1.047]
Omitting Hu, 2013 0.961 [0.884; 1.045]
Omitting Huang, 2013 0.959 [0.883; 1.042]
Omitting Jiang, 2016 0.961 [0.884; 1.045]
Omitting Kim, 2012 0.958 [0.883; 1.041]
Omitting Kontorovich, 2010 0.968 [0.891; 1.051]
Omitting Kou, 2014 0.965 [0.887; 1.049]
Omitting Li, 2016 0.961 [0.884; 1.044]
Omitting Liu, 2010 0.963 [0.885; 1.047]
Omitting Liu, 2014 0.970 [0.894; 1.053]
Omitting Liu, 2015 0.958 [0.882; 1.041]
Omitting Lv, 2013 0.958 [0.882; 1.040]
Omitting Miao, 2016 0.961 [0.884; 1.044]
Omitting Min, 2012 0.961 [0.884; 1.044]
Omitting Mw Zhang−a, 2012 0.961 [0.884; 1.045]
Omitting Mw Zhang−b, 2012 0.961 [0.884; 1.044]
Omitting Ni, 2016 0.962 [0.885; 1.045]
Omitting Pratedrat, 2015 0.964 [0.887; 1.047]
Omitting Pu, 2014 0.961 [0.884; 1.044]
Omitting Ranjbar, 2018 0.962 [0.885; 1.044]
Omitting Su, 2016 0.960 [0.884; 1.043]
Omitting Sushma, 2015 0.956 [0.884; 1.033]
Omitting Tandon, 2017 0.951 [0.882; 1.026]
Omitting Tian, 2009 0.963 [0.886; 1.048]
Omitting Tu, 2012 0.957 [0.882; 1.039]
Omitting Vinci, 2011 0.958 [0.883; 1.040]
Omitting Vinci, 2013 0.961 [0.884; 1.044]
Omitting Wang, 2014 0.964 [0.887; 1.049]
Omitting Wang, 2014 0.958 [0.882; 1.040]
Omitting Wei, 2014 0.967 [0.889; 1.051]
Omitting Yan, 2015 0.964 [0.887; 1.048]
Omitting Yang, 2016 0.964 [0.887; 1.047]
Omitting Zhang, 2012 0.958 [0.882; 1.041]
Omitting Filled: Liu, 2015 0.968 [0.891; 1.051]
Omitting Filled: Zhang, 2012 0.968 [0.891; 1.051]
Omitting Filled: Lv, 2013 0.968 [0.892; 1.052]
Omitting Filled: Hashemi, 2016 0.968 [0.891; 1.051]
Omitting Filled: Kim, 2012 0.968 [0.891; 1.051]
Omitting Filled: Wang, 2014 0.968 [0.892; 1.051]
Omitting Filled: He, 2015 0.970 [0.893; 1.053]
Omitting Filled: Vinci, 2011 0.968 [0.892; 1.051]
Omitting Filled: Tu, 2012 0.969 [0.893; 1.052]
Omitting Filled: Tandon, 2017 0.975 [0.904; 1.052]
Omitting Filled: Sushma, 2015 0.971 [0.898; 1.049]
Random effects model 0.963 [0.888; 1.045]
0.9
1
1.1

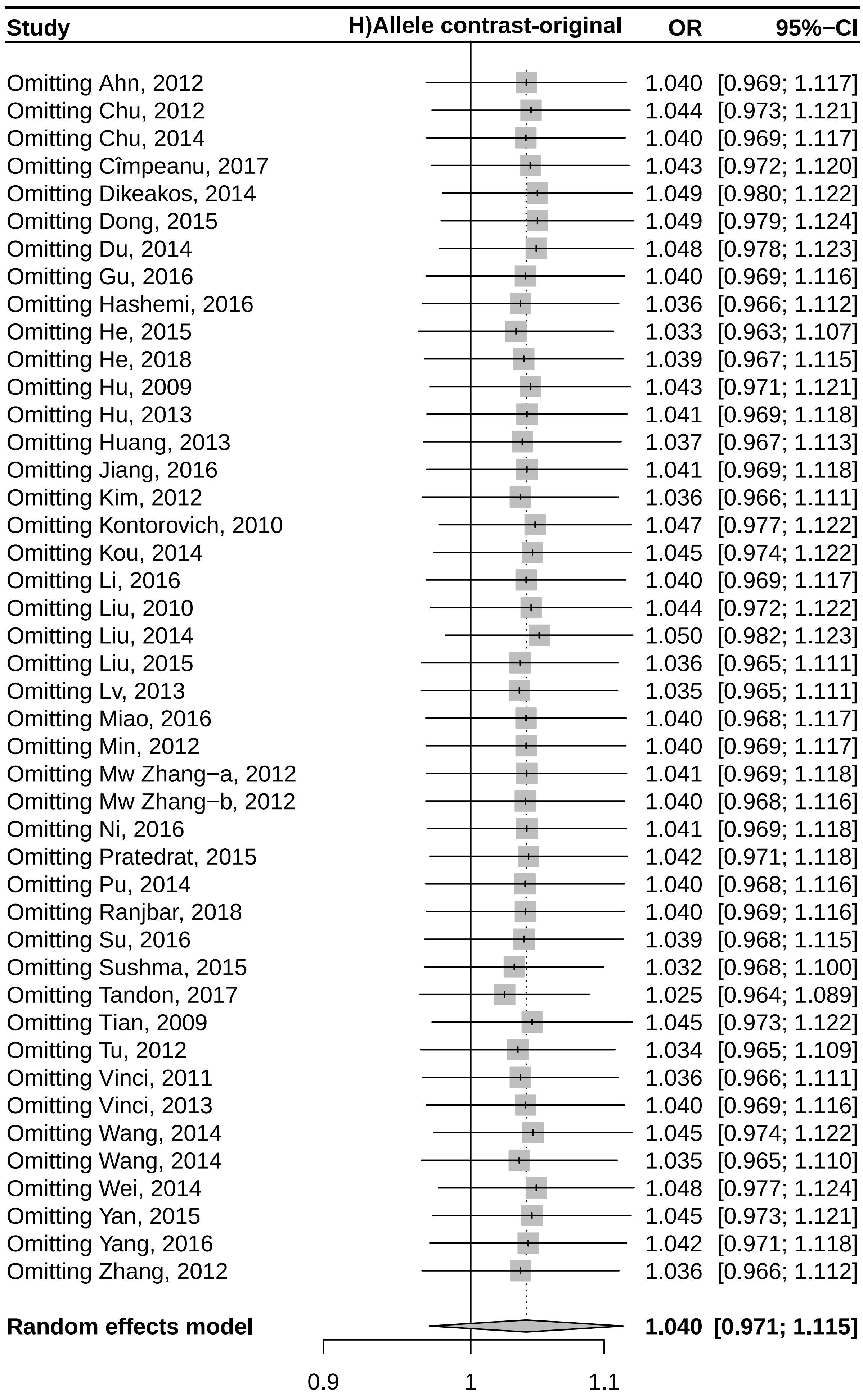
Study
H)Allele contrast-original
OR
95%−CI
Omitting Ahn, 2012 1.040 [0.969; 1.117]
Omitting Chu, 2012 1.044 [0.973; 1.121]
Omitting Chu, 2014 1.040 [0.969; 1.117]
Omitting Cîmpeanu, 2017 1.043 [0.972; 1.120]
Omitting Dikeakos, 2014 1.049 [0.980; 1.122]
Omitting Dong, 2015 1.049 [0.979; 1.124]
Omitting Du, 2014 1.048 [0.978; 1.123]
Omitting Gu, 2016 1.040 [0.969; 1.116]
Omitting Hashemi, 2016 1.036 [0.966; 1.112]
Omitting He, 2015 1.033 [0.963; 1.107]
Omitting He, 2018 1.039 [0.967; 1.115]
Omitting Hu, 2009 1.043 [0.971; 1.121]
Omitting Hu, 2013 1.041 [0.969; 1.118]
Omitting Huang, 2013 1.037 [0.967; 1.113]
Omitting Jiang, 2016 1.041 [0.969; 1.118]
Omitting Kim, 2012 1.036 [0.966; 1.111]
Omitting Kontorovich, 2010 1.047 [0.977; 1.122]
Omitting Kou, 2014 1.045 [0.974; 1.122]
Omitting Li, 2016 1.040 [0.969; 1.117]
Omitting Liu, 2010 1.044 [0.972; 1.122]
Omitting Liu, 2014 1.050 [0.982; 1.123]
Omitting Liu, 2015 1.036 [0.965; 1.111]
Omitting Lv, 2013 1.035 [0.965; 1.111]
Omitting Miao, 2016 1.040 [0.968; 1.117]
Omitting Min, 2012 1.040 [0.969; 1.117]
Omitting Mw Zhang−a, 2012 1.041 [0.969; 1.118]
Omitting Mw Zhang−b, 2012 1.040 [0.968; 1.116]
Omitting Ni, 2016 1.041 [0.969; 1.118]
Omitting Pratedrat, 2015 1.042 [0.971; 1.118]
Omitting Pu, 2014 1.040 [0.968; 1.116]
Omitting Ranjbar, 2018 1.040 [0.969; 1.116]
Omitting Su, 2016 1.039 [0.968; 1.115]
Omitting Sushma, 2015 1.032 [0.968; 1.100]
Omitting Tandon, 2017 1.025 [0.964; 1.089]
Omitting Tian, 2009 1.045 [0.973; 1.122]
Omitting Tu, 2012 1.034 [0.965; 1.109]
Omitting Vinci, 2011 1.036 [0.966; 1.111]
Omitting Vinci, 2013 1.040 [0.969; 1.116]
Omitting Wang, 2014 1.045 [0.974; 1.122]
Omitting Wang, 2014 1.035 [0.965; 1.110]
Omitting Wei, 2014 1.048 [0.977; 1.124]
Omitting Yan, 2015 1.045 [0.973; 1.121]
Omitting Yang, 2016 1.042 [0.971; 1.118]
Omitting Zhang, 2012 1.036 [0.966; 1.112]
Random effects model 1.040 [0.971; 1.115]
0.9
1
1.1
