# Supplementary figures and images for: Association of mir-196a-2 rs11614913 and mir-149 rs2292832 Polymorphisms With Risk of Cancer: An Updated Meta-Analysis
Source: Front Genet. 2019 Mar 15;10:186. doi: 10.3389/fgene.2019.00186 (PMC6429108; doi:10.3389/fgene.2019.00186)

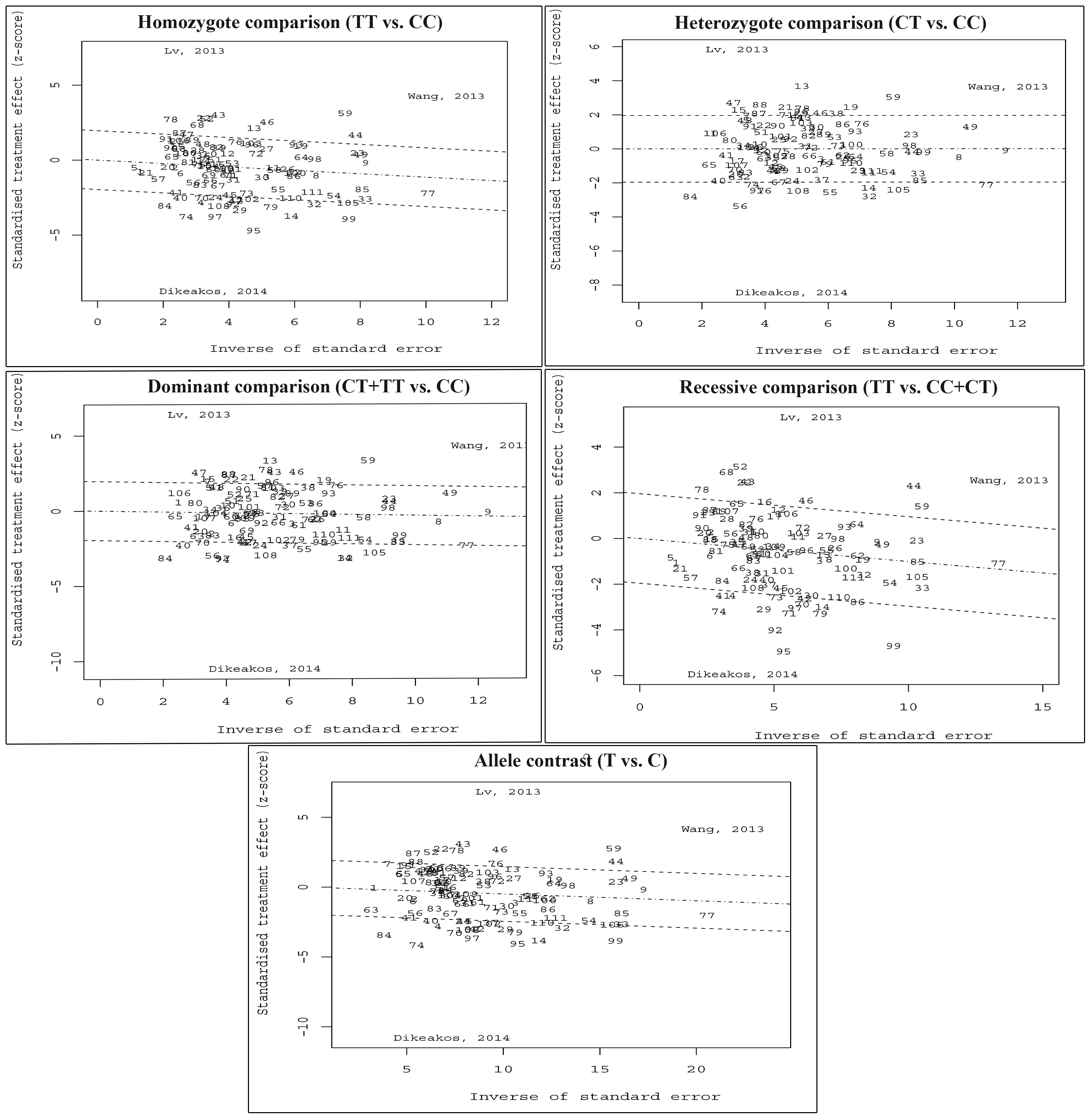

Supplement: Supplementary file 1 [file Data_Sheet_1.ZIP › Supplementary Figures/Suppl. Fig. S7.tif]
