# Supplementary material for: Association of mir-196a-2 rs11614913 and mir-149 rs2292832 Polymorphisms With Risk of Cancer: An Updated Meta-Analysis
Source: Front Genet. 2019 Mar 15;10:186. doi: 10.3389/fgene.2019.00186 (PMC6429108; doi:10.3389/fgene.2019.00186)
Supplement: Supplementary file 2 [file Data_Sheet_2.ZIP › Supp. Table S2.docx]

**Supplementary Table S2.** Meta-analysis of miR-196a rs11614913 and cancer risk in four subgroups that were influenced by departure from HWE in sensitivity analysis. For the breast, hepatocellular, colorectal and lung cancer subgroups, the results of meta-analysis were influenced by excluding HWE violating studies. Therefore, adjusted ORs of individual HWE-deviated studies was used in the meta-analysis of these subgroups. For each subgroup, the table shows meta-analysis of all studies in the subgroup (including those with controls violating HWE), alongside with the results of HWD-sensitivity analysis and HWD-adjusted analysis.

|  |  | **Homozygote (TT vs. CC)** | | | **Heterozygote (CT vs. CC)** | | | **Dominant (TT+CT vs. CC)** | | | **Recessive (TT vs. CT+CC)** | | |
| --- | --- | --- | --- | --- | --- | --- | --- | --- | --- | --- | --- | --- | --- |
| **Subgroup** | **Cases/Controls^a^** | **OR^b^ (95% CI** | ***P*^c^** | **I*^2^*** | **OR^b^ (95% CI** | ***P*^c^** | **I*^2^*** | **OR^b^ (95% CI** | ***P*^c^** | **I*^2^*** | **OR^b^ (95% CI** | ***P*^c^** | **I*^2^*** |
| **BC** |  |  |  |  |  |  |  |  |  |  |  |  |  |
| All**^d^** | 7401/8828 | 0.85 (0.71-1.01) | 0.001 | 56.4 | 0.93(0.81-1.08) | 0.002 | 53.3 | 0.91(0.78-1.06) | 2e-4 | 60.8 | 0.89(0.79-1.01) | 0.024 | 43 |
| HWE**^e^** | 6625/8003 | **0.79[0.66-0.96]** | 0.013 | 50.4 | 0.92[0.79-1.07] | 0.011 | 49.3 | 0.89[0.76-1.04] | 0.002 | 57 | **0.86[0.74-0.99]** | 0.040 | 42.7 |
| Adjusted **^f^** | 7401/8828 | **0.75[0.61-0.93]** | 5e-4 | 59.6 | 0.89[0.78-1.02] | 0.017 | 43.8 | 0.86[0.74-1.00] | 8e-4 | 56.7 | **0.84[0.71-0.98]** | 0.002 | 54.3 |
| **HCC** |  |  |  |  |  |  |  |  |  |  |  |  |  |
| All**^d^** | 5401/6326 | **0.73(0.57-0.94)** | <1e-4 | 69.5 | 0.92(0.78-1.09) | 0.002 | 55.2 | 0.87(0.73-1.04) | <1e-4 | 64.7 | **0.79(0.66-0.95)** | 2e-4 | 63.2 |
| HWE**^e^** | 4355/4903 | 0.86[0.66-1.11] | 0.001 | 61.8 | 1.037[0.88-1.22] | 0.047 | 42.4 | 0.99[0.83-1.17] | 0.011 | 52.3 | 0.86[0.68-1.07] | 6e-4 | 64.1 |
| Adjusted **^f^** | 5401/6326 | **0.69[0.53-0.91]** | <1e-4 | 76.8 | 0.96[0.83-1.10] | 0.036 | 41 | 0.88[0.74-1.04] | 2e-4 | 62.4 | **0.72[0.57-0.90]** | <1e-4 | 77 |
| **CRC** |  |  |  |  |  |  |  |  |  |  |  |  |  |
| All**^d^** | 2567/4211 | 1.21(0.65-2.27) | <1e-4 | 87.6 | 1.12(0.71- 1.75) | <1e-4 | 81.1 | 1.13(0.70-1.85) | <1e-4 | 84.7 | 1.08(0.83- 1.42) | <1e-4 | 78.6 |
| HWE**^e^** | 2220/3680 | **0.82[0.70-0.97]** | 0.089 | 41.7 | 0.92[0.81-1.05] | 0.183 | 29.4 | 0.90[0.80-1.02] | 0.070 | 44.7 | 0.90[0.79-1.03] | 0.137 | 35.1 |
| Adjusted **^f^** | 2567/4211 | 1.17[0.67-2.06] | <1e-4 | 85.5 | 1.16[0.68-1.96] | <1e-4 | 85.3 | 1.16[0.68- 1.95] | <1e-4 | 86.4 | 0.99[0.82-1.20] | 0.036 | 49.7 |
| **LC** |  |  |  |  |  |  |  |  |  |  |  |  |  |
| All**^d^** | 4453/4932 | 0.86[0.63-1.17] | 0.009 | 60.2 | 1.01[0.91-1.12] | 0.057 | 46.9 | 1.00[0.82-1.21] | 0.015 | 57.7 | 0.82[0.65-1.03] | 0.012 | 59 |
| HWE**^e^** | 4203/4677 | **0.76[0.67-0.86]** | 0.238 | 24 | **0.97[0.87-1.08]** | 0.240 | 23.7 | **0.90[0.81-0.99]** | 0.262 | 21 | **0.78[0.71-0.86]** | 0.095 | 42.4 |
| Adjusted **^f^** | 4453/4932 | 0.66[0.33-1.29] | <1e-4 | 87.7 | 0.95[0.85-1.05] | 0.075 | 43.9 | 0.87[0.64-1.18] | 4e-4 | 72.1 | 0.66[0.38-1.15] | <1e-4 | 88.9 |

**a:** Number of cases and controls in each subgroup; **b:** Pooled ORs and 95% CIs; **c:** *P*-value for test of heterogeneity; **d:** This represents meta-analysis of all studies in the corresponding subgroup, including HWE-deviated studies; **e:** This shows meta-analysis of studies in each subgroup after excluding HWE-deviated studies. **f:** This shows results of meta-analysis of all studies in the subgroup while adjusting for departures from HWE as described in materials and methods (HWD-adjusted ORs). **Abbreviations:** BC: breast cancer; HCC: hepatocellular cancer; CRC: colorectal cancer; LC: lung cancer.
