# Supplementary material for: Association of mir-196a-2 rs11614913 and mir-149 rs2292832 Polymorphisms With Risk of Cancer: An Updated Meta-Analysis
Source: Front Genet. 2019 Mar 15;10:186. doi: 10.3389/fgene.2019.00186 (PMC6429108; doi:10.3389/fgene.2019.00186)
Supplement: Supplementary file 2 [file Data_Sheet_2.ZIP › Supp. Table S3.docx]

**Supplementary Table S3. HWD sensitivity analyses for miR-149 polymorphism.** The table shows the results of meta-analysis of studies in which genotype distributions in controls were in agreement with HWE.

|  |  | **Homozygote**  **(TT vs. CC)** | | | **Heterozygote**  **(CT vs. CC)** | | | **Dominant (TT+CT vs. CC)** | | | **Recessive**  **(TT vs. CT+CC)** | | | **Allelic**  **(T vs. C)** | | |
| --- | --- | --- | --- | --- | --- | --- | --- | --- | --- | --- | --- | --- | --- | --- | --- | --- |
| **Groups** | **Cases/Controls^a^** | **OR^b^ (95% CI** | ***P*^c^** | **I*^2^*** | **OR^b^ (95% CI** | ***P*^c^** | **I*^2^*** | **OR^b^ (95% CI** | ***P*^c^** | **I*^2^*** | **OR^b^ (95% CI** | ***P*^c^** | **I*^2^*** | **OR^b^ (95% CI** | ***P*^c^** | **I*^2^*** |
| **All ^d^** | 12873/15569 | 1.00[0.87-1.16] | <1e-4 | 57.3 | 0.95[0.89-1.02] | 0.054 | 29.7 | 0.98[0.87-1.09] | 4e-4 | 50.8 | 1.03[0.95-1.12] | 0.002 | 45.2 | 1.02[0.94-1.09] | <1e-4 | 61.9 |
| **Quality** |  |  |  |  |  |  |  |  |  |  |  |  |  |  |  |  |
| High (>8) | 12341/15191 | 0.98[0.85-1.13] | <1e-4 | 57.2 | 0.94[0.88-1.01] | 0.188 | 18.3 | 0.96[0.86-1.07] | 0.001 | 48.5 | 1.02[0.93-1.12] | 0.001 | 49.7 | 1.01[0.93-1.09] | <1e-4 | 64.3 |
| Low (≤8) | 532/378 | 1.38[0.93-2.05] | 0.122 | 48.2 | 1.11[0.29-4.29] | 0.029 | 66.7 | 1.23[0.42- 3.58] | 0.060 | 59.5 | 1.18[0.89-1.56] | 0.7204 | 0 | 1.17[0.97-1.42] | 0.531 | 0 |
| **Genotyping** |  |  |  |  |  |  |  |  |  |  |  |  |  |  |  |  |
| PCR-RFLP | 7876/9055 | 1.01[0.85-1.21] | 0.006 | 47.8 | 0.97[0.90-1.06] | 0.155 | 23.2 | 1.00[0.87-1.15] | 0.011 | 44.6 | 1.06[0.95-1.18] | 0.029 | 39 | 1.04[0.95-1.14] | 2e-4 | 58.7 |
| Others | 4997/6514 | 0.98[0.73-1.31] | 1e-4 | 71.1 | 0.90[0.80-1.02] | 0.071 | 41.7 | 0.93[0.75-1.16] | 0.003 | 61.5 | 0.99[0.84-1.16] | 0.011 | 56 | 0.98[0.86-1.11] | 5e-4 | 68.4 |
| **Ethnicities** |  |  |  |  |  |  |  |  |  |  |  |  |  |  |  |  |
| Asian | 11198/13364 | 1.01[0.87-1.17] | <1e-4 | 57.4 | 0.97[0.87-1.08] | 0.043 | 33.4 | 0.97[0.87-1.08] | 0.043 | 33.4 | 1.03[0.95-1.13] | 0.006 | 44.2 | 1.03[0.95-1.11] | <1e-4 | 63.3 |
| Caucasians | 1675/2205 | 0.98[0.48-1.96] | 0.022 | 64.9 | 0.93[0.80-1.07] | 0.30 | 16.5 | 0.94[0.82-1.08] | 0.13 | 43.5 | 1.04[0.62-1.72] | 0.052 | 57.4 | 0.95[0.72-1.27] | 0.049 | 57.9 |
| **Cancer categories** |  |  |  |  |  |  |  |  |  |  |  |  |  |  |  |  |
| GI | 4813/5845 | 0.95[0.78-1.16] | 0.013 | 48.3 | **0.88[0.79-0.98]** | 0.349 | 9 | 0.90[0.82-1.00] | 0.090 | 33.2 | 1.06[0.93-1.21] | 0.034 | 42.2 | 1.00[0.91-1.10] | 0.004 | 53.5 |
| HNC | 2215/3144 | 1.33[0.65-2.71] | 0.021 | 65.4 | 1.27[0.74-2.15] | 0.029 | 62.7 | 1.39[0.76-2.55] | 0.004 | 73.8 | 1.10[0.95-1.26] | 0.218 | 30.5 | 1.21[0.79-1.84] | 0.003 | 74.5 |
| Others | 5845/6580 | 0.98[0.76-1.27] | 9e-4 | 65 | 0.96[0.87-1.07] | 0.258 | 18.9 | 0.97[0.81-1.17] | 0.017 | 52.4 | 0.98[0.84-1.13] | 0.015 | 53.1 | 0.99[0.88-1.11] | 6e-4 | 66.2 |
| **Cancer types** |  |  |  |  |  |  |  |  |  |  |  |  |  |  |  |  |
| BC | 568/1772 | 1.32[0.55-3.17] | 0.054 | 65.6 | 1.04[0.88-1.22] | 0.128 | 51.2 | 1.21[0.55-2.66] | 0.046 | 67.5 | **1.20[1.01-1.42]** | 0.235 | 30.9 | 1.15[0.77-1.72] | 0.035 | 70 |
| HCC | 2220/2675 | 0.95[0.60-1.49] | 0.003 | 69.7 | 0.90[0.77-1.05] | 0.156 | 35.6 | 0.92[0.66-1.29] | 0.016 | 61.5 | 1.00[0.76-1.10] | 0.017 | 61 | 0.99[0.78-1.24] | 9e-4 | 73.6 |
| GC | 1471/1985 | 0.95[0.76-1.19] | 0.150 | 38.3 | 0.88[0.73-1.06] | 0.345 | 11 | 0.92[0.77-1.10] | 0.200 | 31.3 | 1.05[0.90-1.23] | 0.182 | 33.8 | 0.99[0.89-1.10] | 0.096 | 46.5 |
| CRC | 1122/1185 | 1.04[0.79-1.37] | 0.638 | 0 | 0.82[0.63-1.06] | 0.512 | 0 | 0.91[0.72-1.16] | 0.799 | 0 | 1.18[0.99-1.40**]** | 0.670 | 0 | 1.06[0.94-1.20] | 0.990 | 0 |
| LC | 1714/1559 | 1.04[0.82-1.31] | 0.116 | 53.5 | 1.03[0.83-1.29] | 0.602 | 0 | 1.04[0.84-1.29] | 0.351 | 4.3 | 0.98[0.85-1.13] | 0.175 | 42.4 | 1.04[0.70-1.52] | 0.157 | 45.8 |
| Others | 4642/6393 | 0.96 [0.69-1.34] | 8e-4 | 67 | 1.01[0.80-1.27] | 0.026 | 50.7 | 1.03[0.77-1.37] | 5e-4 | 68.1 | 0.95[0.81-1.11] | 0.040 | 47.2 | 1.00[0.83-1.20] | <1e-4 | 72.9 |

**a:** n represents number of cases and controls in each group; **b:** Pooled ORs and 95% confidence intervals; **c:** P-value of the heterogeneity test; **d:** meta-analysis of all studies excluding those with the control group not in HWE;

**GI**: gastrointestinal tract cancers; **HNC**: Head and neck cancers; **BC**: Breast cancer; **HCC**: Hepatocellular cancer; **GC**: Gastric cancer; **CRC**: Colorectal cancer; **LC**: Lung cancer;
