# Supplementary material for: Association of mir-196a-2 rs11614913 and mir-149 rs2292832 Polymorphisms With Risk of Cancer: An Updated Meta-Analysis
Source: Front Genet. 2019 Mar 15;10:186. doi: 10.3389/fgene.2019.00186 (PMC6429108; doi:10.3389/fgene.2019.00186)
Supplement: Supplementary file 2 [file Data_Sheet_2.ZIP › Supp. Table S4.docx]

**Supplementary Table S4.** Meta-analysis of miR-149 rs2292832 and cancer risk in three subgroups that were influenced by departure from HWE in sensitivity analysis. For the non-RFLP, breast and colorectal cancer subgroups, the results of meta-analysis were influenced by excluding HWE violating studies. Therefore, meta-analysis with adjusted ORs were performed for these subgroups. For each subgroup, the table shows meta-analysis of all studies in the subgroup (including HWD studies) alongside with the results of HWD-sensitivity analysis and HWD-adjusted meta-analysis.

|  |  | **Homozygote**  **(TT vs. CC)** | | | **Heterozygote**  **(CT vs. CC)** | | | **Dominant (TT+CT vs. CC)** | | | **Recessive**  **(TT vs. CT+CC)** | | |
| --- | --- | --- | --- | --- | --- | --- | --- | --- | --- | --- | --- | --- | --- |
|  | **Cases/Controls^a^** | **OR^b^ (95% CI** | ***P*^c^** | **I*^2^*** | **OR^b^ (95% CI** | ***P*^c^** | **I*^2^*** | **OR^b^ (95% CI** | ***P*^c^** | **I*^2^*** | **OR^b^ (95% CI** | ***P*^c^** | **I*^2^*** |
| **Non-RFLP** |  |  |  |  |  |  |  |  |  |  |  |  |  |
| All **^d^** | 6517/8477 | 0.94[0.76-1.16] | 9e-4 | 61.6 | **0.88[0.79-0.98]** | 0.178 | 25 | 0.911[0.77-1.06] | 0.018 | 48.3 | 1.0[0.88-1.13] | 0.011 | 51.4 |
| HWE **^e^** | 4997/6514 | 0.98[0.73-1.31] | 1e-4 | 71.1 | 0.90[0.80-1.02] | 0.071 | 41.7 | 0.93[0.75-1.16] | 0.003 | 61.5 | 0.99[0.84-1.16] | 0.011 | 56 |
| Adjusted **^f^** | 6517/8477 | 0.81[0.59-1.10] | <1e-4 | 77 | **0.68 [0.48-0.98]** | <1e-4 | 83.3 | 0.75[0.55-1.01] | <1e-4 | 80.2 | 1.04[0.90-1.21] | 3e-4 | 65 |
| **Breast** |  |  |  |  |  |  |  |  |  |  |  |  |  |
| All **^d^** | 1871/1894 | 1.12[0.53-2.36] | 0.014 | 71.6 | 1.02[0.87-1.19] | 0.192 | 36.7 | 1.07[0.59-1.93] | 0.031 | 65.9 | 1.10[0.68-1.77] | 0.063 | 58.8 |
| HWE **^e^** | 1704/1772 | 1.32[0.55-3.17] | 0.054 | 65.6 | 1.04[0.88-1.22] | 0.128 | 51.2 | 1.21[0.55-2.66] | 0.046 | 67.5 | **1.20[1.01-1.42]** | 0.235 | 30.9 |
| Adjusted **^f^** | 1871/1894 | 0.94[0.26-3.39] | <1e-4 | 87.1 | 0.74[0.16-3.27] | <1e-4 | 91.1 | 0.82[0.20-3.36] | <1e-4 | 91.6 | 1.11[0.71-1.74] | 0.083 | 54.9 |
| **Colorectal** |  |  |  |  |  |  |  |  |  |  |  |  |  |
| All **^d^** | 1469/1644 | 1.10[0.86-1.40] | 0.671 | 0 | 0.85[0.67-1.07] | 0.615 | 0 | 0.97[0.78-1.20] | 0.704 | 0 | **1.21[1.04-1.40]** | 0.748 | 0 |
| HWE **^e^** | 1122/1185 | 1.04[0.79-1.37] | 0.638 | 0 | 0.82[0.63-1.06] | 0.512 | 0 | 0.91[0.72-1.16] | 0.799 | 0 | 1.18[0.99-1.40**]** | 0.670 | 0 |
| Adjusted **^f^** | 1469/1644 | 0.95[0.74-1.22] | 0.429 | 0 | 0.64[0.33-1.23] | 0.024 | 64.2 | 0.84[0.67-1.05] | 0.375 | 5.5 | **1.31[1.01-1.69]** | 0.222 | 29.9 |

**a:** represent the number of cases and controls in each contrast; **b:** Pooled ORs and 95% CIs; **c:** *P*-value for test of heterogeneity; **d:** This represents meta-analysis of all studies in the corresponding subgroup, including HWE-deviated studies; **e:** This shows meta-analysis of studies in each subgroup after excluding HWE-deviated studies. **f:** This shows results of meta-analysis of all studies in the subgroup while adjusting for departures from HWE as described in materials and methods (HWD-adjusted ORs).
