# Supplementary material for: Association of mir-196a-2 rs11614913 and mir-149 rs2292832 Polymorphisms With Risk of Cancer: An Updated Meta-Analysis
Source: Front Genet. 2019 Mar 15;10:186. doi: 10.3389/fgene.2019.00186 (PMC6429108; doi:10.3389/fgene.2019.00186)
Supplement: Supplementary file 2 [file Data_Sheet_2.ZIP › Supp. Table S1.docx]

**Supplementary Table S1.** HWD sensitivity analyses for miR-196a2 polymorphism. The table shows the results of meta-analysis of studies in which genotype distributions in controls were in agreement with HWE.

|  |  | **Homozygote**  **(TT vs. CC)** | | | **Heterozygote**  **(CT vs. CC)** | | | **Dominant (TT+CT vs. CC)** | | | **Recessive**  **(TT vs. CT+CC)** | | | **Allelic**  **(T vs. C)** | | |
| --- | --- | --- | --- | --- | --- | --- | --- | --- | --- | --- | --- | --- | --- | --- | --- | --- |
| **Groups** | **Cases/Controls^a^** | **OR^b^ (95% CI** | ***P*^c^** | **I*^2^*** | **OR^b^ (95% CI** | ***P*^c^** | **I*^2^*** | **OR^b^ (95% CI** | ***P*^c^** | **I*^2^*** | **OR^b^ (95% CI** | ***P*^c^** | **I*^2^*** | **OR^b^ (95% CI** | ***P*^c^** | **I*^2^*** |
| **All ^d^** | 36620/42501 | **0.88[0.80-0.98]** | <1e-4 | 74.6 | 1.00[0.93-1.08] | <1e-4 | 66.3 | 0.97[0.89-1.04] | <1e-4 | 74.3 | **0.88[0.82-0.95]** | <1e-4 | 67.7 | **0.94[0.89-0.99]** | <1e-4 | 77.3 |
| **Quality** |  |  |  |  |  |  |  |  |  |  |  |  |  |  |  |  |
| High (>8) | 34305/40306 | **0.85[0.76-0.95]** | <1e-4 | 76 | 0.98[0.91-1.05] | <1e-4 | 65.5 | 0.94[0.86-1.01] | <1e-4 | 74.5 | **0.86[0.80-0.94]** | <1e-4 | 69.5 | **0.92[0.87-0.98]** | <1e-4 | 78.4 |
| Low(≤8) | 2315/2195 | 1.18[0.88-1.59] | 0.038 | 46.4 | 1.24[0.92-1.68] | 3e-4 | 66.8 | 1.24[0.94-1.64] | 4e-4 | 66.2 | 1.06[0.91-1.24] | 0.570 | 0 | 1.12[0.97-1.30] | 0.016 | 51.3 |
| **Genotyping** |  |  |  |  |  |  |  |  |  |  |  |  |  |  |  |  |
| PCR-RFLP | 12850/14481 | 0.86[0.71-1.04] | <1e-4 | 77 | 0.96[0.84-1.10] | <1e-4 | 72.8 | 0.93[0.80-1.08] | <1e-4 | 80.2 | 0.89[0.78-1.01] | <1e-4 | 64.2 | 0.93[0.84-1.03] | <1e-4 | 81.4 |
| Others | 23770/28020 | 0.90[0.81-1.01] | <1e-4 | 71.9 | 1.04[0.96-1.12] | <1e-4 | 57.3 | 1.00[0.92-1.08] | <1e-4 | 65.4 | **0.88[0.80-0.96]** | <1e-4 | 69 | 0.95[0.90-1.01] | <1e-4 | 72.3 |
| **Ethnicities** |  |  |  |  |  |  |  |  |  |  |  |  |  |  |  |  |
| Asian | 28650/31688 | **0.87(0.78-0.96)** | <1e-4 | 72.6 | 1.01(0.94-1.09) | <1e-4 | 60.1 | 0.97(0.89-1.05) | <1e-4 | 68.6 | **0.86(0.80-0.92)** | <1e-4 | 65.3 | **0.93(0.88-0.98)** | <1e-4 | 72.7 |
| Caucasians | 6997/9460 | 0.87(0.62- 1.22) | <1e-4 | 82 | 0.91(0.73- 1.14) | <1e-4 | 79.4 | 0.91(0.70-1.17) | <1e-4 | 85.9 | 0.93(0.74-1.18) | <1e-4 | 72.1 | 0.94(0.78-1.14) | <1e-4 | 87.2 |
| Africans/mixed | 973/1353 | 1.34(0.71-2.50) | 0.064 | 54.9 | 1.25(0.75- 2.07) | 0.018 | 66.4 | 1.26(0.83-1.91) | 0.037 | 60.8 | 1.22(0.61-2.43) | 0.032 | 61.9 | 1.19 (0.90-1.57) | 0.058 | 56.2 |
| **Cancer categories** |  |  |  |  |  |  |  |  |  |  |  |  |  |  |  |  |
| GI | 13585/16576 | 0.88(0.72-1.06) | <1e-4 | 80.2 | 0.98(0.86-1.11) | <1e-4 | 72.5 | 0.95(0.83-1.09) | <1e-4 | 80.3 | 0.90(0.78-1.04) | <1e-4 | 73.2 | 0.95(0.86-1.05) | <1e-4 | 82.9 |
| HNC | 4765/5546 | 1.11(0.89- 1.38) | 0.027 | 55.7 | 1.14(0.89-1.47) | 0.001 | 70.4 | 1.13(0.90-1.42) | 0.001 | 69.6 | 1.01(0.92-1.11) | 0.164 | 33 | 1.04 (0.94-1.16) | 0.031 | 54.6 |
| GyC | 1394/1573 | 0.85(0.53-1.36) | 0.042 | 56.6 | 1.02(0.66-1.58) | 0.061 | 52.5 | 0.96(0.61-1.50) | 0.030 | 59.5 | **0.80(0.68- 0.95)** | 0.346 | 10.8 | **0.88(0.79-0.98)** | 0.089 | 47.6 |
| HM | 1347/2571 | 0.71(0.34- 1.49) | 1e-4 | 80.4 | 0.91 (0.77-1.09) | 0.071 | 50.7 | 0.79(0.52-1.19) | 0.008 | 67.5 | 0.80(0.42-1.51) | 0.00 | 80.2 | 0.83(0.57-1.21) | 0.00 | 80.5 |
| UG | 2624/3119 | 1.05[0.68-1.63] | 0.001 | 72.6 | 1.23[0.93-1.63] | 0.035 | 55.5 | 1.19[0.87-1.62] | 0.006 | 66.7 | 0.91[0.64-1.28] | 0.003 | 69.4 | 1.04[0.81-1.33] | 5e-4 | 75.3 |
| **Cancer types** |  |  |  |  |  |  |  |  |  |  |  |  |  |  |  |  |
| BC | 6625/8003 | **0.79[0.66-0.96]** | 0.013 | 50.4 | 0.92[0.79-1.07] | 0.011 | 49.3 | 0.89[0.76-1.04] | 0.002 | 57 | **0.86[0.74-0.99]** | 0.040 | 42.7 | 0.91[0.82-1.01] | 7e-4 | 60.2 |
| HCC | 4355/4903 | 0.86[0.66-1.11] | 0.001 | 61.8 | 1.037[0.88-1.22] | 0.047 | 42.4 | 0.99[0.83-1.17] | 0.011 | 52.3 | 0.86[0.68-1.07] | 6e-4 | 64.1 | 0.94[0.83-1.06] | 8e-4 | 62.9 |
| GC | 4505/5874 | 0.80 (0.45-1.44) | <1e-4 | 91 | 0.81(0.55-1.20) | <1e-4 | 89.4 | 0.80(0.51-1.25) | <1e-4 | 92.7 | 0.91(0.62-1.33) | <1e-4 | 83 | 0.88(0.64-1.21) | <1e-4 | 93 |
| CRC | 2220/3680 | **0.82[0.70-0.97]** | 0.089 | 41.7 | 0.92[0.81-1.05] | 0.183 | 29.4 | 0.90[0.80-1.02] | 0.070 | 44.7 | 0.90[0.79-1.03] | 0.137 | 35.1 | 0.96[0.84-1.10] | 0.031 | 52.7 |
| LC | 4203/4677 | **0.76[0.67-0.86]** | 0.238 | 24 | **0.97[0.87-1.08]** | 0.240 | 23.7 | **0.90[0.81-0.99]** | 0.262 | 21 | **0.78[0.71-0.86]** | 0.095 | 42.4 | **0.87[0.82-0.93]** | 0.199 | 28.7 |
| PC | 540/933 | 0.96[0.67-1.37] | 0.419 | 0 | 1.21[0.41-3.58] | 0.055 | 65.3 | 1.15[0.43-3.11] | 0.063 | 63.8 | 0.91[0.72-1.13] | 0.90 | 0 | 0.99[0.83-1.17] | 0.180 | 41.6 |
| OC | 2038/2855 | 1.16(0.72-1.86) | 0.001 | 77.7 | 1.31(0.83-2.07) | 7e-4 | 79.1 | 1.25(0.79-1.96) | 2e-4 | 82 | 0.89(0.77-1.03) | 0.259 | 24.2 | 1.06(0.84-1.32) | 0.002 | 75.4 |
| OvC | 843/1100 | 0.81[0.28-2.37] | 0.023 | 68.4 | 1.03[0.38-2.78] | 0.029 | 66.6 | 0.95[0.34-2.63] | 0.017 | 70.4 | **0.73[0.60-0.90]** | 0.332 | 12.1 | 0.88[0.58-1.32] | 0.050 | 61.6 |
| ESCC | 2092/2191 | 0.87(0.41-1.82) | <1e-4 | 85.8 | 1.11(0.96-1.28) | 0.279 | 21.3 | 1.05(0.76-1.45) | 0.028 | 63.1 | 0.79(0.42-1.47) | <1e-4 | 84.1 | 0.95(0.71-1.27) | 3e-4 | 81.1 |
| Others | 7938/11167 | 1.00(0.81-1.23) | <1e-4 | 69.2 | 1.05(0.90-1.23) | <1e-4 | 63.3 | 1.04(0.88-1.23) | <1e-4 | 69.7 | 0.97(0.83-1.13) | <1e-4 | 62.2 | 1.00(0.89-1.12) | <1e-4 | 72.9 |

**a:** represents number of cases and controls in each subgroup; **b:** Pooled ORs and 95% confidence intervals; **c:** P-value of the heterogeneity test; **d:** meta-analysis of all studies excluding those with the control group not in HWE; **Abbreviations:** GI: cancers of digestive system; HNC: Head and neck carcinoma; GyC: Gynecological cancers; HM: Hematological malignancies; UG: Urogenital cancers; BC: Breast cancer, HCC: hepatocellular cancer; GC: gastric cancer; CRC: colorectal cancer; LC: Lung cancer; PC: prostate cancer; OC: Oral cancer; OvC: ovarian cancer; ESCC: esophageal squamous cell carcinoma.
